# The identification of the *Rosa S*-locus and implications on the evolution of the Rosaceae gametophytic self-incompatibility systems

**DOI:** 10.1038/s41598-021-83243-8

**Published:** 2021-02-12

**Authors:** J. Vieira, J. Pimenta, A. Gomes, J. Laia, S. Rocha, P. Heitzler, C. P. Vieira

**Affiliations:** 1 Instituto de Biologia Molecular e Celular (IBMC), Rua Alfredo Allen, 208, 4200-135 Porto, Portugal; 2 Instituto de Investigação e Inovação em Saúde (I3S), Universidade do Porto, Rua Alfredo Allen, 208, 4200-135 Porto, Portugal; 3 Institut de Biologie Moléculaire Des Plantes, CNRS, Université de Strasbourg, UPR 2357, 67000 Strasbourg, France

**Keywords:** Evolution, Genetics, Plant sciences

## Abstract

In Rosaceae species, two gametophytic self-incompatibility (GSI) mechanisms are described, the *Prunus* self-recognition system and the Maleae (*Malus/Pyrus/Sorbus*) non-self- recognition system. In both systems the pistil component is a *S-RNase* gene, but from two distinct phylogenetic lineages. The pollen component, always a F-box gene(s), in the case of *Prunus* is a single gene, and in Maleae there are multiple genes. Previously, the *Rosa S*-locus was mapped on chromosome 3, and three putative *S-RNase* genes were identified in the *R. chinensis* ‘Old Blush’ genome. Here, we show that these genes do not belong to the *S*-locus region. Using *R. chinensis* and *R. multiflora* genomes and a phylogenetic approach, we identified the *S-RNase* gene, that belongs to the *Prunus S*-lineage. Expression patterns support this gene as being the *S*-pistil. This gene is here also identified in *R. moschata*, *R. arvensis*, and *R. minutifolia* low coverage genomes, allowing the identification of positively selected amino acid sites, and thus, further supporting this gene as the *S-RNase*. Furthermore, genotype–phenotype association experiments also support this gene as the *S-RNase*. For the *S*-pollen GSI component we find evidence for multiple F-box genes, that show the expected expression pattern, and evidence for diversifying selection at the F-box genes within an *S*-haplotype. Thus, *Rosa* has a non-self-recognition system, like in Maleae species, despite the *S*-pistil gene belonging to the *Prunus S-RNase* lineage. These findings are discussed in the context of the Rosaceae GSI evolution. Knowledge on the *Rosa S*-locus has practical implications since genes controlling floral and other ornamental traits are in linkage disequilibrium with the *S*-locus.

## Introduction

Gametophytic self-incompatibility (GSI), the most common pre-zygotic self-incompatibility genetic mechanism, prevents self-fertilization between genetically related individuals, where the genotype of the haploid pollen determines its incompatibility type^1^. This mechanism is determined by a single locus, the *S*-locus, and in the most frequent eudicot system^2^, the *S*-pistil gene codes for a protein with ribonuclease activity, called *S-RNase*^3–5^, and the S-pollen gene (s) code(s) for a F-box protein(s)^6–26^. In incompatible crosses, the cytotoxic S-RNases lead to pollen tube RNA degradation, that causes the cessation of pollen tube elongation. Two mechanisms of pollen recognition have been proposed, the self and non-self-recognition systems^6–26^. In the self-recognition mechanism, described in the *Prunus* genus of the Rosaceae family^6, 7, 11, 17, 24, 27^, only one F-box protein is the *S*-pollen GSI specificity determinant, called SFB, that interacts with the self S-RNase (reviewed by^28^). In this system, as expected, the *S-RNase* and *SFB* genes show patterns of co-evolution^29^. In the non-self-recognition mechanism, characterized in Solanaceae, Plantaginaceae, and in the Rosaceae Maleae tribe (*Malus*/*Pyrus*/*Sorbus*), there are multiple F-box proteins, called SLFs in Solanaceae and Plantaginaceae, and SFBBs in Maleae^13–16, 18–26, 30–32^, that interact with all S-RNases but the self-S-RNase^15, 16, 18, 20–26, 30–32^. Even though different recognition interactions occur due to different evolutionary paths, the S-RNase retained the same feature of cytotoxicity to self-pollen in both mechanisms^28, 30^.

Based on phylogenetic inferences, the *S-RNase* GSI system evolved once in core eudicots^33–37^, and has been the subject of multiple duplication events during evolution. In Rosaceae, different duplicates have been recruited for GSI function in *Prunus* and *Malus/Pyrus/Sorbus*^24, 37^. Convergent evolution has also been proposed for the evolution of the *S*-pollen determinant since the *Prunus S*-pollen gene, belongs to a different gene lineage than that of the *Malus/Pyrus/Sorbus S*-pollen genes. Therefore, it is not surprising the presence of different recognition systems in *Prunus* (self-recognition^6, 7, 11, 12, 17, 24, 27, 28, 38^) and in *Malus/Pyrus/Sorbus* (non-self-recognition mechanism^13, 16, 18–20, 24, 25^). How the two systems evolved is still debated. The observation that *Prunus S-RNase* and *SFB* lineage genes are present in *Fragaria* species (an outgroup), suggests that the ancestral Rosaceae *S*-locus was of the self-recognition type, and that the *Malus S*-locus region has a “de novo” evolution^24^. An alternative hypothesis postulates that the divergence between *SFB* and *SFBB* genes occurred early in the establishment of eudicots, and that *Prunus* species started using *SFB* gene as a *S*-pollen factor around the time of the *Prunus* divergence^39^*.* Under this evolutionary hypothesis, the common ancestor of *Prunus* and *Malus*/*Fragaria* would present a non-self-recognition mechanism. In this work, by characterizing the *Rosa S*-locus region we clarify the Rosaceae *S-*locus ancestral state.

Rose (*Rosa sp*., Rosaceae) species are known by their ornamental value and the production of essential oils used in perfume and cosmetic industry. There are about 200 species in this genus, but only 10 species (*R. canina*, *R. chinenesis* ‘Old Blush’ OB), *R. foetida*, *R. gallica*, *R. gigantea*, *R. moschata*, *R. multiflora*, *R. phoenicia*, *R. rugosa*, and *R. wichuraiana* ‘Basye’s Thornless’) have contributed for most of the modern species, throughout processes of hybridization and polyploidization^40^. Based on the absence of seed set after self-pollination, the presence of a GSI system has been postulated for most *Rosa* diploid species^41–43^. Analyses of one of the *R. chinensis* genomes^44^*,* revealed three putative *S-RNase* genes (*SRNase26*, *SRNase30*, and *SRNase36*) located on chromosome 3, as a candidate region of the *Rosa S*-locus^44^. In this work we show that this region is not the *S*-locus. This is the reason why segregation analyses using *SRNase30* as a marker, shows co-segregation with the *S*-locus at a distance of 4.2 cM^44^. Nevertheless, these segregation analyses showed that the *S-*locus region is located on chromosome 3, as previously reported^45^. According to chromosome location, we also exclude a putative *S-*pollen gene –*RrSLF*, cloned from *R. rugosa* pollen RNA, that in phylogenetic analyses clusters with *Prunus SFB*^46^, since the orthologous *R. chinensis* gene is located on chromosome 2.

*Rosa* diploid genomes are of relatively small size (560 Mb^47, 48^), and for *R. chinensis* ‘Old Blush’^44, 49^, and *R. multiflora*^50^ there are assembled genomes publicly available (https://www.ncbi.nlm.nih.gov/sra). Here, we use these datasets to perform phylogenetic analyses to identify the *Rosa S-*locus genes. Transcriptomic analyses using *R. chinensis* pistil and ovary, stamen, leaf, stem, and root tissue (available in SRA database), support the identification of the *Rosa S*-locus genes. To show that the *Rosa S-RNase* gene shows evidence of positive selection, we used low coverage *Rosa* genomes (*R.* *moschata, R.* *laevigata, R.* *rugosa*, *R. persica*, *R.* *xanthina, R. minutifolia, R. odorata, R. arvensis,* and *R.* *majalis*) available at SRA database, to identify further *S-RNase* alleles. Furthermore, we use *R. arvensis* genotype–phenotype association experiments to confirm that the identified gene is the *S-RNase*. We also identify F-box genes in the vicinity of the *S-RNase*, to determine which GSI system is present in *Rosa*. Expression, phylogenetic analyses, polymorphism levels, and evidence for selection favoring diversification of F-box genes within an *S*-haplotype, suggest multiple F-box genes as the *S*-pollen component. Rosaceae GSI evolution is discussed in the context of these findings.

## Results

### *R. chinensis and R. multiflora* Rosaceae* S-RNase* lineage genes

**Figure 1:**
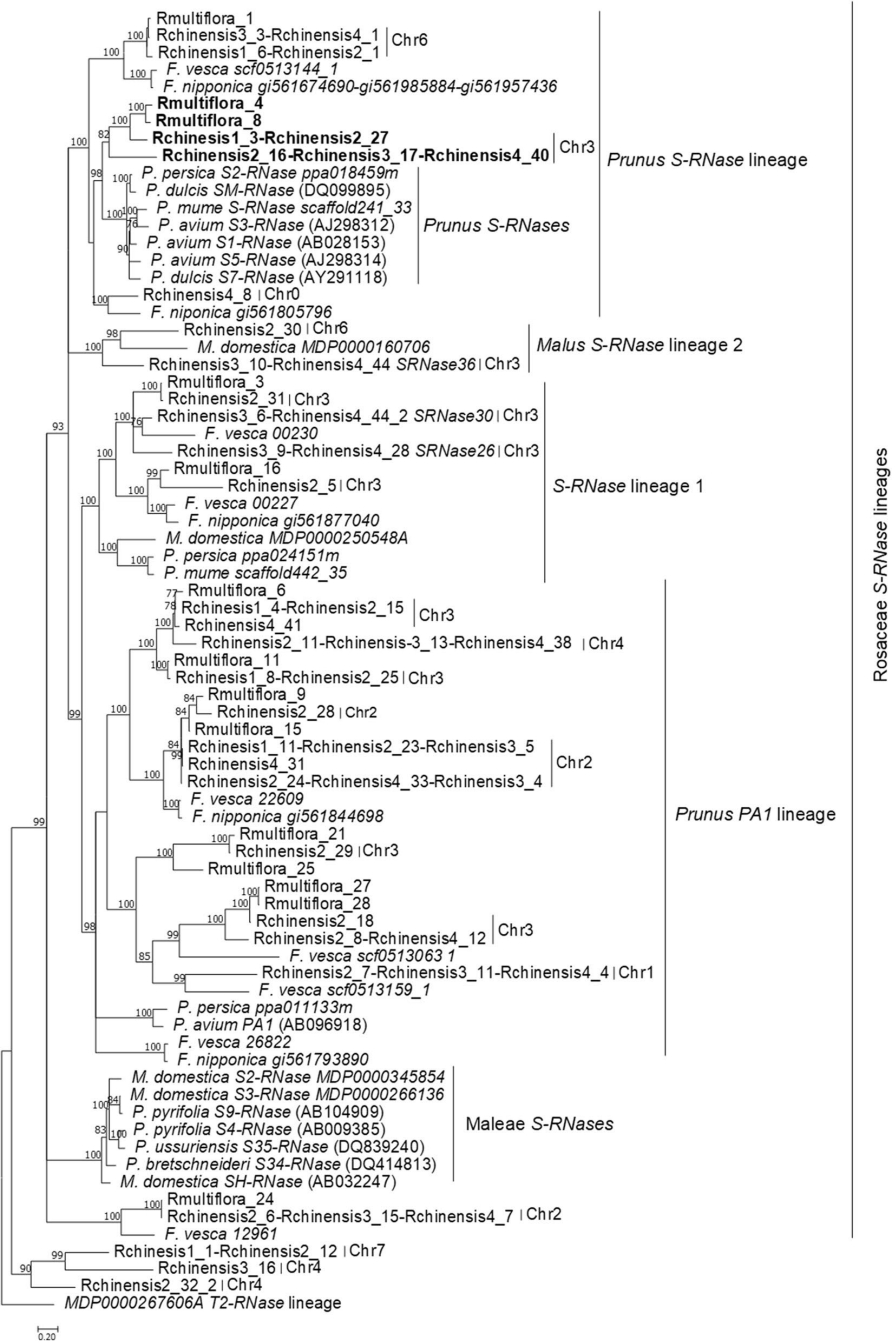
Bayesian phylogenetic tree, showing the relationship of the *R. chinensis and R. multiflora S-RNase* like sequences with *Fragaria, Prunus*, and *Malus S-RNase* lineage genes. For the *R. chinensis* sequences the chromosomal (Chr) location is given. The tree was rooted with *MDP0000267606A T2- RNase,* not involved in GSI^24^. In bold are the *Rosa* sequences that cluster with *Prunus S-RNases*, that could represent the *S-RNase* gene. Numbers below the branches represent posterior credibility values above 70.

*S-RNase*s code for basic proteins (isoelectric point (IP) above 8)^5, 24, 33, 34, 37^, that present two conserved amino acid patterns (pattern 1 and 2^36^), are expressed in pistils (where the rejection of self-pollen occurs during the growth of pollen tubes), stigma, and flowering buds, and present signs of diversifying selection^5, 24, 33, 34, 36, 37^. Of the 80 *S-RNase* like sequences identified in *R. chinensis* (55) and *R. multiflora* (25) genomes (Table 1; Supplementary Table S1), 41 code for putative proteins with a IP above 7 (Table 1; Supplementary Table S2). Phylogenetic analyses of these sequences*,* and 34 reference sequences from^24^ (Supplementary File S1), revealed that the sequences labeled Rchinensis3_16, Rchinensis2_32_2, and Rchinensis1_1-Rchinensis2_12, do not belong to the Rosaceae *S-RNase* lineage (Fig. 1). None of the *Rosa S-RNase* lineage sequences cluster with *Malus/Pyrus S-RNases*. Two groups of sequences Rmultiflora_4, Rmultiflora_8, and Rchinensis1_3-Rchinensis2_27 (gene 1), and Rchinensis2_16-Rchinensis3_17-Rchinensis4_40 (gene 2), cluster with *Prunus S-RNases* (Fig. 1), suggesting that one of them may represent the *Rosa S-RNase* gene. In *R. chinensis* these sequences are located on chromosome 3, where the *S-*locus has been identified^44, 45^. These sequences code for putative proteins with IP above 8, they have two putative introns, and amino acid pattern 2 conserved, features typical of *S-RNases*. Gene 1 sequences also present high levels of synonymous polymorphism (27.9%) as the *S-RNase*s^36^. Although in this group of sequences pattern 1 shows a violation at the first pattern position (Y instead of [FST]), an identical pattern is observed in Solanaceae for the *S-RNase Nicotiana tomentosiformis* XP_018625910_1 (this sequence clusters with other Solanaceae *S-RNases* in phylogenetic analyses; data not shown). Because gene 2 is represented by one sequence, we cannot address levels of diversity. Nevertheless, the presence of identical sequences in the two *R. chinensis* genomes analyzed, suggests low levels of diversity. The putative *SRNase26, SRNase30,* and *SRNase36* present IP below 8, and the first two sequences cluster with *Prunus* and *Malus S-*lineage 1 genes, and *SRNase36* clusters with the *Malus S-*lineage 2 gene. In *Prunus* and *Malus* GSI, these sequences are not involved in specificity determination.

**Table 1:** Summary of the *S-RNase* like sequences identified in the *R. chinensis,* and *R. multiflora* genomes.

*Rosa* genome	Number of sequences						
	Total	Excluding pseudogenes	Presenting				
			Both motifs	Both motifs and IP ≥ 7	Only 2nd motif	Only 2nd motif and IP > 7	Only 1st motif
*R. chinensis* Assembly-CDS (Rchinensis1)	12	12	10	5	−	−	−
*R. chinensis* Assembly-ORF (Rchinensis2)	33	26	26	19	−	−	−
*R. chinensis* GDR-CDS (Rchinensis3)	18	18	17	11	−	−	−
*R. chinensis* GDR-ORF (Rchinensis4)	44	22	22	13	−	−	−
*R. multiflora*	34	25	18	14	4	4	3

### Rchinensis1_3–Rchinensis2_27 is expressed in pistil and ovary tissue, like the *S-RNases*

**Figure 2:**
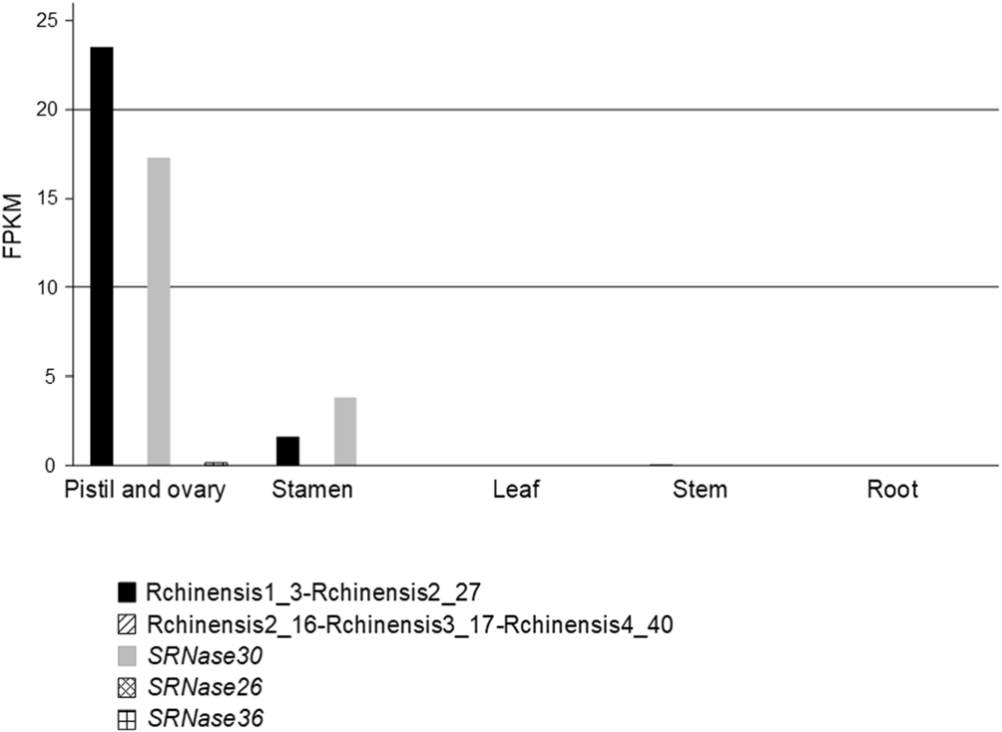
*R. chinensis* expression levels (FPKM) for the two *Prunus S-RNase* lineage genes, as well as the three previous putative *S-RNases,* in pistil and ovary, stamen, leaf, stem, and root tissues.

The *S-RNase* gene is highly expressed in pistils, in stigmas and styles of flowers at anthesis, but also shows low expression in entire flower buds. It should be noted that *S-RNase* lineage genes, can also show a similar pattern of expression, since this expression is inferred to be the ancestral expression of the *S-RNase* lineage genes^24^. Therefore, it is not surprising that *SRNase30,* and *SRNase36*, show expression in pistil and ovary (Fig. 2), as previously reported^44^. *SRNase26* shows no expression in the tissues here analyzed, as well as those used in^44^. Rchinensis1_3-Rchinensis2_27, shows a similar expression to *SRNase30*, but with high levels of expression in pistil and ovary, and low expression in stamen (Fig. 2). Rchinensis2_16-Rchinensis3_17-Rchinensis4_40 is not expressed in the tissues here analyzed. These results are compatible with Rchinensis1_3-Rchinensis2_27 gene, being the *S-*pistil gene determining GSI specificity.

### *Rosa S-RNases* show evidence for positively selected amino acid positions

**Figure 3:**
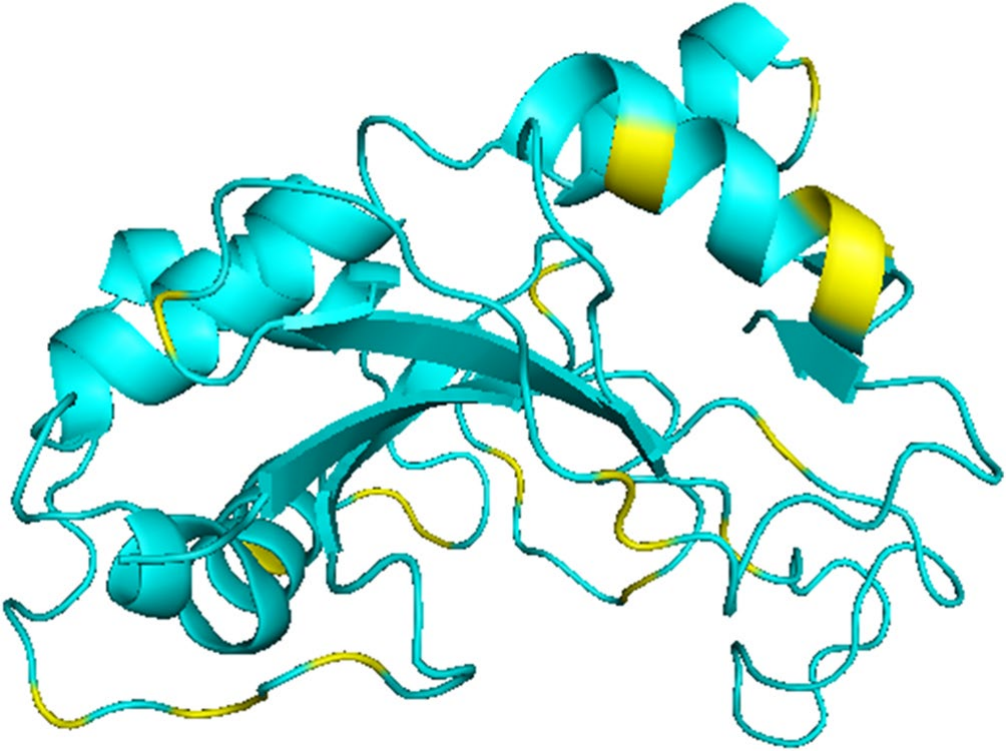
Positively selected amino acid sites, highlighted in yellow on the predicted 3D structure of the *R. chinensis* S-RNase.

To address if the *Rosa* sequences here identified as the *S-*locus pistil gene are the subject of diversifying selection, a feature of the *S-RNase* gene, we first identified and annotated sequences similar to *S-RNases* from other self-incompatible *Rosa* genomes with low coverage (*R.* *moschata, R.* *laevigata, R.* *rugosa*, *R. persica*, *R.* *xanthina, R. minutifolia, R. odorata*, *R. arvenses*, and *R.* *majalis;* Supplementary Table S1). Phylogenetic analyses using sequences presenting an IP above 7 covering at least the exon where motif 2 is located (Supplementary Fig. S1A,B; Supplementary File S2), together with the *Rosa S-RNase* sequences here identified, and the reference sequences from^24^, revealed four additional *Rosa S-RNases* (Rarvensis_11, Rminutifolia_20, Rmoschata17_14, and Rmoschata17_30; Supplementary Fig. S1A,B). Similar analyses with the sequences covering the motif 1, revealed four sequences (Rodorata07_39; Rmoschata08_12; Rminutifolia_7; and Rarvensis_6; Supplementary Fig. S2; Supplementary File S3) that also cluster with *Rosa S-RNases* sequences. Because the phylogenetic relationship of sequences Rarvensis_11 and Rarvensis_6 is similar, they may represent two exons of the same gene from the same *S*-haplotype, and thus were treated as such. The same applies to sequences Rminutifolia_20 and Rminutifolia_7, as well as sequences Rmoschata17_14 and Rmoschata17_34. Using these sequences together with Rchinensis1_3-Rchinensis2_27, Rmultiflora_4, and Rmultiflora_8, we identify 21 amino acid sites under positive selection (results available at http://bpositive.i3s.up.pt/ in the project named *Rosa S*-locus genes; BP2018000004) by performing codeML^51^ analyses. These amino acid sites are, in principle, responsible for GSI specificity^24, 35, 52^.The location of these sites at the predicted 3D structure is mostly around the active site pocket region (Fig. 3), as observed in other Rosaceae and Solanaceae species^25, 52^.

### *R. arvensis* genotype–phenotype association experiments

For 12 *R. arvensis* accessions, obtained from a breeding program of siblings^53^, *S-*haplotype was deduced either through hand-pollinated tests (Supplementary Fig. S3A,B), allowing to perform co-segregation experiments of the *S2*-allele. An amplification product with the expected size (300 bp; see Material and Methods) was obtained in six (Ose (*S*1 *S*2), Url (S4 *S*5), E200 (Ose x Wid (*S*3 *S*6); *S*2 *S*3), E404 (E200 x Ose; *S*1 *S*2), E435 (E200 x Wid; *S*2 *S*6), and E893 (E459 x Url; *S*1 S5) individuals. The sequence of these amplification products, reveled two types of identical sequences. One obtained from Ose, E200, E404, and E435 individuals, those individuals having the *S2-RNase* (GenBank acc. Numbers MW452856–MW452859). The other sequence type was obtained from Url and E893, those individuals having the *S5-RNase* (GenBank acc. numbers MW452860 and MW452861). These results support co-segregation of the *S*-locus with the *S2-RNase* genotypes here surveyed. Therefore, the *S-RNase* gene here identified is on the *S*-locus region.

### Identification of the *Rosa S*-pollen F-box genes

The *R. rugosa S*-locus F-box gene (*RrSLF*; KY446808), expressed in pollen tissue and phylogenetically related with *Prunus SFB* gene, was reported as a putative *Rosa S-*pollen gene^46^. It should be noted that other F-box genes not involved in *S*-pollen specify determination are also expressed in pollen, as well as in other tissues^24^. The expression of *RrSLF* gene in other tissues has not, however, been addressed^46^. This gene has 99% homology at nucleotide level with a *R. chinensis* sequence (CM009583.1) located on chromosome 2, but the *Rosa S-*locus is located on chromosome 3 (^44, 45^, and this work). Furthermore, all *S*-pollen genes described in Rosaceae, Solanaceae, and Plantaginaceae are intronless genes, and the *R. chinensis RrSLF* (PRQ51373) and *R. multiflora* (Rmu_sc0016061.1;BDJD01015883.1) orthologous genes have one intron. The orthologous gene has been identified in all low coverage *Rosa* genomes, except *R. majalis*, covering the entire coding region. Low levels of divergence (0.036 for synonymous and 0.009 for non-synonymous divergence respectively, after Jukes and Cantor correction; N = 10) are obtained for this gene. This is in contrast with the *Prunus SFB* gene, that presents levels of variability above 20%^6, 7, 11, 36^. Therefore, *RrSLF* is not the *S*-pollen gene.

**Figure 4:**
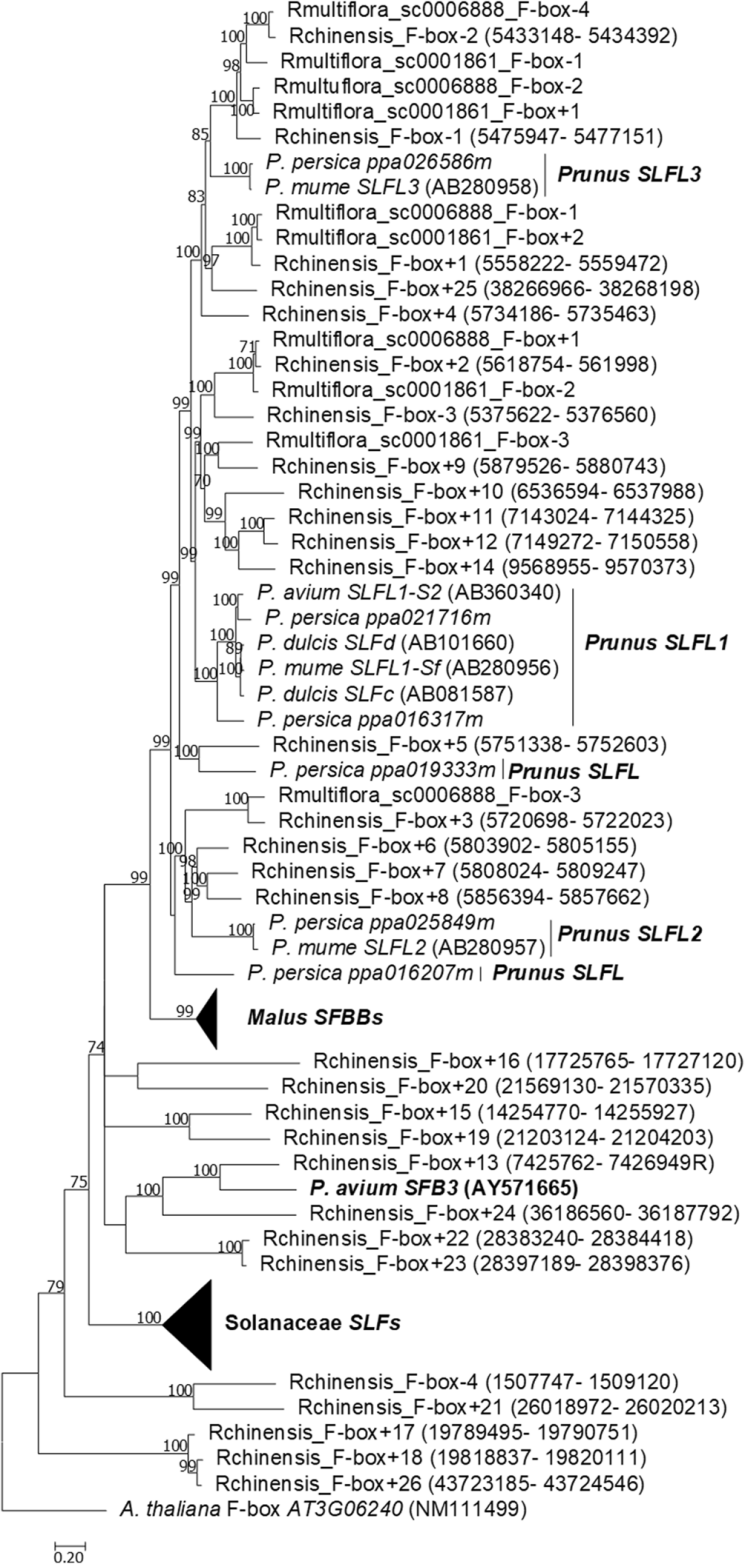
Bayesian phylogenetic tree, showing the relationship of the *SFB/SLFL/SFBB* like genes (called Fbox − and + according to the 5′ or 3′ position relative to the *S-RNase*) from *R. chinensis* chromosome 3, *R. multiflora* sc0006888, and *R. multiflora* sc0001861, with *M. domestica SFBBs, Prunus SFBs* and *SLFLs*, and Solanaceae *SLFs*. The tree was rooted with *A. thaliana* F-box/kelch-repeat, not involved in GSI. Numbers below the branches represent posterior credibility values above 70.

**Figure 5:**
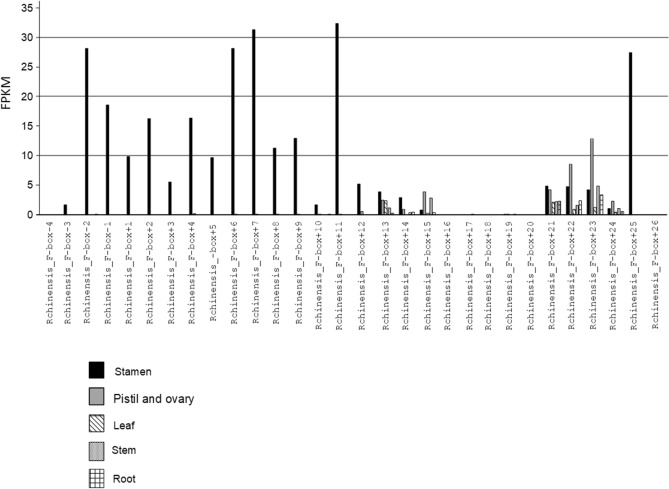
*R. chinensis* expression levels (FPKM) for the F-box genes located on chromosome 3, in pistil and ovary, stamen, leaf, stem, and root tissues.

In *R. chinensis* chromosome 3 there are 30 *SFB/SLFL/SFBB* like genes (called Fbox − and + according to the 5′ or 3′ position relative to the *S-RNase*, respectively; Supplementary Table S3; Supplementary File S4). In the two *R. multiflora* scaffolds where the *S-RNase* is located, there are five such genes (Supplementary Table S3). The phylogenetic analyses of the *R. chinensis* and *R. multiflora SFB/SLFL/SFBB* like genes together with *Prunus SFB* and *SLFL* genes, *M. domestica S1-SFBB*s, *Petunia* and *Nicotiana SLF* sequences, and *A. thaliana* F-box/kelch-repeat, shown in Fig. 4 (Supplementary File S4), revealed that the Rchinensis_F-box + 13 gene clusters with *Prunus SFB* gene. This gene is expressed in all tissues here analyzed (Fig. 5), but the *S*-pollen gene(s) are mainly expressed in anthers /pollen only^7, 10, 12–16, 21, 23–25^, and thus is unlikely to be the *S*-pollen gene. Furthermore, using a 1188 bp gene region obtained from eight *Rosa* genomes (*R. arvensis*, *R. laevigata*, *R. moschata*, *R. xantina*, *R. rugosa*, *R. odorata*, *R. multiflora*, and *R. chinensis*), low average levels of divergence (0.0321 for synonymous and 0.0128 for non-synonymous divergence respectively, after Jukes and Cantor correction) are observed. Moreover, this gene is the neighbor F-box gene of the *T2-RNase* Rchinensis1_8-Rchinensis2_25, that does not cluster with *Prunus* or Maleae *S-RNases* (Fig. 1). Therefore, Rchinensis*_*F-box + 13 gene is not involved in *S*-pollen GSI specificity determination.

**Figure 6:**
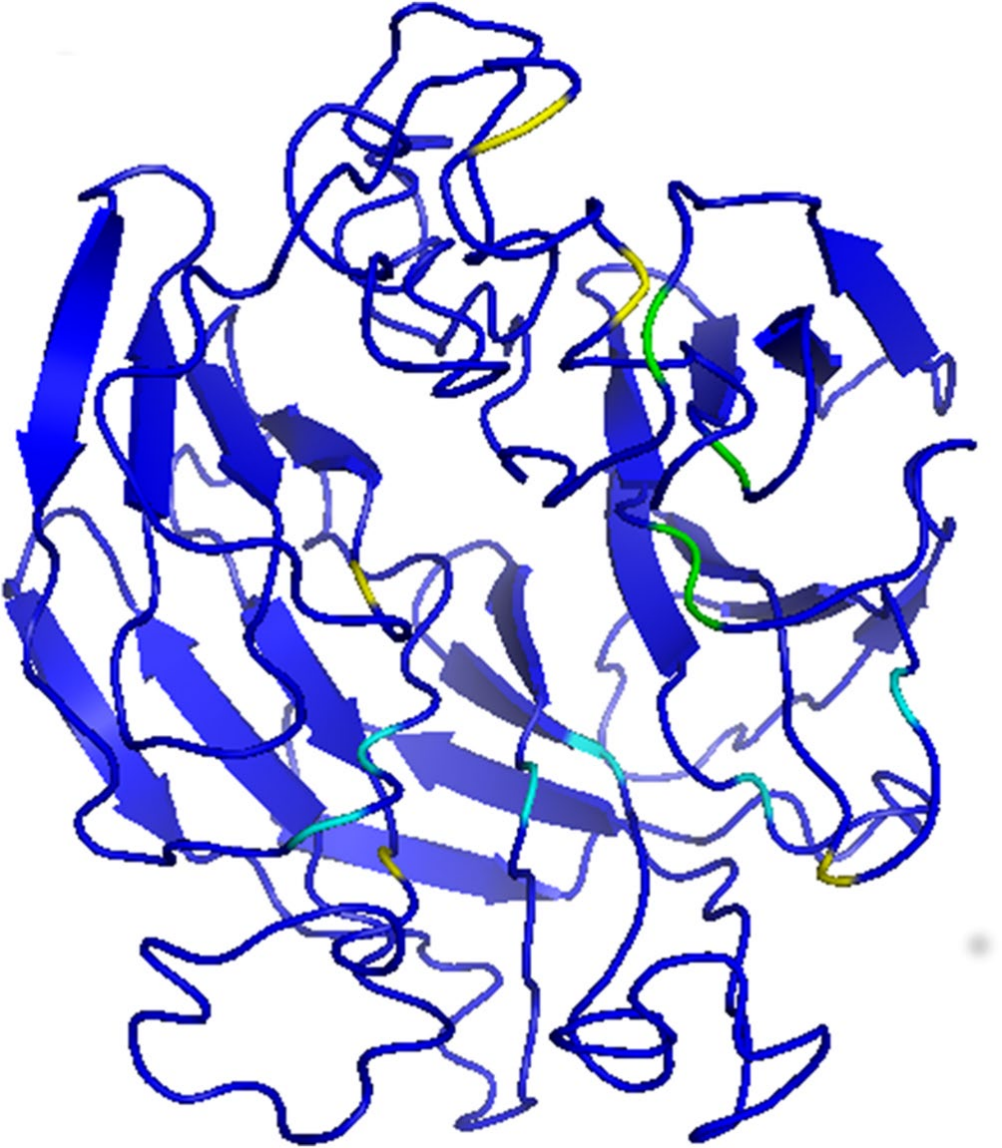
Positively selected amino acid sites on the predicted 3D structure of the *R. chinensis* F-box located in the 5` region of the *S-RNase*. In yellow are highlighted those sites that appear as positively selected in the two datasets analyzed (14 and 13 *R. chinensis* F-box genes in the vicinity of the *S-RNase* that are expressed in stamen), in green those that are located in a region not analyzed when 14 F-box genes are considered, and in blue those that change due to alignment gaps in the two datasets analyzed.

The F-box genes in the vicinity of the *R. chinensis* and *R. multiflora S-RNase* gene cluster, with high support, with different *Prunus SLF* genes (Fig. 4), that are a sister group of *Malus SFBB*s^24^. In *R. chinensis* 14 of these genes (Rchinensis*_*F-box-3 up to Rchinensis_F-box + 11) show expression in stamen only, compatible with being *S*-pollen genes (Fig. 5). Furthermore, the *R. multiflora* orthologs of *R. chinensis* are not in the same order relatively to the *S-RNase* gene (Fig. 4), showing that this region is highly rearranged, as observed in the *Malus/Pyrus/Sorbus S*-locus region^13, 16, 18–20, 24, 25^. This is surprising since we identified the *Rosa S-RNase* as belonging to the *Prunus S-*lineage, and in *Prunus* there is a single *S*-pollen gene*.* In *Prunus* the *S*-pollen gene presents levels of diversity similar to the *S-RNase* gene^11, 29^. Therefore, we also determined levels of synonymous and non-synonymous divergence for the F-box genes surrounding the *R. chinensis S-RNase* (Rchinensis_F- box-1 and Rchinensis_F- box + 1, used as query in a blastn to identify contigs containing the orthologous genes in the low coverage *Rosa* genomes). The low levels of synonymous and non-synonymous divergence for Rchinensis_F-box-1 (0.070 and 0.005 respectively), and Rchinensis_F- box + 1 (0.112 and 0.02129 respectively) are incompatible with the hypothesis that one of them is determining *Rosa S*-pollen specificity. Therefore, multiple F-box genes must be involved in *Rosa* pollen GSI specificity determination, as in *Malus/Pyrus/Sorbus* and Solanaceae species. Using the 14 *R. chinensis* F-box sequences in the vicinity of the *S-RNase*, that are expressed in stamen, we find evidence for positively selected amino acid sites*,* as expected if these genes are involved in *S*-pollen specificity determination (results available at http://bpositive.i3s.up.pt/ under project Rosa *S*-locus genes; BP2018000004). On the predicted 3D structure, these amino acid sites are located in the same regions (Fig. 6) as those observed for *Petunia S-*pollen genes^26^. Evidence for positive selection is also observed for the five F-box genes of the *R. multiflora* scaffold sca0006888 (results available at http://bpositive.i3s.up.pt/ under project Rosa *S*-locus genes; BP2018000004). Therefore, the data suggests that *Rosa S*-pollen specificity is determined by multiple F-box genes, like in *Malus/Pyrus/Sorbus* and Solanaceae species.

## Discussion

In *Rosa*, there are very important traits of horticultural interest such as flower development, architecture, senescence, scent biosynthesis and emission, ease of reproduction, and resistance to biotic and abiotic stresses, that have been selected only once during the history of rose selection, and incorporated into many rose varieties^44, 45, 48^. Indeed, only 10 species have contributed to the genetic make-up of most of the modern rose cultivars, and some old and popular cultivars, such as ‘Old Blush’ have dominated the history of rose selection^40^. This Chinese rose from the Song dynasty (960–1279) conveys several desirable characters such as recessive reblooming habit, recessive lack of prickles (stem) and dominant flower doubleness^44, 45, 48^, all co-segregating with the *S-*locus region. The characterization of the *S-*locus region here performed is thus, very important in order to help breeding selection, and the control of genetic diversity.

The *Rosa S*-locus is composed of a *S-RNase* gene that belongs to the *Prunus* lineage (Fig. 1). This gene shows all expected features of a *S*-pistil gene since it shows expression in pistil and ovary (Fig. 2), evidence for diversifying selection (Fig. 3), and co-segregation with the *S*-locus. In *Fragaria*, that also belongs to the Rosoideae subfamily (the two genera have been diverging for about 50 million years^54^), a *Prunus* lineage *S-RNase* gene has been also reported as the putative *S*-pistil gene^24^. This suggests that the Rosoideae GSI system could be of the self-recognition type, with one *S*-pollen gene, as in *Prunus* (^24^ and references therein). Nevertheless, the *Rosa* F-box gene that clusters with the *Prunus SFB* gene (Fig. 4) is not the neighbor of the *S-RNase* gene, as observed in *Prunus*, and presents expression (Fig. 5) and polymorphism levels incompatible with being the *S-*pollen gene. In *Rosa* there are multiple F-box genes in the vicinity of the *S-RNase*, that show expression compatible with being the *S-*pollen gene (Fig. 5), and evidence for diversifying selection for F-box genes within a *S*-haplotype (Fig. 6). These are the expected features of *S*-pollen genes in a non-self-recognition system, as reported in *Malus/Sorbus/Pyrus*^13, 16, 18–20, 24, 25^ and Solanaceae species^15, 22, 23, 26, 30–32^. Therefore, the *Rosa S*-locus region encompasses a large region, as reported for other species presenting non-self recognition systems, and this could explain why in rose several traits of horticultural interest are linked to the *S-*locus. It should be noted that the *Rosa S*-locus region may be highly rearranged, since we could not align the contig containing the *S-RNase* gene from *R. chinensis* with those from *R. multiflora* as well as the two contigs containing the *S-RNase* gene from *R. multiflora*. This observation has been previously reported for the two available *R. chinensis* genomes, that could not be aligned in this region (conceivably carrying two different *S*-haplotypes; see Fig. 1 of^55^). Pollen transcriptome analyses of multiple *S*-haplotypes are, however needed to determine how many F-box genes in the vicinity of the *Rosa S-RNase* are involved in pollen GSI specificity, as performed in *Malus*^25^ and *Petunia*^21^.

Since multiple *S-*pollen genes are determining *Rosa S*-pollen specificity, as in Maleae species, this suggests that the ancestral Rosaceae GSI system was of the non-self-recognition system type. This implies that during evolution the *Malus S-RNase* lineage, that does not cluster with the *Rosa S-RNase*, has been recruited de novo from a duplicate of the ancestral *S-RNase* gene. Moreover, it implies that the *Prunus S*-pollen gene, that does not cluster with *Rosa S*-pollen genes, evolved de novo from an unrelated F-box gene.

Low levels of variation have been reported in roses but the *S*-locus region, being large and under balancing selection, may help retain a substantial fraction of the variability. Indeed, the evolution within the genus *Rosa*, occurred via interspecific hybridization, allopolyploidization and genetic reticulation among sympatric species^56^. In Europe, the recent post-glaciation period expedited the process of speciation, as the northward extension of the biotopes increased. The diploid *R. arvensis* holds a special place in this process, since, although it belongs to the synstylae group, has a substantial genomic promiscuity with the polyploid caninae group^56, 57^, that may also help maintain diversity levels. The inbred strains that are being established for *R. arvensis*^53^ are a starting point to investigate the diversity of the *S*-locus in European wild roses. Mutants breaking down self-incompatibility, will also help refine the *S-*locus effect on roses molecular diversity.

## Methods

### Identification of putative *S-RNase* like sequences in *Rosa* genomes

The annotations (CDS) of two *R. chinensis* genomes (available at NCBI RefSeq database (www.ncbi.nlm.nih.gov) and GDR database (https://www.rosaceae.org) Supplementary Table S1) were downloaded, and the sequences showing similarity with reference *S-RNases* assigned as Rchinensis1 and Rchinensis3, respectively. Moreover, the corresponding genome sequences were downloaded as a FASTA file, and the *S-RNase* sequences here annotated labeled as Rchinensis2 and Rchinensis4, respectively. *R. multiflora* genome (NCBI assembly database, Supplementary Table S1) was also downloaded as a FASTA file, since annotations are not available for this species. To find and extract protein encoding segments larger than 100 bp, we have used getorf, using the emboss Docker image available at pegi3s Bioinformatics Docker images project (htttps:pegi3s.github.io/dockerfiles). Then, we selected the protein encoding segments that show similarity with reference *S-RNase* sequences, using tblastx (Expect value (e) < 0.05), as implemented in SEDA^58, 59^, using as query *Prunus S3-RNase* (AJ298312), *Malus Sh-RNase* (AB032247), and *Fragaria* putative *S-RNase* (gi561957436, gi561674690 and gi561985884)^24^. Based on this information, we manually annotated the corresponding genome region to identify the exons of each gene. For each putative gene we obtained the corresponding amino acid sequence to calculate IP, using ExPASy software^60^.

We used the same protocol for the genomes here assembled using the short reads of nine *Rosa* genomes downloaded from NCBI SRA database (Supplementary Table S1). In this case, we used FastQC to evaluate read quality, and Cutadapt to trim reads^61^, and ABySS 2.0^62^ for the de novo assembly, using the Docker images available at pegi3s Bioinformatics Docker images project (htttps://pegi3s.github.io/dockerfiles).

### Identification of the F-box genes located in *R. chinensis* chromosome 3 and *R. multiflora* scaffolds where the *S-RNase* is located

The protocol presented for the *S-RNase* like sequences was also used to obtain F-box genes located in *R. chinensis* chromosome 3, and for the two *R. multiflora* scaffolds where the *S-RNase* gene has been identified, using as query *P. avium SFB3* (AAT72121.1), *P. avium SLFL1* (BAG12295.1), and *M. domestica SFBB3-beta* (BAF47180.1).

### Phylogenetic analyses

Phylogenetic analyses of the *R. chinensis* and *R.multiflora S-RNase* like sequences and F-box like genes were performed using sequences aligned with MUSCLE alignment algorithm, as implemented in ADOPS^63^. Only codons with a support value above 2 were used for phylogenetic reconstruction. Bayesian trees were obtained using MrBayes 3.1.2^64^ as implemented in the ADOPS pipeline^63^. The Generalised Time-Reversible (GTR) model of sequence evolution was implemented in the analyses, allowing for among-site rate variation and a proportion of invariable sites. Third codon positions were allowed to have a gamma distribution shape parameter different from that of first and second codon positions. Two independent runs of 1,000,000 generations with four chains each, were carried out. The average standard deviation of split frequencies was always ~ 0.01 and the potential scale reduction factor for every parameter was ~ 1.00, showing that convergence was achieved. Trees were sampled every 100th generation and the first 5000 samples were discarded (burn-in). The tree was converted to Newick format using the Format Conversion website (http://phylogeny.lirmm.fr/phylo_cgi/data_converter.cgi) and edited using Mega7^65^.

The phylogenetic analyses of the low coverage *Rosa* genomes (Supplementary Table S1) sequences were performed with Mega7^65^, using ClustalW alignment algorithm, Neighbor-Joining method, bootstrap test with 10,000 replicates, the p-distance method for computing the evolutionary distances, and pairwise deletion since sequences can have different sizes (Supplementary Table S2).

### Expression of *R. chinensis S-RNase* like, and F-box genes located on chromosome 3 in pistil and ovary, stamen, leaf, stem and root transcriptomes

To estimate expression of the *Rosa S- RNase* like sequences located on chromosome 3, we use RNA-seq data from *R. chinensis* pistil and ovary, stamen, leaf, stem, and root transcriptomes (Supplementary Table S4). We used FastQC to evaluate read quality, and Cutadapt to trim reads^61^. FPKM values were estimated using the RSEM method, as implemented in Trinity^66^, using the *R. chinensis* Refseq CDS, and the *R. chinensis S-RNas*e like and F-box sequences located on chromosome 3.

### *R. arvensis* genotype–phenotype association experiments

Genomic DNA was extracted from leaves of 12 *R. arvensis* individuals, for which the haplotypes were predicted according to their parents and progeny (Supplementary Fig. S3A,B), using the method of^67^. PCRs were performed using the genomic DNA and primers RA-F 5′ GGAAGCCARACTGAAGAT 3′ and RA-R 5´AGCATCACAGTYTCGATCA 3′, designed for conserved regions of the putative *Rosa S-RNase* sequences here identified. Standard amplification conditions were 35 cycles of denaturation at 94 °C for 30 s, 52 °C for 30 s for primer annealing, and primer extension at 72 °C for 2 min. The amplification products with the expected size (353 bp) for individuals Ose, E200, E404, Url, and E893 were cloned using the TA cloning kit (Invitrogen, Carlsbad, CA). For each individual, the insert of 16 colonies was cut separately with *Dde*I, and *Hinf*I restriction enzymes, and only one restriction pattern was observed, and thus three colonies only were sequenced. The ABI PRISM BigDye cycle-sequencing kit (Perkin Elmer, Foster City, CA), and specific primers, or the primers for the M13 forward and reverse priming sites of the pCR2.1 vector, were used to prepare the sequencing reactions. Sequencing runs were performed by STABVIDA (Lisboa, Portugal).

### Identification of positively selected amino acid sites, their location on the crystal structure, and polymorphism levels

For the six sequences identified as putative *Rosa S-RNases*, we inferred positively selected amino acid sites, using codeML^51^, as implemented in ADOPS^63^, using Muscle as the alignment method. Such analyses where also performed for the 14 *R. chinensis* F-box sequences that are in the vicinity of the *S-RNase*, that cluster with *Prunus SLFL,* and that are expressed in stamen. Since the inclusion of Rchinensis_F-box-3 gene sequence excludes from the analyses a large fraction of the 3´region, we performed these analyses after removing this sequence. codeML analyses were also performed for five *R. multiflora* sc0006888 F-box sequences in the vicinity of the *S-RNase*. The details of the analyses can be seen at the B + database (bpositive.i3s.up.pt^68^; Rosa *S*-locus genes BP2018000004). Model comparisons were M2a-M1a and M8-M7. We consider as positively selected those amino acid sites that show a probability higher than 90% for both naive empirical Bayes (NEB) or Bayes empirical Bayes (BEB) methods.

To visualize these positions in the 3D structure, for the S-RNase (translation of Rchinensis1_3–Rchinensis2_27; Supplementary File S1) we first identified the signal peptide using SignalIP (http://www.cbs.dtu.dk/services/SignalP/) website tool available at ExPASy^60^. After removing the signal peptide, the 3D structure was modeled by I-Tasser^69^, and the model with the highest C-score value used. The same methodology was used for the putative *S*-pollen gene (translation of R chinensis_F box-1; Supplementary File S4), but in this case after removing the F-box domain (the first 60 amino acid positions). All structural images were produced using PyMOL (The PyMOL Molecular Graphics System, Version 1.7.4 Schrödinger, LLC.).

Levels of polymorphism were obtained with DnaSp^70^.

## Supplementary Information

Supplementary Information 1.

Supplementary Information 2.

Supplementary Information 3.

Supplementary Information 4.

## Data Availability

All data generated or analyzed during this study are included in this published article (and its Supplementary Data File S1–S4). *R. arvensis S2*- and *S5-RNase* are deposited in GenBank, acc. Numbers MW452856-MW452861.

## References

[1] De Nettancourt D (1977). Incompatibility in angiosperms.

[2] Igic B, Lande R, Kohn JR (2008). Loss of self-incompatibility and its evolutionary consequences. Int. J. Plant Sci..

[3] Roalson EH, McCubbin AG (2003). S-RNases and sexual incompatibility: Structure, functions, and evolutionary perspectives. Mol. Phylogenet. Evol..

[4] McClure B (2009). Darwin's foundation for investigating self-incompatibility and the progress toward a physiological model for S-RNase-based SI. J. Exp. Bot..

[5] Nowak MD, Davis AP, Anthony F, Yoder AD (2011). Expression and trans-specific polymorphism of self-incompatibility RNases in coffea (Rubiaceae). PLoS ONE.

[6] Entani T (2003). Comparative analysis of the self-incompatibility (S-) locus region of Prunus mume: identification of a pollen-expressed F-box gene with allelic diversity. Genes Cells.

[7] Ushijima K (2003). Structural and transcriptional analysis of the self-incompatibility locus of almond: identification of a pollen-expressed F-box gene with haplotype-specific polymorphism. Plant Cell.

[8] Qiao H (2004). The F-box protein AhSLF-S2 controls the pollen function of S-RNase-based self-incompatibility. Plant Cell.

[9] Tsukamoto T, Ando T, Watanabe H, Marchesi E, Kao TH (2005). Duplication of the S-locus F-box gene is associated with breakdown of pollen function in an S-haplotype identified in a natural population of self-incompatible Petunia axillaris. Plant Mol. Biol..

[10] Cheng J, Han Z, Xu X, Li T (2006). Isolation and identification of the pollen-expressed polymorphic F-box genes linked to the S-locus in apple (Malus × domestica). Sex. Plant Reprod..

[11] Nunes MD, Santos RA, Ferreira SM, Vieira J, Vieira CP (2006). Variability patterns and positively selected sites at the gametophytic self-incompatibility pollen SFB gene in a wild self-incompatible Prunus spinosa (Rosaceae) population. New Phytol..

[12] Hua Z, Meng X, Kao TH (2007). Comparison of Petunia inflata S-Locus F-box protein (Pi SLF) with Pi SLF like proteins reveals its unique function in S-RNase based self-incompatibility. Plant Cell.

[13] Sassa H (2007). S locus F-box brothers: multiple and pollen-specific F-box genes with S haplotype-specific polymorphisms in apple and Japanese pear. Genetics.

[14] Wheeler D, Newbigin E (2007). Expression of 10 S-class SLF-like genes in Nicotiana alata pollen and its implications for understanding the pollen factor of the S locus. Genetics.

[15] Kubo K (2010). Collaborative non-self recognition system in S-RNase-based self-incompatibility. Science.

[16] Minamikawa M (2010). Apple S locus region represents a large cluster of related, polymorphic and pollen-specific F-box genes. Plant Mol. Biol..

[17] Tsukamoto, T., Hauck, N. R., Tao, R., Jiang, N. & Iezzoni, A. F. Molecular and genetic analyses of four nonfunctional S haplotype variants derived from a common ancestral S haplotype identified in sour cherry (Prunus cerasus L.). *Genetics* **184**, 411–427. 10.1534/genetics.109.109728 (2010). 10.1534/genetics.109.109728 PMC2828722 19917768

[18] Kakui H (2011). Sequence divergence and loss-of-function phenotypes of S locus F-box brothers genes are consistent with non-self recognition by multiple pollen determinants in self-incompatibility of Japanese pear (Pyrus pyrifolia). Plant J..

[19] Okada K (2011). Related polymorphic F-box protein genes between haplotypes clustering in the BAC contig sequences around the S-RNase of Japanese pear. J. Exp. Bot..

[20] Aguiar B (2013). Patterns of evolution at the gametophytic self-incompatibility Sorbus aucuparia (Pyrinae) S pollen genes support the non-self recognition by multiple factors model. J. Exp. Bot.

[21] Williams JS, Der JP, dePamphilis CW, Kao TH (2014). Transcriptome analysis reveals the same 17 S-locus F-box genes in two haplotypes of the self-incompatibility locus of Petunia inflata. Plant Cell.

[22] Williams JS (2014). Four previously identified Petunia inflata S-locus F-box genes are involved in pollen specificity in self-incompatibility. Mol. Plant.

[23] Kubo K (2015). Gene duplication and genetic exchange drive the evolution of S-RNase-based self-incompatibility in Petunia. Nat. Plants.

[24] Aguiar B (2015). Convergent evolution at the gametophytic self-incompatibility system in Malus and Prunus. PLoS ONE.

[25] Pratas MI (2018). Inferences on specificity recognition at the Malusxdomestica gametophytic self-incompatibility system. Sci. Rep..

[26] Vieira J (2019). Predicting specificities under the non-self gametophytic self-incompatibility recognition model. Front Plant. Sci..

[27] Ikeda K (2004). Primary structural features of the S haplotype-specific F-box protein, SFB Prunus. Sex. Plant Reprod..

[28] Matsumoto, D. & Tao, R. Distinct Self-recognition in the Prunus S-RNase-based gametophytic self-incompatibility system. *Hortic. J.* **85**. 10.2503/hortj.MI-IR06 (2016).

[29] Tsukamoto, T. *et al.* Genetic and molecular characterization of three novel S-haplotypes in sour cherry (Prunus cerasus L.). *J. Exp. Bot.* **59**, 3169–3185. 10.1093/jxb/ern172 (2008). 10.1093/jxb/ern172 PMC2504349 18617504

[30] Sun P, Li S, Lu D, Williams JS, Kao TH (2015). Pollen S-locus F-box proteins of Petunia involved in S-RNase-based self-incompatibility are themselves subject to ubiquitin-mediated degradation. Plant J..

[31] Li J (2017). Electrostatic potentials of the S-locus F-box proteins contribute to the pollen S specificity in self-incompatibility in Petunia hybrida. Plant J..

[32] Wu L (2018). Use of domain-swapping to identify candidate amino acids involved in differential interactions between two allelic variants of type-1 S-locus F-box protein and S3-RNase in petunia inflata. Plant Cell Physiol..

[33] Igic B, Kohn JR (2001). Evolutionary relationships among self-incompatibility RNases. Proc. Natl. Acad. Sci. USA.

[34] Steinbachs JE, Holsinger KE (2002). S-RNase-mediated gametophytic self-incompatibility is ancestral in eudicots. Mol. Biol. Evol..

[35] Vieira J, Morales-Hojas R, Santos RA, Vieira CP (2007). Different positively selected sites at the gametophytic self-incompatibility pistil S-RNase gene in the Solanaceae and Rosaceae (Prunus, Pyrus, and Malus). J. Mol. Evol..

[36] Vieira J, Fonseca NA, Vieira CP (2008). An S-RNase-based gametophytic self-incompatibility system evolved only once in eudicots. J. Mol. Evol..

[37] Ramanauskas K, Igic B (2017). The evolutionary history of plant T2/S-type ribonucleases. PeerJ.

[38] Tao R, Iezzoni AF (2010). The S-RNase-based gametophytic self-incompatibility system in Prunus exhibits distinct genetic and molecular features. Sci. Hortic..

[39] Akagi T, Henry IM, Morimoto T, Tao R (2016). Insights into the prunus-specific S-RNase-based self-incompatibility system from a genome-wide analysis of the evolutionary radiation of S locus-related F-box genes. Plant Cell Physiol..

[40] Gudin S (2000). Rose: genetics and breeding. Plant Breed. Rev..

[41] Cole P, Melton B (1986). Self-compatibility and cross-compatibility relationships among genotypes and between ploidy of the rose. J. Am. Soc. Hortic. Sci..

[42] Heslop-Harrison Y, Shivanna KR (1977). The receptive surface of the angiosperms stigma. Ann. Bot..

[43] Ueda Y, Akimoto S (2001). Cross- and self-compatibility in various species of the Genus Rosa. J. Hortic. Sci. Biotechnol..

[44] Hibrand Saint-Oyant, L. *et al.* A high-quality genome sequence of Rosa chinensis to elucidate ornamental traits. *Nat. Plants* **4**, 473–484. doi:10.1038/s41477-018-0166-1 (2018). 10.1038/s41477-018-0166-1 PMC6786968 29892093

[45] Debener T (2010). Genetic and molecular analyses of key loci involved in self incompatibility and floral scent in roses. Acta Hort..

[46] Wei Y (2017). Cloning and bioinformatics analysis of Rosa rugose S Locus F-Box Gene (RrSLF). Am. J. Plant Sci..

[47] Yokoya K, Roberts AV, Mottley J, Lewis R, Brandham PE (2000). Nuclear DNA amounts in roses. Ann. Bot..

[48] Bendahmane M, Dubois A, Raymond O, Bris ML (2013). Genetics and genomics of flower initiation and development in roses. J. Exp. Bot..

[49] Raymond O (2018). The Rosa genome provides new insights into the domestication of modern roses. Nat. Genet..

[50] Nakamura N (2018). Genome structure of Rosa multiflora, a wild ancestor of cultivated roses. DNA Res..

[51] Yang Z (2007). PAML 4: phylogenetic analysis by maximum likelihood. Mol. Biol. Evol..

[52] Vieira J, Ferreira PG, Aguiar B, Fonseca NA, Vieira CP (2010). Evolutionary patterns at the RNase based gametophytic self-incompatibility system in two divergent Rosaceae groups (Maloideae and Prunus). BMC Evol. Biol..

[53] Heitzler P (2019). Rosa arvensis as a possible genetic model. Acta Hortic..

[54] Xiang Y (2017). Evolution of Rosaceae fruit types based on nuclear phylogeny in the context of geological times and genome duplication. Mol. Biol. Evol..

[55] Smulders MJM (2019). In the name of the rose: A roadmap for rose research in the genome era. Hortic. Res..

[56] Zhu ZM, Gao XF, Fougere-Danezan M (2015). Phylogeny of Rosa sections Chinenses and Synstylae (Rosaceae) based on chloroplast and nuclear markers. Mol. Phylogenet. Evol..

[57] De Cock, K. Genetic diversity of wild roses (Rosa spp.) in Europe, with an in-depth morphological study of Flemish populations. *Ghent University. Faculty of Bioscience Engineering, Ghent, Belgium.* (2008).

[58] López-Fernández H (2019). Bioinformatics protocols for quickly obtaining large-scale data sets for phylogenetic inferences. Interdiscip. Sci. Comput. Life Sci..

[59] López-Fernández H (2020). SEDA: a desktop tool suite for FASTA files processing. IEEE/ACM Trans. Comput. Biol. Bioinform..

[60] Artimo P (2012). ExPASy: SIB bioinformatics resource portal. Nucleic Acids Res..

[61] 61Martin, M. CUTADAPT removes adapter sequences from high-throughput sequencing reads. *EMBnet.journal* **17**, doi:10.14806/ej.17.1.200 (2011).

[62] Jackman, S. D. *et al.* ABySS 2.0: resource-efficient assembly of large genomes using a Bloom filter. *Genome Res.* **27**, 768–777. 10.1101/gr.214346.116 (2017). 10.1101/gr.214346.116 PMC5411771 28232478

[63] Reboiro-Jato D (2012). ADOPS–Automatic Detection Of Positively Selected Sites. J Integr Bioinform.

[64] Huelsenbeck JP, Ronquist F (2001). MRBAYES: Bayesian inference of phylogenetic trees. Bioinformatics.

[65] Kumar, S., Stecher, G. & Tamura, K. MEGA7: Molecular evolutionary genetics analysis version 7.0 for bigger datasets. *Mol. Biol. Evol.* **33**, 1870–1874. 10.1093/molbev/msw054 (2016). 10.1093/molbev/msw054 PMC8210823 27004904

[66] Haas BJ (2013). De novo transcript sequence reconstruction from RNA-seq using the Trinity platform for reference generation and analysis. Nat. Protoc..

[67] Ingram GC (1997). Dual role for fimbriata in regulating floral homeotic genes and cell division in Antirrhinum. EMBO J..

[68] Vazquez N (2018). Large scale analyses and visualization of adaptive amino acid changes projects. Interdiscip. Sci..

[69] Yang J (2015). The I-TASSER Suite: protein structure and function prediction. Nat. Methods.

[70] Rozas J, Sanchez-DelBarrio JC, Messeguer X, Rozas R (2003). DnaSP, DNA polymorphism analyses by the coalescent and other methods. Bioinformatics.

