# Supplementary figures and images for: The identification of the Rosa S-locus and implications on the evolution of the Rosaceae gametophytic self-incompatibility systems
Source: Sci Rep. 2021 Feb 12;11:3710. doi: 10.1038/s41598-021-83243-8 (PMC7881130; doi:10.1038/s41598-021-83243-8)

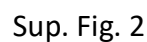

Supplement: Supplementary file 1 — Supplementary Information 1. [file 41598_2021_83243_MOESM1_ESM.pdf]
