# Supplementary material for: The identification of the Rosa S-locus and implications on the evolution of the Rosaceae gametophytic self-incompatibility systems
Source: Sci Rep. 2021 Feb 12;11:3710. doi: 10.1038/s41598-021-83243-8 (PMC7881130; doi:10.1038/s41598-021-83243-8)
Supplement: Supplementary file 2 — Supplementary Information 2. [file 41598_2021_83243_MOESM2_ESM.pdf]

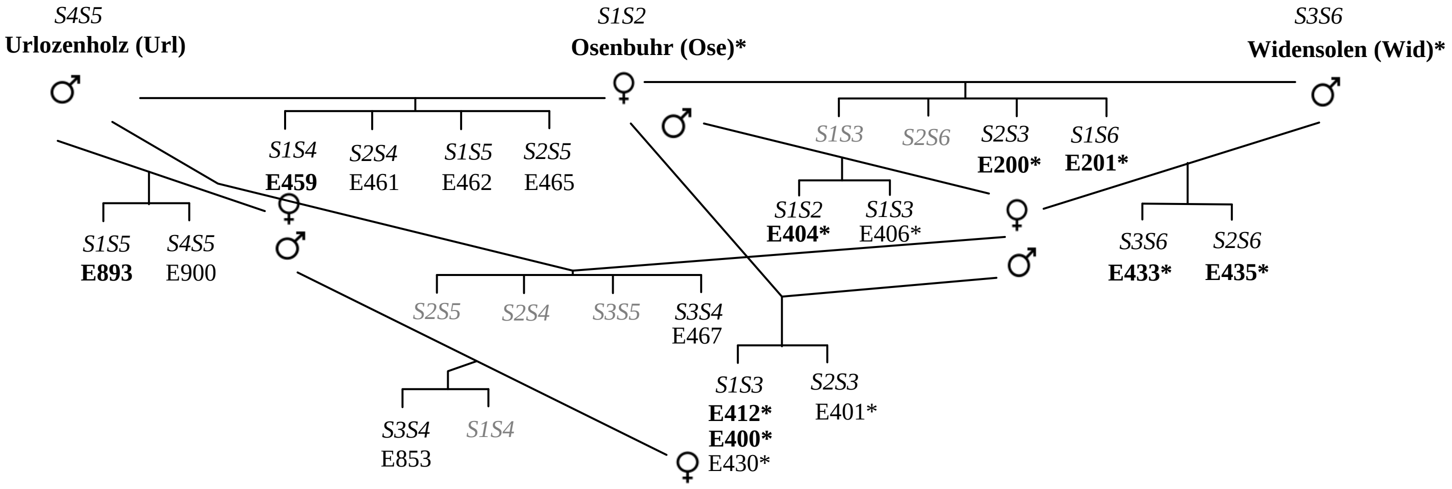

A

S-haplotypes	Accessions (origin, date)	Functional tests
S1 S2	Ose (wild collected, 1997)	founder for S1; no hips with E404; hips with Wid, Url, E200, E201, E400, E401, E406, E412, E430, E459
	E404 (E200 x Ose, 2007)	no hips with Ose; hips with E200, E201
S1 S3	E400 (Ose x E200, 2007)	no hips with E406, E412, E430; hips with Ose, E200, E201
	E406 (E200 x Ose, 2007)	no hips with E400, E412, E430; hips with Ose, E200, E201
	E412 (Ose x E200, 2007)	no hips with E400, E406, E430; hips with Ose, Wid, E200, E201
	E430 (Ose x E200, 2007)	no hips with E400, E406, E412; hips with Ose, E200, E459
S1 S4	E459 (Ose x Url, 2012)	founder for S4; hips with Ose, Wid, Url, E200, E201, E401, E430
S1 S5	E462 (Ose x Url, 2012)	hips with E459
	E893 (E459 x Url, 2017)	not yet mature
S1 S6	E201 (Ose x Wid, 2003)	hips with Ose, Wid, E200, E400, E404, E406, E412, E430, E459
S2 S3	E200 (Ose x Wid, 2003)	founder for S2 and for S3; no hips with E401; hips with Ose, Wid, Url, E201, E400, E404, E406, E412, E430, E459
	E401 (Ose x E200, 2007)	no hips with E200; hips with Ose, Wid, Url, E201, E459
S2 S4	E461 (Ose x Url, 2012)	hips with E459
S2 S5	E465 (Ose x Url, 2013)	hips with E459
S2 S6	E435 (E200 X Wid, 2008)	hips with Wid, E433
S3 S4	E853 (E430 x E459, 2016)	not yet mature
	E467 (E200 x Url, 2013)	not yet mature
S3 S6	Wid (wild collected, 1999)	founder for S6; no hips with E433; hips with Ose, Url, E200, E201, E412, E459
	E433 (E200 X Wid, 2008)	no hips with Wid; hips with E435
S4 S5	Url (wild collected, 2000)	founder for S5; hips with Ose, Wid, E200, E459
	E900 (E459 x Url, 2018)	not yet mature

B
