# Supplementary material for: The identification of the Rosa S-locus and implications on the evolution of the Rosaceae gametophytic self-incompatibility systems
Source: Sci Rep. 2021 Feb 12;11:3710. doi: 10.1038/s41598-021-83243-8 (PMC7881130; doi:10.1038/s41598-021-83243-8)
Supplement: Supplementary file 3 — Supplementary Information 3. [file 41598_2021_83243_MOESM3_ESM.pdf]

The identification of the *Rosa S*-locus and implications on the evolution of the Rosaceae gametophytic self-incompatibility systems

Vieira J[1,2], Pimenta J[1,2], Gomes A[1,2], Laia J[1,2], Rocha S[1,2], Heitzler P[3], and Vieira CP[1,2*]

Supplementary Information

Supplementary Table 1. Breeding system, genome accession number, quality of the assembly, and ploidy of the *Rosa* species used in this work.

Supplementary Table 2. Size, motif 1and 2, and IP of the sequences identified in *R. chinensis, R. multiflora, R. moschata, R. leavigata, R. rugosa, R. persica, R. xantina, R. minutifolia, R. odorata, R. arvensis,* and *R. majalis* genomes.

Supplementary Table 3. Location of the *SFB/SLFL/SFBB* like sequences of *R. chinensis* chromosome 3, *R. multiflora* sc0006888, and *R. multiflora* sc0001861.

Supplementary Table 4. *R. chinensis* transcriptome data used in this study.

Supplementary Figure 1. Neighbor-joining phylogenetic tree, showing the relationship of the *Rosa* sequences covering at least the exon where motif 2 is located, with *Fragaria, Prunus*, and *Malus S-RNase* lineage genes. Since not all sequences overlap, two different phylogenies were produced (A and B) using different sequence subsets aligned using the ClustalW algorithm. For phylogenetic inferences, the neighbor-joining method and pairwise deletion option was used, as implemented in Mega7[64]. The tree was rooted with *MDP0000267606A T2- RNase,* not involved in GSI [36]. Numbers below the branches represent bootstrap support values above 70.

Supplementary Figure 2. Neighbor-joining phylogenetic tree, showing the relationship of the *Rosa* sequences covering the region of motif 1 with those sequences identified as *Rosa S-RNases* (see Figure 1, and Supplementary Fig. 1). The tree was rooted with *MDP0000267606A T2- RNase,* not involved in GSI [36]. Numbers below the branches represent bootstrap support values above 70.

Supplementary Figure 3. *R. arvensis S*-haplotype inferences, according to the pedigree (A) and hand-pollination tests (B). In bold are the individuals used in this study, in grey are those *S*-haplotypes that are expected but not observed. Stars indicate those S-haplotypes that have been confirmed with hand-pollination tests.

Supplementary File 1. The *R. chinensis* and *R. multiflora S-RNase* lineage sequences together with *Fragaria, Prunus*, and *Malus S-RNase* lineage genes**.**

Supplementary File 2. The *Rosa* sequences covering at least the exon where motif 2 is located and those identified as *Rosa S-RNase, Fragaria, Prunus*, and *Malus S-RNase* lineage genes**.**

Supplementary File 3. The *Rosa* sequences covering region of motif 1 and those identified as *Rosa S-RNase* (see Figure 1, and Supplementary Fig. 1).

Supplementary File 4. The *R. chinensis* and *R. multiflora SFB/SLFL/SFBB* like sequences together with *Prunus SFB* and *SLFL* genes, *M. domestica S1-SFBB*s, *Petunia* and *Nicotiana SLF*, and *A. thaliana* F-box/kelch-repeat sequences.

Supplementary Table 1. Breeding system, genome accession number, quality of the assembly, and ploidy of the *Rosa* species used in this work.

Rosa species	Accession number	N50 of the assembly	Ploidy	Breeding system
R. chinensis	1- GCA_002994745.2 (assigned as Rchinensis1)<br>2- after Get ORF(assigned as Rchinensis2)<br>3- https://iris.angers.inra.fr/obh/ (assigned as Rchinensis3)<br>4- after Get ORF(assigned as Rchinensis4)	69643165<br>16984	2x	SI [4]
R. multiflora	GCA_002564525.1 (assigned as Rmultiflora)	90830	2x; 3x	SI [4]
R. moschata	Abyss assembly based on: SRR7077017 (assigned as Rmoschata17)<br>SRR6175508 (assigned as Rmoschata08)	769<br>1652	2x	SI [4]
R. laevigata	Abyss assembly based on SRR7077018 (assigned as Rlaevigata)	1905	2x	SI [4]
R. rugosa	Abyss assembly based on: SRR6175514 (assigned as Rrugosa14)<br>SRR7077019 (assigned as Rrugosa19)	1759<br>1031	2x	Partially SI [4]
R. persica	Abyss assembly based on SRR7077021 (assigned as Rpersica)	2403	2x	SI [77]
R. xanthina	Abyss assembly based on SRR7077022 (assigned as Rxanthina)	1848	2x	SI [2]
R. minutifolia	Abyss assembly based on SRR7077023 (assigned as Rminutifolia)	1685	2x	SI [77]
R. odorata	Abyss assembly based on SRR6175507 (assigned as Rodorata)	1259	2x	SI [4]
R. odorata var. gigantea	Abyss assembly based on SRR6175516 (assigned as Rodorata_gigantea)	801	2x	SI [4]
R. arvenses	Abyss assembly based on SRR6175512 (assigned as Rarvensis)	2946	2x	SI [4]
R. majalis	Abyss assembly based on SRR6175513 (assigned as Rmajalis)	1538	2x	SI [78]

SI-self-incompatible

Supplementary Table 2. Size, motif 1and 2, and IP of the sequences identified in *R. chinensis*, *R. multiflora*, *R. moschata*, *R. leavigata*, *R. rugosa*, *R. persica*, *R. xantina*, *R. minutifolia*, *R. odorata*, *R. arvensis,* and *R. majalis* genomes.

**Sequence Name**	**Size of the putative coding region (bp)**	**Motif 1**	**Motif 2**	**IP**
Rchinensis1_1 (NC 037094; XP_024173652; Chr 7)	339	FTIGGIWPYT	WPAITIL	9.54
Rchinensis1_2 (NC_037091; XP_024195082: Chr 4)	696	FGIHGLWPNY	WPTLACP	4.74
Rchinensis1_3 (NC_037090; XP_024190016; Chr 3)	687	YTIHGLWPSN	WPNLERP	8.81
Rchinensis1_4 (NC_037089: XP_024178267; Chr 2)	699	FTVHGIWPTN	WPSVVTN	8.95
Rchinensis1_5 (NC_037091; XP_024196661; Chr 4)	684	FGIHGLWPNY	WPSMSCP	5.54
Rchinensis1_6 (NC 037093; XP_024164552; Chr 6)	693	FTIHGLWPNN	WPDVKNA	8.8
Rchinensis1_7 (NC_037091; XP_024195588; Chr 4)	801	FTLHGLWDQN	WPDLSAD	5.76
Rchinensis1_8 (NC_037090; XP_024190063; Chr 3)	663	FTIHGVWPAN	WPNVERA	8.34
Rchinensis1_9 (NC_037093; XP_024163375; Chr 6)	867	FTIHGLWPDY	WPSLSCG	6.08
Rchinensis1_10 (NC_037091; XP_024196470; Chr 4)	348	n. a.	n. a.	6.24
Rchinensis1_11 (NC_037089; XP_024178028; Chr 2)	711	n. a	n. a.	8.87
Rchinensis1_12 (NC_037089; XP_024177989; Chr 2)	468	FHVHGIWPSN	WPSVLTT	6.12
Rchinensis2_1 (NC_ 0370931; Chr 6)		Identical to Rchinensis1_6		
Rchinensis2_3 (NC_ 0370911; Chr 4)	804	FTLHGLWDQN	WPDLSAD	5.79
Rchinensis2_4 (NC_ 0370941; Chr 7)	423	FTLHGMWASN	WPTLLDN	5.66
Rchinensis2_5 (NC_ 0370901; Chr 3)	420	FMAFGQYELD	WPQLIAS	9.41

Rchinensis2_6 (NC_ 0370891; Chr 2)#	459	FTLHGLWPQA	WPNLKHT	9.34
Rchinensis2_7 (NC_ 0370881; Chr 1)	579	FTLHGLWPFN	WKSYMRS	8.54
Rchinensis2_8 (NC_ 0370901; Chr 3)	564	WGIHGIWAEK	WSSLSAT	8.96
Rchinensis2_9 (NC_ 0370901; Chr 3)	633	WTIHGLWTCE	WLSNNKN	6.81
Rchinensis2_11 (NC_ 0370911; Chr 4)	567	FTVHGIWPTN	WPSVRRD	9.42
Rchinensis2_12 (NC_ 0370941; Chr 7)		Identical to Rchinesis1_1		
Rchinensis2_15 (NC_ 0370891; Chr 2)		Identical to Rchinesis1_4		
Rchinensis2_16 (NC_ 0370901; Chr 3)	591	FTLHGFWPSN	WPNVKYP	8.78
Rchinensis2_18 (NC_ 0370901; Chr 3)	567	WTIHGLWAER	WPSIMGK	7.60
Rchinensis2_20 (NC_ 0370891; Chr 2) #	330	FTLHGLWPQA	WPDLTHS	6.31
Rchinensis2_23 (NC_ 0370891; Chr 2)		Identical to Rchinesis1_11		
Rchinensis2_24 (NC_ 0370891; Chr 2)	708	FHVHGIWPSN	WPSVLTQ	8.98
Rchinensis2_25 (NC_ 0370901; Chr 3)		Identical to Rchinesis1_ 8		
Rchinensis2_26 (NC_ 0370891; Chr 2)	495	FHVHGIWPSN	WPSVLTT	6.12
Rchinensis2_27 (NC_ 0370901; ch3)		Identical to Rchinesis1_3		
Rchinensis2_28 (NC_ 0370891; Chr 2)	588	NQVSCTWNMA	WPSVLIT	9.07
Rchinensis2_29 (NC_ 0370901; Chr 3)	747	FTIHGLWPSN	WPDVKRT	9.83
Rchinensis2 30 (NC_ 0370931; Chr 6)	459	ITVHGLWPSN	WSDVEKG	7.88
Rchinensis2_31 (NC_ 0370901; Chr 3)	651	FSIHGMWPSN	WPSYTGL	9.13

Rchinensis2_32_1 (NC_ 0370911; Chr 4)	588	FGIHGLWPNY	WPTLACP	4.73
Rchinensis2_32_2 (NC_ 0370911; Chr 4)	513	FGIHGLWPNY	WPSMSCP	8.09
Rchinensis2_32_3 (NC_ 0370911; Chr 4)	693	FGIHGLWPNY	WPTLACP	4.79
Rchinensis3_1 (RC6 G0321700)	861	FTIHGLWPDY	WPSLSCG	6.08
Rchinensis3_2 (RC4 G0029500)	834	FTLHGLWDQN F	WPDLSADR	5.99
Rchinensis3_3 (RC6 G0432600)	714	FTIHGLWPNN	WPDVKNA	8.62
Rchinensis3_4 (RC2 G0245900)		Identical to Rchinensis2_24		
Rchinensis3_5 (RC2 G0245600)		Identical to Rchinesis1_11		
Rchinensis3_6 (*SRNase30*; RC3 G033300)	705	FFIHGLWPSN	WPSFTRR	7.01
Rchinensis3_7 (RC4 G0432700)	699	FGIHGLWPNY	WPTLACP	4.79
Rchinensis3_8 (RC4 G0432600)	684	FTIHGLWPNY	WPSMSCP	5.54
Rchinensis3_9 (*SRNase26*; RC3 G0332600)	681	FTIHGLWPSN	WRSYLIA	7.54
Rchinensis3_10 (*SRNase36*; RC3 G0333600)	660	FTIHGLWPSN	WPDVIHG	7.57
Rchinensis3_11 (RC1 G0140200)		Identical to Rchinensis2_7		
Rchinensis3_12 (RC3 G0162200)	570	WTVHGLWAER	WPSIMGK	6.87
Rchinensis3_13 (RC4 G009400)0		Identical to Rchinensis2_11		
Rchinensis3_14 (RC2 G0233900)	486	FHVHGIWPSN	WPSVLTT	6.12
Rchinensis3_15 (RC2 G0103900)		Identical to Rchinensis2_6		
Rchinensis3_16 (RC4	453	FIIGGFRPG	WPARAAT	9.39

G0432900)				
Rchinensis3_17 (RC3 G0038500)		Identical to Rchinensis2_16		
Rchinensis3_18 (RC7 G0339700)	345	n. a,	n. a.	9.06
Rchinensis4_1 (Chr 6)		Identical to Rchinensis3_3		
Rchinensis4_3 (Chr 7)	405	FTLHGMWASN	WPTLLDN	5.22
Rchinensis4_4 (Chr 1)		Identical to Rchinensis2_7		
Rchinensis4_5_1 (Chr 4)	771	FTLHGLWDQN	WPDLSAD	5.56
Rchinensis4_5_2 (Chr 4)	807	FTLHGLWDQN	WPDLSAD	5.76
Rchinensis4_7 (Chr 2)#		Identical to Rchinensis2_6		
Rchinensis4_8 (Chr 0)	648	FTIHGLWPNN	WPDLRNG	8.42
Rchinensis4_9 (Chr 0)	834	FTLHGLWDQN	WPDLSAD	5.99
Rchinensis4_11 (Chr 3)	636	WTIHGLWTCE	WPSNNKN	6.81
Rchinensis4_12 (Chr 3)		Identical to Rchinensis2_8		
Rchinensis4 28 (*SRNase26*; Chr 3)		Identical to Rchinensis3_9		
Rchinensis4_29 (Chr 3)	570	FTLHGLWPQA	WPDLTHS	6.87
Rchinensis4_31 (Chr 2)	690	FHVHGIWPSN	WPSVLTQ	8.87
Rchinensis4_33 (Chr 2		Identical to Rchinensis2_24		
Rchinensis4_35 (Chr 2)	486	FHVHGIWPSN	WPSVLTT	6.12
Rchinensis4_37 (Chr 2)	336	FTLHGLWPQA	WPDLTHS	6.32
Rchinensis4_38 (Chr 4)		Identical to Rchinensis2_11		
Rchinensis4_40 (Chr 3)		Identical to Rchinensis2_16		
Rchinensis4_41 (Chr 2)	711	FTVHGIWPTN	WPSVVTN	8.59
Rchinensis4_42 (Chr 4)	684	FGIHGLWPNY	WPSMSCP	5.54
Rchinensis4_44 _1 (*SRNase36*; Chr 3)		Identical to Rchinensis3_10		
Rchinensis4_44 _2 (*SRNase30*; Chr 3)		Identical to Rchinensis3_6		
Rmultiflora_1	606	FTIHGLWPNN	WPDVKNA	8.38
Rmultiflora_2	408	n. a.	WPDVKNA	8.91

Rmultiflora_3	628	FSIHGMWPSN	WPSYTGL	9.25
Rmultiflora_4	594	YTIHGLWPNN	WPNLENS	8.38
Rmultiflora_5	246	FTLHGFWPSN	n. a.	
Rmultiflora_6	639	FTVHGIWPTN	WPSVVTN	8.31
Rmultiflora_7	483	n. a.	WPNVKYP	9.26
Rmultiflora_8	657	YTIHGLWPSN	WPNLENL	8.37
Rmultiflora_9	597	FHVHGIWPSN	WPSVLTT	8.53
Rmultiflora_10	672	FTIHGLWPSN	WRSYLIA	6.26
Rmultiflora_11	708	FTIHGVWPAN	WPNVERA	8.95
Rmultiflora_12	426	FTVHGIWPTN	WPSFRRD	6.56
Rmultiflora_14	276	n. a.	WPNVKYP	7.89
Rmultiflora_15	672	FHVHGIWPSN	WPSVLTQ	8.33
Rmultiflora_16	675	FTIHGLWPSN	WPQLIAR	9.12
Rmultiflora_18	150	FTIHGLWPSN	n. a.	
Rmultiflora_19	591	FGIHGLWPNY	WPTLACP	4.71
Rmultiflora_20	591	FGIHGLWPNY	WPTLACP	4.73
Rmultiflora_21	711	FTIHGLWPSN	WPDVKPT	9.42
Rmultiflora_24	459	FTLHGLWPQA	WPNLKHT	9.22
Rmultiflora_25	687	FTIHGLWPSN	WPNVYYY	8.68
Rmultiflora_27	624	WTIHGLWAER	WPSIMGK	7.64
Rmultiflora_28	615	WTIHGLWAER	WPSIMGK	7.64
Rmultiflora_29	402	n. a.	WPSIMGK	8.31
Rmultiflora_31	291	FTIGGIWPYT	n. a.	-
Rmoschata08_1	147	n. a.	n. a.	6.75
Rmoschata08_2#	270	FTLHRLWHQN	WPDLSRD	9.69
Rmoschata08_3	219	n. a.	n. a.	8.66
Rmoschata08_5	408	n. a.	WPNPFWN	5.4
Rmoschata08_6	408	n. a.	WPSFSTT	5.33
Rmoschata08_7	135	FTVHGIWPTN	n. a.	
Rmoschata08_9	147	n. a.	WPSLYGK	5.41
Rmoschata08_10	417	n. a.	WPNVKYP	8.62

Rmoschata08_11	519	FTIHGVWPAN	WPNVERA	9.21
Rmoschata08_12	261	YTIHGLWPSN	n. a.	
Rmoschata08_13	423	n. a.	WPTLACP	4.75
Rmoschata08_14	168	FGIHGLWPNY	n. a.	
Rmoschata08_15	126	FTIHGLWPSN	n. a.	
Rmoschata08_17#	525	FTLHGLWDQN	WPDLSAD	8.97
Rmoschata08_18	714	FTIHGLWPNN	WPDVKNA	8.28
Rmoschata08_20	135	n. a.	WPNVEVP	4.7
Rmoschata08_23	447	n. a.	WPSYTGL	9.15
Rmoschata08_24	321	n. a.	WPSLACP	9.01
Rmoschata08_25	438	ITVHGLWPSN	WSDVEKG	9.04
Rmoschata08_26	291	n. a.	n. a.	9.3
Rmoschata08_27	261	n. a.	WPSTYKE	5.57
Rmoschata08_28	147	n. a.	WPSIMGK	6.92
Rmoschata08_29	171	n. a.	n. a.	8.02
Rmoschata08_30	108	FTIHG	n. a.	
Rmoschata08_31	471	FGIHGLWPNY	WPSMSCP	6.56
Rmoschata08_32	213	FTVHGIWPTN	WPSVLTN	5.67
Rmoschata08_33#	591	FTLHGLWPQA	WPDLTHS	5.95
Rmoschata08_34	186	FTIHGLFFFW	n. a.	
Rmoschata08_36	408	n. a.	n. a.	9.36
Rmoschata08_37	237	FSVHGLWPSN	n. a.	
Rmoschata08_39	180	FTIHGFWPSN	n. a.	
Rmoschata08_40	162	n. a.	WPQLMSH	5.78
Rmoschata08_41	669	WTIHGLWTCE	WPSNNKN	8.09
Rmoschata17_2	141	FTIHGLWPNN	n. a.	
Rmoschata17_3	315	n. a.	n. a.	4.64
Rmoschata17_4	408	LTIHGLWPTN	WPNAYGP	8.89
Rmoschata17_5	147	n. a.	WPNLTGD	4.32
Rmoschata17_6	270	n. a.	WPSLSCA	9.51
Rmoschata17_9	705	FHVHGIWPSN	WPSVLTQ	6.03
Rmoschata17_10		Identical to Rmoschata08_31		

Rmoschata17_12	288	n. a.	n. a.	9.54
Rmoschata17_14	255	YTIHGLWPSN	WPNLELP	9.44
Rmoschata17_15	150	FTIHGLWPSN	n. a.	
Rmoschata17_18	120	FTLHGLWASN	n. a.	
Rmoschata17_23		Identical to Rmoschata08_36		
Rmoschata17_24	228	n. a.	n. a.	7.86
Rmoschata17_27	444	n. a.	WPDLKNG	8.28
Rmoschata17_28	240	n. a.	n. a.	4.97
Rmoschata17_29	114	n. a.	WPQLLAH	4.22
Rmoschata17_30	198	FSIHGMWPSN	n. a.	
Rmoschata17_31	336	FTIHGLWPSN	WRSYPIG	7.00
Rmoschata17_32	219	n. a.	n. a.	8.55
Rmoschata17_36		Identical to Rmoschata08_14		
Rlaevigata_1	228	n. a.	n. a.	6.74
Rlaevigata_2	108	n. a.	n. a.	7.62
Rlaevigata_3	219	FTIHGLWPSN	n. a.	
Rlaevigata_4	444	n. a.	WPDLKNG	8.87
Rlaevigata_6	450	n. a.	WRSYLIG	6.94
Rlaevigata_7	255	n. a.	WPSMSCP	9.15
Rlaevigata_8	240	FTIHGLWPNN	n. a.	
Rlaevigata_9	162	FTIHGLWPSN	n. a.	
Rlaevigata_10	438	n. a.	WPNVEKV	8.25
Rlaevigata_11	210	FGIHGLWPNY	n. a.	
Rlaevigata_12	750	FTIHGLRPSN	WPDVERL	9.12
Rlaevigata_13	510	WGIHGIWAEK	WPSISTQ	4.97
Rlaevigata_16#	462	FTLHGLWPQA	WPNLKHT	9.32
Rlaevigata_17	492	FTLHGMWASN	WPTLLDN	4.77
Rlaevigata_18	462	FTIHGVWPAN	WPNVERA	9.20
Rlaevigata_19	438	FHVHRIWPSN	WSSVLTA	9.14
Rlaevigata_20	420	n. a.	WPSLYAN	5.17
Rlaevigata_21	495	FSIHGMWPSN	WPSYTGL	9.44

Rlaevigata_24	435	ITVHGLWPSN	WSDVEKG	8.71
Rlaevigata_25#	573	FTLHGLWDQN	WPDLSAD	7.72
Rlaevigata_26	561	FTIHGLWPSN	WPQLIAH	5.46
Rlaevigata_27	711	FHVHGIWPSN	WPSVLTQ	8.38
Rlaevigata_28	420	n. a.	WPQLIPY	8.45
Rlaevigata_30	546	FTVHGIWPTN	WPSVLTD	8.71
Rlaevigata_31	234	FTIHGLWPSN	n. a.	
Rlaevigata_32#	390	FTLHGLWPQA	WPDLTHS	7.40
Rlaevigata_33	432	FTIGGIWPYT	WPSLPCP	9.49
Rrugosa14_1	177	FSVHGLWPSN	n. a.	
Rrugosa14_2	171	FTIHGLWPSN	n. a.	
Rrugosa14_3	132	FTVHGIWPTN	n. a.	
Rrugosa14_4	81	n. a.	n. a.	8.15
Rrugosa14_5	420	n. a.	WPTLACP	4.75
Rrugosa14_6	426	n. a.	WPSVLTQ	5.90
Rrugosa14_7	121	FTLHGLWATN	n. a.	
Rrugosa14_8	453	FGIHGLWPNY	WPSMSCP	7.09
Rrugosa14_9	483	FSIHGMWPSN	WPSYTGL	8.98
Rrugosa14_10	114	n. a.	WPQLIAR	8.16
Rrugosa14_11	126	FTIHGLWPSN	n. a.	
Rrugosa14_12	105	FTIHGLWPSN	n. a.	
Rrugosa14_13	624	FTIHGLWPNN	WPDVKNA	8.58
Rrugosa14_15	228	FTVHGIWPTN	WPSVRTD	6.44
Rrugosa14_17	525	LLLHGLWPAN	WPNLTGD	6.05
Rrugosa14_18	392	ITVHGLWPSN	WSDVEKG	8.15
Rrugosa14_19	411	ITVHGLWPSN	WSDVEKG	8.79
Rrugosa14_20	423	n. a.	WPTLACP	4.75
Rrugosa14_23	156	FTIHGLWPSN	n. a.	
Rrugosa14_24	273	FGIHGLWPNY	n. a.	
Rrugosa14_25	135	FTVHGIWPTN	n. a.	8.76
Rrugosa14_26	180	n. a.	WPQLITR	9.30

Rrugosa14_27	435	n. a	WHGLYGK	4.76
Rrugosa14_28	399	FTIHGLWPSN	WPDLENG	7.87
Rrugosa14_29	255	FTIHGLWPSN	n. a.	
Rrugosa14_30	249	n. a.	WPNTYKD	5.16
Rrugosa14_32	423	n. a.	WPDVGGG	8.87
Rrugosa14_33	408	n. a.	WPNVVTP	9.24
Rrugosa14_34	450	n. a.	WRSYPIG	7.66
Rrugosa14_35	516	FTIHGLWPSN	WPNVETP	9.28
Rrugosa14_36	255	n. a.	WPSMSCP	8.91
Rrugosa14_37	108	n. a.	WPSVMTD	4.80
Rrugosa14_38	156	FTIHGLWPSN	n. a.	
Rrugosa14_39#	285	FTLHGLWDQN	WPDLSAD	8.00
Rrugosa14_40	486	FTLHGLWPQA	WPDLTHS	7.48
Rrugosa14_42	627	FTIHGVWPAN	WPNVERA	9.21
Rrugosa19_6	165	n. a.	WPSIMGK	6.92
Rrugosa19_7	183	FTIHGLWPSN	n. a.	
Rrugosa19_8	411	n. a.	WPSNDNE	5.21
Rrugosa19_9		Identical to Rrugosa14_20		
Rrugosa19_10	216	FTLHGFWPSN	n. a.	
Rrugosa19_11	255	FGIHGLWPNY	n. a.	
Rrugosa19_12	201	n. a.	WPQLISH	6.00
Rrugosa19_13	270	n. a.	n. a. *	6.89
Rrugosa19_14	420	n. a.	WPNVKYP	8.85
Rrugosa19_15	342	FHVHGIWPSN	MASVLTT	8.32
Rrugosa19_16	126	n. a.	WPSVLID	4.86
Rrugosa19_17	189	n. a.	WPGISGK	4.60
Rrugosa19_19	186	n. a.	WPDLRNG	6.06
Rrugosa19_21	159	FTLHGLWASN	n. a.	4.20
Rrugosa19_22	258	n. a.	n. a.*	9.81
Rrugosa19_23	270	n. a.	n. a.*	6.89
Rrugosa19_25		Identical to Rrugosa14_25		

Rrugosa19_26		Identical to Rrugosa14_26		
Rrugosa19_27	660	WSIHGIWAEK	WPSLSTD	5.52
Rrugosa19_29		Identical to Rrugosa14_29		
Rrugosa19_31#	471	FTLHGLWPQA	WPNLKHT	9.32
Rrugosa19_33		Identical to Rrugosa14_42		
Rrugosa19_34		Identical to Rrugosa14_38		
Rrugosa19_35		Identical to Rrugosa14_36		
Rrugosa19_36		Identical to Rrugosa14_35		
Rrugosa19_37	141	FTIHG	n. a.	
Rrugosa19_45	249	FTIHGLWPNN	n. a.	
Rrugosa19_47	147	FTIHGLWPNN	n. a.	
Rrugosa19_48	627	WTIHGLWTYE	WPINNKN	9.24
Rrugosa19_50	546	n. a.	WPDVLNG	9.65
Rpersica_2	135	n. a.	n. a.	8.63
Rpersica_4	222	n. a,	n. a.	7.93
Rpersica_5	699	FTIGGIWPYT	WPSLSCP	9.52
Rpersica_6	666	WTIHGLWPVR	WPSLSGK	6.57
Rpersica_7	708	FTLHGLWPFN	WKSYKRS	5.02
Rpersica_8	372	FTVHGIWPTN	WPSVRTD	9.05
Rpersica_9	471	FGIHGLWPNY	WPSMSCP	5.94
Rpersica_10	675	FGIHGLWPNY	WPTLACP	4.55
Rpersica_11	420	FTIHGLWPSN	WPDVRKA	9.18
Rpersica_12	522	FTIHGLWPSN	WPQLIAR	9.38
Rpersica_13	543	FILHGLWPVN	WKNCEKG	4.64
Rpersica_14	651	FSIHGLWPSN	WPDVEKG	8.85
Rpersica_15	177	n. a.	n. a.	9.78
Rpersica_16	174	NTIHAHWPTN	n. a.	10.57
Rpersica_17#	462	FTLHGLWLQA	WPNLKHT	9.30
Rpersica_18	207	FTLHGLWAIN	n. a.	
Rpersica_19	189	n. a.	n. a.	9.72
Rpersica_20	585	FTIHGVWPTN	WPNVERA	9.00

Rpersica_21	315	FHVHGIWPSN	WPSVLTT	6.17
Rpersica_22	156	n. a.	WPNLIGN	9.78
Rpersica_23	552	VTVHGLWPSN	WPDVETG	8.81
Rpersica_24	627	FTLYGFWPVN	WKNYEKG	5.82
Rpersica_25	603	FSIHGLWPSN	WPDVEKG	8.88
Rpersica_26	180	n. a.	WPQLIAH	5.38
Rxanthina_ 1	687	FTLHGLWPFN	WKSYERS	4.95
Rxanthina_ 2#	390	FTLHGLWPQA	WPNLKHA	9.16
Rxanthina_ 3	228	n. a.	n. a.	5.31
Rxanthina_ 4	207	n. a.	n. a.	9.05
Rxanthina_ 6	441	n. a.	WPDLRNG	9.18
Rxanthina_ 7	780	LTIHGLWPGF	WPNVKSP	8.75
Rxanthina_ 8	792	FTIHGLWPSN	WPDVVYL	9.43
Rxanthina_ 9	669	LTIHGLWPSN	APVNCSF	8.6
Rxanthina_ 10	168	FGIHGLWPNY	n. a.	
Rxanthina_ 11	756	FTLHGMWASN	WPTLLDN	7.68
Rxanthina_ 12	654	FSIHGVWPEN	WPNVVIP	4.44
Rxanthina_ 13	552	ITVHGLWPSN	WSDVEKG	9.23
Rxanthina_ 14	153	n. a.	WPSIMGK	6.92
Rxanthina_ 15	255	FTIHGLWPSN	n. a.	
Rxanthina_ 16	420	n. a.	WPNVKFP	8.64
Rxanthina_ 17	258	FTIHGLWPNN	n. a.	
Rxanthina_ 18	648	FTIHGLWPSN	FLPVACP	6.38
Rxanthina_ 19	471	FGIHGLWPNY	WPSMSCP	5.94
Rxanthina_ 20	537	QAMAGLWCGS	WPTLACP	5.54
Rxanthina_ 21	408	FSVHGLWPSN	WPDLERG	9.87
Rxanthina_ 22	261	FTIHGFWPSN	n. a.	
Rxanthina_ 23	696	FFIHGLWPSN	WRSFTRR	9.72
Rxanthina_ 24	507	FTLHGLWPVN	WKNYEKG	4.97
Rxanthina_ 25	546	n. a.	WPSLACP	5.07
Rxanthina_ 27	435	n. a.	WPDVKNA	7.78

Rxanthina_ 29	516	FTIHGVWPAN	WPNVERA	9.01
Rxanthina_ 30	204	FTVHGIWPPN	n. a.	
Rxanthina_ 31	465	FTVHGIWPTN	WPSVLTD	7.14
Rxanthina_ 32	216	FSIHGMWPSN	n. a.	
Rxanthina_ 33	450	n. a.	WRSYLIG	7.66
Rxanthina_ 34	411	n. a.	WPSNDNE	4.82
Rxanthina_ 35	207	FTIHGLWPNN	n. a.	
Rxanthina_ 36	447	n. a.	WPSYTGL	8.84
Rxanthina_ 37	411	FHVHGIWPSN	WPSLLTK	6.57
Rminutifolia_ 3	72	n. a.	n. a.	4.66
Rminutifolia_ 4	540	FTIHGLWPSN	WPQLIAR	8.89
Rminutifolia_ 6	168	FGIHGLWPNY	n. a.	
Rminutifolia_ 7	246	YTIHGLWPSN	n. a.	
Rminutifolia_ 8	471	FGIHGLWPNY	WPSMSCP	6.56
Rminutifolia_ 9	252	n. a.	WPQLIAH	5.41
Rminutifolia_ 10	165	FTIHGLWPNN	n. a.	
Rminutifolia_ 11	162	n. a.	WKNYEKG	9.39
Rminutifolia_ 12	567	FHVHGIWPSN	WPSVLTT	6.70
Rminutifolia_ 13	165	FTIHGLWPNN	n. a.	
Rminutifolia_ 14	111	FTVHGIWPTN	n. a.	
Rminutifolia_ 15	504	n. a.	WKSYNHI	5.90
Rminutifolia_ 17	702	FTIHGLWLSS	WPNVVYG	9.67
Rminutifolia_ 18	189	n. a.	WPSIMGK	5.46
Rminutifolia_ 20	432	n. a.	WPNLEIG	8.25
Rminutifolia_ 21	732	FTVHGLWPTK	WPQLKAH	9.23
Rminutifolia_ 23	486	ITVHGLWPSN	WPDVEKG	7.09
Rminutifolia_ 24	210	FHVHGIWPSN	n. a.	
Rminutifolia_ 26	234	FSVHGLWPSN	n. a.	
Rminutifolia_ 27	429	FTIGGIWPYT	WPSLSCP	9.64
Rminutifolia_ 29	306	n. a.	n. a.	5.90
Rminutifolia_ 30	174	n. a.	WPDVEKG	6.95

Rminutifolia_ 31#	459	FTLHGLWPQA	WPDLTHS	6.58
Rminutifolia_ 32	75	n. a.	WPSVMTD	4.80
Rminutifolia_ 33	477	n. a.	WPDLRNG	9.02
Rminutifolia_ 34	624	FTIHGLWPNN	WPDVKNA	8.86
Rminutifolia_ 35	768	FTIHGLWPSN	WPDAHNP	9.17
Rminutifolia_ 36	495	GLWPFN	WKSYMRS	4.90
Rminutifolia_ 38	507	FTIHGLWPTS	WPNVVTP	9.55
Rminutifolia_ 39	423	n. a.	WPTLACP	4.75
Rminutifolia_ 40	177	n. a.	WPSLYGK	4.78
Rminutifolia_ 41	411	n. a.	WPSNDNE	4.93
Rminutifolia_ 43	195	n. a.	n. a.	8.66
Rodorata07_1	186	FTIHRLWASN	n. a.	
Rodorata07_2	123	FHVHGIWPSN	n. a.	
Rodorata07_3	654	FSIHGMWPSN	WPSYTGL	9.13
Rodorata07_4	234	FSVHGLWPSN	n. a.	-
Rodorata07_6	468	FGIHGLWPNY	WPSMSCP	6.56
Rodorata07_7	369	FGIHGLWPNY	WPSMSCP	7.06
Rodorata07_8	297	n. a.	WPSVVTN	7.01
Rodorata07_9	372	n. a.	n. a.	9.36
Rodorata07_10	231	FTIHGLWPSN	n. a.	
Rodorata07_11#	471	FTLHGLWPQA	WPNLKHT	9.32
Rodorata07_13	168	n. a.	WPSVLIT	5.36
Rodorata07_15	120	FTVHGIWPTN	n. a.	
Rodorata07_16	201	FTLHGFWPSN	n. a.	
Rodorata07_17	111	n. a.	WPSVRRD	6.92
Rodorata07_18	240	FTIHGLWPSN	n. a.	
Rodorata07_19	126	FTVHGIWPTN	n. a.	
Rodorata07_20	237	FTIHGQWPSN	n. a.	
Rodorata07_21	678	FFIHGLWPSN	WPSFTRR	8.05
Rodorata07_24	189	FTIHGLWPNN	n. a.	
Rodorata07_25	450	n. a.	WRSYLIA	7.65

Rodorata07_26	696	FGIHGLWPNY	WPTLACP	4.79
Rodorata07_27	408	n. a.	WPNVKFW	8.58
Rodorata07_28	150	FTTHGLWPSN	n. a.	
Rodorata07_29	147	FTIHGLWPNN	n. a.	
Rodorata07_30	435	n. a.	WPDVKNA	6.99
Rodorata07_31	282	ITVHGLWPSN	WSDVEKG	8.63
Rodorata07_32	519	FTIHGVWPAN	WPNVERA	9.21
Rodorata07_33	177	n. a.	WPSIMGK	8.11
Rodorata07_34	708	FTIHGLWPSN	WPNVYYY	8.47
Rodorata07_35	294	ITVHGLWPSN	WSDVEKG	8.66
Rodorata07_36	111	n. a.	WPSVVTN	5.35
Rodorata07_37	180	FHVHGIWPSN	n. a.	
Rodorata07_38	417	n. a.	WPNVKYP	7.68
Rodorata07_39	87	YTIHGLWPSN	n. a.	
Rodorata07_40	669	WTIHGLWTCE	WPSNNKN	8.35
Rodorata07_43	258	n. a.	WPSVLTQ	5.80
Rodorata07_44	246	n. a.	WPSTYKE	6.10
Rodorata07_45	444	n. a.	WPDLRNG	6.40
Rodorata07_46	420	n. a.	WPDVIHG	6.99
Rodorata07_47	470	FTIHGLWPSN	WPDVKRT	10.07
Rodorata_gigantea_1		Identical to Rodorata07_6		
Rodorata_gigantea_2		Identical to Rodorata07_ 34		
Rodorata_gigantea_3	441	FTTHGLWPSN	WPDVEKG	7.64
Rodorata_gigantea_4		Identical to Rodorata07_46		
Rodorata_gigantea_5	345	FTLHGLWPQA	WPDLTHS	8.87
Rodorata_gigantea_6		Identical to Rodorata07_30		
Rodorata_gigantea_7	339	n. a.	WKSYGSG	9.54
Rodorata_gigantea_8		Identical to Rodorata07_26		
Rodorata_gigantea_9	246	FSVHGLWPSN	n. a.	
Rodorata_gigantea_10	237	n. a.	WPDLKNG	8.89
Rodorata_gigantea_11		Identical to Rodorata07_32		
Rodorata_gigantea_12	378	n. a.	WKSYKRS	4.99

Rodorata_gigantea_13	390	n. a.	n. a.	9.22
Rodorata_gigantea_14	216	n. a.	n. a.	9.14
Rodorata_gigantea_15		Identical to Rodorata07_29		
Rodorata_gigantea_16		Identical to Rodorata07_18		
Rodorata_gigantea_17		Identical to Rodorata07_9		
Rodorata_gigantea_19		Identical to Rodorata07_21		
Rodorata_gigantea_20	215	FTIHGLF	n. a.	
Rodorata_gigantea_21	273	n. a.	n. a.	5.35
Rodorata_gigantea_23		Identical to Rodorata07_8		
Rodorata_gigantea_24		Identical to Rodorata07_16		
Rodorata_gigantea_25	180	FHVHGIWPSN	n. a.	
Rodorata_gigantea_26		Identical to Rodorata07_35		
Rodorata_gigantea_28	237	n. a.	WKSYKRS	5.33
Rodorata_gigantea_29	129	FTIHRLWPSN	n. a.	
Rodorata_gigantea_30		Identical to Rodorata07_21		
Rodorata_gigantea_31#	237	FTLHGLWDQN	WPDLSAD	9.06
Rodorata_gigantea_32		Identical to Rodorata07_10		
Rodorata_gigantea_33	108	n. a.	n. a.	4.57
Rodorata_gigantea_36		Identical to Rodorata07_26		
Rodorata_gigantea_37	162	n. a.	n. a.	9.23
Rodorata_gigantea_38		Identical to Rodorata07_29		
Rodorata_gigantea_39	105	n. a.	WPDISGK	5.41
Rodorata_gigantea_40		Identical to Rodorata07_2		
Rodorata_gigantea_41	126	n. a.	n. a.	8.61
Rodorata_gigantea_42		Identical to Rodorata07_24		
Rodorata_gigantea_43	108	n. a.	n.a.	8.99
Rodorata_gigantea_44		Identical to Rodorata07_6		
Rodorata_gigantea_45	105	n. a.	n. a.	8.06
Rodorata_gigantea_46		Identical to Rodorata07_45		
Rarvensis_1	180	n. a.	WPSVLTD	5.39
Rarvensis_2	660	FTIHGLWPYN	WPDAHSV	8.88

Rarvensis_3	420	n. a.	WPSIMGK	7.79
Rarvensis_4	633	WTIHGLWAYR	WPSNNPN	8.38
Rarvensis_6	179	YTIHGLWPSN	n. a.	
Rarvensis_7	177	n. a.	WPSYTGL	7.91
Rarvensis_8	237	n. a.	WPSVLTQ	5.02
Rarvensis_9	357	FLLHGLWPAN	n. a.	
Rarvensis_10	546	FTLHGLWPFN	WKSYMRS	8.20
Rarvensis_11	396	n. a.	WPNLEYG	7.09
Rarvensis_13	312	n. a.	n. a.	9.46
Rarvensis_14	153	FTVHGIWPTN	n. a.	
Rarvensis_15	216	FTIHGLWPNN	n. a.	
Rarvensis_16	246	n. a.	WPSLARP	8.60
Rarvensis_18	195	FSVHGLWSSN	n. a.	
Rarvensis_19	690	FTIHGVWPAN	WPNVERA	8.96
Rarvensis_20	423	n. a.	WPNLTGD	6.39
Rarvensis_21	418	n. a.	WPDVIHG	6.58
Rarvensis_22	126	FTIGGIWPYT	n. a.	
Rarvensis_23	173	FTIHGLWPSN	n. a.	
Rarvensis_24	444	n. a.	WPDLRNG	7.63
Rarvensis_25	540	FTIHGLWPSN	WPQLIAR	8.87
Rarvensis_26	453	n. a.	WPSFTRR	8.80
Rarvensis_27	576	FGIHGLWPNY	WPTLACP	4.73
Rarvensis_29	120	FTLHGIWASN	n. a.	
Rarvensis_32	267	FGIHGLWPNY	n. a.	
Rarvensis_33	699	FTLHGLWPQA	WPDLTHS	8.52
Rarvensis_34	246	FTIHGLWPSN	n. a.	7.87
Rarvensis_35	210	FTIHGLF	n. a.	
Rarvensis_36	223	n. a.	WPSMSCP	7.97
Rarvensis_38	408	n. a.	WPNPIPN	6.55
Rarvensis_39	408	ITVHGLWPSN	WPDVEKG	8.86
Rarvensis_40	87	FWIHG	n. a.	
Rarvensis_41	108	n. a.	n. a.	8.96

Rmajalis_1	294	FTLHRLWHHN	WPDLSRD	9.51
Rmajalis_2	408	n. a.	WPDLTHS	9.08
Rmajalis_4	114	n. a.	n. a.	7.95
Rmajalis_5	189	n. a.	n. a.	7.06
Rmajalis_6	231	FTLHGFWPSN	n. a.	
Rmajalis_8	132	FTVHGIWPTN	n. a.	
Rmajalis_9	189	n. a.	WPSIMGK	6.29
Rmajalis_10	108	n. a.	WPSVMTD	4.80
Rmajalis_11	531	FTIHGFWPSN	WPDLADG	8.80
Rmajalis_12	315	n. a.	n. a.	9.70
Rmajalis_13	441	n. a.	WPNLERR	6.46
Rmajalis_14	651	FTLHGLWPFN	WKSYNHI	4.92
Rmajalis_15	204	FTIHGLWPSN	n. a.	
Rmajalis_16	243	n. a.	WRSYPIA	6.06
Rmajalis_18	366	n. a.	n. a.	6.20
Rmajalis_19	150	FTIHGLWPSN	n. a.	
Rmajalis_20	645	FTIHGLWPNN	WPDVKNA	8.74

without introns

n. a. not available

In blue are amino acids not allowed in the motifs described in Vieira *et al.*[47].

Supplementary Table 3. Location of the *SFB/SLFL/ SFBB* like sequences of *R. chinensis* chromosome 3, *R. multiflora* sc0006888, and *R. multiflora* sc0001861.

F-box genes	Location	
Rchinensis_F-box-4	1507747	1509120
Rchinensis_F-box-3	5375622	5376560
Rchinensis_F-box-2	5433148	5434392
Rchinensis_F-box-1	5475850	5477151
Rchinensis_F-box+1	5558222	5559472
Rchinensis_F-box+2	5618754	5619989
Rchinensis_F-box+3	5720698	5722023
Rchinensis_F-box+4	5734186	5735463
Rchinensis_F-box+5	5751338	5752603
Rchinensis_F-box+6	5803902	5805155
Rchinensis_F-box+7	5808024	5809247
Rchinensis_F-box+8	5856394	5857662
Rchinensis_F-box+9	5879526	5880743
Rchinensis_F-box+10	6536594	6537988
Rchinensis_F-box+11	7143024	7144325
Rchinensis_F-box+12	7149272	7150558
Rchinensis_F-box+13	7425762	7426949
Rchinensis_F-box+14	9568955	9570373
Rchinensis_F-box+15	14254770	14255927
Rchinensis_F-box+16	17725765	17727120
Rchinensis_F-box+17	19789495	19790751
Rchinensis_F-box+18	19818837	19820111
Rchinensis_F-box+19	21203124	21204203
Rchinensis_F-box+20	21569130	21570335
Rchinensis_F-box+21	26018972	26020213
Rchinensis_F-box+22	28383240	28384418
Rchinensis_F-box+23	28397189	28398376
Rchinensis_F-box+24	36186560	36187792
Rchinensis_F-box+25	38266966	38268198
Rchinensis_F-box+26	43723185	43724546
Rmultiflora_sc0006888_F-box-4	15225	16445
Rmultiflora_sc0006888_F-box-3	62159	63515
Rmultiflora_sc0006888_F-box-2	122932	124119
Rmultiflora_sc0006888_F-box-1	210234	211466
Rmultiflora_sc0006888_F-box+1	310084	311322
Rmultiflora_sc0001861_F-box-3	47382	48596
Rmultiflora_sc0001861_F-box-2	141247	142485
Rmultiflora_sc0001861_F-box-1	226346	227578
Rmultiflora_sc0001861_F-box+1	253971	255152
Rmultiflora_sc0001861_F-box+2	383393	384625

Supplementary Table 4. *R. chinensis* transcriptome data used in this study

Tissue	SRA Acc. number
Stamen	SRX5973706, SRX5973707
Pistil and Ovary	SRX5973701, SRX5973702
Leaf	SRX5973703, SRX5973704
Stem	SRX5973699, SRX5973700
Root	SRX5973697, SRX5973698

Supplementary File 1
>Rmultiflora_1
ATGTCTCTAGGTTCATATGATCATTTTAAATTTGTACAACAATGGCCACCGGCCGTCTGTGATACGTCTGGCTGCAAGA
GATCGGGGTTATCACACTTCACGATCCATGGCCTTTGGCCAAATAATAAAACGTATGTGAAAAATCAACCGACTTGTCT
TACCAGTCATGCCAGCTCATTTAATTCGAATTTGGTAAGCAAGCTGAGCACCTCTTGGCCCGATGTGAAAAATGCAAAT
GATAATGATTTTTGGAACAAACAATGGGACAAGCATGGCACATGTTCCTTGCAGACATACGACCAAGCACAATACTTCG
AGCGGTCATACAACATGTGGAAAGAAACCAACCTGACAAACACCTTGAATAGCCTCATCAAACAGACACCGAGACAACA
GAACGTCACAGATATAGAACAACTCATTCAAGGAGTAACTGCAACTGAAAAGAAACCCCTCCTCCGTTGTGAACAATCT
CATAGTAATGCCAGTAAGTCTCTCTTGAAAGAGATTGTCATTTGCTATGCACACAACGGGATTACTGTGATCGATTGCG
TCAGTCGACCAAAATCCACCTTCGAATGCTATGACGGATTTTGGTTTCCT
>Rmultiflora_3
ATGCTTATTTCTACATTTGCTAAAGCCGCAAACAAATATGACTATCTACAACTAGTGCAACAATGGCCTAAAACGTTCT
GCCATAATAACCGAGCTTGCATTCAAGGTGCAGTCCTCCCGGAGCTGTTCTCGATACATGGTATGTGGCCATCTAATTT
CTCCGGCCAGAACGACGCTTGTGTTGGAACTCGGTTCAGCATGAGAGAGATGCGTCGACACAATCATCTCGAAACTCAA
TTGCTGTCATCCTCATGGCCAAGCTACACTGGTCTGTCCAACCTGCACTTTTGGGAGTATGAGTATAACAAGCACGGCA
CTTGTTCAGAGAATAACCTCAAACAGACGGACTATTTCACCAGAGCCGACGCTTTGTGGAGGCGCTACAATGTTACCAA
TATGCTTTTAACATCGCGCCACCAAATCTCGCCGGGATCCTCCTATCGTTACGCTGACATTTTGTATGCCATTCGACAA
GGAATTGGGGGATACACTCGTTTGATTCTTTGCAAGAAAGATCCGGCAAATAATATTTGGATTCTGCACGAGGTGATAA
TTTGTTTCGATCCCTTGGGGAATAACGTCATCGCTTGTCGCAGAAGATCGAGATATAGTTGCAACAGCGGG
>Rmultiflora_4
TCACAATTAATTTCCACAGGGTCCTATGACTATTTCAAATTTGTTGTACAATGGCCACCAGCCCACTGCAGTGTTTTTA
AATGCCACAATAGTAACCCGCCACACATTTACACCATCCATGGCCTATGGCCAAATAATCGTTCTAAGGCAGTCGGGAA
TAAGTGCCAGGGATCGTCATTTCAAAAGCCGGCTCCTCCATTGGAAGCCAAACTGAAGATCTCTTGGCCTAACCTAGAA
AATTCGAGTGATGCGCACTTTTGGGAACTTCAGTGGAACAAACATGGCAAGTGTTCGGAGCCGACATTTACGCAATCCC
AATACTTCGATCGAGCCCATCAAATTTGGATGATGGAGAATATTAATATTACTAGTATCCTCCAAAATGTAAACATCGT
ATCAGGGAAAACAAAAAGCTACGAAGAGATAGAATTTCCCATTGAATCAAAAACTCACAAGACACCACTCCTTCACTGC
ATAAACACTCAGAAGTTGCATGAAGTGGTACTTTGCTGGGACCATGCTGCAAAAAAATTGATCGACTGTAAACATACAG
AAGCAACATGCTCAAGGAATAGTCCAATTGAGATTCTA
>Rmultiflora_6
ATGCAGAGCCATACGAATACCTACAGTTTGTCTTGCAATACCCTAGAGGTTTACTGTGTTAACGACAAGAAATGCATTC
CACCGGCGAGCTTGCCAGCCAAATTCACGGTTCATGGAATTTGGCCCACGAATATTTCCGAGCCGATTATCAGCTGTGA
CAAGGCACATAAACTACACTCGTTCAACGGAAATCTGATAACTCCGTCGTTGAAAACCGATCTTCTCCAATCTTGGCCC
AGCGTCGTGACAAATAAAGATGATATGTCGTTTTGGGAACACGAATATAATAAGCATGGGTCCTGTACTGCACCAGCTA
TCACTCAGACAGCGTATTTTGAGAGAGCTCACAAGTTCTGGAAGGAGTATGATCTCTATTCCATTTTGGAACAAAAAAA
TATCAAGCCGGGGCAATCTCGATCGTATTCACTGGCTGATTTTGAAGCAGCCGTCAAGTCCAAGATCAGTACCGACACA
AAACCTCTCATCCTTTGTAAGCAGGACAAACTAACTCGTGGGAGCACAACAACCAACTTCATTTTGAGGGAAATTGTGA
TCTGTTTCGATCACCAGGGGACAAATCTGGTCAATTGTACCCGATCAACAGATTGTGACCAGAAGCAGAAGATATACTA
CCCT
>Rmultiflora_8
CAATACTCATTTGTTTTTGTGTCAAGGCCTCATTTTGGAATTTTAATGTTCGTTTGTTTCTTGTCACAATTAATTTCCA
CAGGATCGTATAAATATTTCAAATTTGTTGTACAATGGCCACCAGCCCACTGCAGTGTTTTTAAATGCCACAATAGCAA
CCCGCCAAAGATATACACCATCCATGGCCTATGGCCAAGTAATCGTTCTAATGCAGCTCGCAGTAAGTGCCAGGGATCG
TCATTTCAACAACCGGCTCTTCCATTGGAAGCCAAACTGAAGATCTCTTGGCCTAACCTAGAAAATTTGAGTGATGCGC
AGTTTTGGGAACTTGAATGGAACAGACATGGCAAGTGTTCGGAGCCGACATTTACGCAAACCCAATACTTCAATCGAGC
CCATGAAATTTGGATGATGGATGACATTAATATTACTGATATCCTCCAAAATGTTCACATCGTATCAGGGAAACAAAAA
ACCTACGCAGAGATAGAATTTCCCATTGAATCAAAAACTCAAAAGACAATCCTCCTTCGCTGCCTAAACCCTCAGAAGT
TGCATGAAGTGGTAATTTGCTGGGACCATGCTGCAAAAAATATGATCGACTGTAATCGTACAGAAGCAACATGCTCAAA
CAGCAGTCTAATTGATGTTCTA
>Rmultiflora_9
TACCCACCTGGAGTTTGCTATGACACATCAAAAGGTTATTGCATCTCACCTCTGCCAACCAAGTTCCATGTACATGGAA
TATGGCCTTCTAACTTCTCCGATATACATGTCACTTTGATATGTTTTTCTAATTGTATCTTTTATTTTCAGATGACACC
TCTGCAGACGGATTTGGTAAACTCATGGCCGTCCGTGTTAACAACAAAGAGCAATATGTGGTTCTGGGAGCATGAGTAC
AAGAAACATGGCGCATGTACAGTAGAGTCAGGTGTACCACCTTTCACTCAAAAGTCCTACTTTGAGAAAGGACACCAGC
TATGGAACCAATATGACATCCATTCAGTGCTTGATCAAAGTGGTATTAAGCCGAGTACTGCAACGTCATACACTATGAC
TCAGCTTGTAACCGCAATCCAAAAGAAAATCAGGACTAGCAATACTCCTTTAATCCTGTGCAGGAGGACAACCCTTGGT
GACACACTAAAGGAAGTGGTAATCTGTCTGGATTACCAGGCAACAAATGTGATAAGTTGCGCGCTCAGTAAAAGGAAAA
CAGATTGCCAGGATCCTTCGGGCAAGGTATACTATGTAGCT
>Rmultiflora_11

ATGAAAACCCCAGCAGCAGTCTGTCTTATTTTCTTGCTTTCTGTTATTCTGCTCAAGGGTTCCCACGCAGAGCCATACG
AGTATCTACAATTTGTTTTGCAATACTCCAAAGGCTACTGCTACAACACCCAGATATGTTTTGCGAGGTTGCCAAAAAT
GTTTACCATACACGGTGTCTGGCCCGCGAACATTTCCAATCCCCTGGTGAGATGTAAACAGTCAAAGAAACTACACCAG
TTCAAGCAAAACATGACAACATCACTGCAAAACGATCTTGGCCAATCTTGGCCCAATGTCGAAAGAGCTAAAACCAATA
TAGGGTTTTGGAAAGAGGAATACGAAAAGCATGGGTCTTGTACTGCACCAGCTATCACACAGAAAGCGTACTTTGAGAG
AGCTCACGAGCTGTGGAAGGAGTATGATCTCTATACAATTTTGGATAAAAAGCAGATCAAGCCGGGGCACTCATATGCA
CTCACTGATTTTGAAGCAGCCGTCAAGTCCAAGATCGGTACCGATACAATACCTCTCATCCTGTGCAAAGAGGACACAC
TAACTACTGGGAGCAGCACAACTGGATTAATTCTGAGGGAAATAGTTATCTGTTTCGATCACCAGGGAACAAATCTGGT
CAATTGTACCCGGCCGACAGATTGCAAGCGGCAGAAGAGCACAACTAAAGACAACATGATATACTACGTACCT
>Rmultiflora_15
ATGAAGAATTCAAACTTCATTATCCTTCTATCAGTATATGCACTTGTACAAGTTCTCAACGTTTCAAATGCTGAACCCT
ACGAGTACATGCAATTTGTACTCCAGTACCCACCTGGAGTTTGCTATGACAAATCAAAAGGTGATTGCATCTCACCTCT
CCCAACCAAGTTTCATGTACATGGAATATGGCCTTCTAACTTCTCCAATATACTTGTCCCAATATGTTTTTCTAATTGT
ACCTTTTATTTTCAGATGACACCTCTGCAGACGGATTTGGGAAACTCATGGCCGTCCGTGTTAACACAAAACAACAATA
TGCAGTTCTGGGAGCATGAGTATAGACAGCACGGTGCATGTGCAGTAGAGTCAGGTGTACCACCTTTCACTCAAAGGTC
CTACTTTGAGAAAGGACACCAGCTATGGAACCAATATGACATCCTTGCAGTGCTTCATCAAAGTGGTATTAAACCGGGT
ACTGCAACATCATACACTATGACTCAGCTTGTAACCGCAATCCAAAAGAAAATCGGGAGTAACAATACTCCTTTAATCA
TGTGCAGGAGGACAGCCCTTGGTTACACACTAAGGGAAGTGATAATCTGTCTGGATCACCAGACAACAAATGTGATAAG
TTGTGCGCTCAATAATATTAGAAAAACAGATTGCCGAGATCCTTCGGGCAAGGTATACTATGTAGCT
>Rmultiflora_16
ATGGCATTGCTAAGTACAATAGCCGTGCTGCTTGTTTCTGCTTTTTCATCAGGTGCTAAAGCCGCCAATAAGTACGACT
ACTTCCAGTTTGTCCAGCAATGGCCGGCTACATTCTGCTATCAAAATTCGGATTGTGTCCGGAATCCCCCGCGGAATAC
TTTTACCATCCACGGCCTCTGGCCAAGTAATTACACTGCACCTGAAAAGCCATGCATGTTTGCTGCCCCAAACCATGGT
TTGAAAAACTATTTTCTGCCGCAAGCATGGCCCCAGTTAATTGCTCGTTTCACCGACATGCAATTCTGGCAGACCGAAT
ATAACAAGCACGGTACATGCTCGGAGAACAAACTCAAGCAGACGGAGTATTTTAGAACAGCTCACTGGGCGTGGGTTCG
ATTCAATGCATATTTTCTGTTTGCACGCTCGCCTCTCCAAATCCACCCTGGTAATTACTATTTTAAAGCTGACCTTGAA
CAAGCCATTCGGACAGTCACTACTAAGGACCCTCTGCTTACGTGCAAGAAGATCAAACCCATGGGCATTGATACGTGGA
TATTGCAGGAAGTCATAATCTGTCTTGATGTTTTGGGAGTCCAACCGGTCCCTAGTGGCCCTCGATTACCAACTTGCCG
TGGTCCAATAATATATTATGGTTACCAACTAATTTATATA
>Rmultiflora_21
ATGATCAGAGATGCGGTCGGACAGCCGGTCCCCCAGCCTGTCGGAGAGCTGGTCCCATCACTCCCATGGAGGTTCGGAC
GGCCGTATTATGAGTACCTGCGATTTGTACTGCAAGTTCCCAGAACATTGAATCCAAGCTCAACACTTAACCAGAATTT
CACCATTCATGGCCTATGGCCTAGCAATCACAGCACTCATAATGGTGGAATTCCTTATGTTCAAAATTGTGATCCAAAT
TATATCCTTGATCCTCAACAGGAGCTCAGTCACTCTTGGCCCGATGTAAAGCCGACTGCAAACGATTCGAAGTTTTGGA
CCGACGAATACAGGAAGCACGGCACCTGCGCAAACCGTATATTCCGTACACCAGGGGATTACTTTAGAAAAGCAGATGA
CTTGTATCGCAGTCATCCCATACAACGTTTTTTTAATCTATTCGCCGACCACATCCGCCCAGGACAGCAAGTCCCCGTG
AGCACATTTATTAATCTCGTACCAACGGCGACAGCAAGGAAGAATGCACGACTGTGGTGCAATTGGAATGGAACCCTCT
ACCTACTGAAGGAAGTAGTTATTTGTTACAATAACACCTCGTTTGGTTCACGAAATGAATTTCTTAGAAAAGTTGATCC
TTTGTATTTCTATCGGGAAGGGAAATTAAGATTCCTACTAATGTGTTTGATAACACTAAAAAATGGAATCAATAAT
>Rmultiflora_24
ATGTCTTCATTTCTCACCATTTTTCTCTGTTTTCAACTGCTCCTAGCGGTGCCAAATCCACTTCGAGAGAAAATTTCAG
CTCCTGATTCCTTCCTGTTGGTAATTGTTTGGCCCAATACCTTCTGTCTTTTTCAGAGCAATCCATGTCAACAGCTTCC
ACAATCTTTTACGTTACACGGATTGTGGCCGCAGGCGGAAGGTTCCTCGTTGAAATGTACGAGTGTACCAATGATTGAT
AGCATCTTAAAAGGCAACAAAGATGATTTGGAAAGGTATTGGCCAAACTTGAAGCACACAAAGTTCGATGAGAGTAAAA
AATTCTGGATTAGTGAGTGGATTAAACATGGTAGCTGCTCCGCAAAGACTCCTGCTAATTACTTGAGCCTGGTTTTTGA
TCTAATGAAGAAAATCAAGAAATTTGATGTGAAAAAGATATTTGAAAAGCACGGTAAGATA
>Rmultiflora_25
ATGGGTGGAGCAACAGTGGCATCAGTCATTGTGCTATTTTTCCTTCCACTCGGTGTGATTGGAAATGCGGTCGGACAGC
CGAATCACAATTATGACTACATGCAATTTGTGCAGCAATATCCCAAAACATTGAATGTAAGCTCAGAATATACCGAGCG
TTTCACCATTCATGGCCTATGGCCTAGCAATTCCTTCGGCTACGGTTCTGATCTACATTGTCAAACACCATATGCTAAC
CGTTATCAACTTTCAGGACAGCTGACCAACGAACTCAGAAACACTCTCAGTTACTCTTGGCCCAATGTTTACTACTATT
GGCACGTTACAAAGTTTCAAAACAATTTGATCTTTTGGTACAACGAATACCAGAAGCACGGCAGCTGCGCTGTGGCCCT
TCTGGGTATATTCCCAACAGCGCAGGCTTACTTTGAAAGAGCACATAAGTTGTGGGAAGCAAATGATTTGTATGGTGTA
TTCGTCAACTACAACATCCACCCGGGAGACCGAGTCGACGCTAGCAAATTTAGGACAGCCCTGAGAATTGCGACTGGAT
GGAACGCTCGACTGTCATGCACTGGAACCGGCCTAATGATGGAAGTAGTTATTTGTTATGATATTCATGCAACTCGAGT
GAGGAATTGTCCTAGACCGGGGAATTGTACGGGGCTTATATCATATCCAAAA
>Rmultiflora_27

ATGGAAATCATTTATTTTCTTGCTTTCATATTATTTTTATTTCCCGCAACTGTGCCAGCACAGACAAAAGGTTACGAGT
ATATCATGCTGGTCCTCCAAAATCCTTTTAACAGGAATGGCGCTAGTCCTTGGACCATACATGGCCTGTGGGCCGAGAG
ACTTCAAGGACCATCTCCTAACTATAAATTTGTTCCAGTTAATAGTATTAATAGCGACATTGAAGCTGCACTAATTAAA
GCTTGGCCCAGTATTATGGGTAAACCAAACCAAAGAGTTTGGGAACATGAATGGAGAAAACACGGTTCATGTACCACAG
ATTTATTACCAACTTCTAATGACTACCTACTGAAAGGAGTTTACCTGTGGGAGCAAATCAAGTTTCATGATTTGATGGG
TGTGGGTCCGGGTGCCCAATTTAAGCCAAATACTCAATATGATGCGAACACGGTGCTGGCGGCCATCACAAGCAAATAC
AAGGTCAAAACAATGCTGACCTGTCAGGGTGGGGAAATATTGGATGTTAGGTTTTGTTATTTAGGACCTTGGGTACTGG
TAGATTGCCTGGACACCCAACCACCTCCCCATACAGTTTGTACAGGTTTAATTTATTATCCTAGAATA
>Rmultiflora_28
ATGGAAATCATTTATTTTCTTGCTTTCATATTATTTTTATTTCCCGCAACTGTGCCAGCACAGACAAAAGGTTACGAGT
ATATCATGCTGGTCCTCCAAAATCCTTTTAACAGGAATGGCGCTAGTCCTTGGACCATACATGGCCTGTGGGCCGAGAG
ACTTCAAGGACCATCTCCTAACTATAAATTTGTTCCAATTAATAGCGACATTGAAGCTGCACTAATTAAAGCTTGGCCC
AGTATTATGGGTAAACCAAACCAAAGAGTTTGGGAACATGAATGGAGAAAACACGGTTCATGTACCACAGATTTATTAC
CAACTTCTAATGACTACCTACTGAAAGGAGTTTACCTGTGGGAGCAAATCAAGTTTCATGATTTGATGGGTGTGGGTCC
GGGTGCCCAATTTAAGCCAAATACTCAATATGATGCGAACACGGTGCTGGCGGCCATCACAAGCAAATACAAGGTCAAA
ACAATGCTGACCTGTCAGGGTGGGGAAATATTGGATGTTAGGTTTTGTTATTTAGGACCTTGGGTACTGGTAGATTGCC
TGGACACCCAACCACCTCCCCATACAGTTTGTACAGGTTTAATTTATTATCCTAGAATA
>Rchinensis1_6_Rchinensis2_1
ATGTCTGGTATTCGTATGACGATCCTGAATGGGGTGGCTCTTGCATTTGTTCTTTCCTTGTGTATCCCCATGTGCACCG
GTTCATATGATCATTTTAAATTTGTACAACAATGGCCACCGGCCGTCTGTGATACGTCTGGCTGCAAGAGATCGGGGTT
ATCACGCTTCACGATCCATGGCCTTTGGCCAAATAATAAAACGTATGTGAAAAATCAACCGACTTGTCTTACCAATCGG
GCCAACTCAATTAAGGCTACGATACTCACTAGTTCGAATTTGGTAAGCAAGCTGAGCACCTCTTGGCCCGATGTGAAAA
ATGCAAATGATAATGATTTTTGGAACAAACAATGGGACAAGCATGGCACATGTTCCTTGCAGACATACGACCAAGCACA
ATACTTCGAGCGGTCATACAACATGTGGAAAGAAACCAACCTGACAAAAACCTTGGATAGCCTCATCAAACAGACACCG
AGACAACAGAACGTCACAGATATAGAACAACTCATTCAAGGAGTAACTGCAACTGAAAAGAAACCCCTCCTCCGTTGTG
AACAATCTCATAGTAATGCCAGTAAGTCTCTCTTGAAAGAGATTGTCATTTGCTATGCACACAACGGGATTACTGTGAT
CGATTGCGTCAGTCGACCAAAATCCACCTTCGAATGCTATGACGGATTTTGGTTTCCT
>Rchinesis1_1_Rchinensis2_12
ATGAAATCCCATTCCCCCATCTTCTGTTTGATACTCTCTGCTCTCATAAGCCTGTCAACAACTATTCTAACCGCTCAAG
AAAATCCTGTTTCCTTCTACATTGCTTATCAGTGGCCAGGCTCATACTGTGCAGCAGCAAAGCAGGGTTGTTGCTACCC
AAAGAGTATTAGAAAACATCCCAGTTTCACTATTGGTGGCATATGGCCTTACACTTTTTCTGGGGATAGACCAACCTAC
TGCAAATCTAAGACCCCTTTTAGTTTATCTAAGATATCAAACCTGACCAAGAGTTTGGAAAGGAATTGGCCGGCCATCA
CTATCCTGCCCAAGCCGTAC
>Rchinesis1_3_Rchinensis2_27
ATGGCAATGTTGAAATCATCGTTCGCTTTAATTGCTCTTGCTTTTGCTCTCTGTTTCACTATGAGCATTGGTTCCTATG
AATATTTTAAATTTGTGGTACAATGGCCACCAGCCCACTGCAGGGTTAGAAAATGCACACCCCAAGCCTTGCAACAGAA
AATTTACACCATCCATGGCCTATGGCCGAGTAATTATTCTAAAGCTGTTGTGTATAATTGTCCGGGATCGTCTTTTCAA
AATCCGGCTCCTCCATTGGAAGCCAAACTGAAGATCTCTTGGCCCAACCTAGAACGTCCGAACGATGCAATGTTTTGGG
CACTTGAGTGGGACAGGCATGGCAAGTGTTCGGAGCAGACATTTACACAAACCCAATACTTCAATCGAGCCCATGAAAT
TTGGGTGGGGAAGAATATTACTGACATCCTCCAGAGAGCTAGCATCTTATCGGGGAGACAAAAAGACTACGGAGTTATA
GAATTAGCCGTACGATCAAAAACTCAAAAGACACCCCTCCTTCGCTGCGAACAACCAAAGCAGAAACCTACGCAGAAAC
CTACGCAACCTACGCAGCGGACTCAGTGGTTGCATGAGGTGGTACTTTGCTGGGACTATCATGCCAAAAATATGATTGA
CTGTGATGATACAGAAGCAACATGTCAAGATACTTTTCCAATCGATATTCTG
>Rchinesis1_4_Rchinensis2_15
ATGAAAACAGCAGCAGTCTGTCTCCTTTTCTTGCTCTCTGTTATACTGTTCAAGGGTTCCCATGCAAAGCCATACGAAT
ACCTACAGTTTGTCTTGCAATACCCTAGAGGTTACTGTGTTAACGACAAGAAATGCATTCCACCGGCGAGCTTGCCAGC
CAAATTCACGGTTCATGGAATTTGGCCCACGAATATTTCCGAGCCGATTATCAGCTGTGACAAGGCACAGAAACTACAC
TCGTTCAACGGAAACCTGATAACTCCGTCGTTGAAAACCGATCTTCTCCAATCTTGGCCCAGCGTCGTGACAAATAAAG
ATGATATGTCGTTTTGGGAACACGAATATAATAAGCATGGGTCCTGTACTGCACCAGCTATCACTCAGACAGCGTATTT
TGAGAGAGCTCACAAGTTCTGGAAGGAGTATGATCTCTATTCCATTTTGGAACAAAAAAATATCAAGCCGGGGCAATCT
CGATCGTATTCACTGGCTGATTTTGAAGCAGCCGTCAAGTCCAAGATCGGTACCGACACAAAACCTCTCATCCTTTGTA
AGCAGGACAAACTAACTCGTGGGAGCACAACAACCAACTTCATTTTGAGGGAAATTGTGATCTGTTTCGATCACCAGGG
GACAAATCTGGTCAATTGTACCCGATCAACATATTGTGACCAGAAGCAGAAGATATACTACCCT
>Rchinesis1_8_Rchinensis2_25
ATGAAAACCCCAGCAGCAGTCTGTCTTATTTTCTTGCTTTCTGTTATTCTGCTCAAGGGTTCCCACGCAGAGCCATACG
AGTATCTACAATTTGTTTTGCAATACTCCAAAGGCTACTGCTACAACACCCAGATATGTTTTGCGAGGTTGCCAAAAAT
GTTTACCATACACGGTGTCTGGCCCGCGAACATTTCCAATCCCCTGATGACAACATCACTGCAAAACGATCTTGGCCAA
TCTTGGCCCAATGTCGAAAGAGCTAAAACCAATATAGGGTTTTGGAAAGAGGAATACGAAAAGCATGGGTCTTGTACTG

CACCAGCTATCACACAGAAAGCGTACTTTGAGAGAGCTCACGAGCTGTGGAAGGAGTATGATCTCTATACAATTTTGGA
TAAAAAGCAGATCAAGCCGGGGCACTCATATGCACTCACTGATTTTGAAGCAGCCGTCAAGTCCAAGATCGGTACCGAT
ACAATACCTCTCATCCTGTGCAAAGAGGACACACTAACTACTGGGAGCAGCACAACTGGATTAATTCTGAGGGAAATAG
TTATCTGTTTCGATCACCAGGGAACAAATCTGGTCAATTGTACCCGGCCGACAGATTGCAAGCGGCAGAAGAGCACAAC
TAAAGACAACATGATATACTACGTACCT
>Rchinesis1_11_Rchinensis2_23_Rchinensis3_5
ATGAAGAATTCAAACTTCATTATCCTTCTATCAGTATATGCACTTGTACAAGTTCTCAACGTTTCAAATGCTGAACCCT
ACGAGTACATGCAATTTGTACTCCAGTACCCACCTGGAGTTTGCTATGACCAATCAAAAGGTCATTGCATCTCACCTCT
CCCAACCAAGTTTCATGTACATGGAATATGGCCTTCTAACTTCTCCAATACACTTGTCCGTTGTGGGCACGCACTGAGG
AATAACCCCTTTCATAAGGTGCAGATGACACCTCTACAGACGGATTTGGGAAACTCATGGCCGTCCGTGTTAACACAAA
ACAACAATATGCAGTTCTGGGAGCATGAGTATAGAGAGCACGGTGCATGTGCAGTAGAGTCAGGTGTACCACCTTTCAC
TCAAAGGTCCTACTTTGAGAAAGGACACCAACTATGGAACCAATATGACATCCTTGCAGTGCTTCGTCAAAGTGGTATT
AAACCGGGTACTGCAACATCATACACTATGACTCAGCTTGTAACCGCAATCCAAAAGAAAATTGGGAGTAACAATACCC
CTTTAATCATGTGCAGGAGGACAGCCCTTGGTTACACACTAAAGGAAGTGATAATTTGTCTGGATCACCAGGCAACAAA
TGTGATAAGTTGTGCGCTTAATAATATTAGAAAAACAGATTGCCGAGATCCTTCAGGCAAGGTATACTATGTAGCT
>Rchinensis2_5
TTTGTCCGGCAATGGCCGGCTACATTCTGCTATCATAATTCGGATTGTGTCATCCCCCGCACAGTACTTTTACAATTCA
TGGCCTTTGGCCAATATGAACTTGATCAGATGTTTGCTGCCCCAAACCATGGTTTGAAAGACTATTTTCTGCCGCAAGC
GTGGCCCCAGTTAATTGCTAGTTTCACCAACATGCAATTCTGTCAGAGCGAATATAACAAGCACGGTACCTACTCGGAG
AACAAACTCAAGCAGACGGAGTATTTTAGGACAGCTCACTCAATGCATATTTTCTGTTTGCACACTCGCCTCTCCAAAT
CCACCCTGGTAAAGCTGACCTTGAACAAGCCATTCGGGCAGTCACTACTAAGGACGCTCTGCTTATGTGCAAGAAGATC
AAACCCATGGGCATTGATACGTGGC
>Rchinensis2_6_Rchinensis3_15_Rchinensis4_7
ATGATGTCTTCATTTCTCACCATTTTTCTCTGTTTTCAACTGCTCCTAGCAGTGCCAAATCCACTTCGAGCGAAAATTT
CAGCTCCTGATTCCTTCCTGTTGGTAATTGTTTGGCCCAATACCTTCTGTCTTTTTCAGAGCAATCCATGTCAACAGCT
TCCACAATCTTTTACGTTACACGGATTGTGGCCGCAGGCGGAAGGTTCCTCGTTGAAATGTACGAGTGTACCAATGATT
GATAGCATCTTAAAAGGCAACAAAGATGATTTGGAAAGGTATTGGCCAAACTTGAAGCACACAAAGTTCGATGAGAGTA
AAAAATTCTGGATTAGTGAGTGGATTAAACATGGTAGCTGCTCCGCAAAGACTCCTGCTAATTACTTGAGCCTGGTTTT
TGATCTAATGAAGAAAATCAAGAAATTTGATGTGAAAAAGATATTTGAAAAGCACGGTAAGATA
>Rchinensis2_7_Rchinensis3_11_Rchinensis4_4
ATGATTAATACGAGAGCAAAAGAATCTCGTTTCACTCTTCACGGGCTTTGGCCCTTCAACGCCACTCAATTAGATCCAA
ACAATATTAATTCTGATTTCCAGTATAGACCTATCATAGAGGGTGCCCAGTTACCTATTGTCTTACTATATAAACTCGC
AAAATCTTGGAAAAGTTATATGCGTAGCTATCCGAATGGGGATTTTTGGAAAGATGAATACGACAAGCATGGAACATGC
ACCGTTAAAAAATTTCGTACACCTGTCGAGTACTTCCAGAAGGCAAATGACTTGTGGGAAGCCAACCCAATAGAGGATT
GGTTTCGGCGTGATGGTCGCTTCACAGATACAGATGTCCAGCTCGCTGAGCTGAGAGGCGCGATCATGAACAAGTTCGG
TTCTGTGCCATGGTTTCAGTGTGAGGACAAATTTGTGCGGCAGATAGGCTTGTGTTTTGATGAGGTTGGAGGGTTGGGT
GCGAATTGTCCTAAAGAATATCAAAAGAAGCAATGCCCCCCTACTATACAGTATAGATTG
>Rchinensis2_8_Rchinensis4_12
ATGGTCCTACAGAATCCTTCGACCTTAAAAGGCAAGGCTCCCTGGGGCATACATGGTATTTGGGCAGAAAAACTTGATC
CACTTGTAGGGCCATCTCCACAAAGGTATAAGTATGTGCCAGTTCAATCGTGTCAATCATTTCCTCCATTTGACCCTAA
GATTAGTCAAAAAATTACAAAGGCACTCGATAAAGCTTGGTCGAGTCTTTCGGCTACGTGGCCAAACGTTAAATTTTGG
GAACATGAATATAACAAACACGGTTCATGTATCGTAGATTTATTACCAACTTCTACGGATTACCTAATGAAAGCACTTT
ACCTGTGGGAGCAACTCAAGTTTGACGAGGTGATGGGTAAGGGTAAGCAATTTGAGCCAAATACACTATACAAAACCGA
GGATATTCTTAAGGGCATCAAAGTTTCACATAATGTTCGACCAGAATTGACCTGTATCGGTAATAAACTAACAGATGTT
AGGTTTTGTCATACAAGAAATTGGGTATTGGTAGATTGCGAGGGCATCCAAACATCTTGTACAGGTCAAATTAAATATG
TTAGGCAAGTG
>Rchinensis2_11_Rchinensis3_13_Rchinensis4_38
ATGAGAACACCAGCAGTCTGTCTTATTTTCTTGCTCTCTGTTGGTCTGTTCAAGGGTTCCCATGCAGAGCCATTCGAAT
ACCTACAGTTTGTCTTGCAATACGCTAGAGGTTCCTGTGTTAACGTCAAGAAATGCATTCCACCGGCGAGATTGCCGGC
CAAATTCACGGTTCATGGAATTTGGCCCACGAATATTTCCAAGCAGGAGGTGATCGAGTGTACAGCTGCCGTGAAACTA
CACTCGTTCGACGGAAACCTGATAACACCGTCGCTGAAAACCGATCTTCTCCAATCTTGGCCCAGCGTCCGGAGAGATA
AAGATAATATGACTTTTTGGGAACACGAATATAATAAGCATGGGTCCTGTACTGCACCAGCTATCACTCAGACAGCGTA
TTTTGAGAGAGCTCACAAGTTCTGGAAGGAGTATGATCTCTATTCTGTTTTGGAACAAAAAAAATCAAGCCGGGGCAGT
CTCGATGGTATTCACTGGCTGATTTTCAAGCAGCCGTCCAGTCCAAGATTAGTACCGATTATATACCTCTCATCCTGTG
CAAGGAGGAAGCAT
>Rchinensis2_16_Rchinensis3_17_Rchinensis4_40
ATGTCTACAGGGTATTACGAATATTTCAAACTTGTGCAACAATGGCCACCAACCACTTGCCAAAATGCAAACTGCCGCA
GAGTACCGCCACCTCGCCTCTTTACCCTCCATGGGTTTTGGCCATCTAATTATTCAAACAATGTCGTGGCTAATTGCAC

TAACGCAATATTTCAACGGATGGATCGCTCATTGGTATCTAAACTGAATATATCCTGGCCCAATGTGAAATATCCGAAT
AATATCCAGTTTTGGGATAAACAGTGGAGAAAACATGGCTCATGTTCCGTGCACACGTTTAACCAAACAGAGTACTTCA
CGCAAGCGGATAATCTTTGGAACTCACACAACATTACTGATATTCTTATAACTGGTGGGATCAAGCCAAATGGATCAGA
ATATGCATACGATACGGTAGAGCGGCCCATCCAAATTGCAACCGGGAAGGAACCCGAACTTCGTTGCGCACCATCCTCC
CTTGGAAGGCAATTGTTGCATGAAGTAGTACTTTGTTATAATCATAAGGGAACAACGCCCATCGACTGTAACCCTTTAC
ATTCAACCTGCGATCGTAACTTCCAAATAAAGTTTATT
>Rchinensis2_18
ATGCTGGTCCTCCAAAATCCTTTTAACAGGAATGGCACTAGTCCTTGGACCATACATGGCCTGTGGGCCGAGAGACTTC
AAGGACCATCTCCTAACTATAAATTTGTTCCAGTTAATAATTGTCCATCAGCCCCTCCATTTAACCGTGAGATTAATAG
CGACATTCAAGCTGCACTAATTAAAGCTTGGCCCAGTATTATGGGTAAACCAAACCTAAAATTTTGGGAACATGAATGG
GGAAAACACGGTTCATGTACCACAGATTTATTACCAACTTCTAATGACTACCTACTGAAAGGAGTTTACCTGTGGGAGC
AAATCAAGTTTCATGATTTGATGGGTGTGGGTCCGGGTGCCCAATTTATGCCAAATACTCAATATGATGCGAACACGGT
GATGGCGGCCATCACAAGCAAATACAAGGTCAAAACAATGCTGACCTGTCAGGGTGGGAAATATTGGATGTTAGGTTT
TGTTATTTAGGACCTTGGGTACTGGTAGATTGCCTGGACACCCAACCACCTCCCGGTACAGTTTGTACAGGTTTAATTT
ATTATCCTAGAATA
>Rchinensis2_24_Rchinensis4_33_Rchinensis3_4
ATGAAGAATTCAAACTTCATTATCCTTCTATCAGTATATGCACTTGTACAAGTTCTCAACGTTTCAAATGCTGAACCCT
ACGAGTACATGCAATTTGTACTCCAGTACCCACCTGGAGTTTGCTATGACCAATCAAAAGGTCATTGCATCTCACCTCT
CCCAACCAAGTTTCATGTACATGGAATATGGCCTTCTAACTTCTCCAATACACTTGTCCGTTGTGTGCACGCACTGAGG
AATAACCCCTTTCATAAGGCGCAGATAACACCTCTGCAGACCGATTTGGGAAACTCATGGCCGTCCGTGTTAACACAAA
AAAACAATATGCAGTTCTGGGAGCATGAGTATAGAGAGCACGGTGCATGTGCAGTAGAGTCAGGTGTACCACCTTTCAC
TCAAAGGTCCTACTTTGAGAAAGGACACCAGCTATGGAACCAATATGACATCCTTGCAGTGCTTCGTCAAAGTGGTATT
AAACCGGGTACTGCAACATCATACACTATGACTCAGCTTGTAACCGCAATCCAAAAGAAAATCGGGAGTAACAATACTC
CTTTAATCATGTGCAGGAGGACAGCCCTTGGTTACACACTAAAGGAAGTGATAATCTGTCTGGATCACCAGGCAACAAA
TGTGATCAGTTGCGCGCTCAATAATATTAGAAAAACAGATTGCCGAGATCCTTCGGGCAAGGTATACTATGTAGCT
>Rchinensis2_28
ATGAAGAATTCAAACTTCATTATCCTTCTATCAGTATATGCACTTGTACAAGTTCTCAACGTTTCAAATGCTGAACCAT
ACGAGTACATGCAATTTGTACTCCAGTACCCACCTGGAGTTTGCTATGACAGATCAAAAGGTTATTGCATCTTACCTCT
GCATGCCAACCAAGTTTCATGTACATGGAATATGGCCTTCCAACTTCTCCGATATAGAATGATATATTTTTCTAATTGT
ATCTTTTATTTTCAGATGACACCTCTGCAGACGGATTTAGTAAACTCATGGCCATCCGTGTTAATAACAAAGAGCAATA
TGTGGTTTTGGGAGCATGAGTACGAGAACCATGGCGCATGTACAGTAGAGTCAGGTGTACCACAATTCACTCAAAAGTC
CTACTTTGAGAAAGGACACCAGCTATGGAACCAATATGACATCCATTCAGTGCTTGATCAAAGTGGTATTAAACCAAGT
ACTGCAAAATCATACACTATGACTCAGCTTGTAACCGCAATCAAAAAGAAAATCGGGAGTAACAATACCCCTTTAATCA
TGTGCAGGAAGAAAAAAACTTGGTTACGTACTAAT
>Rchinensis2_29
ATGATCAGAGATGCGGTCGGACAGCCGGTCCCCCAGCCTGTTGGAGAGCTGGTCCCATCACTCCCATGGAGGGTCGGAC
GGCCGTATTATGAGTACCTGCAATTTGTACTGCAAGTTCCCAGAACATTGAATCCAAGCTCAACACTTAACCAGAATTT
CACCATTCATGGCCTATGGCCTAGCAATCACAGCACTCATAATGGTGGAATTCCTTATGTTCAAAATTGTGATCCAAAT
TATATCCTTGATCCTCAACAGGAGGTGAGGTTATTGCTGAGCCGTAACCTCCAAAGGAATCTCAGTCACTCTTGGCCCG
ATGTAAAGCGGACTGCAAACGATTCGAAGTTTTGGACCGACGAATACAGGAAGCACGGCACCTGCGCAAACCGTATATT
CCGTACACCAGGGGATTACTTTAGAAAAGCAGATGACTTGTATCGCAGTCATCCCATACAACGTTTTTTAATCTATTC
GCCAACCACATCCGCCCAGGACAGCAAGTCCCCGTGAGCACATTTATTAATCTCGTACCAACGGCAACAGCAAGGAAGA
ATGCACGACTGTGGTGCAATTGGAATGGAACCCTCTACCTACTGAAGGAAGTAGTTATTTGTTACAATAAGACCTCGTT
CGGTTCACGAAATGAATTTCTTAGAAAAGTTGATCCTTTGTATTTCTATCGGGAAGGGAAATTAAGATTCCTATTAATG
TGTTTGATAACACTAAGAAATGGAATCAATAAT
>Rchinensis2_30
AACGATGTGGCTTTTGTCGTCACTGCTCTAGGTTTGGTCTTCAGTCTGACGTCATCATATACACCCTACCAATACCTGT
TGTTTGTTCAACGGTGGCCAATGTCTGTTTGTTATGATATCAACTGCCCTAACACTTCCCCTCCAATCATCACGGTACA
CGGTTTATGGCCATCGAACTATAATCGACCTCGCTTGAAGTGCTCTGGCGTTGTGACATCTAGTGAAACACTGGTAACA
CATATTATTATGCGATTATATATGAGTCACCAAACAGTACTAAAAGCAAACTTGGATCAACATTCTTGGTCGGATGTGG
AGAAGGGAAACCATATAGGTTTCTGGAAGCATGAGTGGCAGGCTCATGGACAGTGTTTAGACACCGTCTTTCCAGTTTC
CACATACCAAATATTTCCAATGGTGTCGCGATATGTGGAAGAAAAACGTGATAGGCGAGATCCT
>Rchinensis2_31
ATGGCATTCCTAGCAGTTAGGACACTAATTGTTCTTATGCTTATTTCTACATTTGCTAAAGCCGCAAACAAATATGACT
ATCTACAACTAGTGCAACAATGGCCTAAAACGTTCTGCCATAATAACCGAGCTTGCATTCAAGGTGCAGTCCTCCCGGA
GCTGTTCTCGATACATGGTATGTGGCCATCTAATTTCTCCGGCCAGAACGACGCTTGTATGCGTCGACACAATCATCTC
GAAACTCAATTGCTGTCATCCTCATGGCCAAGCTACACTGGTCTGTCCAACCTGCACTTTTGGGAGTATGAGTATAACA
AGCACGGCACTTGTTCAGAGAATAACCTCAAACAGACGGACTATTTCACCAGAGCCGACGCTTTGTGGAGGCGCTACAA

TGTTACCAATATGCTTTTAACATCGCGCCACCAAATCTCGCCGGGATCCTCCTATCGTTACGCTGACATTTTGTATGCC
ATTCGACAAGGAATTGGGGGATACACTCCTTTGATTCTTTGCAAGCAAGATCCGGCAAATAATATTTGGATTCTGCACG
AGGTGATAATTTGTTTCAATCCCTTGGGGAATAACGTCATCGCTTGTCGCAGAAGATCGAGATATAGTTGCAACAGCGG
GTTGATAGACTATCCTAAG
>Rchinensis2_32_2
ATGAGATACAGCACTAGTTTGATTTTGATCAAACTATTGGTAATTCAATACCTGTCAGTTCTTTGCGCTTCGCAGGACT
TTGATTTCTTCTACTTCGTTCAACAGTGGCCTGGAGCATATTGCGACAAAAAGCATAGTTGTTGCTATCCAAAGTCAGG
GAAGCCTGCAGCAGATTTCGGCATTCACGGTCTCTGGCCAAACTACAAGGATGGCTCTTACCCTTCAAACTGTGATCCA
GACAGTGTCTTCGACAAATCTGAGATCTCAGAGTTGATGGGCAGTCTGGAAAAGAGTTGGCCATCTATGAGCTGCCCAA
GCAGTAATGGTTACAGGTTCTGGTCACACGAATGGGAGAAGCATGGCACTTGCTCCGAATCTGAACTTGATCAGAAAGA
TTACTTCCAAGCCGGTCTCAAGCTCAAGGAAAAAGCAAACCTTCTTCAAGCCCTTAAAAAAGCTGGTAATTTAGTTCGT
CTATACTCAATTGTTTATAAGAAATTAACCAAGCTAGAT
>Rchinensis3_3_Rchinensis4_1
ATGTTATTGAATCTAATCAGCTGCTCTTATATAAGTTTTGACATTCTTCCTTTCTGTTATTTTTTTGTTTTTTTTTTTT
CCAACACAATAATGTCTCTAGGTTCATATGATCATTTTAAATTTGTACAACAATGGCCACCGGCCGTCTGTGATACGTC
TGGCTGCAAGAGATCGGGGTTATCACGCTTCACGATCCATGGCCTTTGGCCAAATAATAAAACGTATGTGAAAAATCAA
CCGACTTGTCTTACCAATCGGGCCAACTCAATTAAGGCTACGATACTCACTAGTTCGAATTTGGTAAGCAAGCTGAGCA
CCTCTTGGCCCGATGTGAAAAATGCAAATGATAATGATTTTTGGAACAAACAATGGGACAAGCATGGCACATGTTCCTT
GCAGACATACGACCAAGCACAATACTTCGAGCGGTCATACAACATGTGGAAAGAAACCAACCTGACAAAAACCTTGGAT
AGCCTCATCAAACAGACACCGAGACAACAGAACGTCACAGATATAGAACAACTCATTCAAGGAGTAACTGCAACTGAAA
AGAAACCCCTCCTCCGTTGTGAACAATCTCATAGTAATGCCAGTAAGTCTCTCTTGAAAGAGATTGTCATTTGCTATGC
ACACAACGGGATTACTGTGATCGATTGCGTCAGTCGACCAAAATCCACCTTCGAATGCTATGACGGATTTTGGTTTCCT
>Rchinensis3_6_Rchinensis4_44_2_SRNase30
ATGGAAACGGCATTGCTATGTACAGTCTCCATGCTGGTTATTTCTGCTTTTCCATCCGGTGCTAAAGCCGCGAATCAGT
ACGACTATCTTCAATTTGTTCAACAATGGCCGATAACCTTGTGCTACAACAACCCAGCCTGTATTCCAGGAGCATCACT
CCCACTGGACTTCTTTATCCATGGGTTATGGCCAAGCAATTTCTCTGGCCAGAATCAGCCCTGTGTTGGTACTCCGTTC
GACTATAATGAGATGCTTAGTCAATATCGTCTCCGAACCAAAATGCTACCATTCTCATGGCCAAGCTTCACTCGTAGAT
CCAACATGGGCTTCTGGAAATATGAGTATAATCAACACGGCACTTGTTCGGAGAATAACCTCGCGCAGACGGACTATTT
TTACAAAGCCTATGCTCTCTGGATGCGCTACAATGCAAACTTTATATTATATGCATCTACAGTTATACCAGGCCACAAG
ATTGTGCCGGGATACCTCTACCATTACGCTGACCTTCTAAACTCCGTTCAACAAGCAATTGGTGGATTCACTCCTTCGC
TTATGTGCAAGCACGAGCCTGCAAATAATACTTGGATTCTGCACGAAGTCATCATATGTTTTGATGCCATGGGGAACAA
CGTCATCAATTGTGTTAGAGGATCTAGTTGCTCTGCCACTACCACCGGGTATATACACTATCCTTTGCAA
>Rchinensis3_9_Rchinensis4_28_SRNase26
ATGGGGATTAATGTCATCCTGATTCTTCTCTGCCTTATCAGCAACGTTGCTAGTGCAGCCAACACATACGATTATCTTC
AACTAGTACTGCAATGGCCAAACACGTTCTGCATCAGTAACCAAAAGTGCATATCAAATCTCCCACTAGACTTCACCAT
ACATGGGTTATGGCCTAGCAATTTCTCCGGCCAAAATAACCCATGTGTGGGTGCGCCATTTAATAGAGCTCAGATGTCC
GCGAACTACCGTCTTCAAACCATATTGCTCCCATCCTCGTGGAGAAGCTACCTTATCGCTGTCTCCAACACAAACTTCT
GGCAGACTGAGTATAACAAACATGGTACATGTTCGGAGCTCAATTTGCCACAGACGGAATACTTCACCAAAACCCATTG
GCTATGGATGGTCAACAACATTTATAGTATATTTTCGTCTTCGGTAAACAGACAAGGATATCCAATCTTGCCGGGTTTC
ACCTATGACTACGCCGATCTTCTTGCCGCAGTTCAACTAGGAATCGGTGGCGTCACTCCCGTGCTTACTTGCATGGTCA
TAAATAACAACCAATACGTTCTTCACGAAGTGGTTATTTGTTATGATGCCTTGGGGATCAACCGCATCCATTGTGTGCG
ACAATCGAGTTGCTCTTTCGCTCCTAACGGACGTGTGCTCTATCCT
>Rchinensis3_10_Rchinensis4_44_SRNase36
ATGGCACAAAATAATATGGCTTACGTCCTCATGACCTGCACTACTGTTGCCTTACTCTTTACTCTGGCCTCCTCGTATA
CCGCCTACGAATACTTCTTGTTTGTTCAACAGTGGTCCAAGACCGTTTGTGGTAACAGCTGCAAAGCTCCCTCTCCAGT
TTTTACGATACATGGGTTGTGGCCCTCCAACCACACTGGCCCTCAGCTGAAGTGCACTGGTGCAGCATATAATCCCACA
GAGATGAACAACCAACCCATCTTGAAAAAGAACCTTGAAACTAAATCATGGCCAGATGTTATTCATGGGCAGCATGAGA
TTTTCTGGATACACGAATGGGGCTTCCACGGCAAATGTTCAGATCTCGTCTTCCCACAGACGAAGTATTTTGAGCGCAG
CCATGAAATGTGGACCGATATGGTCATAGGAGATATGCTTAAGAAGGCGTCCATCGTCCCCGCGGCCGGAGCAAAGTAT
AGCCTTCCCGACATTGAAAAGGCCATCAAAGCCAAGACTTCGCACGACCCATTAATCCGCTGCTTCAAGAATCAGTTGA
AGGAAGTAGCATTATGTTACGATTACTATGGCAAAAACGTGGTTGACTGTGATAAATCGCAAAGCAATTGCCCTCAAGC
TCCAACTCTTATAGAATATCCGAAC
>Rchinensis3_16
ATGTGTTGGCCAGGATCCTACGGTGGCCCTCAGCACAATATTTGCTATCCAACTACAGGAAAACCTGCACAAAGTTTCA
TTATCGGGGGCTTTCGGCCAGGTTTTAACAATGGTTCAATCCCAACCTACTGCGATCCCAACAACCCCTTCAATGAAAC
AAAGGTATACAGTGTCAGGGATAATCGACGATTTGCAAAAATATTGGCCATCGGCTGGCCTGCCCGAGCAGCAACGGTA
CGCAGCTTTGGGCCGAAGAATGGGAAAAACATGCATGGCACTTGCTCGCAGTCTGTCCTTGATCGGTATAATTACTTCT

TTCATGCTACCGGCCTCAGAGATACATTGAAAGACATCCACGGATACCTTCAAGATTATGGAATCCGACCAGATGGGAC
AGCGTACAACCTAAACACCCAAATATTATCTGAGAAAATCATTTCTTGGCCTCAA
>Rchinensis4_8
GCATTTAACATTCTTTTTCTCTCAATAATATCTACAGCGAGCTACCAATATTTTCAGTTTGTGCAGCAATGGCCACCAA
ACTTCTGCTACGGTAGATATCGTCCTTGCAATAGGCAATCATTCACCATTCATGGCCTGTGGCCAAATAATTTTTCACA
AGTTAATTACAATCATATTTGTTCAGGGGCCGCATTTAAAAATCAGATGTATTTCAATCAAACACTGGTAAACGACCTG
ACTCGCTCTTGGCCGGACCTGAGAAATGGGAACCATCAAAGGTTTTGGGAAGAGCAGTGGGATAAACATGGCAAATGCT
CGGATCAGACGTATGGGCAAACGCGATACTTCCAGCGCGCCTTCGAGATGTGGAACCAATTCGATATTACTGATATCTT
TACTGCCGCTGGCGTCGTATCTCCGCCAGGGACACAACCAAGTAAAGTTAACGCGAGCGACTTAGAAGCACGCATTCAA
GCAGTAACTCATATGGCCCCTATCCTTCGGTGCAGAAAAGTTACAGTGCAGAACGTTCAACGGCCAGTTGAGCTAATTT
ATGAAGTGGGCATTTGTTACGATTATCATGGAACAAATCTGTATCCTTGTACCAGCACACAAGGAAGATGCGGCGTAAA
TGATATATACTTT
>Rchinensis4_31
ATGAAGAATTCAAACTTCATTATCCTTCTATCAGTATATGCACTTGTACAAGTTCTCAACGTTTCAAATGCTGAACCCT
ACGAGTACATGCAATTTGTACTCCAGTACCCACCTGGAGTTTGCTATGACCAATCAAAAGGTCATTGCATCTCACCTCT
CCCAACCAAGTTTCATGTACATGGAATATGGCCTTCTAACTTCTCCAATACACTTGTCCGTTGTGGGCACGCACTGAGG
AATAACCCCTTTCATAAGACGGATTTGGGAAACTCATGGCCGTCCGTGTTAACACAAAACAACAATATGCAGTTCTGGG
AGCATGAGTATAGAGAGCACGGTGCATGTGCAGTAGAGTCAGGTGTACCACCTTTCACTCAAAGGTCCTACTTTGAGAA
AGGACACCAACTATGGAACCAATATGACATCCTTGCAGTGCTTCGTCAAAGTGGTATTAAACCGGGTACTGCAACATCA
TACACTATGACTCAGCTTGTAACCGCAATCCAAAAGAAAATTGGGAGTAACAATACCCCTTTAATCATGTGCAGGAGGA
CAGCCCTTGGTTACACACTAAAGGAAGTGATAATTTGTCTGGATCACCAGGCAACAAATGTGATAAGTTGTGCGCTTAA
TAATATTAGAAAAACAGATTGCCGAGATCCTTCAGGCAAGGTATACTATGTAGCT
>Rchinensis4_41
ATGAAAACAGCAGCAGTCTGTCTCCTTTTCTTGCTCTCTGTTATACTGTTCAAGGGTTCCCATGCAGAGCCATACGAAT
ACCTACAGTTTGTCTTGCAATACCCTAGAGGTTACTGTGTTAACGACAAGAAATGCATTCCACCGGCGAGCTTGCCAGC
CAAATTCACGGTTCATGGAATTTGGCCCACGAATATTTCCGAGCCGATTATCAGCTGTGACAAGGCACAGAAACTACAC
TCATATTACTCGGCTTGTTTTGTTTTTCAGATAACTCCGTCGTTGAAAACCGATCTTCTCCAATCTTGGCCCAGCGTCG
TGACAAATAAAGATGATATGTCGTTTTGGGAACACGAATATAATAAGCATGGGTCCTGTACTGCACCAGCTATCACTCA
GACAGCGTATTTTGAGAGAGCTCACAAGTTCTGGAAGGAGTATGATCTCTATTCCATTTTGGAACAAAAAAATATCAAG
CCGGGGCAATCTCGATCGTATTCACTGGCTGATTTTGAAGCAGCCGTCAAGTCCAAGATGGGTACCGACACAAAACCTC
TCATCCTTTGTAAGCAGGACAAACTAACTCGTGGGAGCACAACAACCAACTTCATTTTGAGGGAAATTGTGATCTGTTT
CGATCACCAGGGGACAAATCTGGTCAATTGTACCCGATCAACAGATTGTGACCAGAAGCAGAAGATATACTACCCT
>MDP0000267606AT2Lineage
ATGATTCTCTCTGCTCAATTCGCCTCTGCAGTAGCACTGATTGCGGTGGGCGCTTCTCTGTGCCTGATCGATGCCAAGC
AAGCCGGAATCGGATTCGAAATCGGAAGCAGAGGAGGAGGAGGGCAGAGGGAGTTCGATTACTTCAACTTGGCCCTGCA
ATGGCCTGGCACTTTCTGTCAGCGCACCCGCCATTGTTGCTCCTCCAATGCTTGCTGCCGCGGCTCAAATGCTCCAACC
ATGTTTACAATCCATGGATTGTGGCCTGACTACAATGATGGAACTTGGCCTGCCTGTTGCACACGGAAAACCTTTGATG
ATAAGGAGATCTCAACATTGCACGATGCTTTAGAGAAATACTGGCCATCTTTAAGCTGTGGTAAACCATCATCCTGCCA
TGGTGGAAAAGGATCATTTTGGGGTCATGAGAAGCACACTTGCTCCTCTCCAGTAGTTGGAGATGAATACAATTACTTT
TTGACAAATGTCTATTTTAAGTACAATGTCACTCAAATCCTGAATGAAGCAGGATATGTACCATCCAATACTGAAAAAT
ATCCTCTTGGAGGCATTGTTTCTGCTATTCAGAATGCTTTCCGGGCAACCCCGAGGTTGGTTTGCAAAAAAGGGGCCTT
GGAGGAACTTCATCTATGCTTCTACAAGGATTTCCAGCCTCGGGATTGTCTGGTTGGATCTGGCAGTCTAAGTGACAAG
TTAGCTTCAAGTAGCTCATGTCCCAGTTTTGTCAGCATACCAGCATATGCATCATTGGATTCCTGCAGCTCAACTCTTA
ACCTGCAGAATGTCAATCTGCCC
>M_domestica_MDP0000160706_Malus_S_RNase_lineage_2
ATGGAGTCGAAGTTAGTGCAAGTCATATTTTTCTTCTTGTTTGTCACAGTTTCATCCGCTTACACACCATTCCAATACT
TTATGCATGTTCAGTTCTGGCCAGCTGCTGAATGCCAAGCTACAGGGGGGAATAAATGCCACATCATGCAACCATCTCA
TCTTCAGTTCACTATTCATGGTCTCTGGCCTGCGAATAAATCATCCTCTTCATCCCTTGGCTGCAAGGGGGATGCCTAT
AATGTGAATGGGATGAATGATACACTGAAAAAGGAGCTACTCAGTTCGTGGTGGGATTGGAGAAAAGGGGAGCATGTCG
AATTCTGGCAACGAGAGTATGATAAGCACGGAAAGTGCTCCGACAATGTGTTCCTGAAGACCGAGTACTTCCGGAAGAC
CCTAGCGATGTACCATGATTTTGATATAGCTCAAATCCTGCAGAAGGCTAATATTGTACCCCAACTACTTCAGCCCAAG
ACGTCGTTGTACAAATTGTACTCCATCGATCAGATAACAAAGGCCATTGAGTCCAAAACCGGCGGACATTCCGTCAATA
TCAGATGCTATCAACAAAACCAGAAGAAGAGCAACAATGGAAGTAAACCATTAATATATTTGGCCCAAGTCGCCCTCTG
CTATAATAGATCTGGCAATAGCAGAACTAACTGTGATCCAGGCGAAACAACCAGCTGCAACTTTAATGAATCAGAACCG
AAAACTGTACCAAGAATAGCCCACATAAAGCTCTGG
>M_domestica_MDP0000250548A_S_RNase_lineage_1
ATGCACTTCTTCAAGATTGCTCTCCTTGTCCTCACTCTTTACTTCATCACCTCTCTCGGAAAGATCAATGCTGCCACAC
CATATGACATCTTCCAGTTCGTCCAGCAATCCCCATTGGCCTTCTGCTACGGAACCGATATCTGCGGAGACCAACCAGT

ACTGCCCCGTACCTTTACGACGCACGGCCTCTGGCCAAGCAGCAGCACCAATCCAGCTGCTCCTTTAACCTGCGCTGGT
ACCGCATTCAGCCGTAGTGAGATGAATGATCCCGACAATCAATATCTGCAACTACTTTTGTCCTATTCCTGGCCCAACT
TCAACATTAGACAAACAAATATGGATTTCTGGGAGTATGAGTACAACAAGCACGGCAGGTGCTCGGACAATACATTTTC
CCAGACACAATACTTCCGTGAAGCTTATAGATTGTGGTCTACCTACAATGCACTCCATCTATTTTCTCAAACAACTTGG
ATTGTACCAGGCTATCCATATCGTTATATCGACCTTGAATTGGCCATTCGACGGACTATAGGAGGGAAAGCACCTCTTC
TTATGTGCAAGTATAACGGGGTGAGCCCGTATCTGGTGGAAGTCGTCATCTGCTTTGACTACACTGCAGCGAATCCGGT
CGATTGTGTCAGGACAACAAATTGTGGAAATCCTAATGCATTGGGCAAATTTTCATCTAATATATGGTGGAATAGGCAG
>M_domestica_S2_RNase_MDP0000345854
ATGGGGACTACGCGGATGGTATATATAGTTACGATGTTATTTTCATTAATTGTATTAATATTGTCTTCGTCTACGGTGG
GATACGATTATTTTCAATTTACGCAGCAATATCAGCCGGCTGCCTGCAACTCTAATCCTACTCCTTGTAAAGATCCTCC
TGACAAGTTGTTTACGGTTCACGGTTTGTGGCCTTCAAACATGAATCGAAGTGAATTATTTAATTGCAGTAGTTCAAAC
GTGACTTATGCGAAGATACAAAATATCCGAACCCAGTTGGAAATGATTTGGCCAAACGTATTCAATCGAAAAAATCATT
TAGGCTTCTGGAATAGAGAGTGGAACAAACACGGCGCCTGTGGGTATCCCACAATACGGAACGACTTGCATTACTTTCA
AACAGTAATCAAAATGTACATAACCCAGAAACAAAACGTCTCTGATATCCTCTCAAAGGCCAAGATTGAACCGGATGGA
AATATCAGGACACAGAAGGAAATTGTAGATGCCATAAGAAAAGGTATCCATGGTAAGGAACCAAAACTCAAGTGCCAAA
AGAATACTCAGATGACTGAATTGGTTGAGGTCACTCTTTGCAGCGATGGCAACTTAAAGCAGTTCATAGATTGCCCCCA
CCATTTTCCAAATGGATCACGACATAACTGCCCCACCAATCATATTCTG
>M_domestica_S3_RNase_MDP0000266136
ATGGGGATTACAGGGATGATATATATGGTTACAATGGTATTTTCATTAATTGTATTAATATTGTCTTCGTCCGCGGTGA
AATTCGATTATTTTCAATTTACGCAGCAATATCAGCCGGCTGTCTGCAGCTCTAATCCTACTCCTTGTAAGGATCCTCC
TGACAAGTTGTTTACGGTTCATGGTTTGTGGCCTTCAAATGTTAATGGAAGTGACCCCAAGAAATGCAAAACTACAATC
TTGAACCCTCAAACGATAACAAATCTTACAGCCCAGCTGGAAATTATTTGGCCAAACGTACTCAATCGAAAGGCTCATG
CACGCTTCTGGCGTAAACAGTGGCGTAAACATGGCACCTGTGGGTACCCCACAATAGCGGACGACATGCATTACTTTAG
CACAGTAATCGAAATGTACATAACCAAGAAACAAAATGTCTCTGAAATCCTCTCAAAGGCGAAAATTAAACCGGAGAAG
AAATTCAGGACACGGGACGACATTGTGAATGCCATAAGCCAAAGTATCGACTATAAGAAACCAAAACTCAAGTGCAAGA
ACAATAATCAGATAACTGAATTGGTTGAGGTCGGTCTTTGCAGCGATAACAACTTAACGCAGTTCATAGATTGCCCCCG
CCCATTTCCACAAGGATCACCATTTTTCTGCCCCACCAATAATATTCAGTAT
>P_persica_ppa024151m_S_RNase_lineage_1
ATGTTGTTTCTGTTTCTTGCTTTCTTTTATGTCAACAACATTCCATGTGACTGCAATGCTCCTGGACAGTATGACTACT
TCCAGTTTGTTCAGGAGTCTCCTTTTCTATTTTGCAAAAATATTAATGGAAAGGGATGCAACATCCTACCTCTGCCACG
GATATTTACTATTCATGGCTTGTGGCCAAGCAACTTCACACATCGTCATAAACCTTGCGTTGGTGCACAATTTAGTAGA
GATATAAATGAAGCCTCCAATGATGAATTGCGAGCTGATCTGGAGTTGTCATGGCGAAGCTTCATCAGTGGGCGGTCAA
ATATGGACTTCTGGGAATATGAATACAACAAACACGGCAAGTGCTCCGACGACAAGTTTTCGCAGACGCAATACTTCGA
CCGTGCCAGGAGTTTGTGGAAGCAATACAAACCTCATGCTCTATTTTCTAATCGCTCGCTTGAACCAGGCAAGTCATAT
AGTTTTACTGGCCTTGAACAAGCCATCCGATCATTTATAGGCGGGAGCAGACCTCTTCTCCTTTGCGAGCAATCTAAGA
GGAAGAAGAACAAGAAGAAGACAAAA
>P_persica_S2_RNase_ppa018459m
ATGGGGATGTTGAAATCGTCACTCGCTTTCCTTGTTCTTGTTTTTGCTTTCTTCTTTTGTTACGTTATGAGCAGCGGAT
CTTATGACTATTTTCAATTTGTGCAACAATGGCCACCGACTAACTGCAGAGTTCGCGTCAAGCGACCTTGCTCCAATCC
CCGGCCATTACAATATTTCACCATCCATGGCCTATGGCCAAGCAATTATTCAAACCCAAAGATGCCCAGTAATTGCACA
GGGTCGCAATTTAAGAAACAGAATTTGTACCCTTATATGCAATCCAAACTGAAGATATCTTGGCCGGACGTGGAAAGTG
GGAATGATACAAAATTTTGGGAAGGCGAATGGAATAAACATGGTACATGTTCCGAACGAACACTTAACCTAATGCAATA
CTTCCAGCGATCCCACGCAATGTGGAAATCACACAATATTACAGAGATCCTTAAAAACGCTTCAATCGTACCACATCCG
ACAAAAACATGGAAGTACTCGGACATAGAATCACCCATTAAAAGAGCAACTAAAAGAACACCCGTCCTTCGTTGCAAAC
GTGATCCAGTACAGGCGAATACTCAGTTGTTACATGAAGTGGTATTTTGTTATGAATATGATGCGCTAAAGCTGATTGA
CTGTAATCGAACAGATTGCTGGAATAACGTTGACATTAAGTTTCAA
>P_persica_ppa011133m_Prunus_PA1
ATGCCATTAAGATATGAGATATTGGCAGGGGCTTCTCTGCAAGCTATTACCACTCATGGGCAACCGTATGACTACCTAC
AATATGTACTACAATGGCAGAAGACGAAATGCTTGAAGGTCGGGAAACGGTGCACTCCATATGAGAAGTTCACTACTCA
CGGCCTCTGGCCAACCAACCTCTCCAAGATATTGACATGCAAATCAGCTTCAAAATTTAGCAGCACTATGCTGCAAAAT
AATGCTTCATTGCTATCGAAGATGAAAAAGTCCTGGCCAAATCTGGAGCAGAGAGTTGCCCAAGGAAAAGACAACGATA
TGTGGTTTTGGGCGATGGAGTACGAAAAACACGGTATATGTGCCAAGTTTTCTAGCCAGAATACTTACTTGTCAAAAGC
ATGTGATTTGTGGGAAGAAAACAAGATTAAGGAGATTTTTGAGCACCACAAAATCTTTCCAACAAACGCGACGTCATAT
AAAGATGTTTTGCTTATGAATGCTATTGAAATGGAAACTCGCAGTTCTCCTCTCCTTCTTTGCCATAGAGTCAACGGCG
ATTATTTGTTGTGGGAGGTTGTTCTTTGCTATGACGACACGGCTAAAAAACGGATGAATTGTTCTGATCAAAGTGCAAG
ACAAACAAATTGCGGAACGGACATCTATTATAAA
>P_mume_S_RNase_scaffold241_33

ATGGGGATGCTGAAATCGTCACTCGCTTTCCTTGTTCTTGCTTTTGCTTTCTTCTTCCTATTCATCACAATAATTTTGG
CAGGATCTTACGACTATTTTCAATTTGTGCAACAATGGCCACCGACCAACTGCAGAGTTCGCATCAAGCGACCTTGCTC
CAACCCCCGGCCATTACAATATTTCACCATCCATGGCTTATGGCCCAGTAATTATTCAAACCCAACGGTGCCCAGTAAT
TGTAATGGGTCAAAATTTGACGCAAGGAAAGTGTCACCTCAACTACGAAACAAACTGACGAAATCTTGGCCGGACGTGG
AAAGTGGCAATGATACAAAATTTTGGGAAGGCGAATGGAACAAACATGGTACGTGTTCCGAGCAGACACTTCACCAAAT
GCAGTATTTCGAGATATCCCACGACATGTGGCTGTCCCACAATATAACACATATCCTTAAAAACGCTACAATCGTACCA
AGTGCCACACAAACGTGGAAGTACTCGGACATAGTAACACCCATTAAAATAGCAACTAAAAGAACACCCCTCCTTCGTT
GCAAACCTGATCCAGCACAAAATAAGAGCGGACCGAAGACTCAGTTGTTACATGAAGTGGTACTTTGTTATGGATATAA
TACAAAAAAGCAGATTGACTGTAATCGAACAGCAGGATGCTGGAATAATGTTGACATCAAGTTTCAA
>P_mume_scaffold442_35_S_RNase_lineage_1
ATGGTACTTGACCACCAATTGGCTCGGCTTTCAGTCCCGTTCACATGCTGCAATGCCGCTGGACAGTATGACTACTTTC
AGTTTGTTCAGGAGTCTCCTTTTGTATTTTGCAAAAATATTACTGGAAAGGGATGCAACATCCTACCTCTGCCACGGAT
ATTTACCATTCATGGCTTGTGGCCAAGCAACTTCAAACATCGTCATAAACCTTGCGTTGGTGCACAATTTAGTAGAGAT
ATGAATGAAGCCTCCAATGATGAATTGCGAGCTGATCTGGAGTTGTCATGGCGAAGCTTCATCAGTGGGCGGTCAAATA
TGGACTTCTGGGAATATGAATACAACAAACACGGCAAGTGCTCCGACCACATATTTTCGCAGACGCAATACTTCGACCG
TGCCAGGAGTTTGTGGAAGCAATACAAAGCTCATACTCTATTTTCTAATCGCTCGGTTGAACCAGGCAAGTCATATCGT
TTTACTGACCTTGAACAAGCCATCCGATCATTTATAGGCGGGAGAAGACCTCTTCTCCTTTGCATGCAATCTGAGTGGA
AGAAGAAGACAAAACAATATTTGCTGGAAGTCGTAATTTGCTTTGATGACATAGCAATTAATCCAGTGAATTGTCCCAA
GAGGATGTCAAAAGAATGTCTTCCACAATCCCCTGTATGGTATATGCTA
>M_domestica_SH_RNase_AB032247
ATGGGGACCGGGATGATATATATGGTTATGATGGTATTTTCACTAATTTTATTAATATTGCCTTCGTCCACGGTGGGAT
TCGATTATTATCAATTTACGCAGCAATATCAGCCGGCTGTCTGCAACTCTAATCCAACTCCTTGTAAGGATCCTACTGA
CAAGTTGTTTACTGTTCACGGTTTGTGGCCTTCAAACTCGAATGGAAATGACCCAAAATATTGCAACGCACAGCAATAT
CAGACGATGAAAATACTCGAACCCCAGTTGGTAATTATTTGGCCGAACGTACTCAATCGAAACGATCATGAAGGCTTCT
GGCGTAAACAGTGGGAGAAACATGGCTCCTGTGCGTCTTCCCCAATACAGAACCAGAAGCATTACTTTGATACAGTAAT
CAAAATGTACACAACCCAGAAACAAAACATCTCTGAAATCCTCTCAAAGGCGAATATAAAACCGGGTAGGAAAAACAGG
ACACTGGTGGATATTGAAAATGCCATACGTAATGTTATCAACAATATGACACCACAATTCAAGTGCCAAAAGAATACTA
GGACATCATTGACTGAATTGGTTGAGGTCGGTCTTTGCAGCGATAGCAACTTAACGCAGTTCATAAATTGCCCCCGCCC
ATTTCCACGAGGATCACGGTATTTCTGCCCCACCAATATTCAGTAT
>P_pyrifolia_S9_RNase_AB104909
ATGGGGATTACAGGGATGATATATATGGTTACAATGGTATTTTCATTAATTGTATTAATATCGTCTTCGCCCGTGATGA
AATTCGATTATTTTCAATTTACGCAGCAATATCAGCCGGCTGTCTGCAGCTCTAATCCTACTCCTTGTAGGGATCCTCC
TGACAAGTTGTTTACGGTTCATGGTTTGTGGCCCTCAAATGTTAATGGAAGTGACCCCAAGAAATGCAAAACTACAATT
TTGAACCCTCAAACGATAACAAATCTTACAACCCAGCTGGAAACTATTTGGCCAAACGTACTCAATCGAAGGGCAAATG
TGCGCTTCTGGCGTAAACAGTGGCGTAAACATGGCACCTGTGGGTACCCCACAATAGCGGACGACATGCATTACTTTAG
CACAGTAATCGAAATGTACATAACCAAGAAACAAAACGTCTCTGAAATCCTCTCAAAGGCGAAAATTAAACCGGAGAAG
AAATTCAGGAAACGGGACGACATTGTAAATGCCATAAGCCAAAGTATCGACTACAAGAAACCAAAACTCAAGTGCAAGA
ACAATAATCAGATAACTGAATTGGTGGAGGTCGGTCTTTGCAGCGATAACAACTTAACGCAGTTCATAGATTGCCCCCG
CCCATTTCCACAAGGATCACCATTTTTCTGCCCCACCAATAATATTCAGTAT
>P_pyrifolia_S4_RNase_AB009385
ATGGGAATTACGGGGATGACATATATGTTTACAATGGTATTGTCATTAATTGTATTAATATTCTCTGCGTCCACGGTGG
GATTCGATTATTTTCAATTTACGCAGCAATATCAGCCGGCCGTATGCAACTCTAATCCTACTCCTTGTAACGATCCTAC
TGACAAGTTGTTTACGGTTCACGGTTTGTGGCCTTCAAACAGGAATGGACCTGACCCAGAAAAATGCAAGACTACAACC
ATGAATTCTCAGAAGATAGGAAATATGACAGCCCAGTTGGAAATTATTTGGCCGAACGTTCTCAATCGAAGCGATCATG
TAGGCTTCTGGGAAAGAGAGTGGCTCAAACATGGCACCTGTGGGTATCCCACAATAAAGGACGACATGCATTATTTAAA
AACAGTAATCAAAATGTACATAACCCAGAAACAAAACGTCTCTGCAATCCTCTCAAAGGCGACGATTCAACCGAACGGG
AATAACAGGTCACTGGTGGATATTGAAAATGCCATACGCAGTGGTAACAACAATACGAAACCAAAATTCAAGTGCCAAA
AGAATACTAGGACGACGACTGAATTGGTTGAGGTCACTCTTTGCAGTAATAGAGACTTGACTAAGTTCATAAATTGCCC
CCACGGGCCTCCAAAAGGATCACGATATTTCTGCCCCGCCAATGTTAAGTAT
>P_ussuriensis_S35_RNaseDQ839240
ATGGGAAATACGGGGATGATATATATGTTTACAATGGTATTTTCATTAATAGTATTAATATTGTCTTCGTCAACGGTGG
GATACGATTATTTTCAATTTACGCAGCAATATCAGCCGGCCGTATGCAACTCTAATCCTACTCCTTGTAACGATCGTCC
TGAAAAATTGTTTACGGTTCACGGTTTGTGGCCTTCAAACAAGAAGGGACCTGACCCAGAAAAATGCAAGAATATACAA
ATGAATTCTCAGAAGATAGGAAATATGGCAGCCCAGTTGGAAATTATTTGGCCGAATGTGCTCAATCGAACCGATCATG
TAGGCTTCTGGGAAAGAGAGTGGCTCAAACATGGCACCTGCGGGTATCCCACAATAAGGGACGACATGCATTACTTAAA
AACAGTAATCAAAATGTACATAACCCAGAAACAAAACGTCTCTGCAATTCTCTCGAAGGCGATGATTCAACCGAACGGG
CAAAACAGGTCATTGGTGGATATTGAAAATGCCATACGCAGTGGTACCAACAATACGAAACCAAAATTCAAGTGCCAAA

AGAATACTAGGACGACGACTGAATTAGTTGAGGTCACTCTTTGCAGTGATAGAGACTTAACGAAGTTCATAAATTGCCC
CCAACCACAACAAGGATCACGATATCTCTGCCCCGCCGATGTTCAGTAT
>P_avium_S3_RNase_AJ298312
ATGGCTATGTTGAAATCGTCACTCTCTTTCCTTGTTCTTGGTTTTGCTTTCTTCTTGTGTTTCATTATCAGCGCTGGTG
ATGGATCTTATGTCTATTTTCAATTTGTGCAACAATGGCCACCGACCACCTGCAGAGTTCAGAAGAAATGCTCTAAACC
CCGGCCATTACAAAACTTCACCATTCATGGCCTATGGCCAAGTAATTATTCAAACCCAACGATGCCCAGTAATTGCAAT
GGGTCGCGATTTAAGAAAGAGCTATTGTCCCCTCGAATGCAATCCAAACTGAAGATATCTTGGCCGAACGTTGTAAGTA
GCAACGATACAAAATTTTGGGAAAGTGAATGGAACAAACATGGTACTTGTTCCGAACAGACACTTAACCAAGTGCAATA
CTTCGAGATATCCCACGAAATGTGGAACTCGTTCAATATTACAGATATCCTTAAAAACGCTTCAATCGTACCACATCCG
ACACAAACATGGAAGTACTCGGACATAGTATCAGCCATTCAGAGTAAAACTCAAAGAACACCCCTCCTTCGTTGCAAAA
CGGATCCAGCACATCCTAACGCGAATACTCAGTTGTTACATGAAGTGGTATTTTGTTATGGATATAATGCAATAAAGCA
GATTGATTGTAATCGAACAGCAGGATGCAAAAATCAAGTTAACATCTTGTTTCCA
>P_dulcis_SM_RNase_DQ099895
ATGGGGATGTTGAAATCGTCACTCGCTTTCCTTGTTCTTGTTTTTGCTTTCTTCTTTTGTTACGTTATGAGCAGCGGAT
CTTATGACTATTTTCAATTTGTGCAACAATGGCCACCGACTAACTGCAGAGTTCGCGTCAAGCGACCTTGCTCCAATCC
CCGGCCATTACAATATTTCACCACCCATGGCCTATGGCCAAGCAATTATTCAAACCCAAAGATGCCCAGTAATTGCACA
GGGTCGCAATTTAAGAAACAGAATTTGTACCCTTATATGCAATCCAAACTGAAGATATCTTGGCCGGACGTGGAAAGTG
GGAATGATACAAAATTTTGGGAAGGCGAATGGAATAAACATGGTACATGTTCCGAACGAACACTTAACCTAATGCAATA
CTTCCAGCGATCCCACGCAATGTGGAAATCACACAATATTACAGAGATCCTTAAAAACGCTTCAATCGTACCACATCCG
ACAAAAACATGGAAGTACTCGGACATAGAATCACCCATTAAAAGAGCAACTAAAAGAACACCCGTCCTTCGTTGCAAAC
GGGATCCATCACATCCTAACAACTCTCAGTTGTTACATGAAGTGGTATTTTGTTATGATTATAAGGCAAAAAAGCAGAT
TGATTGTAATCGAACAGCAGGATGCTGGAATAATGTTGACATCAAGTTTGAA
>P_avium_S1_RNase_AB028153
ATGGCGATGTTGAAATCGTCACTCGCTTTCCTTGTTCTTGCTTTTGCTTTCTTCTTTTGTTACGTTATGAGCAGTGGAT
CTTATGACTACTTTCAATTTGTGCAACAATGGCCACCGACCAACTGCAGAGTTCGCATCAAGCGACCTTGCTCCAAACC
CCGGCCATTACAAAATTTCACCATCCATGGCCTATGGCCAAGTAATTATTCAAATCCAACGAAGCCCAGTAATTGCAAC
GGGTCAAAATATGAGGACAGGAAAGTGTACCCTAAATTGCGATCCAAACTGAAGAGATCTTGGCCCGACGTGGAAAGTG
GCAATGATACAAGATTTTGGGAAGGCGAATGGAACAAACATGGCAGATGTTCCGAACAGACACTTAACCAAATGCAATA
CTTCGAGATATCTCACGACATGTGGGTGTCGTACAATATTACAGAGATCCTTAAAAACGCTTCAATCGTACCACATCCG
ACACAAAAATGGAGCTACTCCGACATAGTATCACCCATTAAAACAGCAACTAAAAGAACACCCCTCCTTCGTTGCAAAA
CTGATCCAGCAACTAATACTGAGTTGTTACATGAAGTGGTATTTTGTTATGAATATCATGCGTTAAAACAGATTGACTG
TAATCGAACAGCAGGATGCAAAAATCCACAAGCCATCTCGTTTCAA
>P_avium_S5_RNase_AJ298314
ATGGCGATGTTGAAATCGTCACTCGCTTTACTTGTTCTTGCTTTTGCTTTCTTCTTTTGTTATGTTATGAGCAGTGGAT
CTTATGACTATTTTCAATTTGTGCAACAATGGCCACCGACCAACTGCAGAGTTCGAACGAAATGCTCCAACCCCCGGCC
ATTACAATATTTCACCATCCATGGCCTATGGCCAAGTAATTATTCAAACCCAAAGATGCCCAGTAATTGCATTGGGTCG
CAATTTAACGAAAGTAAAGTGTATCCCCGATTGAGATCCAAATTGAGGATATCGTGGCCGGACGTGGAAAGTGGCAATG
ATACAAAGTTTTGGGGAGACGAATGGAACAAACATGGTACATGTTCCCAACGGATCCTTAACCAATTCCAATACTTTGA
GCGATCCCAGCAAATGTGGAGATCTTACAATATTACAAATATCCTTAAAAAAGCTCAAATCGTACCAAATGCGACACAA
ACGTGGAGCTACTCGGACATAGTATCACCCATTAAGACAGCAACTAACAGAACACCTCTCCTTCGTTGCAAATCTCAGC
CTAAGAGCCAAGCGAATTTTCAGTTGTTACATGAAGTGGTACTTTGTTTTGATTATAATGCGCTAGTACATATTGACTG
TAATCGAACAGCAGGATGCTGGAATAACGTTGACATTAAGTTTCAA
>P_dulcis_S7_RNase_AY291118
ATGGGGATGTTGAAATCGTCACTCGCTTTCCTTGTTCTTGGTTTTGCTTTCTTCTTTTGTTACGTTATGAGCAGTGGAT
CTTATGACTATTTTCAATTTGTGCAACAATGGCCACCGACCAACTGCAGAGTTCGCATGAAGCGACCTTGCTCCAATCC
CCGGCCATTACAATATTTCACAATCCATGGCCTATGGCCAAGTAATTTTTCAAACCCAACGAAGCCCAGTAATTGCAAT
GGGACTAAATTTGATGCAAGGAAAGTGTATCCTGAAATGCGATCCGATCTGAAGATATCTTGGCCAGACGTGGAAAGTG
GCAATGATACAAAATTTTGGGAAGACGAATGGAACAAACACGGTACATGTTCTGAACAGACACTTAATCAATTCCAGTA
CTTTGAGCGATCCCACGAAATGTGGATGTCGTACAATATTACAGAGATCCTTAAAAACGCTTCAATCGTACCACATCCG
GCAAAAACATGGACCTATTCGGACATAGTATCACCCATCAAAGCAGCAACTGGAAGAACACCGCTTCTTCGTTGCAAAT
ACGATAACAACACTCAGTTGTTACATGAAGTGGTATTTTGTTATGGCTATAAAGCAATAAAGCAGATTGACTGTAATCG
GCCAGGATGCAAAAATAAAATTGACATCAAGTTTCAA
>P_bretschneideri_S34_RNase_DQ414813
ATGGGGATGACGGGGATGATATATATGGTTACGATGGTATTTTCATTAACTGTATTAGTATTGTGTTCCTCTACGGTGG
GATACGATTATTTTCAATTTACGCAGCAATATCAGCCGGCTGTCTGCAACTCTAGTACTACTCCTTGTAAGGATCCTGC
TGACAAGCTGTTTACGGTTCACGGTTTGTGGCCCTCAAACTGGAATGGATCCCACCCAGTAAATTGCACGAATAAAACC
ATGAATTCCCTGACGATGGGAAATCTGACAGCCCAGTTGGAAATTATTTGGCCGAATGTACTCAATCGAAACGATCATG
CAGGCTTCTGGAATAGACAGTGGAACAAACATGGTACCTGTGGAGTCCCCAAAATAAACGACAGCTTGCAATACTTTCG

AACAGTAATCAAAATGTACATAACTCAGAAACAAAACGTCTCTGAAATCCTCGCAAAGGCGAATATTAAACCGGAGGGG
AAAAACAGGACACTGGTGGATATTCTAAAAGCCATACGCAGTGGTACAAACAATAAGGCACCAAAACTCAAGTGCCAAA
AGAAATCTTCGATGACTGAATTGGTTGAGGTCAGTCTTTGCAGCGATCACAACATAACGCAGTTCATAAATTGCCCCCG
CCCATTCCCACAAGGATCACCACATTTTTGCCCAAACAATAGTATTCAGTAT
>P_avium_PA1_AB096918_Prunus_PA1
ATGTTAAAACTACTCCTTCTTGTGCTCTTTTCAGCGGCGTCTCTGCAAGCTATCACCACTCATGGGCAACCGTATGACT
ATCTACAATATGTACTACAATGGCCGAACACGAAATGCGTGAAGGCACGGTGCATTCCAGGGATTCAAAAAACTGAGTT
CACTACTCACGGCCTCTGGCCAACCAACCTCTCCAAGATATTGACATGCAATTCAGCTTCAAAATTTAGCAGCACTATG
CTGCAAAATGATGCTACATTGGTATCGAAATTGAAAACTTCTTGGCCAAATCTGGAGCAGAGAGTTGCCCAAGGAAAAG
ACAATGATATGTGGTTTTGGGCGATGGAGTACGAAAAACACGGCACATGTGCTAAGTTTTCCAGCCAGAATACTTACTT
GTCAAAAGCATGTGATTTGTGGGAAGAAAACAAGATTAAGGATATTTTTGCCAAACACAAAATCATTCCAAGAAACGCG
ACGTATAAAGATGTTTTGCTTACGAATGCTATTCAAATGGAAACTCGCAGTTCGCCTCTCCTTCTTTGCCATAGAGTCA
ACGGCGGTGATTTGTTGTGGGAGGTTGTACTTTGCTATGACGACACGGCTAAAAAACGGATGAATTGTTCTGATCAAAG
TGCAAGACAAACAAATTGCGGAACGGACATCTATTATAAA
>F_vesca_00230_S_RNase_lineage_1
ATGGAAATGGCATTGCTAGCGGTTTTTCTTATCTCAGCTGCTGCTTGTTCATCAGTCACTGAAGCAGCCAAGGCCTACG
ACTACTTCCAGTTCGTACAACAATATCCTATTACCTTATGCATGTTCGACACAAGTTGCATTCCAGGACAATCACTGCC
TCGATACTTCTACATCCACGGCTTATGGCCCAGTAACTTCACTTATCCGCATGACGATTGTGTGGGTACACTGTTCGAC
TACAATCAGATGAGCAGTGATCCTACTCTCTCAAACAATTTGCTGCAATCATGGCCCAGCTTCACGTCCAAATCCCACA
TCAAATTCTGGGCGGATGAGTATCACAAGCACGGGACCTGTTCGGAGATAGACTACCCGCAGCACCGCTATTTTGCCGA
AGCCCATATGCTCTGGACGTTCGTCAATGCATACCGTATCTTAGCAACATACAACATCGTTCCCGGCAGAACCTACCCT
GCGGTTGACATTATATACTTCGTCCAAGCAGCACTCAAAGGAATCACACCATCTGTTATGTGCAAGGCCAATTCTGCGG
GTCAAGAATATCTCCACGAGATCATCATATGTCTTGACAAAACGCTTACCAGATTCATCAATTGTGTTCGACCATCCAC
TTGCCGTACTACAGCGGTAATGTATCCC
>F_vesca_00227_S_RNase_lineage_1
ATGGGGATCATCCTACTTGTCCTGCTGCTTCTCTCTGCGTGTGGTGCAGATGATGCAGCGAACATCTACGACTACATGC
AGTTCGTCCAGCAATGGCCGGCGACATTCTGCTATGGTAATCCGAATTGTGTCCCAAATCCACCACACAGTACTTTTAC
GATTCACGGCCTCTGGCCCAGCAATTACTCGACACCTGAATTTCCATGCGTTGGCACACCCTTTGATCCCTCGCAGATG
TTCGCTGCCGAAAACCGTGGTTTGAGGGACTATTTTCTGCCGCAAGCATGGCCACAGTTGAATGCTCGTTATACCAACT
TGGAGTTCTGGAAATTCGAGTACGAAAAGCACGGTATGTGCTCGGAGAACTATCTGAATCAGATGGATTATTTCAGGAA
AGCTTACTGGGCGTGGGCTCGATTCAATGCTTATGTTCTGTTTGCGGCATCGCCTAAGCAAATCTACCCTGGCAATTAC
TATTACACAAGAGATATTGAAGCAGCCATTCAGAGAGTGACCAGTGAGAAGCCTCTGCTTATGTGCAAGAAGGTAAGAG
TAGGTACGGTTGATAGTTGTCTATTGCAGGAGGTCATCATCTGTCTTGATCGTTGGGCAGACAACGTTGTACCTTGTCG
CCTTCGACTGTCATCGTGCAACGTCCCAATTATATATTATGGTTAC
>F_vesca_26822
ATGCATGAGTGTGTACGTATCTTAATTTTCATATATGGCAATCTCATTTCATATTTGGCAATCTCATTAATTGATGAAA
ACACAATCGACTATACAAAGCCGATCATTTCACAAGGAACACCCTTCCAGTACTACCAACTGGTCATGCGATGGCCAAA
AGCAGCATGCTACCACAAACCTGCTGAGCCAAAGATTTGTGTTATGGATGATGATCATGTTCCGATGAGGTTCACCCTC
CACGGAATGTGGGCGACGAACTGGACAAATATCAAAGATGAAATTCGGTGTGAAAAGGCTGGAACGCCATTTGATGAAG
ATGAGATGACTAAACAAAAGACACTGCAAGAACAACTGGAGCAAAGCTGGCCGACGTTACTGGATAATTATCCAACTAA
TATGGAATTCTGGAAGCACGAATACGATAAGCATGGCCGATGCAGCCAAGATACAACTACTCAGACCGAATACTTCGAA
AAAGCTCACAAATTGTGGCAGGAATTGCAGGTTGTGTACCAAGCACTTGCAGATGACAAGATCATACCTGCAGCTGATA
AGATTCACACCTACAACCAGATCGAGCGAGCAATCGCTAAACGATTCGGCAGTAGTTGGAAGGTTGTGCTTTTTTGCAG
AAAGGTGAATCTGACGCCTCGTGGTACCGGTGGAACAAAAACGATTCTACTTCTTTTGCATGAAATCGTATTCTGCTTT
GATAACAAAGCAATGGAAAAGAAGAGTTGCAACAGAAAATCGAACTGCGAGAACGCGAAAACTATAACACTGATAAAT
>F_vesca_22609
ATGATGAAGAATTCAGTCTTCGTTTTCTTTCTGTCAGTCTATGTCCTTGTCCAACTTATCAAGCTTTCAAATGCTGAAC
CATTTGAGTACATGCAGTTTGTGCTCCAGTACCCACTTGGAGTTTGTTATGGTACACAAAAAGGTGATTCCAAATCACC
TCTGCCTACCAAGTTTACCGTTCACAGCATCTGGCCGTCAAACTTCTCCGATACACGAGTCGTTTGTAGGCAAGCACTG
ATAAGTCACCCCTTTAATAATGCGCAGATGACGTCGTCTCTGCAGACTGATTTGACAAACTCATGGCCATCTGTGATAA
CAAAAAAAAGCGACATGCAGTTTTGGCAACATGAGTATGAGGAGCATGGCGCTTGTTCAGTAGACAAGGGTGTACCACT
GTTCACCCAGAAGTCCTACTCTGAGAGAGGAAACCAGCTCTGGAACCAATATGACATCCATGCACTGCTTGATCAAAGC
AATATCAAACCGGATTCTTCAAAGCCATGCAAAATGACTGAGATTGTAGCTGCAATCCAAAAGAAAATCGGGAAAAACA
CACTCCTGTACGAAGGTGCAAGGAAGATAACAAAATTTACATAC
>F_vesca_12961
ATGTTGTCATTTCTCATTGTTTTGCTTAGTGTTCAAATGCTGCTAGCAGCAAATCCACCTCCACCTCCACCTCCAGCTG
TTCCAGCTCTTGATTCATACAAGCTGGTCATTGAATGGCCAAACACTTTTTGTCTTGTTGAGCCACAGCCACCATGCCA
ACAGCATCCACAGTCTTTTACATTGCATGGCCTTTGGCCACAGGCGGGTGGTCGATCGTTAGTGAACTGTCCTGGTTCA

CCAATGGAAGATCAAACCCTAGAGGCAAATAAAGGTGATCTGACAAAGTTTTGGCCTGATCTTAGGAACTCGGACTTTG
ATAAGAGTAAAAGCTTTTGGCGTCAGGAGTGGGACACACACGGTCGCTGTTCTGGCAAGCCTCCGGCCGACTACCTTAC
CATGGTCTTTAATGCAGTAAAAAAGTATGACGTGCAAAAGTTACTTGCAAAGAATGGAATTTTACCAGGTGATCCGACA
GGGCATACGGCGGCACAGTTTGAACAGGCAATATTCAAAGAAACTAAACTGCACACAGAGATTGAATGCCGGACGAAGG
ACTCGAAGGAGTATCTATTTCAAATTTATTTCTGCTTAACACCTCAAGGACAGTTCAAGAATTGTCCGATCCGTTACAG
GTGTGCAAATCAACAAATCTACATGCCCTTGCCTCCGCCCAGTCCC
>F_vesca_scf0513144_1_Prunus_S_RNase_lineage
ATGCATGGTATGTTTCTATATATTGTTGACGTCGGTTTGCCGAAGGAAAGGTCTATCTATATAACAAGCATGAGATTCG
TGACTGTGTTATTGTTGTTGTTTCTTGTTTCTAGCACTATAATGTCTCGAGGATCTTATGATCATTTCAAATTTGTACA
ACAATGGCCACCGGCCTTCTGTGCCACGAATCGCTGTAAACCAGGGGCAAAACAGATGTTAATGTTCACTATCCATGGT
CTCTGGCCAGATAATAAAACTTATGTGCCTTGTCGTGCCCTTTTACCGTTGTTTGCAGGCCGTCAGATCCTTAATGATG
CGAGTCTGGTAAACAAGCTGTTGACCTCTCCAAATCTGAAAGGAATTGATCGCAAGTTTTGGAAAGAAGAATGGGACAA
ACACGGAAGATGTTCCTCGCAGACGTACGACCAAAGACAATATTTCGAGCGGTCACACAACATGTGGATGGAAACGGAC
TTGACCAACACCTTTGGCAACCTTATCAAAGGAAACAGTCTACAACGGAACGTCGCAGATATAGAAAACCTGATTCAAA
AGGTAACTGCAACGGAAAAAAAACCACTCCTCCGTTGTAAAAACAAAAGATCCACACCTCACGATGAAGTATTAACTGA
AATTATAATTTGCTATGCACACAATGGAACTAGTCTGATCGATTGTGTCGGTCCATCCACCTGCACGGACGGATTTTAC
TTTCCT
>F_vesca_scf0513063_1_Prunus_PA1_lineage
ATGGGTTTTGTTTTTTTTCTGGTGGTTGTGCCGCTTACCTCCAAAGCACTTTCGGGTGGTCATCGCCGTCAACATATCA
ATGCATACGGCGATGATCCCGGCTATGATCTGGTGTTATTTGTCCTACAAGTCACTAATCCTCGTGACAAGGAGAATGG
ACCTTACTTAACCATCTTCGGCCTTTGGGCCGAATATCTTCAGAATGGGGCCCCCCCTGAGACCGGGTACCTTCAACCG
TACCTAAGAAAGAGGCTCCATGTAATATGGCCAACCAGAAGAGACGGAAGCTACCCAACAGACCGGGCCTTTTGGCTGC
ACGAAGTTGATAAACATGGGACCTGTATGGCAGATATATTCGGACGGGGGGAGCAGTACCTACAAAAGGCAAAAGAATT
GTATGAGCAGATCGGGATAGGCCAGTTCTTGGGTCGTAACGGAAGGTGGCTGCCAAAAATTGAATTCAATACCGCGGAT
TTACTGGGGGCGATCGGTACTGAACGCAGACCACTATTAGAATGTGAAGGAAATCATCTCGTCAGAATTTTATTCTGCT
ACAGCAAACACTGGGTGATTAAACCCTGCTTGCCCACCCACCCCGATCCAGCAAGAGATTGTCCCGATAGGATTTCTTA
TGTTCTGCCGCAGCAGCAG
>F_vesca_scf0513159_1_Prunus_PA1_lineage
ATGGAAGCACTTGTGATATTATCATTTGTCCTTCTATCTATTTTTCCTCAACTGAGTGCGCAGCTAGGAGAACCGTATT
ACCAATACATACAACTCTCACTTCAAGTCCCCAAGTCGAGGGACCCTCAATCTACTTTGGAGTATTTCCCTTTGCACGG
CTTTCGGCCGGTAAACACATCTCTATCTAACCCGAATTTGCCTTTGGGTTTTAACCGGTATACACCTATCGAAAGGTAC
CTGAGAACTGAACTCAAGTTATGTTGGATGAGTATAGGAGTACCAGGCCAACGCACGATGACGGATGAGCGATTTTGGC
TATATGAATACAGTAAACATGGGCAATGTACTATTAACGAATATCCGACCCCTCAAGATTACTTCGCCATGGCAGTTAA
CCTGTGGCATCTCTATCCTGTGGATAAGTGGTTTAAGAATAATGGATTGCAGCCAAAAAAAGATAAACCGCTCCAAGAT
TTTGTTGCTGTCATCTCAAAAGAATTCGGCGCTATTCCATGGCTAACGTGTTACGACAACGGGAAGAAGCTGAAGGAGG
TGGGCTTGTGTTTCTTAAAATCATCCAGTGTGAATGCGAATCCTGCCCCTACAAGTTGTCCTATAAGGGCACTAACGTA
TGATCTAAGATGTAATTATTTGATACAATATGATCTAGGTCCA
>F_nipponica_gi561674690_gi561985884_gi561957436_Prunus_S_RNase_lineage
ATGCATGGGATGTTTCTATATATTGTTGACGTCGGTTTGCCGAAGGAAAGGTCTATCTATATAACAAGCATGAGATTCG
TGACTGTGTTATTGTTGTTGTTTCTTGTTTCTAGCACTATAATGTCTCGAGGATCTTATGATCATTTCAAATTTGTACA
ACAATGGCCACCGGCCTTCTGTGCCATGAATCGCTGTAGACCAGGGGCAAAACAGATGTTGATGTTCACTATCCATGGT
CTCTGGCCAGATAATAAAACTTATCTGCCTTGTCGTGCCCTTTCACCGTTGTTTAACGGGCAGATCCTTGATGCGAGTC
TGGTAAACAAGCTGTCGACCTCTTGGCCAAATCTGAAAGGAATTGATGCCAATTTTTGGGAAGAAGAATGGGACAAACA
CGGAAGATGTTCCTCGCAGACGTACGACCAAAGACAATATTTCGAGCGGTCACACAACATGTGGATGGAAACGGACTTG
ACCAACACCTTTGGCAACCTTATCAAAGGAAACAGTCTACAACGGAACGTCAGAGATATAGAAAACCTGATTCAAAAAG
TAACTGCAACGGAAAAAAAACCACTCCTCCGTTGTAAAAAAAAAGCATCCACGCCTCACGATGAATTTTTAACTGAAAT
TATAATTTGCTATGCACACAATGGAACTAGTCTGATCAATTGTGCCAGTCCATCCAACTGCACGGACGGATTTTACTTT
CCT
>F_niponica_gi561805796_Prunus_S_RNase_lineage
CTCTTTTCCACAGGTTACTACGACTACTTTCAGTTGGTGCAACAATGGCCACCCAACGTCTGCTTTAAAAGAAAGTCAC
CTTGCTACAATAACACGCCATCACTAATCTTTGGTATTCATGGCCTATGGCCAAGTAATAATACAGTGAATAAAAACCC
CAGTTGCCCACCGAACCCATTTAAACCGCAGGTATATATGATCTTTTGCAGGGTAAGCATGTTAAATAATCAAACGCTG
CTAGCGGACCTTAGCCGTTCTTGGCCCGACGTGGTAAATGGGGACGATCCAACGTTTTGGGCCAAGGAGTGGGACAAAC
ATGGCACATGTTCAGACCAGACATTCGGGCAAAAACGATACTTCCAGCGATCCCACGAGATTTGGGAACAATATAATAT
TACGGATATCTTTAAAAAAGCTGGCTTGATATCGATGACGGGGCCAGCAGTACCAAGACGATGCAACGTCGCAAACATA
CTAACACGCATTGAAGCGGTGACTGGGAAGAAACCTGTCCTTTATTGTCAATGGAAATCGATATACCAGCCGCAGCCGC
AAGGACAACCACCAAAGAGGCTAACCATTCGATTGATTCAGGAAGTGAGCCTTTGTTTTGATTATTATGGAACGACATT
AACTGATTGTACCAATATCAATACACTATGCAAAGGACGTAATGTCTGGTTT

>F_nipponica_gi561877040_S_RNase_lineage_1
ATGGGGATCATCCAACTTACTGTCCTGCTGCTTCTCTCTGCGTGTGGTGCAGCTGATGCGGCGAACCTCTACGATTACA
TGCAGTTCGTCCAGCAATGGCCGGCGACATTTTGCTATGCTAACCCGGATTGTATCCCAAATCCACCGCACAGTACTTT
TACGATTCACGGCCTTTGGCCTAGCAATTACTCGACTTTTGAAGGGAAATGCGTTGGCACACCCTTTGATCCATCGCCG
GTATTGCATTATTGCATGCATGGAATATTATATCACTTCTATTCATTTAGTCTTTCATTTTCAGAATTCTTTCTGCCGC
TAGCATGGCCTCAGTTGGACGCTCATTATACCGACATGGAGTTCTGGAAATACGAGTACAACAAGCACGGTACGTGCTC
GGAGAACTATCTGAATCAGATGGATTATTTCAAGAAAGCTCACTGGGCGTGGGTTCGGTTCAATGCTTATAATCTGTTT
GCAGCCTCGCCTATGCAAATCTACCCTGGTAATTACTACTATACAAGAGATATTGAAGCAGCCATTGTGAGAGTGACCA
ATGTGAAGCCTCTGCTTATGTGCAAGAAGGTGAGAGTGGGTAAGGTTGATAGTTGGCTATTGCAGGAGGTCATCATCTG
TCTTGATCGTTGGGCAGACAACGTTGTACCTTGTGGCCCTCGATGGTCATCGTGCAACGTTCCAATTATATATTATGGT
AAC
>F_nipponica_gi561793890
ATGCATGAGTGTGTACGTATCTTAATTTTCATATATGGCAATCTCATTATTGATGAAAACACAATCGACTATACAAAGC
CGATCATTTCACAAGGAACACCCTTCCAGTACTACCAACTGGTCATGCGATGGCCAAAAGCAGCATGCTACCACAAACC
TGCTGAGCCAAAGATTTGTGTTATGGATGATGATCATGTTCCGATGAGGTTCACCCTCCACGGAATGTGGGCGACGAAC
TGGACAAATGTCAAAGATGAAATTCAGTGTGATAAGGCTGGAACGCCATTTGATGAAGATGAGATGACTAAACAAAAGA
CACCGCAAGAACAACTGCAGCAAAGCTGGCCGACGTTACTGGATAATTATCCAACTAATATGAAGTTTTGGGAGCACGA
GTATGATAAACATGGACGATGCAGCCAAGATACAACCACTCAGACCGAATACTTCGAAAAAGCTCACAAATTGTGGCAG
GAATTGCAGGTTGTGTACCAAGCACTTGCAGATGACAAGATCATACCTGCAGCTGATAAGACTCACACCTACAACCAGA
TCGAGCAAGCAATCGCAAAACGATTCGGCAGTAGTTGGAAGGTTGTGATTTTTTGCAGAAAGGTGAATCTGACGTCTCG
TGGTACCGGTGGAAAAAAAACGATTCTACTTCTTTTGCATGAAATCGTATTCTGCTTTGATAACAAAGCAATGGACAAG
AAGAGTTGCAACAGAAAATCG
>F_nipponica_gi561844698
ATGATGAAGAATTCAGTCTTGGTTATCTTTCTGTCAGTATATGTCCTTGTCCAACTTATCAAGCTTTCAAATGCTGAAC
CATTTGAGTACATGCAGTTTGTGCTCCAGTACCCCCTTGGAGTTTGTTATGGTACACAAAAAGGTGATTGCAAATCACC
TCTGCCTACCAAGTTTTCCGTTCACGGCATCTGGCCGTCAAACTTCTCCGATACACGAGTCGTTTGTAGGCAAGCACTG
ATAAGTCACCCCTTTGATAATGCGCAGACGACGTCGTCTCTGAAGACTGATTTGACAAACTGGCCATCTGTGATAACAA
AAAATAGCGACATGCAGTTTTGGCAACATGAGTATGAGGAGCATGGCGCTTGTTCAGTAGACAAGGGTGTACCACCGTT
CACCCAGAAGTCCTACTTTGAGAGAGGAAACCAGCTCTGGAACATGCAATATGACATCCATGCACTGCTTGATCAAAGC
AACATCAAACCAGATTCTTCAAAGCCA

Supplementary File 2
>Rmultiflora_4
TCACAATTAATTTCCACAGGGTCCTATGACTATTTCAAATTTGTTGTACAATGGCCACCAGCCCACTGCAGTGTTTTTA
AATGCCACAATAGTAACCCGCCACACATTTACACCATCCATGGCCTATGGCCAAATAATCGTTCTAAGGCAGTCGGGAA
TAAGTGCCAGGGATCGTCATTTCAAAAGCCGGCTCCTCCATTGGAAGCCAAACTGAAGATCTCTTGGCCTAACCTAGAA
AATTCGAGTGATGCGCACTTTTGGGAACTTCAGTGGAACAAACATGGCAAGTGTTCGGAGCCGACATTTACGCAATCCC
AATACTTCGATCGAGCCCATCAAATTTGGATGATGGAGAATATTAATATTACTAGTATCCTCCAAAATGTAAACATCGT
ATCAGGGAAAACAAAAAGCTACGAAGAGATAGAATTTCCCATTGAATCAAAAACTCACAAGACACCACTCCTTCACTGC
ATAAACACTCAGAAGTTGCATGAAGTGGTACTTTGCTGGGACCATGCTGCAAAAAAATTGATCGACTGTAAACATACAG
AAGCAACATGCTCAAGGAATAGTCCAATTGAGATTCTA
>Rmultiflora_8
CAATACTCATTTGTTTTTGTGTCAAGGCCTCATTTTGGAATTTTAATGTTCGTTTGTTTCTTGTCACAATTAATTTCCA
CAGGATCGTATAAATATTTCAAATTTGTTGTACAATGGCCACCAGCCCACTGCAGTGTTTTTAAATGCCACAATAGCAA
CCCGCCAAAGATATACACCATCCATGGCCTATGGCCAAGTAATCGTTCTAATGCAGCTCGCAGTAAGTGCCAGGGATCG
TCATTTCAACAACCGGCTCTTCCATTGGAAGCCAAACTGAAGATCTCTTGGCCTAACCTAGAAAATTTGAGTGATGCGC
AGTTTTGGGAACTTGAATGGAACAGACATGGCAAGTGTTCGGAGCCGACATTTACGCAAACCCAATACTTCAATCGAGC
CCATGAAATTTGGATGATGGATGACATTAATATTACTGATATCCTCCAAAATGTTCACATCGTATCAGGGAAACAAAAA
ACCTACGCAGAGATAGAATTTCCCATTGAATCAAAAACTCAAAAGACAATCCTCCTTCGCTGCCTAAACCCTCAGAAGT
TGCATGAAGTGGTAATTTGCTGGGACCATGCTGCAAAAAATATGATCGACTGTAATCGTACAGAAGCAACATGCTCAAA
CAGCAGTCTAATTGATGTTCTA
>Rchinensis1_3_Rchinensis2_27
ATGGCAATGTTGAAATCATCGTTCGCTTTAATTGCTCTTGCTTTTGCTCTCTGTTTCACTATGAGCATTGGTTCCTATG
AATATTTTAAATTTGTGGTACAATGGCCACCAGCCCACTGCAGGGTTAGAAAATGCACACCCCAAGCCTTGCAACAGAA
AATTTACACCATCCATGGCCTATGGCCGAGTAATTATTCTAAAGCTGTTGTGTATAATTGTCCGGGATCGTCTTTTCAA
AATCCGGCTCCTCCATTGGAAGCCAAACTGAAGATCTCTTGGCCCAACCTAGAACGTCCGAACGATGCAATGTTTTGGG
CACTTGAGTGGGACAGGCATGGCAAGTGTTCGGAGCAGACATTTACACAAACCCAATACTTCAATCGAGCCCATGAAAT
TTGGGTGGGGAAGAATATTACTGACATCCTCCAGAGAGCTAGCATCTTATCGGGGAGACAAAAAGACTACGGAGTTATA

GAATTAGCCGTACGATCAAAAACTCAAAAGACACCCCTCCTTCGCTGCGAACAACCAAAGCAGAAACCTACGCAGAAAC
CTACGCAACCTACGCAGCGGACTCAGTGGTTGCATGAGGTGGTACTTTGCTGGGACTATCATGCCAAAAATATGATTGA
CTGTGATGATACAGAAGCAACATGTCAAGATACTTTTCCAATCGATATTCTG
>Rchinensis2_16_Rchinensis3_17_Rchinensis4_40
ATGTCTACAGGGTATTACGAATATTTCAAACTTGTGCAACAATGGCCACCAACCACTTGCCAAAATGCAAACTGCCGCA
GAGTACCGCCACCTCGCCTCTTTACCCTCCATGGGTTTTGGCCATCTAATTATTCAAACAATGTCGTGGCTAATTGCAC
TAACGCAATATTTCAACGGATGGATCGCTCATTGGTATCTAAACTGAATATATCCTGGCCCAATGTGAAATATCCGAAT
AATATCCAGTTTTGGGATAAACAGTGGAGAAAACATGGCTCATGTTCCGTGCACACGTTTAACCAAACAGAGTACTTCA
CGCAAGCGGATAATCTTTGGAACTCACACAACATTACTGATATTCTTATAACTGGTGGGATCAAGCCAAATGGATCAGA
ATATGCATACGATACGGTAGAGCGGCCCATCCAAATTGCAACCGGGAAGGAACCCGAACTTCGTTGCGCACCATCCTCC
CTTGGAAGGCAATTGTTGCATGAAGTAGTACTTTGTTATAATCATAAGGGAACAACGCCCATCGACTGTAACCCTTTAC
ATTCAACCTGCGATCGTAACTTCCAAATAAAGTTTATT
>Rchinensis3_9_Rchinensis4_28_SRNase26
ATGGGGATTAATGTCATCCTGATTCTTCTCTGCCTTATCAGCAACGTTGCTAGTGCAGCCAACACATACGATTATCTTC
AACTAGTACTGCAATGGCCAAACACGTTCTGCATCAGTAACCAAAAGTGCATATCAAATCTCCCACTAGACTTCACCAT
ACATGGGTTATGGCCTAGCAATTTCTCCGGCCAAAATAACCCATGTGTGGGTGCGCCATTTAATAGAGCTCAGATGTCC
GCGAACTACCGTCTTCAAACCATATTGCTCCCATCCTCGTGGAGAAGCTACCTTATCGCTGTCTCCAACACAAACTTCT
GGCAGACTGAGTATAACAAACATGGTACATGTTCGGAGCTCAATTTGCCACAGACGGAATACTTCACCAAAACCCATTG
GCTATGGATGGTCAACAACATTTATAGTATATTTTCGTCTTCGGTAAACAGACAAGGATATCCAATCTTGCCGGGTTTC
ACCTATGACTACGCCGATCTTCTTGCCGCAGTTCAACTAGGAATCGGTGGCGTCACTCCCGTGCTTACTTGCATGGTCA
TAAATAACAACCAATACGTTCTTCACGAAGTGGTTATTTGTTATGATGCCTTGGGGATCAACCGCATCCATTGTGTGCG
ACAATCGAGTTGCTCTTTCGCTCCTAACGGACGTGTGCTCTATCCT
>Rchinensis3_6_Rchinensis4_44_2_SRNase30
ATGGAAACGGCATTGCTATGTACAGTCTCCATGCTGGTTATTTCTGCTTTTCCATCCGGTGCTAAAGCCGCGAATCAGT
ACGACTATCTTCAATTTGTTCAACAATGGCCGATAACCTTGTGCTACAACAACCCAGCCTGTATTCCAGGAGCATCACT
CCCACTGGACTTCTTTATCCATGGGTTATGGCCAAGCAATTTCTCTGGCCAGAATCAGCCCTGTGTTGGTACTCCGTTC
GACTATAATGAGATGCTTAGTCAATATCGTCTCCGAACCAAAATGCTACCATTCTCATGGCCAAGCTTCACTCGTAGAT
CCAACATGGGCTTCTGGAAATATGAGTATAATCAACACGGCACTTGTTCGGAGAATAACCTCGCGCAGACGGACTATTT
TTACAAAGCCTATGCTCTCTGGATGCGCTACAATGCAAACTTTATATTATATGCATCTACAGTTATACCAGGCCACAAG
ATTGTGCCGGGATACCTCTACCATTACGCTGACCTTCTAAACTCCGTTCAACAAGCAATTGGTGGATTCACTCCTTCGC
TTATGTGCAAGCACGAGCCTGCAAATAATACTTGGATTCTGCACGAAGTCATCATATGTTTTGATGCCATGGGGAACAA
CGTCATCAATTGTGTTAGAGGATCTAGTTGCTCTGCCACTACCACCGGGTATATACACTATCCTTTGCAA
>Rchinensis3_10_Rchinensis4_44_SRNase36
ATGGCACAAAATAATATGGCTTACGTCCTCATGACCTGCACTACTGTTGCCTTACTCTTTACTCTGGCCTCCTCGTATA
CCGCCTACGAATACTTCTTGTTTGTTCAACAGTGGTCCAAGACCGTTTGTGGTAACAGCTGCAAAGCTCCCTCTCCAGT
TTTTACGATACATGGGTTGTGGCCCTCCAACCACACTGGCCCTCAGCTGAAGTGCACTGGTGCAGCATATAATCCCACA
GAGATGAACAACCAACCCATCTTGAAAAAGAACCTTGAAACTAAATCATGGCCAGATGTTATTCATGGGCAGCATGAGA
TTTTCTGGATACACGAATGGGGCTTCCACGGCAAATGTTCAGATCTCGTCTTCCCACAGACGAAGTATTTTGAGCGCAG
CCATGAAATGTGGACCGATATGGTCATAGGAGATATGCTTAAGAAGGCGTCCATCGTCCCCGCGGCCGGAGCAAAGTAT
AGCCTTCCCGACATTGAAAAGGCCATCAAAGCCAAGACTTCGCACGACCCATTAATCCGCTGCTTCAAGAATCAGTTGA
AGGAAGTAGCATTATGTTACGATTACTATGGCAAAAACGTGGTTGACTGTGATAAATCGCAAAGCAATTGCCCTCAAGC
TCCAACTCTTATAGAATATCCGAAC
>Rrugosa14_8
TGGCCTGGAGCATATTGCGACACAAAGCATAGTTGTTGCTATCCAAAGTCAGGGAAGCCTGCAGCAGATTTCGGCATTC
ACGGTCTCTGGCCAAACTACAAGGATGGCTCTTACCCTTCAAACTGTGATCCAGACAGTGTCTTCGACAAATCTGAGGT
ACACGACACCTTTACTTTAATCAACATATATATAACCTTAATATCAGAGTTGATGGGCAATCTGGAAAAGAGTTGGCCA
TCTATGAGCTGCCCAAGCAGTAATGGTTACAGGTTCTGGTCACACGAATGGGAGAAGCATGGCACTTGCTCCGAATCTG
AACTTGATCAGAAAGATTACTTCCAAGCCGGTCTCAAGCTCAAGGAAAAAGCAAACCTTCTTCAAGCCCTTAAAAAAGC
TGGTAATTTAGTTCGTTTATACTCAATTGTTTATAAGAAATTAACCAAGCTAGAT
>Rrugosa14_9
ATGGCATTCCTAGCAGTTAGGACACTAATTGTTCTTATGCTTATTTCTACATTTGCTAATGCCGCAAACAAATATGACT
ATCTACAACTAGTGCAACAATGGCCTAAAACGTTCTGCCATAATAACCAAGCTTGCATTCAAGGTGCAGCCCTCCCGGA
GCTGTTCTCGATACATGGTATGTGGCCATCTAATTTCTCCGGCCAGAACGACGCTTGTGTTGGAACTCGGTTCAGCATG
AGAGAGATGCGTCGACACAATCATCTCGAAACTCAATTGCTGTCATCCTCATGGCCAAGCTACACTGGTCTGTCCAACC
TGCACTTTTGGGAGTACGAGTATAACAAGCACGGCACTTGTTCAGAGAATAACCTCAAACAGACGGACTATTTCACCAG
AGCCGACGCTTTGTGGAGGCGCTACAATGTTACCAATATGCTTTTAACATCGCGCCACCAAATCTCGCCAGGATCCTCC
TATCGT
>Rrugosa14_13

ATGTCTCTAGGTTCATATGATCATTTTAAATTTGTACAACAATGGCCACCGGCCGTCTGTGACACGTCTGGCTGCAACA
GATCGGGGTTATCACGCTTCACGATCCATGGCCTTTGGCCAAATAATAAAACGTATGTGAAAAATCAACCGACTTGTCT
TACCAATCAGACCAACTCATTTAAGGCTGCGATACTCACTAATTCGAATTTGGTAAGCAAGCTGAGCACCTCTTGGCCC
GATGTGAAAAATGCAAATGATAATTTTTTTTGGAAGAAACAATGGGACAAGCATGGCACATGTTCCTTGCAGACATACG
ACCAAGCACAATACTTCGAGCGGTCATACAACATGTGGAAAGAAACCAACCTGACAAACACCTTGGATAGCCTTATCAA
ACAGACACCGAGACAACAGAACGTCACAGATATAGAACAACTCATTCAAGGAGTAACTGCAACTGAAAAGAAACCCCTC
CTCCGTTGTGAACAATCTCATAGTAATGCCAGTAAGTCTCTCTTGAAAGAGATTGTCATTTGCTATGCACACAACGGGA
TTACTGTGATCGATTGCGTCAGTCAACCAAAATCCACCTTCGAATGCTATGACGGATTTTGGTTTCCT
>Rrugosa14_18
CCCAGTGTGGCTTTTGTCGTCACTGCTCTAGCTTTGGTCTTCAGTCTGACGTCATCATATACACCCTACCAATACCTGT
TGTTTGTTCAACGGTGGCCAAAGCCTGTTTGTTATGATATCAACTGCCCTAACACTCCCCCTCCAATCATCACAGTACA
CGGTCTATGGCCATCGAACTATAATGGACCTCGCTTGAAGTGCTCAGGCGTTATGAGTCACCAAACAGTACTAAAAGCA
AACTTGGAACAACGTTCTTGGTCGGATGTGGAAAAGGGAAACCATATGGGTTTCTGGAAGCATGAGCAGCAGGCTCATG
GACAGTGTTTAGACACCGTCTTTCCAGTTTCCACATACCAAATATTTCCAATGGTGTCACGATATGTGGAAGAA
>Rrugosa14_19
CCCAGTGTGGCTTTTGTCGTCACTGCTCTAGCTTTGGTCTTCAGTCTGACGTCATCATATACACCCTACCAATACCTGT
TGTTTGTTCAACGGTGGCCAAAGCCTGTTTGTTATGATATCAACTGCCCTAACACTCCCCCTCCAATCATCACAGTACA
CGGTCTATGGCCATCGAACTATAATGGACCTCGCTTGAAGTGCTCAGGCATGAGTCACCAAACAGTACTAAAAGCAAAC
TTGGAACAACGTTCTTGGTCGGATGTGGAAAAGGGAAACCATATGGGTTTCTGGAAGCATGAGCAGCAGGCTCATGGAC
AGTGTTTAGACACCGTCTTTCCAGTTTCCACATACCAAATATTTCCAATGGTGTCACGATATGTGGAAGAAAAACGTGA
TAGGCGAGATCCT
>Rrugosa14_28
TTTGTGCAACAATGGCCACCAACTTTTTGCAAAATTAAAAACTGCGTCCCAAACACACCCAGAAACTTCACCATCCATG
GTTTGTGGCCAAGTAATGCTTCATACAGTTCGGCGGGTTTGTGTCCTGGAGCGGTATTTAACTTCAATTTGGCAGGTGC
GTTAGCACCTCTATTACAAAACTCTTGGCCAGACCTGGAAAATGGTAACGCCACGAGATTTTGGAAACTTGAGTGGGAT
AGACATGGCAAATGTTCCGACCAGACATTAGACCAAAGAAGTTACTTCACACAATCCCACAACATTTGGACTAGATATC
ACATTCTTGATATCCTTAAAAACTCTCAAATAGAGCCAGATGGTAGTTTGTACTCTTACACATCAATAGAATCCTCCAT
CCGA
>Rrugosa14_35_Rrugosa19_36
CCGGCCGGACAGCCGTATTATGACTACCTGCAATTTGTACTGCAGTATCCCAGAACAGTGAATCCAATATCAAAATTAG
ACAAGGTTTTCACCATTCATGGCCTATGGCCTAGCAATTACAGCGGATGTCCTCTGAACAAAAGCGGCCTTCGAGATGA
ACTCCTTCACTCTTGGCCCAATGTAGAGACTCCAAACAATTCGGACTGGTTTTGGAGCTACGAATACAATAAGCACGGC
ACCTGCACCAAACTCATATTCACGGAGCCGCAGGCTTACTTTAAAAAAGCAGATGACTTGTATCGCACACCACCTTTGG
ATAATTTATTCACCCGCATCAGCATCCCCGCGGGAGAGCTAGTCGACACGCGAACATTTAAGAACCGCGTAAAAAAGGC
GATTGGAGGGATTCCACGACTGAGGTGCAGTCAAACCAATGGAACCCTACTGCAGGAAGTAGTTATTTGTTACAATAAA
ACTGCAACTAATGTGACTGATTGTCGAAAAAAAAAAACC
>Rrugosa14_39
AATGTGGAGCACTTGAGATTGGCCCTGACCAATCCAAATGCTTATTGCTCATTAAGCGCTTGTGTTGCACCTATTCCAC
AGTCTTTTACTCTTCACGGACTATGGGATCAGAATTTTGTCAAGATTAGTGGAATAACCCCCTTGAAGTCAATTCATCT
AAGAGGAGTTTCAACTCAAGATCTTCTCACCTATTGGCCAGACCTGTCTGCAGATCGAGCAATCCATGGGAGAATCAAC
TGCTTTTCATTTTGGAGGCATGAATGGGGATCCCATGGTGCACATATA
>Rrugosa14_40
CCAGATAGTTTCTTGATTGAGATCGATTTGCTAAAACCCTCTCTTCACCACCTCATCACCACCATGAAGTCGATTTTGA
TTTCATTTGTCACAATTTTTATCAGTTTTCAACTTCTCCTAGCAGCGCCAAATCCACCTCAAGCCCCACCTCCAGCGCC
CACTCCACCTCCTGATTCCTTCATTTTGGTCATTGTGTGGCCCAATACCTTCTGTCTTTTTGAGTCATGTCAGCTGCAT
CCACAATCTTTTACACTACATGGATTGTGGCCACAGGCGAATGGGAAATCCTTGAAAGATGATTTGAACAAGTATTGGC
CAGACTTGACGCATTCCAAGTTTGAAGAGAGTAAAAGCTTTTGGATACATGAGTGGGAAAAACATGGTCGTTGCTCTGC
AAAGAGTCCTGCTAATTACTTGAGCCTGGTTTTTGATCTGATGAAAAAACATGATGTGGAACAGATATTTAAAAACAAT
GGTAAGAAA
>Rrugosa19_15
CATGCAATTTGTACTCCAGTATACCCACCTGGAGTTTGCTATGACACATCAAAAGGTTATTGCATCTCACCTCTGCCAA
CCAAGTTCCATGTACATGGAATATGGCCTTCTAACTTCTCCGATATACATGTCACTTGTGGTCAAGCACTGAAGAATAA
CCCCTTTGATAACGCGCAGGTGTACGGATTTGGTAAACTCATGGCCTCCGTGTTAACAACAAAGAGCAATATGTGGTTC
TGGGAGCATGAGTACAAGAAACATGGCGCATGTACAGTAGAGTCAGGTGTACCACCTTTCACTCAAAAGTCCTACTTTG
AGAAAGGACACCAGCTATGGAACCAA
>Rrugosa19_31
ATGAAGTCGATGATGTCTTCATTTCTCACCATTTTTCTCTGTTTTCAACTGCTCCTAGCGGTGCCAAATCCACTTCGAG
AGAAAATTTCAGCTCCTGATTCCTTCCTGTTGGTAATTGTTTGGCCCAATACCTTCTGTCTTTTTCAGAGCAATCCATG

TCAACAGCTTCCACAATCTTTTACGTTACACGGATTGTGGCCGCAGGCGGAAGGTTCCTCGTTGAAATGTACGAGTGTA
CCAATGATTGATAGCATCTTAAAAGGCAACAAAGATGATTTGGAAAGGTATTGGCCAAACTTGAAGCACACAAAGTTCG
ATGAGAGTAAAAAATTCTGGATTAGTGAGTGGATTAAACATGGTAGCTGCTCCGCAAAGACTCCTGCTAATTACTTGAG
CCTGGTTTTTGATCTAATGAAGAAAATCAAGAAATTTGATGTGAAAAAGATATTTGAAAAGCACGGTAAGATA
>Rrugosa19_33_Rrugosa14_42
ATGAAAACCCCAGCAGCAGTCTGTCTTATTTTCTTGCTTTCTGTTATTCTGCTCAAGGGTTCCCACGCAGAGCCATACG
AGTATCTACAATTTGTTTTGCAATACTCCAAAGGCTACTGCTACAACACCCAGATATGTTTTGCGAGGTTGCCAAAAAT
GTTTACCATACACGGTGTCTGGCCCGCGAACATTTCCAATCCCCTGGTGAGATGTAAACAGTCAAAGAAACTACACCAG
TTCAAGCAAAACATGACAACATCACTGCAAAACGATCTTGGCCAATCTTGGCCCAATGTCGAAAGAGCTAAAACCAATA
TAGGGTTTTGGAAAGAGGAATACGAAAAGCATGGGTCTTGTACTGCACCAGCTATCACACAGAAAGCCTACTTTGAGAG
AGCTCACGAGCTGTGGAAGGAGTATGATCTCTATACAATTTTGGATAAAAAGCAGATCAAGCCGGGGCACTCATATGCA
CTCACTGATTTTGAAGCAGCCGTCAAGTCCAAGATCGGTACCGACACAAAACCTCTCATCCTTTGTAAGCAGGCCAAAC
TAACTCGTGGGAGCACAACAACCAACTTCATTTTGAGGGAAATTGTGATCTGTTTCGATCACCAGGGGACAAAT
>Rrugosa19_48
TTGTTTATTCATCCCAAAAACAAATACAGAGGGATGAAAATTATTCCTATTCTTGCCCTCCTATTATTCCTATTCCCCT
CAACTGTACAGGGCGAAATGAAATATTATGACGGTCAAATGCTTGTGTTACAAAATAGCAAAGCTGATTTATGGACGAT
TCATGGCCTTTGGACTTACGAACTTACCGGAAAAGAACCTGATTTCTACTATAAGCAACTTAGTGGCAAGGAACGATCG
ATACTCATTAGGGTTTGGCCTATTAACAATAAGAATATCAGGATAACCTATCAATTCTGGAAGTATGAATATGAAAAAC
ACAGTTCATGTACCGCAGATATATTACCAAGCTGTAGAGATTATTTAATGAAAGCAACTGTACTGTGGAATATACTCAA
GTTAGATGATTTGCTGGGTCCTAACGGCAAGTATAAGCCAAACTCTTCATTTCAAGCCCAGGACTTGCTGAACGACATG
GAAGTGAAGTATAAGGTCCGACCACTGTTGAAGTGTAATCTTCAGGGGGAACTACTGGAAGTTTGGTTTTGTTATACAA
AATTATGGAAAAGCATATTTTGCGCGCGCACGACAGACTCTTGTATGGGTCATATTAAATATGTTAGGGCA
>Rodorata_gigantea_3
ATGGCACAAAACACTTGCAAACCCAATGTGGCTTTTATCATCACTGCTCTGTCTTCGGTCTTCGGTCTGACATCATCAT
ATACACCCTACCAATACCTGTTGTATGTACAGCAGTGGCCCAAGTCTGTTTGTTATGATATCAACTGCCCTAACACTCC
CCCTCCAATCTTCACGACACACGGCCTATGGCCGTCGAACTATAATGGGCCTCGCTTGGTGTGCTCTGGTGGTGTGCCA
TTTAGTGAAACACTGGTAACACTTTATTATATGAATCAACAAACAATACTAAAAGCAAACTTGGAACAACATTCTTGGC
CGGATGTGGAAAAGGGGAACCATATGGGTTTCTGGAGGCATGAGTGGCAGGCTCATGGACAATGTTCAGATACCATCTT
TCCACAAACCAAATATTTCCAATGGTGTCACGATATGTGGAAGAAA
>Rodorata_gigantea_5
ACACGCTTTCGACACGTAAAATTGCATAATGAATTGAAATTATCGCATCCACAATCTTTTACACTACATGGATTGTGGC
CACAGGCGAATGGGAAATCCTTGGTGGACTGTGCTGGTTCAGAAATGGATGATAGCACCTTAAATGGCAAGAAAGATGA
TTTGAACAAGTATTGGCCAGACTTGACGCATTCCAAGTTTGAAGAGAGTAAAAGCTTTTGGATACATGAGTGGGAAAAA
CATGGTCGTTGCTCTGCAAAGAGTCCTGCTAATTACTTGAGCCTGGTTTTTGATCTGATGAAAAAACATGATGTGGAAC
AGATATTTAAAAACAATGGTAAGAAA
>Rodorata_gigantea_31
TTTACTCTTCACGGACTATGGGATCAGAATTTTGTCAAGATTAGTGGAATAACCCCCTTGAAGTCAATTCATCTAAGAG
GAGTTTCAACTCAAGATCTTCTCACCTATTGGCCAGACCTGTCTGCAGATCGAGCAACCCATGGGAGAGTCAACTGCTT
TTCATTTTGGAGGCATGAATGGGGATCCCATGGTGCACATATATGTGCAAGGTTTGGCTGCAGAAGAATCGACTATTTC
>Rodorata07_3
ATGGCATTCCTAGCAGTTAGGACACTAATTGTTCTTATGCTTATTTCTACATTTGCTAAAGCCGCAAACAAATATGACT
ATCTACAACTAGTGCAACAATGGCCTAAAACGTTCTGCCATAATAACCGAGCTTGCATTCAAGGTGCAGTCCTCCCGGA
GCTGTTCTCGATACATGGTATGTGGCCATCTAATTTCTCCGGCCAGAACGACGCTTGTATGCGTCGACACAATCATCTC
GAAACTCAATTGCTGTCATCCTCATGGCCAAGCTACACTGGTCTGTCCAACCTGCACTTTTGGGAGTATGAGTATAACA
AGCACGGCACTTGTTCAGAGAATAACCTCAAACAGACGGACTATTTCACCAGAGCCGACGCTTTGTGGAGGCGCTACAA
TGTTACCAATATGCTTTTAACATCGCGCCACCAAATCTCGCCGGGATCCTCCTATCGTTACGCTGACATTTTGTATGCC
ATTCGACAAGGAATTGGGGGATACACTCCTTTGATTCTTTGCAAGCAAGATCCGGCAAATAATATTTGGATTCTGCACG
AGGTGATAATTTGTTTCAATCCCTTGGGGAATAACGTCATCGCTTGTCGCAGAAGATCGAGATATAGTTGCAACAGCGG
GTTGATAGACTATCCTAAG
>Rodorata07_11
ATGAAGTCGATGATGTCTTCATTTCTCACCATTTTTCTCTGTTTTCAACTGCTCCTAGCAGTGCCAAATCCACTTCGAG
AGAAAATTTCAGCTCCTGATTCCTTCCTGTTGGTAATTGTTTGGCCCAATACCTTCTGTCTTTTTCAGAGCAATCCATG
TCAACAGCTTCCACAATCTTTTACGTTACACGGATTGTGGCCGCAGGCGGAAGGTTCCTCGTTGAAATGTACGAGTGTA
CCAATGATTGATAGCATCTTAAAAGGCAACAAAGATGATTTGGAAAGGTATTGGCCAAACTTGAAGCACACAAAGTTCG
ATGAGAGTAAAAAATTCTGGATTAGTGAGTGGATTAAACATGGTAGCTGCTCCGCAAAGACTCCTGCTAATTACTTGAG
CCTGGTTTTTGATCTAATGAAGAAAATCAAGAAATTTGATGTGAAAAAGATATTTGAAAAGCACGGTAAGATA
>Rodorata07_21_Rodorata_gigantea_19

ATGGAAACGGCATTGCTATGTACAGTCTCCATGCTGGTTATTTCTGCTTTTCCATCCGGTGCTAAAGCCGCGAATCAGT
ACGACTATCTTCAATTTGTTCAACAATGGCCGATAACCTTGTGCTACAACAACCCAGCCTGTATTCCAGGAGCATCACT
CCCACTGGACTTCTTTATCCATGGGTTATGGCCAAGCAATTTCTCTGGCCAGAATCAGCCCTGTATGCTTAGTCAATAT
CGTCTCCGAACCAAAATGCTACCATTCTCATGGCCAAGCTTCACTCGTAGATCCAACATGGGCTTCTGGAAATATGAGT
ATAATCAACACGGCACTTGTTCGGAGAATAACCTCGCGCAGACGGACTATTTTTACAAAGCCTATGCTCTCTGGATGCG
CTACAATGCAAACTTTATATTATATGCATCTACAGTTATACCAGGCCACAAGATTGTGCCGGGATACCTCTACCATTAC
GCTGACCTTCTAAACTCCGTTCAACAAGCAATTGGTGGATTCACTCCTTCGCTTATGTGCAAGCACGAGCCTGCAAATA
ATACTTGGATTCTGCACGAAGTCATCATATGTTTTGATGCCATGGGGAACAACGTCATCAATTGTGTTAGAGGATCTAG
TTGCTCTGCCACTACCACCGGGTATATACACTATCCTTTGCAA
>Rodorata07_31
CCCTACCAATACCTGTTGTTTGTTCAACGGTGGCCAAAGCCTGTTTGTTATGATATCAACTGCCCTAACACTCCCCCTC
CAATCATCACAGTACACGGTCTATGGCCATCGAACTATAATGGACCCCGCTTGAAGTGCTCAGGCGCTGTGACATTTAG
TGAAACACTGGTAACACATTATTATATGAGTCACCAAACAGTACTAAAAGCAAACTTGGAACAACGTTCTTGGTCGGAT
GTGGAAAAGGGAAACCATATAGGTTTCTGGAAGCATGAGTGGCAG
>Rodorata07_32_Rodorata_gigantea_11
ATGAAAACCCCAGCAGCAGTCTGTCTTATTTTCTTGCTTTCTGTTATTCTGCTCAAGGGTTCCCACGCAGAGCCATACG
AGTATCTACAATTTGTTTTGCAATACTCCAAAGGCTACTGCTACAACACCCAGATATGTTTTGCGAGGTTGCCAAAAAT
GTTTACCATACACGGTGTCTGGCCCGCGAACATTTCCAATCCCCTGGTGAGATGTAAACAGTCAAAGAAACTACACCAG
TTCAAGCAAAACGTGATGACAACATCACTGCAAAACGATCTTGGCCAATCTTGGCCCAATGTCGAAAGAGCTAAAACCA
ATATAGGGTTTTGGAAAGAGGAATACGAAAAGCATGGGTCTTGTACTGCACCAGCTATCACACAGAAAGCGTACTTTGA
GAGAGCTCACGAGCTGTGGAAGGAGTATGATCTCTATACAATTTTGGATAAAAAGCAGATCAAGCCGGGGCACTCATAT
GCACTCACTGATTTTGAAGCAGCCGTCAAGTCCAAGATCGGTACC
>Rodorata07_34_Rodorata_gigantea_2
ACAGTGGCATCAGTCATTGTGCTATTTTTCCTTCCACTCGGTGTGATTGGAAATGCGGTCGGACAGCCGAATGTGACCG
GAAATGCGGTCGGACAGCCGAATCACAATTATGACTACATGCAATTTGTGCAGCAATATCCCAAGACATTGAATGGAAG
CTCAGAATATACCGAGCGTTTCACCATTCATGGCCTATGGCCTAGCAATTCCTTCGGCTACGGTTCTGATTATCTAGAT
TGTCAAACACGATATGCTAACCGTTATCAACTTTCAGGACAGCTGACCAACGAACTCAAAAACACTCTCAGTTACTCTT
GGCCCAATGTTTACTACTATTGGCACGTTACAAAGTTTCAAAACAATTTGATCTTTTGGTACAACGAATACCAGAAGCA
CGGCAGCTGCGCTGTGGCCCTTCTGGGTATATTCCCAACAGCGCAGGCTTACTTTGAAAGAGCACATAAATTGTGGGAA
GCAAATGATTTGCATGGTGTATTCGTCAACGACAACATCCACCCGGGAGACCGAGTCGACGCTAGCAAATTTAGGACAG
CCCTGAGAACTGCGACTGGATGGAACGCTCGACTGTCGTGCACTGGAACCGGCCTACTGATGGAAGTAGTTATTTGTTA
TGATATTCATGCAACTCGAGTGAGGGATTGTCCTAGACGGGGGAATTGTACGGGGCTTATATCATATCCAAAA
>Rodorata07_35_Rodorata_gigantea_26_Rodorata_gigantea_27
CCCTACCAATACCTGTTGTTTGTTCAACGGTGGCCAATGTCTGTTTGTTATGATATCAACTGCCCTAACACTTCCCCTC
CAATCATCACGGTACACGGTTTATGGCCATCGAACTATAATCGACCTCGCTTGAAGTGCTCTGGCGTTGTGACATCTAG
TGAAACACTGGTAACACATATTATTATGCGATTATATATGAGTCACCAAACAGTACTAAAAGCAAACTTGGATCAACAT
TCTTGGTCGGATGTGGAGAAGGGAAACCATATAGGTTTCTGGAAGCATGAGTGGCAG
>Rodorata07_38
ATGGATCGCTCATTGGTATCTAAACTGAATATATCCTGGCCCAATGTGAAATATCCGAATAATATCCAGTTTTGGGATA
AACAGTGGAGAAAACATGGCTCATGTTCCGTGCACACGTTTAACCAAACAGAGTACTTCACGCAAGCGGATAATCTTTG
GAACTCACACAACATTACTGATATTCTTATAACTGGTGGGATCAAGCCAAATGGATCAGAATATGCATACGATACGGTA
GAGCGGCCCATCCAAATTGCAACCGGGAAGGAACCCGAACTTCGTTGCGCACCATCCTCCCTTGGAAGGCAATTGTTGC
ATGAAGTAGTACTTTGTTATAATCATAAGGGAACAACGCCCATCGACTGTAACCCTTTACATTCAACCTGCGATCGTAA
CTTCCAAATAAAGTTTATT
>Rodorata07_40
TTGTTTATTCATCCCAAAAACAAATACAGAGGGATGAAAATTATTCCTTTCCTTGCCCTCCTATTTTTCCTATTCCCCT
CAACTGTACGGGGCGAAATGAAATATTATGACGGTCAAATGCTTGTCTTACAAAATAGCAAAGTTGATTTATGGACGAT
TCATGGCCTTTGGACTTGCGAACTTACCGGAAAAGAACCTGATTTCTACTATAAGCAAGTTACCGATTGTTCACCATTT
GAAAGATTTAATTTAAATGAGCTTAGTGGCAGGGAACGATCGATACTCACTAGGGTTTGGCCTAGTAACAATAAGAATA
CGAGGATAACCTATCAATTCTGGGAGTATGAATATGAAAAACACGGTTCATGTACCACAGATATATTACCAAGCTGTAG
AGATTATTTAATGAAAGCAACTGTATTGTGGAATATACTCAAGTTTGACGATTTGCTGGGTCCTAACGGCAAGTATAAG
CCAAACACTTCATTTCAAGCCCAAGACTTGCTGGATGACATGGAAGTGAAGTATGAGGTCCGACCACTGTTGAAGTGTA
ATCTTCAGGGGGAACTACCGGAAGTTTGGTTTTGTTATACAAAAGTATGGAAAAGCATATTTTGCGCGTCCACGACAGA
CTCTTGTATGGGTCATATTAAATATGTTAGGACA
>Rodorata07_47
CTTGGTATGATCAGAGATGCGGTCGGACAGCCGGTCCCCCAGCCTGTCGGAGAGCTGGTCCCATCACTCCCATGGAGGG
TTGGACGGCCGTATTATGAGTACCTGCAATTTCTACTGCAAGTTCCCAGAACATTGAATCCAAGCTCAACACTTAACCA
GAATTTCACCATTCATGGCCTATGGCCTAGCAATCACAGCACTCATAATGGTGGAATTCCTTATCTGAGCCGTAACCTC

CAAAGGAATCTCAGTCACTCTTGGCCCGATGTAAAGCGGACTGCAAACGATTCGAAGTTTTGGACCGACGAATACAGGA
AGCACGGCACCTGCGCAAACCGTATATTCCGTACACCAGGGGATTACTTTAGAAAAGCAGATGACTTGTATCGCAGTCA
TCCCATACAACGTTTTTTTAATCTATTCGCCAACCACATCCGCCCAGGACAGCAAGTCCCCGTGAGCACATTT
>Rmoschata08_2
AATGCTTATTGCTCATTAAGGGCTTGTGTTGCACCTATCCCACAGTCTTTTACTCTTCACAGACTATGGCATCAGAATT
TTGTCAAGATTGGTGGAATAACCCCCTTGCGGTCAATTCATCTAAGAGGTGTTTCAACACGAGATCTTCTCAACTATTG
GCCAGACCTCTCTAGAGACCGAGCAATTTGGAAGCATGAATGGGAATCCCACGGCGCACATATATGTGCAAGGCTTGGC
TGCAGAAGAATAGAAGTCTATTTCAGTGTGTCT
>Rmoschata08_11
ATGAAAACGCCAGCAGCAGTCTGTCTTATTTTCTTGCTTTCTGTTATTCTGCTCAAGGGTTCCCACGCAGAGCCATACG
AGTATCTACAATTTGTTTTGCAATACTCCAAAAGCTACTGCTACAACACCCAGATATGTTTTGCGAGGTTGCCAAAAAT
GTTTACCATACACGGTGTCTGGCCCGCGAACATTTCCAATCCCCTGGTGAGATGTAAACAGTCAAAGAAACTACACCAG
TTCAAGCAAAACATGATGACAACATCACTGCAAAACGATCTTGGCCAATCCTGGCCCAATGTCGAAAGAGCTAAAACCA
ATTTAGGGTTTTGGAAAGAGGAATACGAAAAGCATGGGTCTTGTACTGCACCAGCTATCACACAGAAAGCGTACTTTGA
GAGAGCTCACGAGCTGTGGAAGGAGTATGATCTCTATACAATTTTGGATAAAAAGCAGATCAAGCCGGGGCACTCATAT
GCACTCACTGATTTTGAAGCAGCCGTCAAGTCCAAGATCGGTACC
>Rmoschata08_17
AATGCTTATTGCTCATTAAGGGCTTGTGTTGCACCTATTCCACAGTCTTTTACTCTTCACGGACTATGGGATCAGAATT
TTGTCAAGATTAGTGGAATAACCCCCTTGAAGTCAATTCATCTAAGAGCAGTTTCAACTCAAGATCTTCTCACCTATTG
GCCAGACCTGTCTGCAGATCGAGCAACCCATGGGAGAGTCAACTGCTTTTCATTTTGGAGGCATGAATGGGGATCCCAT
GGTGCACATATATGTGCAAGGTTTGGCTGCAGAAGAATCGACTATTTCAATTGGTCTCTTAAACTTGCAAGCCAATGCA
TTAACGACCTTGAAGCTCTTTTTGCCAAATACGGGAATATTCGGGATGGAAAGACCATGTATGATCCAAGATATGTACG
CAGCATTATCTTTCGAGAGCTAGAGGTGCATGTGTTGGTGATATGCAAGCCTCTGCGTGACAGTTACATTCTCTTTGAA
ATTCATTTTTGCTTCACTACTAGAAATGTGGCCTATGGCCACAGCCCAACA
>Rmoschata08_18
ATGTTATTGAATCTAATCAGCTGCTCTTATATAAGTTTTGACATTCTTCCTTTCTGTTTTTTTTTTGTTTTTTTTTTTT
TCAACACAATAATGTCTCTAGGTTCATATGATCATTTTAAATTTGTACAACAATGGCCACCGGCCGTCTGTGATACGTC
TGGCTGCAAGAGATCGGGGTTATCACACTTCACGATCCATGGCCTTTGGCCAAATAATAAAACGTATGTGAAAAATCAA
CCGACTTGTCTTACCAGTCATGCCAACTCATTTAAGGCTACGATACTCACTAGTTCGAATTTGGTAAGCAAGCTGAGCA
CCTCTTGGCCCGATGTGAAAAATGCAAATGATAAAGATTTTTGGAACAAACAATGGGACAAGCATGGCACATGTTCCTT
GCAGACATACGACCAAGCACAATACTTCGAGCGGTCATACAACATGTGGAAAGAAACCAACCTGACAAACACCTTGGAT
AGCCTCATCAAACAGACACCGAGACAACAGAACGTCACAGATATAGAACAACTCATTCAAGGAGTAACTGCAACTGAAA
AGAAACCCCTCCTCCGTTGTGAACAATCTCATAGTAATGCCAGTAAGTCTCTCTTGAAAGAGATTGTCATTTGCTATGC
ACACAACGGGATTACTGTGATCGATTGCGTCAGTCGACCAAAATCCACCTTCGAATGCTATGACGGATTTTGGTTTCCT
>Rmoschata08_25
AGAACGATGGCACAAAATACTTGCAAGCCCAATGTGGCTTTTGTCGTCACTGCTCTAGGTTTGGTCTTCAGTCTGATGT
CATCATATACACCCTACCAATACCTGTTGTTTGTTCAACGGTGGCCAAAGTCTGTTTGTTATGATATCAACTGCCCTAA
CACTCCCCCTCCAATCATCACGGTACACGGTTTATGGCCATCGAACTATAATCGACCTCGCTTGAAGTGCTCTGGCATG
AGTCACCAAACAGTACTAAAAGCAAACTTGGATCAACATTCTTGGTCGGATGTGGAGAAGGGAAACCATATAGGTTTCT
GGAAGCATGAGTGGCAGGGTCATGGACAGTGTTTAGACACCGTCTTTCCAGTTTCCACATACCAAATATTTCCAATGGT
GTCGCGATATGTGGAAGAAAAACGTGATAGGCGAGATCCT
>Rmoschata08_41
TTGTTTATTCATCCCAAAAACAAATACAGAGGGATGAAAATTATTCCTTTTCTTGCCCTCCTATTTTTCCTATTCCCCT
CAACTGTACGGGGCGAAATGAAATATTATGACGGTCAAATGCTTGTCTTACAAAATAGCAAAGTTGATTTATGGACGAT
TCATGGCCTTTGGACTTGCGAACTTACCGGAAAAGAACCTGATTTCTACTATAAGCAAGTTACCGATTGTTCACCATTT
GAAAGATTTAATTTAAATGAGCTTAGTGGCAGGGAAAGATCGATACTCACTAGGGTTTGGCCTAGTAACAATAAGAATA
CGAGGATAACCTATCAATTCTGGGAGTATGAATATGAAAAACACGATTCATGTACCACAGATATATTACCAAGCTGTAG
AGATTATTTAATGAAAGCAACTGTATTGTGGAATATACTCAAGTTTGACGATTTGCTGGGTCCTAACGGCAAGTATAAG
CCAAACACTTCATTTCAAGCCCAAGACTTGCTGGACGACATGGAAGTGAAGTATGAGGTCCGACCACTGTTGAAGTGTA
ATCTTCAGGGGGAACTACTGGAAGTTTGGTTTTGTTATACAAAAGTATGGAAAAGCATATTTTGCGCGTCCACGACAGA
CTCTTGTATGGGTCATATTAAATATGTTAGGACA
>Rmoschata17_4
CACCAGTACCTAGCTCCCAACTTTCCAGCAACTTATCCCCATTTGACCATTCACGGTCTCTGGCCTACCAATTACAGCC
GTCGTGCTGTTGTTTCCGTTGAAAACTGTAGTTTTACAGATTATACGCTGTCTGCTAAATTGGAAAAGGAGCTCGATAC
GTTTTGGCCCAATGCTTATGGTCCTTCAAAGAATCAGGGTTTTCGGATCCACGAATACCGAAGACACGGCAGCTGCACA
AAGCTAAAGTTCAGATCAGCGGAGGATTACTTTGGAACGGCTCTTTACTGGTATAATCATATAATGACGAGTTGGATAA
CGCATAAAGTGCCAGCTCTTTCCCAAGAAGGGTCTCACGGAGTCTTAACTAGTACGTTTTCAACGTCCGTCGAACTAGC
GATTGGGGGAAAA

>Rmoschata17_14
TCCTATGAATATTTTAAATTTGTGGTACAATGGCCACCAGCCCACTGCAGAGTTAGAAAATGCACACCCCAAGCCTTGC
AAAAGAAAATTTACACCATCCATGGCCTATGGCCGAGTAATTATTCTAAAGCTGTCGTGTATAAGTGTCCCGGATCGTC
TTTTCAAAATCCGGTAAATGCTCCTCCATTGGAAGCCAAACTGAAGATCTCTTGGCCCAACCTAGAACTTCCGAATGAT
GCAATGTTTTGGGCACTT
>Rmoschata17_31
TTCACCATACATGGGTTATGGCCTAGCAATTTCTCCGGCCAAAATAACCCATGTGTGGGTACGCCATTTAATAGAGCTC
AGATGTCCGCAAACTATCGTCTTCAAACCATATTGCTCCCATCCTCGTGGAGAAGCTACCCTATCGGTGTCTCCAACAC
AGACTTCTGGGAGAATGAGTATAACAAACATGGCACATGTTCGGAGCTCAATTTGCCACAGACGGAATACTTCACCAAA
ACCCATTGGCTATGGATGGTCAACAACATTTATAGTATTTTTTCATCTTCGGCCAACAGACAAGGATATCTAATCTTGC
CGGGTTTCACCTATGACTAC
>Rlaevigata_12
ATGAGGCCTAAGAGTTGTATTCTCAATTCTAGGTGGCCTCAATCATTTTCCTTCATGATATGCATTAATCAAAACTATA
TACAACTGGTCCCACAGCGGGTCGGACAACCGTATTATGAGTATCTGCAATTTGTACTGCAAGGTACGACAACAGTGAA
TCCGTCAAATCCAGACTTAAGCCATTACCCGCCTTTCACCATTCATGGCCTGCGGCCTAGCAATTTCAGCCGTTTTCGT
GATCCAATTCCTTACATTCAAAATTGTAATCCAAATCGAAAATATAACCCTAAAACTTGGGCAGGTGACAACGACCTCA
AAAATGATCTCCGTCAATTTTGGCCCGATGTAGAGCGTCTAAACAATTTGAGGTTTTGGACCCACGAATACAGGCAGCA
CGGCAGCTGCGCCAACCTTGTATTCGAAAGCCGGGAGGGCAACCTTGTACTCGAAAAACGGGAGGCTTACTTTAGAACA
GCAGATTACTTGTATCATCATCGCATACCAAATTTGTATGACTTATTCAACAACATCCGCATCCCCCGCGGCCGCTGGT
CTGGCACAGTCAACACTACTACATATATGTTCGAGATACGGGATGCGAATGGAGGGTTTCCACGACTGCGGTGCAATGG
AACAAATAACCACTTCCTGAGGGAAGTAGTTATTTGTTACGATAAAGATGCAATTAACGTGATTGATTGTCCATTCTTA
GGGTGGAATTGTCCCAAGTTCATAGCATTTCCAATA
>Rlaevigata_16
ATGATGTCTTCATTTCTCACCATTTTTCTCTGTTTTCAACTGCTCCTAGCAGTGCCAAATCCACCTCCAGCGAAAATTT
CAGCTCCTGATTCCTTCCTGTTGGTAATTGTTTGGCCCAATACCTTCTGTCTTTTTCAGAGCAATCCATGTCAACAGCT
TCCACAATCTTTTACGTTACACGGATTGTGGCCGCAGGCGGAAGGTTCCTCGTTGAAATGTACGAGTGTACCAATGATT
GATAGCATCTTAAAAGGCAAGAAAGATGATTTGGAAAGGTATTGGCCAAACTTGAAGCACACAAAGTTCGATGAGAGTA
AAAAATTCTGGATTAGTGAGTGGATTAAACATGGTAGCTGCTCCGCAAAGACTCCTGCTAATTACTTGAGCCTGGTTTT
TGATCTAATGAAGAAAATCAAGAAATTTGATGTGAAAAAGATATTTGAAAAGCATGGTAAGATA
>Rlaevigata_18
GGTTCCCACGCAGAGCCATACGAGTATCTACAATTTGTTTTGCAATACTCCAAAGGCTACTGCTACAACACCCAGATAT
GTTTTGCGAGGTTGCCAAAAATGTTTACCATACACGGTGTCTGGCCCGCGAACATTTCCAATCCCCTGGTGAGATGTAA
ACAGTCAAAGAAACTACACCAGTTCAAGCAAAACGTGATGACAACATCACTGCAAAACGATCTTGGCCAATCTTGGCCC
AATGTCGAAAGAGCTAAAACCAATATAGGGTTTTGGAAAGAGGAATACGAAAAGCATGGGTCTTGTACTGCACCAGCTA
TCACACAGAAAGCGTACTTTGAGAGAGCTCACCAGCTGTGGAAGGAGTATGATCTCTATACAATTTTGGATAAAAAGCA
GATCAAGCCGGGGCACTCATATGCACTCACTGATTTTGAAGCAGCCGTCAAGTCCAAGATCGGTACC
>Rlaevigata_19
CCACGAGGACTCTGCTATAACACAACAAAAAGTTATTGCATCTCACCTCTGCCAACCAAGTTTCATGTACACAGGATAT
GGCCTTCTAACTTCTCCGATATTATTATGACACCTCTGCAGACGGATTTGGTAAACTCATGGTCGTCCGTGTTAACAGC
AAAGAGCAATATGTGGCTCTGGGAGCATGAGTACGAGAACCATGGCGCATGTGCAGTAGAGTCAGGTGTACCACCTTTC
ACTCAGAAGTCCTACTTTGAGAAAGGGCACCAGCTATGGAACCAATATGACATCCATGCAGTGCTTAATCAAAGTGGTA
TTGTACCGGGTACTGCAACATCATATACTAGCATTGTAACCGCAATCCAAAAGAAAATTGGGAGTAACAATACTCCTTT
AATCAAGTGCAGGACGACTGTGAAGACCCGAAATTTCAGT
>Rlaevigata_21
ATGGCATTCCTAGCAGTTAGGACACTAATAATTGTTCTTATGCTTATTTCTACATTTGCTAAAGCCGCAAAAAAATATG
ACTATCTACAACCAGTGCAACAATGGCCTAAAACGTTCTGCCATAATAACCAAGCTTGCATTCAAGGTGCAGCCCTCCC
GGAGCTGTTCTCGATACATGGTATGTGGCCATCTAATTTCTCCAGCCAGAACGACGCTTGTGTTGGAACTCGGTTCAGC
ATGAGAGAGATGCGTCGACACAATCATCTGGAAACTCAATTGCTGTCATCATCATGGCCAAGCTACACTGGTCTGTCCA
ACCTGCACTTCTGGGAGTACGAGTATAACAAGCACGGCACTTGTTCAGAGAATAACCTCAAACAGACGGACTATTTCAC
CAGAGCCGACGCTTTGTGGAGGCGCTACAATGTTACCAATTTGCTTGTAACATCGCGCCACCAAATCTCGCCGGGATCC
TCCTACCGTACGTTACGC
>Rlaevigata_24
AACGATGGCACAAAATACTTGCAAGCCCAATGTGGCTTTTGTCGTCACTGCTCTAGCTTTGGTCTTCACTCTAACGTCA
TCATATACACCCTACCAATACCTGTTGTTTATCGACGGTGGCCAAAGTCTGTTTGTTATGATACCAACTGCCCTAACAC
TCCCCCTCCAATCATCACAGTACACGGTCTATGGCCATCGAACTATAATGGAACTCGCTTGAAGTGCTCTGGCATGAGT
CACCAAACAGTACTAAAAGCAAACTTGGAACAACATTCTTGGTCGGATGTGGAGAAGGGAAACCATATAGGTTTCTGGA
AGCATGAGTGGCAGGCTCATGGACAGTGTTTAGACACCGTCTTTCCAGTTTCCACATACCAAATATTTCCAATGGTCTC
ACGATATGTGGAAGAAAAACGTGATAGGCAAGTTCCT

>Rlaevigata_25
AATCTGGAGCACTTGAGATTGGCCCTGACCAATCCAAATGCTTATTGCTCATTAAGGGCTTGTGTTGCACCTATTCCAC
AGTCTTTTACTCTTCACGGACTATGGGATCAGAATTTTGTCAAGATTCGTGGAATAACCCCCTTGAAGTCAATTCATCT
AAGAGGAGTTTCAACTCAAGATCTTCTCACCTATTGGCCAGACCTGTCTGCAGATCGAGCAACCCATGGGAGAGTCAAC
TGCTTTTCATTTTGGAGCCATGAATGGGGATCCCATGGTGCACATATATGTGCAAGGTTTGGCTGCAGAATAATCGACT
ATTTCAATTGGTCTCTTCAACTTGCAAGCCAATGCATCAACGACCTTGAAGCTCTTTTTGCCAAATACGGGATTATTCG
GGATGGAAAGACCATGTATGATCCAAGATATGTACGCAGGATTATCTTTCGAGAGCTAGAGGTGCATGTGTTGGTGATA
TGCAAGCCTCTGCGTGACAGTTACATTCTCTTTGAAATTCACTTTTGCTTGGATATGACAAGGGACTTTGCGGATTGTA
CCAACTGGTACAGGGAGGAT
>Rlaevigata_27
ATGAAGAATTCAAACTTCATTATCCTTCTATCAGTATATGCACTTGTACAAGTTCTCAACGTTTCAAATGCTTCACCCT
ACGAGTACATGCAATTTGTACTCCAGTACCCACCTGGAGTTTGCTATGACACATCAAAAGGTTATTGCGTCTCACCTCT
CCCAACCAAGTTTCATGTACATGGAATATGGCCTTCTAACTTCTCCAATATACTTGTCCGTTGTGGGCACGCACTGAGG
AATAACCCTTTTCATGAGGCGCAGATGACACCTCTGCAGACGGATTTGGGAAACTCATGGCCGTCCGTGTTAACACAAA
ACAACAATATGCAGTTCTGGGAGCATGAGTATGGAGAGCACGGTGCATGTGCAGTAGAGTCAGGTGTACCACCTTTCAC
TCAAAGGTCCTACTTTGAGAAAGGACACCAGCTATGGAACCAATATGACATCCTTGCAGTGCTTCATCAAAGTGGTATT
AAACCGGGTACTGCAACATCATACACTATGACTCAGCTTGTAACCGCAATCCAAAAGAAAATCGGGAGTAACAATACTC
CTTTAATCATGTGCAGGAGGACAGCCCTTGGTTACACACTAAAGGAAGTGATAATCTGTCTGGATCACCAGGCAACAAA
TGTGATAAGTTGCGCGCTCAATAATATTAGAAAAACAGATTGCCGAGATCCTTCGGGCAAGGTATACTATGTAGCT
>Rlaevigata_30
TCATCATATACAGCAAAAATGAAAACACCAGCAGTCTGTCTCCTTTTCTTGCTCTCTGTTATACTGTTCAAGGGTTCCC
ATGCAGAGCCATACGAATACCTACAGTTTGTCTTGCAATACCCTAGAGGTTACTGTGTTAACGACAAGAAATGCATTCC
ACCGGCGAGCTTGCCAGCCAAATTCACGGTTCATGGAATTTGGCCCACGAATATTTCCGAGCCGACTATCAGCTGTGAC
AAGGCACAGAAACTACACTCGTTCAACGGAAACCTGATAACTCCGTCGTTGAAAACCGATCTTCACCGATCTTGGCCCA
GCGTCCTGACAGATAAAGATGATATGTCGTTTTGGGAACACGAATATAATAAGCATGGGTCCTGTACTGCACCAGCTAT
CACTCAGACAGCGTATTTTGAGAGAGCTCACAAGTTCTGGAAGGAGTATGATCTCTATTCCATTTTGGAACAAAAAAAT
ATCAAGCAGGGGCAATCTCGATCGTATTCACTGGCTGATTTTGAAGCAGCCGTCAAGTCCAAGATCGGTACC
>Rlaevigata_32
CCACCTCCAGCGCCCACTCCACCTCCTGATTCCTTCATTTTGGTCATTGTGTGGCCCAATACCTTCTGTCTTTTTGAGT
CATGTCAGCTGCATCCACAATCTTTTACACTACATGGATTGTGGCCACAGGCGAATCGGAAATCCTTGGTGGACTGTGC
TGGTTCAGAAATGGATGATAGCACCTTAAATGGCAAGAAAAATGATTTGAACAAGTATTGGCCAGACTTGACGCATTCC
AAGTTTGAAGAGAGTAAAAGCTTTTGGATACATGAGTGGGAAAAACATGGTCGTTGCTCTGCAAAGAGTCCTGCTAATT
ACTTGAGCCTGGTTTTTGATCTGATGAAAAAACATGATGGGGAACAGATATTTAAAAACAATGGTAAGAAA
>Rlaevigata_33
TGGCCAGGCTCATACTGTACAGCAGCAAAGCAGGGTTGTTGCTACCCAAAGATTAGAAAACATCCCAGTTTCACAATTG
GTGGCATATGGCCTTATACTTTTTGTGGGGATAGACCAACCTACTGCAAATCTAAGACCCCTTATAGTTTATCTAAGAT
ATCAAACCTGACCAAGAGTTTGGAAAGGAATTGGCCATCACTACCCTGCCCAAGCCGTACTAGTAGCATTAGCAGTAGG
AAACTATGGATGCAAGAATGGCAGAAATATGGGACTTGTGCGGAATCACTCTTTGGAGGTCAATACCAATACTTTCATG
CAGGTCTCAATCTGAGGAAGAAAATAGACATGCTCCAGATACTCAGCAATGCAGGTCTGATCACTACTATAATCCAACT
TTCGGATTTCGGTTCAAATAAATTAGTGGAACTC
>Rpersica_5
TGGCCAGGCTCATACTGTGCAGCATCAAAGCAGGGTTGTTGCTACCCAAAGAGTATTAAAAAACATCCCAGTTTCACAA
TTGGTGGCATATGGCCTTATACTTTTTCTGGGGATAGACCAACCTACTGCAAATCTAAGACCCCTTTTAGTTTATCCAA
GATCTCAAACCTGACCAAGAGTTTGGAAAGGAATTGGCCATCACTATCCTGCCCAAGCCGTAGTAGTAGCAGTAGCAGT
AAGAAACCATGGATGCAAGAATGGCAGAAATATGGGACTTGTGCAGAATCACTCTTTGGATGTCAATACCATTACTTTC
ACGCAGGTCTCAATCTCAGGAAGAAAATAGACATGCTCCAGATACTCAGCAATGCAGGTCTGATCACTACCATAATCCA
ACTTTTGGTTTTCATTTTAGGCAGACTAGTCGAACTCAGAACTTTTCACTTGTCGGTATTGGATGCAGGAATCCATCCA
AACGGGTCATTTTATGAGATGACTGCATTTTTCAATGCCATGCAAAAGGCCACTATCTATCTTCCGGGGTTTATCTGTA
ACAAAGACAAATCAGGCAACAAACAGCTCTACCAGATTATACTCTGTGGAAACACCGCCGGAACTAGAATCACAGACTG
CCTCGGCAACCCAAGATCGAATACTGGACAATGCCCCGAAAAATTTAAGTTCCCTCCCCTGAAA
>Rpersica_8
CTACAGTTTGTCTTGCAATACGCTAGAGGTTACTGTGTTAACGTCAACAAATGCATTCCACAGGCGAGCTTGCCGGTCA
AATTCACGGTTCATGGAATTTGGCCCACGAATATTTCCAAGCCGGTGATCGAGTGTAAAGCTGCACAGAAACTACACCA
ATTCAACAAAAAGCTGATAACACAGTCGCTGGAAACCGATCTTCTCCAATCTTGGCCCAGCGTCCGGACAGATAAAGAT
AATATGACTTTTTGGAAAGACGAATATAATAAGCATGGGTCCTGTACTGCACCAGCTATCACTCAGACAGCATATTTTG
AGAGAGCTCACAAGTTCTGGAAGGAGTATGATCTCTATTCTGTTTTGGAACAAAAA
>Rpersica_11

CTCAGAAATGCAGTCGGAGAGGCGGCCGGACAGCCTTATTATGACTACCTGCAATTTGTGCAGCAATATCCCAGAACAG
TGAATCGAAGCTCAAGATTAGACAATTTCACCATTCATGGCCTATGGCCTAGCAATTACAACAATATTTATGTTATATA
TTGTGGTCAAAATATCAGCGAGACTGCACTTAAAAAGCAGCTGAGCCCCGGCCTCCGAGGTAATCTCACTCACTCTTGG
CCCGATGTAAGGAAGGCAACCCATTCGGGGTTTTGGACCAACGAATCCAATAAGCACGGCACCTGCGCCAGCCATACAT
TCACAACGCCGAAGGAATACTTTGAGAAAGCAGATTGCTTGTATCGCAACTTCTCTGGCGGACAAAATTTGTATGATTT
ATTCCTCCGCGGCAAGCAACAC
>Rpersica_12
CCGTGCTGCTTGTTTCTGCTTTTTCATCAGGTGCTAAAGCCGCCAATATGTACGACTACTTCCAGTTTGTCCAGCAATG
GCCGGCCGGCTACATTCTGCTATCGTAATCCGGATTGTGTCCGGAATCCCCCGCGGAATACTTTTACCATTCATGGCCT
GTGGCCAAGTAATTACACGACACCTGAAAAGCCATGCGTTGGCACATCCTTTAACTATTTTCTGCCGCAAGCATGGCCC
CAGTTAATTGCTCGTTTCACCAATATGCGATTCTGGCAGAGCGAATATAACAAGCACGGTACCTGCTCGGAGAACAAAC
TCAAGCAGACGGAGTATTTTAGGACAGCGCACTGGGCGTGGGTTCGATTCAATGCATATTTTCTGTTTGCAGACTCGCC
TCTCCAAATCCACCCTGGTAATTACTATCTCAAAGCTGACCTTGACCAAGCAATCGGGCAGTCACTACTAAGGACCCTC
TGCTTATGTGCAAGAAGATCAAACCCATGGGCATTGATACGTGGC
>Rpersica_14
TATTGCTTATTCTACGTTCTTTCATACGCAGTAATATGCATAGGATCTTATGATTATTTCCAATTTGTCCTACAATGGC
CACCAACGGTGTGTAGAATGAAACCATGCATCCAGGCAAATCCGCCCAGAAACTTCAGCATCCATGGTCTGTGGCCAAG
TAATTATTCATTCAATATGGTGCCAAGTGTCTGCGGTGGGGCGGCATACGTCCCCGCTGTGACTAATGCGTTAGCACCT
TTACTAAGAGTCTCTTGGCCGGACGTGGAAAAAGGCAACGAACAGGACTTCTGGAAACTTGAGTGGGACAGACATGGCA
AATGTTCCGAACAAACATTAAACCAAACAAGATACTTCACACAATCCCACAACATTTGGAATCGATATCATATTCTTGA
TATCCTTAAAAACTCTCAAATAGAACCAAACGGTAGTTTGTACCTTTACACAGATATACAATCCTCCATCCGAAAAGCA
ACTCAAATGACACCTCGTATTCGAACGAAGCAACTTTTAACCGATCCTAAGAACGCATCATCGTTTGCTTTCTACTTGC
ACGAAATGGTCCTATGTTATAAGTATGACGGGGTGACACCGGAAAACTGCTCGGCAGCAGGCTCACGGCAACAATATGT
CAAATTTGCTATAAAA
>Rpersica_17
ATGATGTTTTCATTTCTCACCATTTTTCTCTGTTTTCAACTGCTCCTAGCAGTGCCAAATCCACCTCGAACGCCAATTC
CAGCTCCTGATTCCTTCATGTTTGTAATTGTTTGGCCCAATACCTTCGGTCTTTTTGAGTGCGATCCATGTCAACAGCT
TCCACAATCTTTTACGTTACACGGATTGTGGCTGCAGGCGAAAGGCATTTCCTTGTCAAAATGTGAGGGTGTAATGAAT
GATAGCACCTTAAAAGGCAAGAAAGATGATTTGGAGAGGTATTGGCCAAACTTGAAGCATACAAAGTTCGATGAGAGTA
AATACTTCTGGATTAGTGAGTGGAATAAACATGGTAGCTGCTCCGCAAAGACTCCTGCTAATTACCTGAGCCTGGTTTT
TGATCTAATGAAGAAAATCAAGAAATTTGGTGTCAAAAAGATATTTGAAAAGCATGGTAAGAAA
>Rpersica_20
GGCTCTATATATAGACTTACATGTTTGTTGAACCATACGAATTCTAGCTGGCTGCAATATCAGCAAAATGAAAACGCCA
GCAGCAGTCTGTCTTATTTTCTTGCTTTCTGTTATTCTGCTCAAGGGTTCCCACGCAGAGCCATACGAGTATCTACAAT
TTGTTTTACAATACTCCAAAGGCTACTGCTACAACACCCAGATATGTTTTGCGACGTTGCCAAAATGTTTACCATACAC
GGTGTCTGGCCCACGAACATTTCCAATCCCCTGGTGAGATGTAAACAGTCAAAGAAACTACAGCCGTTCAACCAAAACG
TGATGACAACATCACTGCAAAACGATCTTGGCCAATCTTGGCCCAATGTCGAAAGAGCTAAAACCAATATAGGGTTTTG
GAAAGAGGAATACGAAAAGCATGGGTCTTGTACTGCACCAGCTATCACACAGAAAGCGTACTTTGAGAGGGCTCACGAG
CTGTGGAAGGAGTATGATCTCTATACAATTTTGGATAAAAGTCAGATCAAGCCGGGGCACTCATATGCACTCACTGATT
TTGAAGCAGCCGTCAAGTCCAAGATCGGTACC
>Rpersica_23
AGAACGATGGCACAAAAGACTTGCAAGCCCAATGTGGTTTTTGTCGTCACTGCTTTGGCTTTGGTCTTCAGTCTAACGT
CATCATATACGCCCTACCAGTACCTGTTGTTTGTTCAACAGTGGCCAAACTCTGTTTTTTATGATATCAATTGCCCTAA
CACTCCCCCTCCAATCGTCACGGTACACGGTCTATGGCCGTCAAACTATAATTCGCCTCGCTTGAAGTGCTTTAGCGGT
ATAAGTCACCAAACAGTACTAAAAGCAAATTTGGAACATTCCTGGCCGGATGTGGAAACGGGAAACCATATGGGTTTCT
GGAAGCATGAGTGGCAGGCTCATGGACAATGTTCAGACACCGTCTTTCCACAAACCAAATATTTTCAATGGTGCGTGTC
ACGAATGTGGAAGAAAAACGTGATAGGCGAGATCCTTGAAAGGTCGACGTCCATCATCCCAGGACCCAAAAAGTATAGC
TTGTCTGCCATAGAAAATGCTAACAGAGCAAACACTTCGCATGACCCTTATATTCGGTCTGCCCAAGGTTCGGAAGAT
>Rpersica_25
ATGAGTGGTGGAGCCTATCATTTTTTCCAGTTTGTGCAACAATGGCCACCAACTCTTTGCAAAATTAAAAATTGCGTTG
TCCCAAACGCACCCAGAAACTTCAGCATCCACGGTTTGTGGCCAAGTAATGCTTCATACAGTTCGGGGGGTTTGTGTCC
TGGGGCGCGATTTGACTACGGTTTGGCAGGTGTGTTAGCACCTCTATTACAAAAATCTTGGCCGGACGTGGAAAAAGGT
AACGACACGAAATTTTGGAAACTTGAGTGGGATAGGCATGGAAAATGTTCCGATCAGACATTCGACCAAAGAAGATACT
TCACACAATCCCACAACATTTGGACGAGATATCATATTCTTAATATCCTTAAAAACTCTCAAATAGAACCAAATGGTAG
TTTTTACTCTTACACATCAATAGAATCCTCCATCCGAAGTGCAACTCAAACGAGACCCCGTATTCGAACAAGGAATCTT
CCAACTGATCCTAACAACAATACGTATGCTCACTACTTGCACGAAATAGTCCTTTGTTTTAAGTATGATGGGGTGACAC
CGGACAACTGCTCCGCGGTAGTCGGACCACCAGGAGATGTCAAATTTGAA
>Rxanthina_2

CCAATTCCAGCTCCTGATTCCTTCATGTTGGTAATTGTTTGGCCCAATACCTTCTGTCTTTTTGAGAGCGATCCATGTC
AACAGCTTCCACAATCTTTTACGTTACACGGATTGTGGCCGCAGGCGAAAGGCATTTCCTTGTCGAAATGTGAGGGTGT
AATGAATGATAGCACCTTAAAAGGCAAGAAAGATGATTTGGAAAGGTATTGGCCAAACTTGAAGCATGCAAAGTTCGAT
GAGAGTAAATACTTCTGGATTAGTGAGTGGAATAAACATGGTAGCTGCTCCGCAAAGACTCCTGCTAATTACTTGAGCC
TGGTTTTTGATCTAATGAAGAAAATCAAGAAATTTGATGTGAAAAAGATATTTGAAAAGCATGGTAAGAAA
>Rxanthina_7
ATGGGTGGTCTCCACTACAAATTACCTACACTTGTGAGTGTAGTAACAGTGGCATCAGTCATTGTGCTATGTTTCCTTC
CACTTGGTGTGATTGGAAATGCAGTCGGAGATGGACAGCCGGTCACAGATGCGGTCGGACATCCGCCCAGATATCAGTA
CATGCAATTTGTACTGCAAGTTCCCAAAACGGTGAATCCATACTCACCTAACCAATACCCGCATTTGACCATTCATGGT
CTATGGCCTGGTTTTTCCGACAATAGTCGTCTTGAATTTTGTAATCCGCCATCTTATGACCAGCATCAACTTTCAACGC
TGCCCCTCCAAAATAATCTCGCTCACTCTTGGCCCAATGTAAAGAGTCCAAACAATTTGGAGTTTTGGTCCAAGGAATA
CAACAGGCATGGCAGCTGCGCCTACCATATATTGGCTGGATTGAAAAACCCGAAGACTTTCTTGGAGGCTTACTTTACA
GAAGCAAATAATTTGTATCGCCCTCAGTACGCACAACATTTGTATCAGTTATTCCACTACATCCGCCGCCAGAGCCCGT
ATGCCATTGTCCGCAACAGCCAGTCTGGCAGGATCGACATTTGGAGTTATTTGCAGGCGATTGAAAATGAGAATGGAGG
GAAAGCTCCACGACTGTTGTGCAATGAAACAAATAGCTACTTACTGAAGGAAGTAGTTATTTGTTACGACATGTTTCTA
AAATACGTGATTCCATGTCCTTTTCCAATAGGGAAGTATTGTCCGCGGTTCATATCATTTCCAATA
>Rxanthina_8
TTGGGATCTCCACTACATACACTACCTACACTTATGGCTGCAGTAACAGTGGCATCAGTCATTGTGCTATTTTTCCTTC
CACTTGGTGCGATCAGAAATGCAGTCGGACAGCCGGTCGGACAGCCGTATTATCAGTACCTGCAATTTGTACTGCAATA
TCCCAGAACAGTGAATCAAAGCTCAGATATTGAAAAGAGATTCACCATTCATGGCCTATGGCCTAGCAATTACAGCAAG
GCTCAAAATCGTCGTGTTAGAAATTGTAGTCCAAAATATCCCGCTGATATTCTTTTAAAGCAGAATCTCGATGAGTCTT
GGCCCGATGTAGTATATTTAAACAATTCGAACTTTTGGACCAAGCAATACAGAAAGCACGGCAGCTGCGCCAACCATAC
ATTCACACCGCAGGATTACTTTAGAAAAGCAGATGACTTGTATCGCAAACATTGGAATGGTTTGTATAATTTATTCGTC
GACGGCGGCATCGTCCCCGGAAAGCCAAACAACACTGGCACATATACGAATCTCGTACAAAATGCGACTGGAAGGAAAC
TTCCACGACTGCGGTGCGAGGGAAACCTACTGAAAGAAGTAGTTATTTGTTACAATAAAACTGCAACTGAAATTATTAA
TTGTCCTTCAGACATAGGGGAGAATTGTGTGGGGTCCATATTAATACCAACAGGAATTTCCCTTTATGGTCCAACCATA
TTGGTATTGTCCTTTAGTTTACCCTTGGGGCTAATTATCTTTAAATATGGTCCAAGCTATATTAAATCTAAGCTAGCT
>Rxanthina_9
ATGGCATTGCTAAGTACAATAGCCGTGCTGCTTGTTTCTGCTTTTTCATCAGGTGCTAAAGCCGCCAATAAGTACGACT
ACTTCCAGTTTGTCCAGCAATGGCCGGCTACATTCTGCTATCATAATCCGGATTGTGTCCCAAATCCCCCGCACAGTAC
TTTGACAATTCATGGCCTTTGGCCAAGTAATTACACGACACCTGAAAAGCCATGCGTTGGCACACCCTTTAATGTTTGC
TGCCCCAAACCATGGTTTGAAAGACTATTTTCTGCCGCAAGCATGGCCCCAGTTAATTGCTCGTTTCACCAACATGCAA
TCTGGCAGAGTGAATATAACAAGCACGGTACCTGCTCGGAGAACAAACTCAAGCAGACGGAGTATTTTAGGACAGCTCA
CTGGGCGTGGGTTCGATTCAATGCATATTTTCTGTTTGCAGACTCGCCTCTCCAAATCCACCCTGGTAATTACTATCTT
AAAGCTGACCTTGAACATGCCATTCGGGCAGTCACTACTAAGGACCCTCTGCGTATGTGCAAGAAGATCAAACCCATGG
GCATTGATACGTGGCTAGAAGTCATCATCTGTCTTGATGTTTTGGGAGTCCAACCGATCCCTTGTCGCCCTCGATTACC
AACTTGCCTCGGTCCAATTATATATTATGGTTAC
>Rxanthina_11
CTTTGCAAATTAACCATGTCAGTCTTCAAAGGAAATTCCGTTCTCCTTCTTCTTGTTTTAGTACTGTTTTCTCTCCAGC
ATGGAACTGATTCACTAGGTAAACCCTTTGAATATTATCAACTCGTGATGCGATGGCCCAAAGCAGTTTGTTACCATAA
ACCTGATGAGCCGTACAGGTGTGTAATGGAAGCAGATCGTGTGCCGTTGAGGTTTACCCTCCACGGCATGTGGGCCTCC
AACTGGATTAATGTGGGAGATGGAATTGATTGTGATGAGGCTGGAACACCATTTCAGATAGATGAGACATTGCAAGAAC
AACTAAAGCAAAGCTGGCCGACGTTACTGGATAACTATCCAACTGATATGAAATTTTGGCAACACGAGTATGATAAGCA
TGGCCAATGTAGCCAAGACACTACTACTCAGACTGAGTACTTCTCACGCGCTCACAAGTTGTGGCAGGAATTGCAGGCT
GTCTACCAAGCATTTGCCGATGAAAAAATCGTACCAGCAGCTGACAAAACATACACCTACACCCAGCTCGAGCAAGCTA
TTGGAAAACGATTCGGCAGTAATTGGAAGGTTGTCATTCTTTGCAGAAAGGTGAATCTCTCACCTGGTGGTCGTGGACC
AAAACAAATTCTACTTCTTTTGCATGAAATCGTATTCTGTTTCGATAACAAAGCAACGGAGAAGAAGAGTTGCAACAGA
AAAACAAATTGTGAGAACGCGAAAACTATAACCTTCATAAAT
>Rxanthina_13
AGAACGATGGCACAAAAGACTTGCAAGCCCAAAGTGGCTTTTGTCGTCACTGCTCTAGCTTTGGTCTTCAGTCTGACGT
CATCATATACACCCTACCAATACCTGTTGTTTGTTCAACAGTGGCCAAAGTCTGTTTGTTATGATATCAACTGCCCTAA
CACTCCCCCTCCAATCATCACGGTACACGGTCTATGGCCATCGAACTATAATGGACCTCGCTTGAAGTGCTCTGGCGGT
ATGAGTCACCAAACAGTACTAAAAGCAAACTCGGAACAACATTCCTGGTCGGATGTGGAAAAGGGAAACCATATGGGTT
TCTGGAAGCAGGAGTGGCAGGCTCATGGACAGTGTTCAGACACCGTCTTTCCACATACCAAATATTTCCAATGGACTCA
CGATATGTGGAACAAAAACGTGATAGGCGAGATCCTTGAAAGGTCGCCCATCATTCCAAGACCCAAAAGGTATAGCTTG
TACGCCATAGAAAATGCCATCAGAGCAAACACTTCGCATGACCCTTATATTCGCCGTGCCCAAAGTTCGGACAAT
>Rxanthina_21

CTTGTCCAAATGTGGCCACCGGGTCATTGTCATTTCCCACCACATTGCACTGCACCGATACCTCGGAACTACTTTAGTG
TGCATGGCTTATGGCCAAGTAATCACAGTGTGCCCAAGATTACATGCAAGAATGCATATGTTCCCTTCTCCGAAACACT
GGTACGGCGCAACAAACCCCTACTGCAGGACCTAAGTAAATATTGGCCAGACTTGGAAAGAGGGAAAGATTTCAGATTC
TGGGAACATGAGTACAGGGATCATGGGAGGTGTATGTATAACATACACAGGGAGCACCAAAATTCCTTGAGGGCAGCTG
CACCAGCCCCTTTCAAAAAAAAAAAAAAAAGTCCCTATTCCGTTCATCTCTCATACAATCAATCACTTTAATGCCCTGCT
TAGAATAACT
>Rxanthina_23
ATGGAAACGGCATTGCTAAGTACAATAGCCGTGCTGCTTATTTTTGCTTTTTCATCAGGTGCTAAAGCCGCGAATCATT
ATGACTATCTTCAGTTTGTTCAACAATGGCCGATAACCTTGTGCTACAAAAATTCAGACTGTATTCCAGGTGCAAGACT
CCCACTGGGCTTCTTTATCCATGGGTTATGGCCGAGCAATTTCTCTGGCCCGAATCAGCCTTGTGTTGGTACTCCGTTC
AACTCTAATATGTTAAATCAACATAGTCTCATGGGGAAGCTGGAAGTCTCATGGCGAAGCTTCACTCGTAGACCTAACA
TGAGCTTCTGGGAGCATGAATACAACCGTCACGGCACTTGTTCGGAGAACAACCTCCCGCAGACGAGATATTTTTCCAA
AGCCCATCGTCTCTGGGAGAGCTACAAAGCACACCGTATGTTCCGTAAATCTAGAGTCATGGGCCACCGGATCGTGCCG
GGATACCCCTACCGTTACCGTGACCTTCGAAAGGCCGTTCGACAAGAAATTGGCGGAAAAAATCCTTCGCTTATTTGCA
AGCACGAGCCTGCAAATAATACTTGGATGCTGCACGAAGTCATCATATGTTTTAATGCCACGCGGTACAACGTCATCGA
TTGTGTTAGAAGATCTAGTTGCTCCGGCGCTGCCAACGGGTATATACACTATCCTTCGCGA
>Rxanthina_29
ATGAAAACGCCAGCAGCAGTCTGTCTTATTTTCTTGCTTTCTGTTATTCTGCTCAAGGGTTCCCACGCAGAGCCATACG
AGTATCTACAATTTGTTTTACAATACTCCAAAGGCTACTGCTACAACACCCAGATATGTTTTGCGACGTTGCCAAAAAT
GTTTACCATCCACGGTGTCTGGCCCGCGAACATTTCCAATCCCCTGGTGAGATGTAAACAGTCAAAGAAACTACACCCG
TTCAACCAAAACATGACAACATCACTGCAAAACGATCTTGGCCAATCTTGGCCCAATGTCGAAAGAGCTAAAACCAATA
TAGGGTTTTGGAAAGAGGAATACGAAAAGCATGGGTCTTGTACTGCACCAGCTATCACACAGAAAGCGTACTTTGAGAG
AGCTCACGAGCTGTGGAAGGAGTATGATCTCTATACAATTTTGGATAAAAAGCAGATCAAGCCGGGGCACTCATATGCA
CTCACTGATTTTGAAGCAGCCGTCAAGTCCAAGATCGGTACC
>Rxanthina_31
GCAGAGCCATACGAATACCTACAGTTTGTCTTGCAATACCCTAGAGGTTACTGTGTTAACGACAAGAAATGCATTCCAC
CGGCGAGCTTGCCAGCCAAATTCACGGTTCATGGAATTTGGCCCACGAATATTTCCGAGCCGATTATCAGCTGTGACAA
GGCACAGAAACTACACTCGTTCAACGGAAACCTGATAACTCCGTCGTTGAAAACCGATCTTCTCCAATCTTGGCCCAGC
GTCCTGACAGATAAAGATGATATGTCGTTTTGGGAACACGAATATAATAAGCATGGGTCCTGTACTGCACCAGCTATCA
CTCAGACAGCGTATTTTGAGAGAGCTCACAAGTTCTGGAAGGAGTATGATCTCTATTCCATTTTGGAACAAAAAAATAT
CAAGCCGGGGCAATCTCGATCGTATTCACTGGCTGATTTTGAAGCAGCCGTCAAGTCCAAGATCGGTACC
>Rminutifolia_4
GTGTTAATCATGGATATGGCATTGCTAAGTACAATAGCCGTGCTGCTTGTTTCTGCTTTTTCATCAGGTGCTAAAGCCG
CTAATAAGTACGACTACTTCCAGTTTGTCCAGCAATGGCCGGCTACATTCTGCTATCAAAATCCGGGTTGTGTTCGGAA
TCCCCCGCGGAATACTTTTACCATTCATGGCCTTTGGCCAAGTAATTACACGACACCTGAAAAGCCATGCGTTGGCACA
CCCTTTAGTCCAGCTGAGATGTTTGCTGCCGAAAACCATGGTTTGAAAGACTATTTTCTGCCGCGAGCATGGCCCCAGT
TAATTGCTCGTTTCACCAACATGCAATTCTGGCGGACCGAATATAACAAGCACGGTACCTGCTCGGAGAACAAACTCAA
GCAGACGGAGTATTTTTGGACAGCTCACTGGGCGTGGGTTCGATTCAATGCATATTTTCTGTTTGCACACTCGCCTCTG
CAAATCCACCCTGGTAATTACTATCATAAAGCTGACCTTGAACAAGCCATTCGGGCAGTCACTACT
>Rminutifolia_17
ATGGCTGGAGTAACAGTGGCAGTCATTGTGCTATTTTTCCTTTCACTTGGTGCGGTCGGACAGCCGGGCGGACATCATG
GCCGACAGCCGGTCCGACATCGGGTCCGACATCAGTATGACCTTCAACTTGTACTGCAATATCCCAGAACACGGAACCG
AGGATCAAATATTCGCGAGCGTTTCACCATTCATGGCCTATGGCTCAGCAGTAAATTCAAGCCTCATACTTATTGTGGA
CGACGCCTTGGTGACATTTCAAACCTGCTGACCAAAGTCCTCAAAAACGAACTCAGTTACTCTTGGCCCAATGTTGTTT
ACGGTGACTCTCGGAATTTGAATTTTTGGACCGACGAATACAATAAGCACGGCAGCTGCGCCGTTTACTGGAAAATATT
CAGAACAGTGGAGGAGTACTTTCGAAAAGCAGATGACTTGTATTTGCAAACAAGCAATTTATATAAAGTATTCGGCAAC
GGCATCCACCCGGGAAACAAAGTCGACGTTAGCCACTATAGGAGTCTCGTAAAAACAGAGACTGGGAAAATTCCACGAC
TGCAGTGCATCGATGGAAGTTTTGGAACCCGACTGCTGGAAGTAGCTATTTGTTACAAAAAACAGAGTACTTTATTCAA
AGTGGTTGATTGTCCTAAACTTGGGAATTGTACGGGGCTCATATCATATCCAACAGAAATAGCCCTT
>Rminutifolia_21
ATGGGGATCATCCTACTCGTCCTGCTTCTCTCTGCGTGGGAGGCAGCTGATGCAACCAAACCCTACTACTACTTCCAGT
TTGTGCAGCAATGGCCGGCTGAAATTTGTTTTCGCAATCCAGTTTTTCGCCATGGCCATCCAGTTCGTATCCAACATTT
CCCAAAGTTTTTTACAGTGCACGGCCTTTGGCCAACTAAAAACTCACGGACTGCTCGGACTCCTCCACCGGCTTGTGTT
GGCACACCATTTGATGAAGCTGAGATGTCCAAAAACCGTGACTTGATGAAAGATCTTCGAAAAGCATGGCCCCAATTGA
AAGCTCATACTACTAATATGGAGAAAGCTGATGCTGCAAATATGCACTTCTGGAAAGAAGAGTATAATAAGCACGGAAT
TTTCTCGGAGAACAAGCTGGACCAGACGCATTATTTTCAGGAAGCTTACCGGATGTGGTCTCTGTACAGAGCAGATCAG
CTGTTTGAAAAATCATCACCGGCAATCTACCCTGGAAGTTCCTATACTCTCACTGCCCTTGAAAATGCCATTTGGGAAG
GGACTGGGGGCCGGCCTCTTCTTAGCTGCAGGCCCAGGAAGAGAAAAGTAGGTGCCAAATATGAAAATGTTTGGTTTTT

GGAGGAGGTCATCATTTGTTTTGATTATCTGAGACAGAACGTCATCGATTGTGTTCGACGGACAAATTGCGGTGTAACA
ATATATTATCGTCGTTAC
>Rminutifolia_23
TCTATTTCTAGTACTTGCGTGGCGGAGGGATTAAATTTCCTAACAATTATGATATCAACGTACTGCCCTAACACTCCCC
CTCCAATCATCACGGTACACGGTCTATGGCCATCGAACTATAATGGACCTCGCTTGAAGTGCTCTGGCGGTATGAGTCA
CCAAACATTACTAAAAGCAAACTTGGAACAACATTCCTGGCCGGATGTGGAAAAAGGAAACCATATGGGTTTCTGGAAG
CATGAGTGGGCGGAGTGGCAGGCTCATGGACAGTGTTCGGACACCGTCTTTCCAGTTTCCACATACAAAATATTTCAAT
GGTGTCACGATATGTGGAACAAAAACGTGATAGGCGAGATCCTTGAAAGGTCGCCCATCATCCCAAGACCCAAAACGTA
TAGCCTGTACGCCATAGAAAATGCCATCAGAGCAAACACTTCGCATCACCCTTATATTCGCTGTGCCCAAACTCCAAAG
TTCGAAGAT
>Rminutifolia_27
TGGCCAGGCTCATACTGTGCAGCAGCAAAGCAGGGTTGTTGCTACCCAAAGAGTATTAGAAAACATCCCAGTTTCACAA
TTGGTGGCATATGGCCTTATACTTTTTCTGGGGATAGACCAACCTACTGCAAATCTAAGACCCCTTTTAGTTTATCCAA
GAGTTTGGAAAGGAACTGGCCATCACTATCCTGCCCAAGCCGTACTAGTAGCAGTAAGAAACTATGGATGCAAGAATGG
CAGAAATATGGGACTTGTGCAGAATCACTCTTTGGAGGTCAATACCAATACTTTCATGCAGGTCTCAATCTGAGGAAGA
AAATAGACATGCTCCAGATACTCAGCAATGCAGGTCTGATCACTACTATAATCCAACTTTCGGATTTCAGCTCAAATAA
ATTAGTGGAACTCAAAACTTTTCGCTTAATA
>Rminutifolia_34
ATGTCTCTAGGTTCATATGATCATTTTAAATTTGTACAACAATGGCCACCGGCCGTCTGTGACACGTCTGGCTGCAACA
GATCGGGGTTATCACGCTTCACGATCCATGGCCTTTGGCCAAATAATAAAACGTATGTGAAAAATCAACCGACTTGTCT
TACCAATCAGGCCAACTCATTTAAGGCTAAGATACTCACTAGTTCGAATTTGGTAAGCAAGCTGAGCACCTCTTGGCCC
GATGTGAAAAATGCAAATGATAAATTTTTTTGGAAGAAACAATGGGACAAGCATGGCACATGTTCCTTGCAGACATACG
ACCAAGCACAATACTTCGAGCGGTCATACAACATGTGGAAAGAAACCAACCTGACAAACACCTTGGATAGCCTCATCAA
ACAGACACCGAGACAACAGAACGTCACAGATATAGAACAACTCATTCAAGGAGTAACTGCAACTGAAAAGAAACCCCTC
CTCCGTTGTGAACAATCTCATAGTAATGCCAGTAAATCTCTCTTGAAAGAGATTGTCATTTGCTATGCACACAACGGGA
TTACTGTGATCGATTGCGTCAGTCAACCAAAATCCACCTTCGAATGCTATGACGGATTTTGGTTTCCT
>Rminutifolia_35
ATGGCTGCGACAGTGTTTCTGTTGTTCTTACTTCCATTTGCTGCTGAGGGTCTAATCACAAATTCGAGCCAGGGGCAGT
ACCGCTCCAGTTTGAGCTACCACAAGGGTTGGAAGAGCATTCCCGGGCAGGCCGTGGGATGGCCGGCGTACCAATACCT
GCAGTTTGTACTACAAAATCCTAATTCTTTGGATCCAAAAACACCAGTGCAGAGCTATTTTACCATACATGGCCTTTGG
CCTTCCAATATCAGCAATAGTCATCCCCATGCTGTTGTTTCCGTTGAAAACTGTAGTTACAGTCCTCGCAGTCCTCATG
AGTATTGGTTGGATAACATCCTCGATCATATTTGGCCAGATGCTCATAATCCCTTTTCAAAGGAAATGCAGCTGCACAA
CAATGAGCGTTTTTGGGAAAACGAATACAACAAGCACGGCAGTTGCACTTGCACAAATCGAAATTTCTTGACACCGAAG
GATTACTTTAAAAAAGCTGTTGAATTGTGGAACAAGCTTAATATTCGTTACTTTCCTCACATACAACCGGGAAGCAGAG
TCCCATATCGTGACTATTTACGGCTCGTCAAACAAGCGCTTGATAAACACCAAACAGTAAATCCAATACTGTATTGCCA
CCCAATGAACAGACAACGTGAACAACAACTCAAGGAAGTAGTTATTTGTCTGGATAAAAATGCGAGTATGCTGAAGAAT
TGTACCGCCGTCAGAGGGCCATTGTGCAAGAGTTGGGGGAAGATATACTCGAGC
>Rminutifolia_38
AGATTCACCATTCATGGGCTATGGCCTACGAGCAAATACAATACTGCGATTACATATTGTAATACTCGAAATTATGCAG
CTGCTGATCTTCGCAAACAGCTGCGGCGAACCGATCTCCCAGATGAACTCCTTCAGTCTTGGCCCAATGTAGTGACTCC
AAACAATCCGAATGCAGTTTGGTCCTTCTGGAAATACCAATACAACAAGCACGGCAGCTGCGCCTACTGGAGATTCCAA
AGACTGGAGGCTTACTTTAGAAAAGCAGATGACTTGTATCGCAGTGATAACGTACAACATTTGTATGATTCATTCACCA
GCAATCGCCAAGTCTCTGCTATCAGATATATGAATGACGTAAAAAATTATGGAGGGAAATTTCCAGGACTGCGGTGCAA
TGGAACGGGATTCCTGATAGAAGTGATTATCTGTTACACAAAAAAAGCAACTAAAATGATTGATTGCCCTAAACGGGGG
AATTGTACGAGGCTAATTGAATATCCAATG
>Rarvensis_2
GTGGGGCAGCCGTATTACCAGTACCTGCAGTTTGTAGTACAAAATCCTAAATCTTTGGATCCATGCTCACCAGTACGGA
GCTATTTTACCATACATGGCCTTTGGCCTTACAATATCAGCAATAATAAACACCATGATGTTGTTTCCATTGAAAACTG
TAGTTACTGTCCTCCCAGACCTCATCAGTATTGGCTGCCTACGAATTTGGAAAACAGCCTCGATATAGTTTGGCCGGAT
GCTCATAGTGTTCGACCCAATCATCAGCGTTTTCGGGAAAACGAATACAAAAAACACGGCAGCTGCACAAACCGAATTT
TCCCGAGACCGGAGGATTACTTTGAAAAAGCTCTTCACTGGTATGATCTGATAATCGAAAGACTGTTAGATCATCATCA
TCGTAAACTATATCCCAACATCCAACATCTGGTATCCCAACCAGGAAACCGAGTCCGCACTGGTCTGCTTTTAAATGAC
GTCAGACAAGCGATTAAAGACGAGCGAAGGATTACAGCCGAGCCACTACTGTTGTGCAACGGAGGGAAAGGCCAACCAC
AACAACTCAAGGAAATAGTTATTTGTCTCAATCAACATGCAACTGAAGCGACTGATTGTACCAAAAGAAGAGGGTTGTG
TAGGGGGGGAATATACCTTCAGCCT
>Rarvensis_4
ATGAAAATGATTCCTTTTCTTGCTCTCCTACTTTTCCTATTCCCCTCAACTGTAAGGGCCGAAATGAAATATTATGACG
GTCAAACGCTTGTGTTACAAAATTCAGATGAGTTATGGACCATTCACGGCCTTTGGGCCTACCGACTTACTGGAAAAGA

ACCTAAATTCTACTATCAGCCAGTTACCGATTGTTCACCATTTAAAAAATTTAATTTAAATGAGGTAATTGGCAACGAA
CGAGGGACACTCGCTAGGGTTTGGCCTAGTAACAATCCGAATACGAGGATGTCCTATAAATTCTGGCAGTATGAATATG
AAAAACACGGTTCATGTACCGCAGATATATTACCAAGCTGTAGAGATTATTTAATGAAAGCAGTTACACTGTGGAATAT
ACTCCAGTTAGACAATTTGCTGGGTCCTAAAGGCAAGTATAAGCCAAACACTTTATTTCAAGCCCAGGACTTGCTGTAC
GACATGGAAGTGGAGTATAAGGTCCGACCTCTGTTGAAGTGTAATAATCAGGGGGAACTACTGGAAATTTGGTTTTGTT
ATACAGAAGTATGGAAAAGCATATTTTGCCAGCCCACGACAGACTCTTGTACGGGTCATATTAAATATGTTAGGGCA
>Rarvensis_10
GTCCCACGGATGATTAATACGAAAGCAAAAGAATCTCGTTTCACTCTTCACGGCCTTTGGCCCTTCAACGCTACACAAT
TAGATCCAAACAATATTAATTCTGATTTCCAGTATAGACCTATCATAGAGTGTGCCCAGTTACCTACTGTCTTACTGTA
TAAACTTGCAAAATCTTGGAAAAGTTATATGCGTAGCTATCCGAATGGGGATTTTTGGAAAGATGAATACGACAAGCAT
GGAACATGCACCGTTGAAAAATTTCGTACACCTGCCGAGTACTTCCAGAAGGCAAATGACTTGTGGGAAGCCAACCCAA
TAGAGGATTGGTTTCGGCGTGATGGTCGCTTCACAGATACAGATGTCCAGCTCGCTGAGCTGAGAGGCGCGATCATGAA
CAAGTTCGGTTCTGTGCCATGGTTTCAGTGTGAGGACAAATTTGTGCGGCAGATAGGCTTATGTTTTGATGAGGTTGGA
GGGCTGGGTGCGAATTGTCCTAAAGAATATCAAAAGAAGCAATGTCCCCCTACTGTACAGTATAGATTG
>Rarvensis_19
TGTCTTATTTTCTTGCTTTCTGTTATTCTGCTCAAGGGTTCCCACGCAGAGCCATACGAGTATCTACAATTTGTTTTGC
AATACTCCAAAGGCTACTGCTACAACACCCCGATATGTTTTGCGAGGTTGCCAAAAATGTTTACCATACACGGTGTCTG
GCCCGCGAACATTTCCAATCCCCTGGTGAGATGTAAACAGTCAAAGAAATTACACCAGTTCAAGCAAAACGTGATGACA
ACATCACTGCAAAACGATCTTGGCCAATCTTGGCCCAATGTCGAAAGAGCTAAAACCAATATAGGGTTTTGGAAAGAGG
AATACGAAAAGCATGGGTCTTGTACTGCACCAGCTATCACACAGAAAGCGTACTTTGAGAGAGCTCACGAGCTGTGGAA
GGAGTATGATCTCTATACAATTTTGGATAAAAAGCAGATCAAGCCGGGGCACTCATATGCACTCACTGATTTTGAAGCA
GCCGTCAAGTCCAAGATCGGTACCRATACAATACCTCTCATCCTGTGCAAAGAGGACACACTAACTACTGGGAGCAGCA
CAACTGGATTAATTCTGAGGGAAATAGTTATCTGTTTCGATCACCAGGGAACAAATCTGGTCAATTGTACCCGGCCGAC
AGATTGCAAGCGGCAGAAGAGCACAACTAAGGACAACATGATATACTACGTACCT
>Rarvensis_25
ATGGCATTGCTAAGTACAATAGCCGTGCTGCTTGTTTCTGCTTTTTCATCAGGTGCTAAAGCCGCCAATAAGTACGACT
ACTTCCAGTTTGTCCAGCAATGGCCGGCTACATTCTGCTATCAAAATTCGGCTTGTGTCCGGAATCCCCCGCGGAATAC
TTTTACCATCCACGGCCTCTGGCCAAGTAATTACACGGCACCTGAAAAGCCATGCGTTGGCACACCCTTTAGTCCAGCT
GAGATGTTTGCTGCCCCAAACCATGGTTTGAAAGACTATTTTCTGCCGCAAGCATGGCCCCAGTTAATTGCTCGTTTCA
CCGACATGCAATTCTGGCAGACCGAATATAACAAGCACGGTACGTGCTCGGAGAACAAACTCAAGCTGACGGAGTATTT
TAGAACAGTTCACTGGGCGTGGGTTCGATTCAATGCATATTTTCTGTTTGCACACTCGCCTCTCCAAATCCACCCTGGT
AATTACTATTTTAAAGCTGACCTTGAACAAGCCATTCGGGCAGTCACTACTAAGGACCCTCTGCTT
>Rarvensis_33
ATGAAGTCGATTTTGATTTCATTTGTCACAATTTGTATCAGTTTTCAACTTCTCCTATCAGCGCCAAATCCACCTCAAG
CCCCACCTCCTGATTCCTTCCTTTTGGTCATTGTGTGGCCCAATACCTTCTGTCTTTTTGAGTCATGTCAGCTGCATCC
ACAATCTTTTACACTACATGGATTGTGGCCACAGGCGAATGGGAAATCCTTGGTGGACTGTGCTGGTTCAGAAATGGAT
GATAGCACCTTAAATGGCAAGAAAGATGATTTGAACAAGTATTGGCCAGACTTGACGCATTCCAAGTTTGAAGAGAGTA
AAAGCTTTTGGATACATGAGTGGGAAAAACATGGTCGTTGCTCTGCAAAGAGTCCTGCTAATTACTTGAGCCTGGTGTT
TGATCTGATGAAAAAACATGATGTGGAACAGATATTTAAAAACAATGGAATTTTACCAAGCAAGAACGGGTATCCGATG
ACACAGTTGGGACAGGCAATATTTGATGACACTAAATTGTGGACAAGGATTAAATGCCAATCGAACGACGGACACCAGT
TTCTATTTCAAATATATTTCTGCTTAACAGCTCAAGGCCAATTCAGGAATTGTTCTAAAAGCGGTGCGGACTATACAGG
GTGTACCAAAAGGCTGCCAGTCACCTTCCCCTTACCTTCGCCCGGTCTCATAGTAAAAAGCAAGAAG
>Rarvensis_39
CTTTGGTCTTCAGTCCGACGTCATCATATACAATACCTATTGTTTGTTCAACGGTGGCCAAAGTCTGTTTGTTATGATA
TCAACTGCCCTAACACTCCCCCTCCAAACATCACGGTACACGGTCTATGGCCATCGAACTATAATGGACCTCGCTTGAA
GTGCTCTGGCGTTGTGACATTTAGTGAAACACTGGTAACACATTATTATATGAGTCACCAAACATTACTAAAAGCAATC
TTGGAACAACATTCCTGGCCGGATGTGGAAAAGGGAAACCATATGGGTGTCTGGAAGCGTGAGTGGCAGGCTCATGGAC
AGTGTTCAGACGCCGTCTTTCCACATACCAAATATTTCCAATGGTGTCACGATATGTGGAAGAAAAACGTGATAGGCGA
GATCCTTGAAAGG
>Rmajalis_1
TTGCTGCTCTTCACTCTTCCCTCAGTATATTCTCAGAATGTGGAGCACTCGAGATTGGCTCTAACCAATCCAAAGGCTT
ATTGCTCATTAAGGGCTTGTGTTGCACCTATCCCACAGTCTTTTACTCTTCACAGACTATGGCATCATAATTTTGTCAA
GATTGGTGGAATAACCCCCTTGCGGTCAATTCATCTAAGAGGTGTTTCAACACAAGATCTTCTCAACTATTGGCCAGAC
CTCTCTAGAGATCGAGCAATTTGGAAGCATGAATGGGAATCCCACGGCGCACATATA
>Rmajalis_11
ATGAGTATTTTTACATTGTTTCTCCCCCCAAACTCCACAGGCGGATACTATGAATATTTCAAGATGGTGGAACAGTGGC
CGTTAGGATATTGCACAGGTCGACCAGATCCTTGCAACAAAACTCCACCAAACATGTTCACCATCCATGGTTTTTGGCC
CAGTAATTATTCAACAAATCCAGCAGATACATGCAAAGGACTTCGTTCAACGAGACTATGATGCGTAAAAATCAAACA

TTGGTATCCGCACTACAGAGAGTATGGCCAGACCTGGCGGACGGAGATGATTTTGGGTTCTGGAGACGTCAGTGGGACA
AACATGGGAAATGTTCGGAGCAAACATTTCAGCAAATGCAATATTTCGAGCGTGCAAATGTTATTTGGAACAAACCGAA
TATGACTTTGATCTTTAAAAATAATGGAATCCTAGCAGGGGGGGCAAAATATAACTCCACAGACTTGGTATCACGCATT
GAAAAGGTGCTTCGGACCACGCCCCTACTTCATTGCAAATGGGTAACGGATCCTAAT
>Rmajalis_20
TTTTTTTTTTTCAACACAATAATGTCTCTAGGTTCATATGATCATTTTAAATTTGTACAACAATGGCCACCGGCCGTCT
GTGACACGTCTGGCTGCAACAGATCGGGGTTATCACGCTTCACGATCCATGGCCTTTGGCCAAATAATAAAACGTATGT
GAAAAATCAACCGACTTGTCTTACCAATCAGACCAACTCATTTAAGGCTGCGATACTCACTAATTCGAATTTGGTAAGC
AAGCTGAGCACCTCTTGGCCTGATGTGAAAAATGCAAATGATAAATTTTTTTGGAAGAAACAATGGGACAAGCATGGCA
CATGTTCCTTGCAGACATACGACCAAGCACAATACTTCGAGCGGTCATACAACATGTGGAAAGAAACCAACCTGACAAA
CACCTTGGATAGCCTTATCAAACAGACACCGAGACAACAGAACGTCACAGATATAGAACAACTCATTCAAGGAGTAACT
GCAACTGAAAAGAAACCCCTCCTCCGTTGTGAACAATCTCATAGTAATGCCAGTAAGTCTCTCTTGAAAGAGATTGTCA
TTTGCTATGCACACAACGGGATTACTGTGATCGATTGCGTCAGTCAACCAAAATCCACCTTCGAATGCTATGACGGATT
TTGGTTTCCT
>MDP0000267606AT2Lineage
ATGATTCTCTCTGCTCAATTCGCCTCTGCAGTAGCACTGATTGCGGTGGGCGCTTCTCTGTGCCTGATCGATGCCAAGC
AAGCCGGAATCGGATTCGAAATCGGAAGCAGAGGAGGAGGAGGGCAGAGGGAGTTCGATTACTTCAACTTGGCCCTGCA
ATGGCCTGGCACTTTCTGTCAGCGCACCCGCCATTGTTGCTCCTCCAATGCTTGCTGCCGCGGCTCAAATGCTCCAACC
ATGTTTACAATCCATGGATTGTGGCCTGACTACAATGATGGAACTTGGCCTGCCTGTTGCACACGGAAAACCTTTGATG
ATAAGGAGATCTCAACATTGCACGATGCTTTAGAGAAATACTGGCCATCTTTAAGCTGTGGTAAACCATCATCCTGCCA
TGGTGGAAAAGGATCATTTTGGGGTCATGAGAAGCACACTTGCTCCTCTCCAGTAGTTGGAGATGAATACAATTACTTT
TTGACAAATGTCTATTTTAAGTACAATGTCACTCAAATCCTGAATGAAGCAGGATATGTACCATCCAATACTGAAAAAT
ATCCTCTTGGAGGCATTGTTTCTGCTATTCAGAATGCTTTCCGGGCAACCCCGAGGTTGGTTTGCAAAAAAGGGGCCTT
GGAGGAACTTCATCTATGCTTCTACAAGGATTTCCAGCCTCGGGATTGTCTGGTTGGATCTGGCAGTCTAAGTGACAAG
TTAGCTTCAAGTAGCTCATGTCCCAGTTTTGTCAGCATACCAGCATATGCATCATTGGATTCCTGCAGCTCAACTCTTA
ACCTGCAGAATGTCAATCTGCCC
>M_domestica_MDP0000160706_Malus_S_RNase_lineage_2
ATGGAGTCGAAGTTAGTGCAAGTCATATTTTTCTTCTTGTTTGTCACAGTTTCATCCGCTTACACACCATTCCAATACT
TTATGCATGTTCAGTTCTGGCCAGCTGCTGAATGCCAAGCTACAGGGGGGAATAAATGCCACATCATGCAACCATCTCA
TCTTCAGTTCACTATTCATGGTCTCTGGCCTGCGAATAAATCATCCTCTTCATCCCTTGGCTGCAAGGGGGATGCCTAT
AATGTGAATGGGATGAATGATACACTGAAAAAGGAGCTACTCAGTTCGTGGTGGGATTGGAGAAAAGGGGAGCATGTCG
AATTCTGGCAACGAGAGTATGATAAGCACGGAAAGTGCTCCGACAATGTGTTCCTGAAGACCGAGTACTTCCGGAAGAC
CCTAGCGATGTACCATGATTTTGATATAGCTCAAATCCTGCAGAAGGCTAATATTGTACCCCAACTACTTCAGCCCAAG
ACGTCGTTGTACAAATTGTACTCCATCGATCAGATAACAAAGGCCATTGAGTCCAAAACCGGCGGACATTCCGTCAATA
TCAGATGCTATCAACAAAACCAGAAGAAGAGCAACAATGGAAGTAAACCATTAATATATTTGGCCCAAGTCGCCCTCTG
CTATAATAGATCTGGCAATAGCAGAACTAACTGTGATCCAGGCGAAACAACCAGCTGCAACTTTAATGAATCAGAACCG
AAAACTGTACCAAGAATAGCCCACATAAAGCTCTGG
>M_domestica_MDP0000250548A_S_RNase_lineage_1
ATGCACTTCTTCAAGATTGCTCTCCTTGTCCTCACTCTTTACTTCATCACCTCTCTCGGAAAGATCAATGCTGCCACAC
CATATGACATCTTCCAGTTCGTCCAGCAATCCCCATTGGCCTTCTGCTACGGAACCGATATCTGCGGAGACCAACCAGT
ACTGCCCCGTACCTTTACGACGCACGGCCTCTGGCCAAGCAGCAGCACCAATCCAGCTGCTCCTTTAACCTGCGCTGGT
ACCGCATTCAGCCGTAGTGAGATGAATGATCCCGACAATCAATATCTGCAACTACTTTTGTCCTATTCCTGGCCCAACT
TCAACATTAGACAAACAAATATGGATTTCTGGGAGTATGAGTACAACAAGCACGGCAGGTGCTCGGACAATACATTTTC
CCAGACACAATACTTCCGTGAAGCTTATAGATTGTGGTCTACCTACAATGCACTCCATCTATTTTCTCAAACAACTTGG
ATTGTACCAGGCTATCCATATCGTTATATCGACCTTGAATTGGCCATTCGACGGACTATAGGAGGGAAAGCACCTCTTC
TTATGTGCAAGTATAACGGGGTGAGCCCGTATCTGGTGGAAGTCGTCATCTGCTTTGACTACACTGCAGCGAATCCGGT
CGATTGTGTCAGGACAACAAATTGTGGAAATCCTAATGCATTGGGCAAATTTTCATCTAATATATGGTGGAATAGGCAG
>M_domestica_S2_RNase_MDP0000345854
ATGGGGACTACGCGGATGGTATATATAGTTACGATGTTATTTTCATTAATTGTATTAATATTGTCTTCGTCTACGGTGG
GATACGATTATTTTCAATTTACGCAGCAATATCAGCCGGCTGCCTGCAACTCTAATCCTACTCCTTGTAAAGATCCTCC
TGACAAGTTGTTTACGGTTCACGGTTTGTGGCCTTCAAACATGAATCGAAGTGAATTATTTAATTGCAGTAGTTCAAAC
GTGACTTATGCGAAGATACAAAATATCCGAACCCAGTTGGAAATGATTTGGCCAAACGTATTCAATCGAAAAAATCATT
TAGGCTTCTGGAATAGAGAGTGGAACAAACACGGCGCCTGTGGGTATCCCACAATACGGAACGACTTGCATTACTTTCA
AACAGTAATCAAAATGTACATAACCCAGAAACAAAACGTCTCTGATATCCTCTCAAAGGCCAAGATTGAACCGGATGGA
AATATCAGGACACAGAAGGAAATTGTAGATGCCATAAGAAAAGGTATCCATGGTAAGGAACCAAAACTCAAGTGCCAAA
AGAATACTCAGATGACTGAATTGGTTGAGGTCACTCTTTGCAGCGATGGCAACTTAAAGCAGTTCATAGATTGCCCCCA
CCATTTTCCAAATGGATCACGACATAACTGCCCCACCAATCATATTCTG
>M_domestica_S3_RNase_MDP0000266136

ATGGGGATTACAGGGATGATATATATGGTTACAATGGTATTTTCATTAATTGTATTAATATTGTCTTCGTCCGCGGTGA
AATTCGATTATTTTCAATTTACGCAGCAATATCAGCCGGCTGTCTGCAGCTCTAATCCTACTCCTTGTAAGGATCCTCC
TGACAAGTTGTTTACGGTTCATGGTTTGTGGCCTTCAAATGTTAATGGAAGTGACCCCAAGAAATGCAAAACTACAATC
TTGAACCCTCAAACGATAACAAATCTTACAGCCCAGCTGGAAATTATTTGGCCAAACGTACTCAATCGAAAGGCTCATG
CACGCTTCTGGCGTAAACAGTGGCGTAAACATGGCACCTGTGGGTACCCCACAATAGCGGACGACATGCATTACTTTAG
CACAGTAATCGAAATGTACATAACCAAGAAACAAAATGTCTCTGAAATCCTCTCAAAGGCGAAAATTAAACCGGAGAAG
AAATTCAGGACACGGGACGACATTGTGAATGCCATAAGCCAAAGTATCGACTATAAGAAACCAAAACTCAAGTGCAAGA
ACAATAATCAGATAACTGAATTGGTTGAGGTCGGTCTTTGCAGCGATAACAACTTAACGCAGTTCATAGATTGCCCCCG
CCCATTTCCACAAGGATCACCATTTTTCTGCCCCACCAATAATATTCAGTAT
>P_persica_ppa024151m_S_RNase_lineage_1
ATGTTGTTTCTGTTTCTTGCTTTCTTTTATGTCAACAACATTCCATGTGACTGCAATGCTCCTGGACAGTATGACTACT
TCCAGTTTGTTCAGGAGTCTCCTTTTCTATTTTGCAAAAATATTAATGGAAAGGGATGCAACATCCTACCTCTGCCACG
GATATTTACTATTCATGGCTTGTGGCCAAGCAACTTCACACATCGTCATAAACCTTGCGTTGGTGCACAATTTAGTAGA
GATATAAATGAAGCCTCCAATGATGAATTGCGAGCTGATCTGGAGTTGTCATGGCGAAGCTTCATCAGTGGGCGGTCAA
ATATGGACTTCTGGGAATATGAATACAACAAACACGGCAAGTGCTCCGACGACAAGTTTTCGCAGACGCAATACTTCGA
CCGTGCCAGGAGTTTGTGGAAGCAATACAAACCTCATGCTCTATTTTCTAATCGCTCGCTTGAACCAGGCAAGTCATAT
AGTTTTACTGGCCTTGAACAAGCCATCCGATCATTTATAGGCGGGAGCAGACCTCTTCTCCTTTGCGAGCAATCTAAGA
GGAAGAAGAACAAGAAGAAGACAAAA
>P_persica_S2_RNase_ppa018459m
ATGGGGATGTTGAAATCGTCACTCGCTTTCCTTGTTCTTGTTTTTGCTTTCTTCTTTTGTTACGTTATGAGCAGCGGAT
CTTATGACTATTTTCAATTTGTGCAACAATGGCCACCGACTAACTGCAGAGTTCGCGTCAAGCGACCTTGCTCCAATCC
CCGGCCATTACAATATTTCACCATCCATGGCCTATGGCCAAGCAATTATTCAAACCCAAAGATGCCCAGTAATTGCACA
GGGTCGCAATTTAAGAAACAGAATTTGTACCCTTATATGCAATCCAAACTGAAGATATCTTGGCCGGACGTGGAAAGTG
GGAATGATACAAAATTTTGGGAAGGCGAATGGAATAAACATGGTACATGTTCCGAACGAACACTTAACCTAATGCAATA
CTTCCAGCGATCCCACGCAATGTGGAAATCACACAATATTACAGAGATCCTTAAAAACGCTTCAATCGTACCACATCCG
ACAAAAACATGGAAGTACTCGGACATAGAATCACCCATTAAAAGAGCAACTAAAAGAACACCCGTCCTTCGTTGCAAAC
GTGATCCAGTACAGGCGAATACTCAGTTGTTACATGAAGTGGTATTTTGTTATGAATATGATGCGCTAAAGCTGATTGA
CTGTAATCGAACAGATTGCTGGAATAACGTTGACATTAAGTTTCAA
>P_persica_ppa011133m_Prunus_PA1
ATGCCATTAAGATATGAGATATTGGCAGGGGCTTCTCTGCAAGCTATTACCACTCATGGGCAACCGTATGACTACCTAC
AATATGTACTACAATGGCAGAAGACGAAATGCTTGAAGGTCGGGAAACGGTGCACTCCATATGAGAAGTTCACTACTCA
CGGCCTCTGGCCAACCAACCTCTCCAAGATATTGACATGCAAATCAGCTTCAAAATTTAGCAGCACTATGCTGCAAAAT
AATGCTTCATTGCTATCGAAGATGAAAAAGTCCTGGCCAAATCTGGAGCAGAGAGTTGCCCAAGGAAAAGACAACGATA
TGTGGTTTTGGGCGATGGAGTACGAAAAACACGGTATATGTGCCAAGTTTTCTAGCCAGAATACTTACTTGTCAAAAGC
ATGTGATTTGTGGGAAGAAAACAAGATTAAGGAGATTTTTGAGCACCACAAAATCTTTCCAACAAACGCGACGTCATAT
AAAGATGTTTTGCTTATGAATGCTATTGAAATGGAAACTCGCAGTTCTCCTCTCCTTCTTTGCCATAGAGTCAACGGCG
ATTATTTGTTGTGGGAGGTTGTTCTTTGCTATGACGACACGGCTAAAAAACGGATGAATTGTTCTGATCAAAGTGCAAG
ACAAACAAATTGCGGAACGGACATCTATTATAAA
>P_mume_S_RNase_scaffold241_33
ATGGGGATGCTGAAATCGTCACTCGCTTTCCTTGTTCTTGCTTTTGCTTTCTTCTTCCTATTCATCACAATAATTTTGG
CAGGATCTTACGACTATTTTCAATTTGTGCAACAATGGCCACCGACCAACTGCAGAGTTCGCATCAAGCGACCTTGCTC
CAACCCCCGGCCATTACAATATTTCACCATCCATGGCTTATGGCCCAGTAATTATTCAAACCCAACGGTGCCCAGTAAT
TGTAATGGGTCAAAATTTGACGCAAGGAAAGTGTCACCTCAACTACGAAACAAACTGACGAAATCTTGGCCGGACGTGG
AAAGTGGCAATGATACAAAATTTTGGGAAGGCGAATGGAACAAACATGGTACGTGTTCCGAGCAGACACTTCACCAAAT
GCAGTATTTCGAGATATCCCACGACATGTGGCTGTCCCACAATATAACACATATCCTTAAAAACGCTACAATCGTACCA
AGTGCCACACAAACGTGGAAGTACTCGGACATAGTAACACCCATTAAAATAGCAACTAAAAGAACACCCCTCCTTCGTT
GCAAACCTGATCCAGCACAAAATAAGAGCGGACCGAAGACTCAGTTGTTACATGAAGTGGTACTTTGTTATGGATATAA
TACAAAAAAGCAGATTGACTGTAATCGAACAGCAGGATGCTGGAATAATGTTGACATCAAGTTTCAA
>P_mume_scaffold442_35_S_RNase_lineage_1
ATGGTACTTGACCACCAATTGGCTCGGCTTTCAGTCCCGTTCACATGCTGCAATGCCGCTGGACAGTATGACTACTTTC
AGTTTGTTCAGGAGTCTCCTTTTGTATTTTGCAAAAATATTACTGGAAAGGGATGCAACATCCTACCTCTGCCACGGAT
ATTTACCATTCATGGCTTGTGGCCAAGCAACTTCAAACATCGTCATAAACCTTGCGTTGGTGCACAATTTAGTAGAGAT
ATGAATGAAGCCTCCAATGATGAATTGCGAGCTGATCTGGAGTTGTCATGGCGAAGCTTCATCAGTGGGCGGTCAAATA
TGGACTTCTGGGAATATGAATACAACAAACACGGCAAGTGCTCCGACCACATATTTTCGCAGACGCAATACTTCGACCG
TGCCAGGAGTTTGTGGAAGCAATACAAAGCTCATACTCTATTTTCTAATCGCTCGGTTGAACCAGGCAAGTCATATCGT
TTTACTGACCTTGAACAAGCCATCCGATCATTTATAGGCGGGAGAAGACCTCTTCTCCTTTGCATGCAATCTGAGTGGA
AGAAGAAGACAAAACAATATTTGCTGGAAGTCGTAATTTGCTTTGATGACATAGCAATTAATCCAGTGAATTGTCCCAA
GAGGATGTCAAAAGAATGTCTTCCACAATCCCCTGTATGGTATATGCTA

```
>M_domestica_SH_RNase_AB032247
ATGGGGACCGGGATGATATATATGGTTATGATGGTATTTTCACTAATTTTATTAATATTGCCTTCGTCCACGGTGGGAT
TCGATTATTATCAATTTACGCAGCAATATCAGCCGGCTGTCTGCAACTCTAATCCAACTCCTTGTAAGGATCCTACTGA
CAAGTTGTTTACTGTTCACGGTTTGTGGCCTTCAAACTCGAATGGAAATGACCCAAAATATTGCAACGCACAGCAATAT
CAGACGATGAAAATACTCGAACCCCAGTTGGTAATTATTTGGCCGAACGTACTCAATCGAAACGATCATGAAGGCTTCT
GGCGTAAACAGTGGGAGAAACATGGCTCCTGTGCGTCTTCCCCAATACAGAACCAGAAGCATTACTTTGATACAGTAAT
CAAAATGTACACAACCCAGAAACAAAACATCTCTGAAATCCTCTCAAAGGCGAATATAAAACCGGGTAGGAAAAACAGG
ACACTGGTGGATATTGAAAATGCCATACGTAATGTTATCAACAATATGACACCACAATTCAAGTGCCAAAAGAATACTA
GGACATCATTGACTGAATTGGTTGAGGTCGGTCTTTGCAGCGATAGCAACTTAACGCAGTTCATAAATTGCCCCCGCCC
ATTTCCACGAGGATCACGGTATTTCTGCCCCACCAATATTCAGTAT
>P_pyrifolia_S9_RNase_AB104909
ATGGGGATTACAGGGATGATATATATGGTTACAATGGTATTTTCATTAATTGTATTAATATCGTCTTCGCCCGTGATGA
AATTCGATTATTTTCAATTTACGCAGCAATATCAGCCGGCTGTCTGCAGCTCTAATCCTACTCCTTGTAGGGATCCTCC
TGACAAGTTGTTTACGGTTCATGGTTTGTGGCCCTCAAATGTTAATGGAAGTGACCCCAAGAAATGCAAAACTACAATT
TTGAACCCTCAAACGATAACAAATCTTACAACCCAGCTGGAAACTATTTGGCCAAACGTACTCAATCGAAGGGCAAATG
TGCGCTTCTGGCGTAAACAGTGGCGTAAACATGGCACCTGTGGGTACCCCACAATAGCGGACGACATGCATTACTTTAG
CACAGTAATCGAAATGTACATAACCAAGAAACAAAACGTCTCTGAAATCCTCTCAAAGGCGAAAATTAAACCGGAGAAG
AAATTCAGGAAACGGGACGACATTGTAAATGCCATAAGCCAAAGTATCGACTACAAGAAACCAAAACTCAAGTGCAAGA
ACAATAATCAGATAACTGAATTGGTGGAGGTCGGTCTTTGCAGCGATAACAACTTAACGCAGTTCATAGATTGCCCCCG
CCCATTTCCACAAGGATCACCATTTTTCTGCCCCACCAATAATATTCAGTAT
>P_pyrifolia_S4_RNase_AB009385
ATGGGAATTACGGGGATGACATATATGTTTACAATGGTATTGTCATTAATTGTATTAATATTCTCTGCGTCCACGGTGG
GATTCGATTATTTTCAATTTACGCAGCAATATCAGCCGGCCGTATGCAACTCTAATCCTACTCCTTGTAACGATCCTAC
TGACAAGTTGTTTACGGTTCACGGTTTGTGGCCTTCAAACAGGAATGGACCTGACCCAGAAAAATGCAAGACTACAACC
ATGAATTCTCAGAAGATAGGAAATATGACAGCCCAGTTGGAAATTATTTGGCCGAACGTTCTCAATCGAAGCGATCATG
TAGGCTTCTGGGAAAGAGAGTGGCTCAAACATGGCACCTGTGGGTATCCCACAATAAAGGACGACATGCATTATTTAAA
AACAGTAATCAAAATGTACATAACCCAGAAACAAAACGTCTCTGCAATCCTCTCAAAGGCGACGATTCAACCGAACGGG
AATAACAGGTCACTGGTGGATATTGAAAATGCCATACGCAGTGGTAACAACAATACGAAACCAAAATTCAAGTGCCAAA
AGAATACTAGGACGACGACTGAATTGGTTGAGGTCACTCTTTGCAGTAATAGAGACTTGACTAAGTTCATAAATTGCCC
CCACGGGCCTCCAAAAGGATCACGATATTTCTGCCCCGCCAATGTTAAGTAT
>P_ussuriensis_S35_RNaseDQ839240
ATGGGAAATACGGGGATGATATATATGTTTACAATGGTATTTTCATTAATAGTATTAATATTGTCTTCGTCAACGGTGG
GATACGATTATTTTCAATTTACGCAGCAATATCAGCCGGCCGTATGCAACTCTAATCCTACTCCTTGTAACGATCGTCC
TGAAAAATTGTTTACGGTTCACGGTTTGTGGCCTTCAAACAAGAAGGGACCTGACCCAGAAAAATGCAAGAATATACAA
ATGAATTCTCAGAAGATAGGAAATATGGCAGCCCAGTTGGAAATTATTTGGCCGAATGTGCTCAATCGAACCGATCATG
TAGGCTTCTGGGAAAGAGAGTGGCTCAAACATGGCACCTGCGGGTATCCCACAATAAGGGACGACATGCATTACTTAAA
AACAGTAATCAAAATGTACATAACCCAGAAACAAAACGTCTCTGCAATTCTCTCGAAGGCGATGATTCAACCGAACGGG
CAAAACAGGTCATTGGTGGATATTGAAAATGCCATACGCAGTGGTACCAACAATACGAAACCAAAATTCAAGTGCCAAA
AGAATACTAGGACGACGACTGAATTAGTTGAGGTCACTCTTTGCAGTGATAGAGACTTAACGAAGTTCATAAATTGCCC
CCAACCACAACAAGGATCACGATATCTCTGCCCCGCCGATGTTCAGTAT
>P_avium_S3_RNase_AJ298312
ATGGCTATGTTGAAATCGTCACTCTCTTTCCTTGTTCTTGGTTTTGCTTTCTTCTTGTGTTTCATTATCAGCGCTGGTG
ATGGATCTTATGTCTATTTTCAATTTGTGCAACAATGGCCACCGACCACCTGCAGAGTTCAGAAGAAATGCTCTAAACC
CCGGCCATTACAAAACTTCACCATTCATGGCCTATGGCCAAGTAATTATTCAAACCCAACGATGCCCAGTAATTGCAAT
GGGTCGCGATTTAAGAAAGAGCTATTGTCCCCTCGAATGCAATCCAAACTGAAGATATCTTGGCCGAACGTTGTAAGTA
GCAACGATACAAAATTTTGGGAAAGTGAATGGAACAAACATGGTACTTGTTCCGAACAGACACTTAACCAAGTGCAATA
CTTCGAGATATCCCACGAAATGTGGAACTCGTTCAATATTACAGATATCCTTAAAAACGCTTCAATCGTACCACATCCG
ACACAAACATGGAAGTACTCGGACATAGTATCAGCCATTCAGAGTAAAACTCAAAGAACACCCCTCCTTCGTTGCAAAA
CGGATCCAGCACATCCTAACGCGAATACTCAGTTGTTACATGAAGTGGTATTTTGTTATGGATATAATGCAATAAAGCA
GATTGATTGTAATCGAACAGCAGGATGCAAAAATCAAGTTAACATCTTGTTTCCA
>P_dulcis_SM_RNase_DQ099895
ATGGGGATGTTGAAATCGTCACTCGCTTTCCTTGTTCTTGTTTTTGCTTTCTTCTTTTGTTACGTTATGAGCAGCGGAT
CTTATGACTATTTTCAATTTGTGCAACAATGGCCACCGACTAACTGCAGAGTTCGCGTCAAGCGACCTTGCTCCAATCC
CCGGCCATTACAATATTTCACCACCCATGGCCTATGGCCAAGCAATTATTCAAACCCAAAGATGCCCAGTAATTGCACA
GGGTCGCAATTTAAGAAACAGAATTTGTACCCTTATATGCAATCCAAACTGAAGATATCTTGGCCGGACGTGGAAAGTG
GGAATGATACAAAATTTTGGGAAGGCGAATGGAATAAACATGGTACATGTTCCGAACGAACACTTAACCTAATGCAATA
CTTCCAGCGATCCCACGCAATGTGGAAATCACACAATATTACAGAGATCCTTAAAAACGCTTCAATCGTACCACATCCG
ACAAAAACATGGAAGTACTCGGACATAGAATCACCCATTAAAAGAGCAACTAAAAGAACACCCGTCCTTCGTTGCAAAC
```

GGGATCCATCACATCCTAACAACTCTCAGTTGTTACATGAAGTGGTATTTTGTTATGATTATAAGGCAAAAAAGCAGAT
TGATTGTAATCGAACAGCAGGATGCTGGAATAATGTTGACATCAAGTTTGAA
>P_avium_S1_RNase_AB028153
ATGGCGATGTTGAAATCGTCACTCGCTTTCCTTGTTCTTGCTTTTGCTTTCTTCTTTTGTTACGTTATGAGCAGTGGAT
CTTATGACTACTTTCAATTTGTGCAACAATGGCCACCGACCAACTGCAGAGTTCGCATCAAGCGACCTTGCTCCAAACC
CCGGCCATTACAAAATTTCACCATCCATGGCCTATGGCCAAGTAATTATTCAAATCCAACGAAGCCCAGTAATTGCAAC
GGGTCAAAATATGAGGACAGGAAAGTGTACCCTAAATTGCGATCCAAACTGAAGAGATCTTGGCCCGACGTGGAAAGTG
GCAATGATACAAGATTTTGGGAAGGCGAATGGAACAAACATGGCAGATGTTCCGAACAGACACTTAACCAAATGCAATA
CTTCGAGATATCTCACGACATGTGGGTGTCGTACAATATTACAGAGATCCTTAAAAACGCTTCAATCGTACCACATCCG
ACACAAAAATGGAGCTACTCCGACATAGTATCACCCATTAAAACAGCAACTAAAAGAACACCCCTCCTTCGTTGCAAAA
CTGATCCAGCAACTAATACTGAGTTGTTACATGAAGTGGTATTTTGTTATGAATATCATGCGTTAAAACAGATTGACTG
TAATCGAACAGCAGGATGCAAAAATCCACAAGCCATCTCGTTTCAA
>P_avium_S5_RNase_AJ298314
ATGGCGATGTTGAAATCGTCACTCGCTTTACTTGTTCTTGCTTTTGCTTTCTTCTTTTGTTATGTTATGAGCAGTGGAT
CTTATGACTATTTTCAATTTGTGCAACAATGGCCACCGACCAACTGCAGAGTTCGAACGAAATGCTCCAACCCCCGGCC
ATTACAATATTTCACCATCCATGGCCTATGGCCAAGTAATTATTCAAACCCAAAGATGCCCAGTAATTGCATTGGGTCG
CAATTTAACGAAAGTAAAGTGTATCCCCGATTGAGATCCAAATTGAGGATATCGTGGCCGGACGTGGAAAGTGGCAATG
ATACAAAGTTTTGGGGAGACGAATGGAACAAACATGGTACATGTTCCCAACGGATCCTTAACCAATTCCAATACTTTGA
GCGATCCCAGCAAATGTGGAGATCTTACAATATTACAAATATCCTTAAAAAAGCTCAAATCGTACCAAATGCGACACAA
ACGTGGAGCTACTCGGACATAGTATCACCCATTAAGACAGCAACTAACAGAACACCTCTCCTTCGTTGCAAATCTCAGC
CTAAGAGCCAAGCGAATTTTCAGTTGTTACATGAAGTGGTACTTTGTTTTGATTATAATGCGCTAGTACATATTGACTG
TAATCGAACAGCAGGATGCTGGAATAACGTTGACATTAAGTTTCAA
>P_dulcis_S7_RNase_AY291118
ATGGGGATGTTGAAATCGTCACTCGCTTTCCTTGTTCTTGGTTTTGCTTTCTTCTTTTGTTACGTTATGAGCAGTGGAT
CTTATGACTATTTTCAATTTGTGCAACAATGGCCACCGACCAACTGCAGAGTTCGCATGAAGCGACCTTGCTCCAATCC
CCGGCCATTACAATATTTCACAATCCATGGCCTATGGCCAAGTAATTTTTCAAACCCAACGAAGCCCAGTAATTGCAAT
GGGACTAAATTTGATGCAAGGAAAGTGTATCCTGAAATGCGATCCGATCTGAAGATATCTTGGCCAGACGTGGAAAGTG
GCAATGATACAAAATTTTGGGAAGACGAATGGAACAAACACGGTACATGTTCTGAACAGACACTTAATCAATTCCAGTA
CTTTGAGCGATCCCACGAAATGTGGATGTCGTACAATATTACAGAGATCCTTAAAAACGCTTCAATCGTACCACATCCG
GCAAAAACATGGACCTATTCGGACATAGTATCACCCATCAAAGCAGCAACTGGAAGAACACCGCTTCTTCGTTGCAAAT
ACGATAACAACACTCAGTTGTTACATGAAGTGGTATTTTGTTATGGCTATAAAGCAATAAAGCAGATTGACTGTAATCG
GCCAGGATGCAAAAATAAAATTGACATCAAGTTTCAA
>P_bretschneideri_S34_RNase_DQ414813
ATGGGGATGACGGGGATGATATATATGGTTACGATGGTATTTTCATTAACTGTATTAGTATTGTGTTCCTCTACGGTGG
GATACGATTATTTTCAATTTACGCAGCAATATCAGCCGGCTGTCTGCAACTCTAGTACTACTCCTTGTAAGGATCCTGC
TGACAAGCTGTTTACGGTTCACGGTTTGTGGCCCTCAAACTGGAATGGATCCCACCCAGTAAATTGCACGAATAAAACC
ATGAATTCCCTGACGATGGGAAATCTGACAGCCCAGTTGGAAATTATTTGGCCGAATGTACTCAATCGAAACGATCATG
CAGGCTTCTGGAATAGACAGTGGAACAAACATGGTACCTGTGGAGTCCCCAAAATAAACGACAGCTTGCAATACTTTCG
AACAGTAATCAAAATGTACATAACTCAGAAACAAAACGTCTCTGAAATCCTCGCAAAGGCGAATATTAAACCGGAGGGG
AAAAACAGGACACTGGTGGATATTCTAAAAGCCATACGCAGTGGTACAAACAATAAGGCACCAAAACTCAAGTGCCAAA
AGAAATCTTCGATGACTGAATTGGTTGAGGTCAGTCTTTGCAGCGATCACAACATAACGCAGTTCATAAATTGCCCCCG
CCCATTCCCACAAGGATCACCACATTTTTGCCCAAACAATAGTATTCAGTAT
>P_avium_PA1_AB096918_Prunus_PA1
ATGTTAAAACTACTCCTTCTTGTGCTCTTTTCAGCGGCGTCTCTGCAAGCTATCACCACTCATGGGCAACCGTATGACT
ATCTACAATATGTACTACAATGGCCGAACACGAAATGCGTGAAGGCACGGTGCATTCCAGGGATTCAAAAAACTGAGTT
CACTACTCACGGCCTCTGGCCAACCAACCTCTCCAAGATATTGACATGCAATTCAGCTTCAAAATTTAGCAGCACTATG
CTGCAAAATGATGCTACATTGGTATCGAAATTGAAAACTTCTTGGCCAAATCTGGAGCAGAGAGTTGCCCAAGGAAAAG
ACAATGATATGTGGTTTTGGGCGATGGAGTACGAAAAACACGGCACATGTGCTAAGTTTTCCAGCCAGAATACTTACTT
GTCAAAAGCATGTGATTTGTGGGAAGAAAACAAGATTAAGGATATTTTTGCCAAACACAAAATCATTCCAAGAAACGCG
ACGTATAAAGATGTTTTGCTTACGAATGCTATTCAAATGGAAACTCGCAGTTCGCCTCTCCTTCTTTGCCATAGAGTCA
ACGGCGGTGATTTGTTGTGGGAGGTTGTACTTTGCTATGACGACACGGCTAAAAAACGGATGAATTGTTCTGATCAAAG
TGCAAGACAAACAAATTGCGGAACGGACATCTATTATAAA
>F_vesca_00230_S_RNase_lineage_1
ATGGAAATGGCATTGCTAGCGGTTTTTCTTATCTCAGCTGCTGCTTGTTCATCAGTCACTGAAGCAGCCAAGGCCTACG
ACTACTTCCAGTTCGTACAACAATATCCTATTACCTTATGCATGTTCGACACAAGTTGCATTCCAGGACAATCACTGCC
TCGATACTTCTACATCCACGGCTTATGGCCCAGTAACTTCACTTATCCGCATGACGATTGTGTGGGTACACTGTTCGAC
TACAATCAGATGAGCAGTGATCCTACTCTCTCAAACAATTTGCTGCAATCATGGCCCAGCTTCACGTCCAAATCCCACA
TCAAATTCTGGGCGGATGAGTATCACAAGCACGGGACCTGTTCGGAGATAGACTACCCGCAGCACCGCTATTTTGCCGA

AGCCCATATGCTCTGGACGTTCGTCAATGCATACCGTATCTTAGCAACATACAACATCGTTCCCGGCAGAACCTACCCT
GCGGTTGACATTATATACTTCGTCCAAGCAGCACTCAAAGGAATCACACCATCTGTTATGTGCAAGGCCAATTCTGCGG
GTCAAGAATATCTCCACGAGATCATCATATGTCTTGACAAAACGCTTACCAGATTCATCAATTGTGTTCGACCATCCAC
TTGCCGTACTACAGCGGTAATGTATCCC
>F_vesca_00227_S_RNase_lineage_1
ATGGGGATCATCCTACTTGTCCTGCTGCTTCTCTCTGCGTGTGGTGCAGATGATGCAGCGAACATCTACGACTACATGC
AGTTCGTCCAGCAATGGCCGGCGACATTCTGCTATGGTAATCCGAATTGTGTCCCAAATCCACCACACAGTACTTTTAC
GATTCACGGCCTCTGGCCCAGCAATTACTCGACACCTGAATTTCCATGCGTTGGCACACCCTTTGATCCCTCGCAGATG
TTCGCTGCCGAAAACCGTGGTTTGAGGGACTATTTTCTGCCGCAAGCATGGCCACAGTTGAATGCTCGTTATACCAACT
TGGAGTTCTGGAAATTCGAGTACGAAAAGCACGGTATGTGCTCGGAGAACTATCTGAATCAGATGGATTATTTCAGGAA
AGCTTACTGGGCGTGGGCTCGATTCAATGCTTATGTTCTGTTTGCGGCATCGCCTAAGCAAATCTACCCTGGCAATTAC
TATTACACAAGAGATATTGAAGCAGCCATTCAGAGAGTGACCAGTGAGAAGCCTCTGCTTATGTGCAAGAAGGTAAGAG
TAGGTACGGTTGATAGTTGTCTATTGCAGGAGGTCATCATCTGTCTTGATCGTTGGGCAGACAACGTTGTACCTTGTCG
CCTTCGACTGTCATCGTGCAACGTCCCAATTATATATTATGGTTAC
>F_vesca_26822
ATGCATGAGTGTGTACGTATCTTAATTTTCATATATGGCAATCTCATTTCATATTTGGCAATCTCATTAATTGATGAAA
ACACAATCGACTATACAAAGCCGATCATTTCACAAGGAACACCCTTCCAGTACTACCAACTGGTCATGCGATGGCCAAA
AGCAGCATGCTACCACAAACCTGCTGAGCCAAAGATTTGTGTTATGGATGATGATCATGTTCCGATGAGGTTCACCCTC
CACGGAATGTGGGCGACGAACTGGACAAATATCAAAGATGAAATTCGGTGTGAAAAGGCTGGAACGCCATTTGATGAAG
ATGAGATGACTAAACAAAAGACACTGCAAGAACAACTGGAGCAAAGCTGGCCGACGTTACTGGATAATTATCCAACTAA
TATGGAATTCTGGAAGCACGAATACGATAAGCATGGCCGATGCAGCCAAGATACAACTACTCAGACCGAATACTTCGAA
AAAGCTCACAAATTGTGGCAGGAATTGCAGGTTGTGTACCAAGCACTTGCAGATGACAAGATCATACCTGCAGCTGATA
AGATTCACACCTACAACCAGATCGAGCGAGCAATCGCTAAACGATTCGGCAGTAGTTGGAAGGTTGTGCTTTTTTGCAG
AAAGGTGAATCTGACGCCTCGTGGTACCGGTGGAACAAAAACGATTCTACTTCTTTTGCATGAAATCGTATTCTGCTTT
GATAACAAAGCAATGGAAAAGAAGAGTTGCAACAGAAAATCGAACTGCGAGAACGCGAAAACTATAACACTGATAAAT
>F_vesca_22609
ATGATGAAGAATTCAGTCTTCGTTTTCTTTCTGTCAGTCTATGTCCTTGTCCAACTTATCAAGCTTTCAAATGCTGAAC
CATTTGAGTACATGCAGTTTGTGCTCCAGTACCCACTTGGAGTTTGTTATGGTACACAAAAAGGTGATTCCAAATCACC
TCTGCCTACCAAGTTTACCGTTCACAGCATCTGGCCGTCAAACTTCTCCGATACACGAGTCGTTTGTAGGCAAGCACTG
ATAAGTCACCCCTTTAATAATGCGCAGATGACGTCGTCTCTGCAGACTGATTTGACAAACTCATGGCCATCTGTGATAA
CAAAAAAAAGCGACATGCAGTTTTGGCAACATGAGTATGAGGAGCATGGCGCTTGTTCAGTAGACAAGGGTGTACCACT
GTTCACCCAGAAGTCCTACTCTGAGAGAGGAAACCAGCTCTGGAACCAATATGACATCCATGCACTGCTTGATCAAAGC
AATATCAAACCGGATTCTTCAAAGCCATGCAAAATGACTGAGATTGTAGCTGCAATCCAAAAGAAAATCGGGAAAAACA
CACTCCTGTACGAAGGTGCAAGGAAGATAACAAAATTTACATAC
>F_vesca_12961
ATGTTGTCATTTCTCATTGTTTTGCTTAGTGTTCAAATGCTGCTAGCAGCAAATCCACCTCCACCTCCACCTCCAGCTG
TTCCAGCTCTTGATTCATACAAGCTGGTCATTGAATGGCCAAACACTTTTTGTCTTGTTGAGCCACAGCCACCATGCCA
ACAGCATCCACAGTCTTTTACATTGCATGGCCTTTGGCCACAGGCGGGTGGTCGATCGTTAGTGAACTGTCCTGGTTCA
CCAATGGAAGATCAAACCCTAGAGGCAAATAAAGGTGATCTGACAAAGTTTTGGCCTGATCTTAGGAACTCGGACTTTG
ATAAGAGTAAAAGCTTTTGGCGTCAGGAGTGGGACACACACGGTCGCTGTTCTGGCAAGCCTCCGGCCGACTACCTTAC
CATGGTCTTTAATGCAGTAAAAAAGTATGACGTGCAAAAGTTACTTGCAAAGAATGGAATTTTACCAGGTGATCCGACA
GGGCATACGGCGGCACAGTTTGAACAGGCAATATTCAAAGAAACTAAACTGCACACAGAGATTGAATGCCGGACGAAGG
ACTCGAAGGAGTATCTATTTCAAATTTATTTCTGCTTAACACCTCAAGGACAGTTCAAGAATTGTCCGATCCGTTACAG
GTGTGCAAATCAACAAATCTACATGCCCTTGCCTCCGCCCAGTCCC
>F_vesca_scf0513144_1_Prunus_S_RNase_lineage
ATGCATGGTATGTTTCTATATATTGTTGACGTCGGTTTGCCGAAGGAAAGGTCTATCTATATAACAAGCATGAGATTCG
TGACTGTGTTATTGTTGTTGTTTCTTGTTTCTAGCACTATAATGTCTCGAGGATCTTATGATCATTTCAAATTTGTACA
ACAATGGCCACCGGCCTTCTGTGCCACGAATCGCTGTAAACCAGGGGCAAAACAGATGTTAATGTTCACTATCCATGGT
CTCTGGCCAGATAATAAAACTTATGTGCCTTGTCGTGCCCTTTTACCGTTGTTTGCAGGCCGTCAGATCCTTAATGATG
CGAGTCTGGTAAACAAGCTGTTGACCTCTCCAAATCTGAAAGGAATTGATCGCAAGTTTTGGAAAGAAGAATGGGACAA
ACACGGAAGATGTTCCTCGCAGACGTACGACCAAAGACAATATTTCGAGCGGTCACACAACATGTGGATGGAAACGGAC
TTGACCAACACCTTTGGCAACCTTATCAAAGGAAACAGTCTACAACGGAACGTCGCAGATATAGAAAACCTGATTCAAA
AGGTAACTGCAACGGAAAAAAAACCACTCCTCCGTTGTAAAAACAAAAGATCCACACCTCACGATGAAGTATTAACTGA
AATTATAATTTGCTATGCACACAATGGAACTAGTCTGATCGATTGTGTCGGTCCATCCACCTGCACGGACGGATTTTAC
TTTCCT
>F_vesca_scf0513063_1_Prunus_PA1_lineage
ATGGGTTTTGTTTTTTTTCTGGTGGTTGTGCCGCTTACCTCCAAAGCACTTTCGGGTGGTCATCGCCGTCAACATATCA
ATGCATACGGCGATGATCCCGGCTATGATCTGGTGTTATTTGTCCTACAAGTCACTAATCCTCGTGACAAGGAGAATGG

ACCTTACTTAACCATCTTCGGCCTTTGGGCCGAATATCTTCAGAATGGGGCCCCCCCTGAGACCGGGTACCTTCAACCG
TACCTAAGAAAGAGGCTCCATGTAATATGGCCAACCAGAAGAGACGGAAGCTACCCAACAGACCGGGCCTTTTGGCTGC
ACGAAGTTGATAAACATGGGACCTGTATGGCAGATATATTCGGACGGGGGGAGCAGTACCTACAAAAGGCAAAAGAATT
GTATGAGCAGATCGGGATAGGCCAGTTCTTGGGTCGTAACGGAAGGTGGCTGCCAAAAATTGAATTCAATACCGCGGAT
TTACTGGGGGCGATCGGTACTGAACGCAGACCACTATTAGAATGTGAAGGAAATCATCTCGTCAGAATTTTATTCTGCT
ACAGCAAACACTGGGTGATTAAACCCTGCTTGCCCACCCACCCCGATCCAGCAAGAGATTGTCCCGATAGGATTTCTTA
TGTTCTGCCGCAGCAGCAG
>F_vesca_scf0513159_1_Prunus_PA1_lineage
ATGGAAGCACTTGTGATATTATCATTTGTCCTTCTATCTATTTTTCCTCAACTGAGTGCGCAGCTAGGAGAACCGTATT
ACCAATACATACAACTCTCACTTCAAGTCCCCAAGTCGAGGGACCCTCAATCTACTTTGGAGTATTTCCCTTTGCACGG
CTTTCGGCCGGTAAACACATCTCTATCTAACCCGAATTTGCCTTTGGGTTTTAACCGGTATACACCTATCGAAAGGTAC
CTGAGAACTGAACTCAAGTTATGTTGGATGAGTATAGGAGTACCAGGCCAACGCACGATGACGGATGAGCGATTTTGGC
TATATGAATACAGTAAACATGGGCAATGTACTATTAACGAATATCCGACCCCTCAAGATTACTTCGCCATGGCAGTTAA
CCTGTGGCATCTCTATCCTGTGGATAAGTGGTTTAAGAATAATGGATTGCAGCCAAAAAAAGATAAACCGCTCCAAGAT
TTTGTTGCTGTCATCTCAAAAGAATTCGGCGCTATTCCATGGCTAACGTGTTACGACAACGGGAAGAAGCTGAAGGAGG
TGGGCTTGTGTTTCTTAAAATCATCCAGTGTGAATGCGAATCCTGCCCCTACAAGTTGTCCTATAAGGGCACTAACGTA
TGATCTAAGATGTAATTATTTGATACAATATGATCTAGGTCCA
>F_nipponica_gi561674690_gi561985884_gi561957436_Prunus_S_RNase_lineage
ATGCATGGGATGTTTCTATATATTGTTGACGTCGGTTTGCCGAAGGAAAGGTCTATCTATATAACAAGCATGAGATTCG
TGACTGTGTTATTGTTGTTGTTTCTTGTTTCTAGCACTATAATGTCTCGAGGATCTTATGATCATTTCAAATTTGTACA
ACAATGGCCACCGGCCTTCTGTGCCATGAATCGCTGTAGACCAGGGGCAAAACAGATGTTGATGTTCACTATCCATGGT
CTCTGGCCAGATAATAAAACTTATCTGCCTTGTCGTGCCCTTTCACCGTTGTTTAACGGGCAGATCCTTGATGCGAGTC
TGGTAAACAAGCTGTCGACCTCTTGGCCAAATCTGAAAGGAATTGATGCCAATTTTTGGGAAGAAGAATGGGACAAACA
CGGAAGATGTTCCTCGCAGACGTACGACCAAAGACAATATTTCGAGCGGTCACACAACATGTGGATGGAAACGGACTTG
ACCAACACCTTTGGCAACCTTATCAAAGGAAACAGTCTACAACGGAACGTCAGAGATATAGAAAACCTGATTCAAAAAG
TAACTGCAACGGAAAAAAAAACCACTCCTCCGTTGTAAAAAAAAAAGCATCCACGCCTCACGATGAATTTTTAACTGAAAT
TATAATTTGCTATGCACACAATGGAACTAGTCTGATCAATTGTGCCAGTCCATCCAACTGCACGGACGGATTTTACTTT
CCT
>F_niponica_gi561805796_Prunus_S_RNase_lineage
CTCTTTTCCACAGGTTACTACGACTACTTTCAGTTGGTGCAACAATGGCCACCCAACGTCTGCTTTAAAAGAAAGTCAC
CTTGCTACAATAACACGCCATCACTAATCTTTGGTATTCATGGCCTATGGCCAAGTAATAATACAGTGAATAAAAACCC
CAGTTGCCCACCGAACCCATTTAAACCGCAGGTATATATGATCTTTTGCAGGGTAAGCATGTTAAATAATCAAACGCTG
CTAGCGGACCTTAGCCGTTCTTGGCCCGACGTGGTAAATGGGGACGATCCAACGTTTTGGGCCAAGGAGTGGGACAAAC
ATGGCACATGTTCAGACCAGACATTCGGGCAAAAACGATACTTCCAGCGATCCCACGAGATTTGGGAACAATATAATAT
TACGGATATCTTTAAAAAAGCTGGCTTGATATCGATGACGGGGCCAGCAGTACCAAGACGATGCAACGTCGCAAACATA
CTAACACGCATTGAAGCGGTGACTGGGAAGAAACCTGTCCTTTATTGTCAATGGAAATCGATATACCAGCCGCAGCCGC
AAGGACAACCACCAAAGAGGCTAACCATTCGATTGATTCAGGAAGTGAGCCTTTGTTTTGATTATTATGGAACGACATT
AACTGATTGTACCAATATCAATACACTATGCAAAGGACGTAATGTCTGGTTT
>F_nipponica_gi561877040_S_RNase_lineage_1
ATGGGGATCATCCAACTTACTGTCCTGCTGCTTCTCTCTGCGTGTGGTGCAGCTGATGCGGCGAACCTCTACGATTACA
TGCAGTTCGTCCAGCAATGGCCGGCGACATTTTGCTATGCTAACCCGGATTGTATCCCAAATCCACCGCACAGTACTTT
TACGATTCACGGCCTTTGGCCTAGCAATTACTCGACTTTTGAAGGGAAATGCGTTGGCACACCCTTTGATCCATCGCCG
GTATTGCATTATTGCATGCATGGAATATTATATCACTTCTATTCATTTAGTCTTTCATTTTCAGAATTCTTTCTGCCGC
TAGCATGGCCTCAGTTGGACGCTCATTATACCGACATGGAGTTCTGGAAATACGAGTACAACAAGCACGGTACGTGCTC
GGAGAACTATCTGAATCAGATGGATTATTTCAAGAAAGCTCACTGGGCGTGGGTTCGGTTCAATGCTTATAATCTGTTT
GCAGCCTCGCCTATGCAAATCTACCCTGGTAATTACTACTATACAAGAGATATTGAAGCAGCCATTGTGAGAGTGACCA
ATGTGAAGCCTCTGCTTATGTGCAAGAAGGTGAGAGTGGGTAAGGTTGATAGTTGGCTATTGCAGGAGGTCATCATCTG
TCTTGATCGTTGGGCAGACAACGTTGTACCTTGTGGCCCTCGATGGTCATCGTGCAACGTTCCAATTATATATTATGGT
AAC
>F_nipponica_gi561793890
ATGCATGAGTGTGTACGTATCTTAATTTTCATATATGGCAATCTCATTATTGATGAAAACACAATCGACTATACAAAGC
CGATCATTTCACAAGGAACACCCTTCCAGTACTACCAACTGGTCATGCGATGGCCAAAAGCAGCATGCTACCACAAACC
TGCTGAGCCAAAGATTTGTGTTATGGATGATGATCATGTTCCGATGAGGTTCACCCTCCACGGAATGTGGGCGACGAAC
TGGACAAATGTCAAAGATGAAATTCAGTGTGATAAGGCTGGAACGCCATTTGATGAAGATGAGATGACTAAACAAAAGA
CACCGCAAGAACAACTGCAGCAAAGCTGGCCGACGTTACTGGATAATTATCCAACTAATATGAAGTTTTGGGAGCACGA
GTATGATAAACATGGACGATGCAGCCAAGATACAACCACTCAGACCGAATACTTCGAAAAAGCTCACAAATTGTGGCAG
GAATTGCAGGTTGTGTACCAAGCACTTGCAGATGACAAGATCATACCTGCAGCTGATAAGACTCACACCTACAACCAGA
TCGAGCAAGCAATCGCAAAACGATTCGGCAGTAGTTGGAAGGTTGTGATTTTTTGCAGAAAGGTGAATCTGACGTCTCG

TGGTACCGGTGGAAAAAAAACGATTCTACTTCTTTTGCATGAAATCGTATTCTGCTTTGATAACAAAGCAATGGACAAG
AAGAGTTGCAACAGAAAATCG
>F_nipponica_gi561844698
ATGATGAAGAATTCAGTCTTGGTTATCTTTCTGTCAGTATATGTCCTTGTCCAACTTATCAAGCTTTCAAATGCTGAAC
CATTTGAGTACATGCAGTTTGTGCTCCAGTACCCCCTTGGAGTTTGTTATGGTACACAAAAAGGTGATTGCAAATCACC
TCTGCCTACCAAGTTTTCCGTTCACGGCATCTGGCCGTCAAACTTCTCCGATACACGAGTCGTTTGTAGGCAAGCACTG
ATAAGTCACCCCTTTGATAATGCGCAGACGACGTCGTCTCTGAAGACTGATTTGACAAACTGGCCATCTGTGATAACAA
AAAATAGCGACATGCAGTTTTGGCAACATGAGTATGAGGAGCATGGCGCTTGTTCAGTAGACAAGGGTGTACCACCGTT
CACCCAGAAGTCCTACTTTGAGAGAGGAAACCAGCTCTGGAACATGCAATATGACATCCATGCACTGCTTGATCAAAGC
AACATCAAACCAGATTCTTCAAAGCCA
>Rrugosa14_32
ATGAGTCAAGATCCAACATTGGTATCCGAACTACAGAGATCGTGGCCAGACGTGGGAGGTGGAGATGATATGGGGTTTT
GGGGACGTCAGTGGAATAAACATGGGACATGTTCGGAACAGACATTTGGGCAAATTCAATATTTTCAGCGAGCAGACGC
CATTTGGAACAAAGCGAAAATGAATATTACTATGATCCTTAAAAACAATAAAATCTCAGCAGGGGGGGGGAAATATAAT
TACACGCATATAGAAGGAGTCATCAAACGGGCAATCGGTGACACACCCATCATTCGTTGCATATGGAGAAAGAGGGCTC
AGTTGTTGCATGAAGTGGTTACTTGTTGGTCCCAAGACGGTCAAAATTTGATCAAATGTAACTCTACGCAAAAATGCAG
TTCGAATTTTGATATCGAATTTTTA
>Rrugosa14_33
CTGCGGCGAACCGATCTCCCAGATGAACTCCTTCAGTCTTGGCCCAATGTAGTGACTCCAAACAATCCGAATGCAGTTT
GGTCCTTCTGGAAATACCAATACAACAAGCACGGCAGCTGCGCCTACTGGAGATTCCAAAGACTGGAGGCTTACTTTAG
AAAAGCAGATGACTTGTATCGCAGTGATAACGTACAACATTTGTATGATTCATTCACCAGCAATCGCCAAGTCTCTGCT
ATCAGATATATGAATGACGAAAAAAATTATGGAGGGAAATTTCCAGGACTGCGGTGCAATGGAACGGGATTCCTGATGG
AAGTGAGTATCTGTTACACAAAAAAAGCAACTAAAATGATTGATTGCCCTAAACGGGGGAATTGTACGAGGCTAATTGA
ATATCCAATG
>Rrugosa14_34
ATGTCCGCGAACTATCATCTTCAAACCATATTGCTCCCATCCTCGTGGAGAAGCTACCCTATCGGTGTCTCCAACACAG
ACTTCTGGGAGAATGAGTATAACAAACATGGCACATGTTCGGAGCTCAATTTGCCACAGACGGAATACTTCACCAAAAC
CCATTGGCTATGGATGGTCAACAACATTTATAGTATTTTTTCATCTTCGGCCAACAGACAAGGATATCTAATCTTGCCG
GGTTTCACCTATGACTACATCAATCTTCTTGCCGTTGTTCAACTAGGAATCGGTGGCTTCACTCCCGTGCTTACTTGCA
AGGTCATAAATAAAAACCAATACGTTCTTCACGAAGTGGTTATTTGTTATGATGCCTTGGGGATCAACCGCATCCATTG
TGTGCGACAATCGAGTTGCTCTTTCGCTCCTAACGGACGTGTGCTCTATCCT
>Rrugosa14_36_Rrugosa19_35
ATCTCAGAGTTGATGGGCAGTCTGGAAAAGAGTTGGCCATCTATGAGCTGCCCAAGCAGTAATGGTTACAGGTTCTGGT
CACACGAATGGGAGAAGCATGGCACTTGCTCCGAATCTGAACTTGATCAGAAAGATTACTTCCAAGCCGGTCTCAAGCT
CAAGGAAAAAGCAAACCTTCTTCAAGCCCTTAAAAAAGCTGGTAATTTAGTTCGTTTATACTCAATTGTTTATAAGAAA
TTAACCAAGCTAGAT
>Rrugosa19_14
ATGGATCGCTCATTGGTATCTAAACTGAATATATCGTGGCCGAATGTGAAATATCCGAATAATATCCAGTTTTGGGATA
AACAGTGGAGAAAACATGGCTCATGTTCCGTGCACACGTTTAACCAAACAGAGTACTTCACGCAAGCGGATAATCTTTG
GAACTCACACAACATTACTGATATTCTTATAACTGGTGGGATCAAGCCAAATGGATCAGAATATGCATACGATACGGTA
GGGCGGCCCATCCAAATTGCAACCGGGAAGGTACCCGAACTTCGTTGCGCACCATCCTCCCCTGGAAGGCAATTGTTGC
ATGAAGTAGTACTTTGTTATAATCATAAGGGAACAACGCCCATCGACTGTAACCCTTTACAGGCAACCTGCGATCGTAA
GTCCCTAATAAAGTTTACTCAG
>Rrugosa19_22
AAAATCGTACCAGCAGCTGACAAAACTTACACCTACACCCAGCTCGAGCAAGCTATTGGAAAACGATTTGGCAGTAATT
GGAAGGTTGTCATTCTTTGCAGAAAGGTGAATCTCTCACCTGGTGGTCGTGGACCAAAACAAATTCTATTTCTTTTGCA
TGAAATCGTATTCTGTTTCTATAACAAAGCAACGGAGAAGAAGAGTTGCAACAGAAAAACAAATTGTGAGAACGCGAAA
ACTATAACCTTCATAAAT
>Rrugosa19_50
CTGAGCCACCAACTCCAAACGGATCTCAGTACGTCTTGGCCCGATGTACTGAACGGAATCAATTCGCGGTTTTGGACCC
ACGAATACAATAAGCACGGCAACTGCGCCAACCATACATTCACAACGCCGCAGGCTTACTTTAAGAAAGCAGATTACTT
GTATCGCAGCTTCGCTGGCGGACAAAATTTGAATGGTTTATTCGTCCGCGGCGGCATCCGCCCGGGACAGCAAAGCGAC
ACTATGATATATATGAATCTCGTAAAAAATGCGACTGGAGAGTTTCCCCGACTGTGGTGCAGTGGAAATAGACTGAAAG
AAGTAGTTATTTGTTACAATAAAGATGCAACTCAAGTGATTGATTGTCCTCCACTCGTAAGGCCGAATAATTGTACGGG
GCGCATATTAATATCAACAAGAAGACCCCCTTATGGTCTAACCATAAGACCCCCTTATGGTTCAACCATATTGGTATTG
TCCTTTAGTTTACCCTTGGGGCTAATTATCTTTAAATATGGTCCAAGCTATATTAAATCTAAGCTAGCT
>Rodorata_gigantea_7

TTCGATGAAGCATTTAAAACCATTCTAGAAAGCAATTGGAAGAGTTATGGAAGCGGGTCGAAAAAAAGTTTTTGGGATC
ATGAGTACAATAAACATGGAACCTGCACCATCCTTAAAATTCATAATCCTCGCGCTTTTTTCAAAAAAAAAAAAAAAA
GTTATGGTCAGACACTAACGTGCACCCAGTGCTTGATGGATCGGTTTTACCAAATGTTCTGAGCTCCTTTGATGATGTG
ATGTCCACCATCGAGAGGGGATTTGGAAAAAGGCCAACTGCGTTTATGTTAAAGAATATGCCCGGACAGACCTGCAACA
AGTGCGTTTATGTTACGACCTCC
>Rodorata_gigantea_10
ATGTATACAAATCAAACATTGGTAAATGACTTGACTCGCTCTTGGCCGGACCTGAAAAATGGGAACAATGGAAGGTTTT
GGAAAGAGCAGTGGGATAAACATGGCAAATGCTCAGAACAGACATATACACAAACACAATACTTCCAGCGCTCCTTCCA
GATGTGGAACCAATTCGATATTACTAGTATCTTAACTGCCGCTGGCGTCGTATCTCCGCCAGGGACACAACCAAGTAAA
>Rodorata_gigantea_13
TTGCAGTTTTGGACCGATGAATACAATAAGCACGGCAGCTGTGCACAGCCTAAACTCCGAACACCGGAGGCTTTCTTTA
GAAAAGCGGTTGACTTGTATCGCAGTAATGACATACAAGATTTAAAGAATTTATTCGCCGGCATTCGCAATAGCATCCC
CCACGGACAGCGATTCCACGTGGGTCTTTGCAGCAATTATATGCAAACCGTAGAAAGTGCGATAGCAGGGAAACAGCCA
CGACTGTGGTGCAAGAAAAACCAACAGGGACACATACTGAAGGAACTCAAGGAAGTAGTTATTTGTTACGATGGACTTG
CAGCTGTTGTGATTCCTTGTCCTCGAGGCATAGGGCCGAATTGTTATCAGAAGTTCATACATTATCCAATA
>Rodorata_gigantea_14
GATTTTGAAGCAGCCGTCAAGTCCAAGATCGGTACCGACACAAAACCTCTCATCCTTTGTAAGCAGGACAAACTAACTC
GTGGGATCACAACAACCAACTTCATTTTGAGGGAAATTGTGATCTGTTTCGATCACCAGGGGACAAATCTGGTCAATTA
TATTGTACCCGATCAACAGATTGTGACCAGAAGCAGAAGATATACTACCCTTAGC
>Rodorata07_8_Rodorata_gigantea_23
TACTCGGCTTGTTTTGGTTTTCAGATAACTCCGTCGTTGAAAACCGATCTTCTCCAATCTTGGCCCAGCGTCGTGACAA
ATAAAGATGATATGTCGTTTTGGGAACACGAATATAATAAGCATGGGTCCTGTACTGCACCAGCTATCACTCAGACAGC
GTATTTTGAGAGAGCTCACAAGTTCTGGAAGGAGTATGATCTCTATTCCATTTTGGAACAAAAAAATATCAAGCCGGGG
CAATCTCGATCGTATTCACTGGCTGATTTTGAAGCAGCCGTCAAGTCCAAGATCGGTACC
>Rodorata07_9_Rodorata_gigantea_17
TCTTGGCCCAATTTAGTGGCTCCAAACAATCCGAATGCAGTTTGGTCCTTCTGGAAATACCAATACAACAAGCACGGCA
GCTGCGCCTACTGGAGATTCCATAGACTGGAGGATTACTTTAGAAAAGCAGATGACTTGTATCGCGGTGATAGCGTACA
ACATTTGTATGATTCATTCACCAGCAATCACCAAGTCTCTGCTATCAGATATAGGAATGACGTAAAAAATTATGGAGGG
AAATTTCCAGGACTACGGTGCAATGGAACGGGATTCCTGATGGAAGTAGTTATCTGTTACACAAAAAAAACAACTAAAA
TGATTGATTGCCCTAAACGGGGGAATTGTACGAGGCTAATTGAATATCCAATG
>Rodorata07_25
ATGTCCGCGAACTACCGTCTTCAAACCATATTGCTCCCATCCTCGTGGAGAAGCTACCTTATCGCTGTCTCCAACACAA
ACTTCTGGCAGACTGAGTATAACAAACATGGTACATGTTCGGAGCTCAATTTGCCACAGACGGAATACTTCACCAAAAC
CCATTGGCTATGGATGGTCAACAACATTTATAGTATATTTTCGTCTTCGGTAAACAGACAAGGATATCCAATCTTGCCG
GGTTTCACCTATGACTACGCCGATCTTCTTGCCGCAGTTCAACTAGGAATCGGTGGCGTCACTCCCGTGCTTACTTGCA
TGGTCATAAATAACAACCAATACGTTCTTCACGAAGTGGTTATTTGTTATGATGCCTTGGGGATCAACCGCATCCATTG
TGTGCGACAATCGAGTTGCTCTTTCGCTCCTAACGGACGTGTGCTCTATCCT
>Rodorata07_27
ATTAGTCAAAAAATTACAAAGGCACTCGATAAAGCTTGGTCGAGTCTTTCGGCTACGTGGCCAAACGTTAAATTTTGGG
AACATGAATATAACAAACACGGTTCATGTATCGTAGATTTATTACCAACTTCTACGGATTACCTAATGAAAGCACTTTA
CCTGTGGGAGCAACTCAAGTTTGACGAGGTGATGGGTAAGGGTAAGCAATTTGAGCCAAATACACTATACAAAACCGAG
GATATTCTTAAGGGCATCAAAGTTTCACATAATGTTCGACCAGAATTGACCTGTATCGGTAATAAACTAACAGATGTTA
GGTTTTGTCATACAAGAAATTGGGTATTGGTAGATTGCGAGGGCATCCAAACATCTTGTACAGGTCAAATTAAATATGT
TAGGCAAGTG
>Rmoschata08_3
AAGGAGTATGATCTCTATTCTGTTTTGGAACAAAAAAAAATCAAGCCGGGGCCGTCTCGATGGTATTCACTGGCTGATT
TTCAAGCAGCCGTCCAGTCCAAGATTGGTACCGACAAAATACCTCTCATCCTGTGCAAGGAGACCGCATTAACTCGTGG
GAGCCGAACAACTGAATTAATTCTGAGGGAAATAGTTATCTGTTTCGATCACCAGGGGACA
>Rmoschata08_10
ATGGATCGCTCATTGGTATCTAAACTGAATATATCGTGGCCGAATGTGAAATATCCGAATAATATCCAGTTTTGGGATA
AACAGTGGAGAAAACATGGCTCATGTTCCGTGCACACGTTTAACCAAACAAAGTACTTCACGCAAGCGGATAATCTTTG
GAACTCACACAACATTACTGATATTCTTATAACTGGTGGGATCAAGCCAAATGGATCAGAATATGCATACGATACGGTA
GAGCGGCCCATCCAAATTGCAACCGGGAAGGTACCCGAACTTCGTTGCGCACCATCCTCCCTTGGAAGGCAATTGTTGC
ATGAAGTAGTACTTTGTTATAATCATAAGGGAACAACGCCCATCGACTGTAACCCTTTACAGGCAACCTGCGATCCTAA
CTTCCTAATAAAGTTTATT
>Rmoschata08_23
ATGCGTCGACACAACAATCTCGAAACTCAATTGCTGTCATCCTCATGGCCAAGCTACACTGGTCTGTCCAACCTGCACT
TTTGGGAGTACGAGTATAACAAGCACGGCACTTGTTCAGAGAATAACCTCAAACAGACGGACTATTTCACCACAGCCGA

CGCTTTGTGGAGGAGCTACAATGTTACCAATATGCTTTTAACATCGCGCCACCAAATCTCGCCGGGATCCTCCTATCGT
TACGCTGACATTCTCAATGCCATTCGACAAGGAATTGGGGGATACACTCCTTTGATTCTTTGCAAGAAATATCGGGCAA
AAAATAATTTGATGATTCTGCACGAGGTGATAATTTGTTTCGATCCCTTGGGGAATAACGTCATCGCTTGTGGCAGAAG
ATCGAGACATAGTTGCTCTTACACTAACAGCGGGTTGATAGACTATCCTAAG
>Rmoschata08_24
ATAATCGACTATTTGGAAACAGATTGGCCGTCGCTGGCCTGCCCGAGCCGCAACAGTACGCAGCTTTGGGCCGAAGAAT
GGAAAACACATGGCACTTGCTCACAGTCTGTCCTTCATCAGTACAAGTACTTCGCGAACGGTTCGACCCTCAAAGCCTC
GGTAGACATCCTCAAAATGCTACAACTTGCTGGTACCTATTTTTTCATCTTTACCTCCCTCGTCATTTATATTATTCGA
TTCATGCATTTGAAAAATAAGTTATCATCAATACTATTCCAACTTGCAGGAATCCAAGCGCGCAGATGGGAAAGTTTAA
CT
>Rmoschata08_26
GACAAACTTTGGAATGCGCACAACCTTACTGATATCCTTCGATCTGCTGGCATAGTTGGAAATGGGTCGCCAAGGAATT
ACATAAACATAGAAGGCCCCATTCAAAACAAAACTGGAAAGGTACCCCTCCTTCGTTGCAGGGAGCCACTAAGTGAAAG
GCATAAACCTCGATATCAGCGGACTCAATTATTACATGAAGTGTCCTTTTGTTACAATTATGATGGAATAACTCCGATC
GACTGTAACCGCGCACAGAGCACGTGCAATTCGACAAGACCAGTAACGTTTACTGAA
>Rmoschata17_6
AGTTTGGAAAGGAACTGGCCATCACTATCCTGCGCAAGCCGTACTAGTAGCAGTAAGAAACTATGGATGCAAGAATGGC
AGAAATATGGGACTTGTGCAGAATCACTCTTTGGAGGTCAATACCAATACTTTCATGCAGGTCTCAATCTGAGGAAGAA
AATAGACATGCTCCAGATACTCAGCAATGCAGGTCTGATCACTACTATAATCCAACTTTCGGATTTCAGCTCAAATAAA
TTAGTGGAACTCAAAACTTTTCGCTTAATA
>Rmoschata17_12
CAGGCTGTCTACCAAGCATTTGCCGATGAAAAAATCGTACCAGCAGCTGACAAAACTTACACCTACACCCAGCTCGAGC
AAGCTATTGGAAAACTATTTGGCAGTAATTGGAAGGTTGTCATTCTTTGCAGAAAGGTGAATCTCTCACCTGGTGGTCG
TGGACCAAAAAAAATTCTACTTCTTTTGCATCAAATCGTATTCTGTTTCGATAACAAAGCAACGGACAAGAAGAGTTGC
AACAGAAAAACAAATTGTGAGAACGCGAAAACTATAACCTTCATAAAT
>Rmoschata17_23_Rmoschata08_36
CTGCGGCGAACCGATCTCCCAGATGAACTCCTTCAGTCTTGGCCCAATGTAGTGACTCCAAACAATCCGAATGCAGTTT
GGTCCTTCTGGAAATACCAATACAACAAGCACGGCAGCTGCGCCTACTGGAGATTCCAAAGACTGGAGGCTTACTTTAG
AAAAGCAGATGACTTGTATCGCAGTGATAACGTACAACATTTGTATGATTCATTCACCAGCAATCGCCTAGTCTCTGCT
ATCAGATATATGAATGACGTAAAAAATTATGGAGGGAAATTTCCAGGACTGCGGTGCAATGGAACGGGATTCCTGATAG
AAGTGATTATCTGTTACACAAAAAAAGCAACTAAAATGATTGATTGCCCTAAACGGGGGAATTGTACGAGGCTAATTGA
ATATCCAATG
>Rmoschata17_24
GGATATCTAATCTTGCCGGGTTTCACCTATGACTACACCGATCTTCTTGCCGCTGTTCAACTAGGAATCGGTGGCGTCA
CTCCCGTGCTTACTTGCAAGGTCATAAATAAAAACCAATACGTTCTTCACGAAGTGGTTATTTGTTATGATGCCTTGGG
GATCAACCGCATCCATTGTGTGCGACAATCGAGTTGCTCTTTCGCTCCTAACGGACGTGTGCTCTATCCT
>Rmoschata17_27
ATGTATTCAAATCAAACATTGGTAAAAGACCTGACTAGCTCTTGGCCGGACCTGAAAAATGGGAACAATCAAAGGTTTT
GGAGAGAGCAGTGGGATAAACATGGCAAATGCTCAGAACAGACATATACACAAACGCGATACTTCCAGCGCTCCTTCGA
GATGTGGAACCAATTCGATATTACTAATATCTTTACTGCCGCTGGCGTCGTATCACCGCCTGGGACAAAACCACGTATA
GTCAACGCGAGCGACTTAGAAGCACGCATTGAAGCCGTAACTCATTCGATACCTGTCCTTCGGTGCAGAAAAGCGGCAG
TTCTCAGAGGTCAACCGCCAGTTGATTTAATTTATGAAGTGGGCATTTGTTACGATTATGTTGGAACAAATCCGTATCC
TTGTGACCGTTCACACGGAAGATGCACTGCAAATGATATTTACTTT
>Rmoschata17_32
CTCCAACGAGCTGGCATCTTATCGAGGACACAAAAAGACTACGGAGACATAGAACTAGCCATACGATCAAAAACTCAAA
AGACACCCCTCCTTCGCTGCGAACAACCTAAGCAGAAACCTACGCAGAAACCTAAGCAGCAGACTCAGTGGTTGCATGA
AGTGGTACTTTGCTGGGACCATCATGCAAAAAATATGATTGACTGTGATGATACAGAAGCA
>Rlaevigata_4
ATGTATTCAAATCAAACATTGGTAAAAGACCTGGCTCGCTCTTGGCCGGACCTGAAAAATGGGAACAATCAAAGGTTTT
GGAGAGAGCAGTGGGATAAACATGGCAAATGCTCAGAACAGACATATGCACAAACGCGATACTTCCAGCGCTCCTTCGA
GATGTGGAACCAATTCGATATTACTAATATCTTTACTGCCGCTGGCGTCGTATCATCGCCTGGGACAAAACCACGTATA
GTCAACGCGAGCGACTTAGAAGCACGCATTGAAGCGGTAACTCGTTCGATACCTGTCCTTCGGTGCAGAAAAGCCGCAG
TTCTCAGAGGCCAACCACCAGTTGATTTAATTTATGAAGTGGGCATTTGTTTCGATTATGTTGGAACAAATCCGTATCC
TTGTGACCGTTCACACGGGAGATGCACTGCAAATGATATTTACTTT
>Rlaevigata_7
ATCTCAGAGTTGATTGGCCGTCTGGAAAAGAATTGGCCATCTATGAGCTGCCCAAGCAGTAATGGTTACAGGTTCTGGT
CACACGAATGGCAGAAGCATGGCACTTGCTCCGAATCTGAACTTGATCAGAAAGATTACTTCCAAGCCGGTCTCAAGCT

CAAGGAAAAAGCAAACCTTCTCCAAGCCCTTAAAAAAGCTGGTAATTTAGTTCGTCTATACTCAATTGTTTATGACAAA
TTAATCAAGCTATAT
>Rlaevigata_10
ATGAACCAGTCCACTACACTTGTATCCAAACTGAAGATATCTTGGCCCAACGTGGAAAAAGTTGGGAAGGAAATGAATT
TTTGGACGGATGAGTGGAACAAACATGGGACATGTTCCGAGCAGAAATATAAGCAAACAGATTATTTCGAGCGGTCTCA
CGAAATGTGGAAAGAAAACAATGTCACTGATATTTTAGATAACGCTGGAATCAAACCAAATGGAACATTACTCCGGAAC
TACGTGGACATAGAAGGACCGATTAAAGCAGCAATTAACAACAAGACGCCTCTCCTCCGTTGCAAAAATGAGACAATGA
GCAATACTCCCCGCTTGCATGAAATTGTCATTTGCTATGCTCATGACGGTACTACTTTGACCGACTGTATCAGTAGCGA
GGCGACGTGCAAAAGGATAAATAAAATCAAGTTTTCTTTA
>Rlaevigata_28
ATGGCCCAAAACGGTCAATTGGAGAACAATCTTCGAAGAGCATGGCCCCAATTGATACCTTATTTTACAGATATGGACT
TCTGGGGAGATGAATACAATAAGCACGGTACTTGCTCGGAGAACAAGCTGGACCAGACGCAATATTTTCGGAAAGCTTA
CCGGATGTGGTCTCTGTACAGAGCAGATCAGCTGTTTCTAAACTCAGGAATCTACGGTGGACGTTACCATACTCGCACT
GCCCTTGAAAATGCCATTTGGCGAGGGACTAGAGGCTGGCCTCTGCTTAGCTGCTGGTCCAGGAACATAAAAGTAGCAG
GTGCCAAATATGAAAATGTTTGGTTTTTGGAGGAGGTCATCATTTGTTTTGATTATCTGGGGAGAACGTCATCGATTGT
GTTCGACGGACAAATTGCGGTG
>Rpersica_4
GAAGCAGCCGTCAAGTCCAAGATCGGTACCGATACAATACCTCTCATCCTGTGCAAAGAGGACACACTAACTACTGGAA
GCAGCACAACTGGATTAATTCTGAGGGAAATAGTTATCTGTTTTGATCACCAGGGAACAAATCTGGTCAACTGTACCCG
GCTGACAGATTGCAAGCGGCAGAAGAGCACAACTAAAGACAATATGATATACTACGTACCT
>Rxanthina_4
GATTTTGAAGCAGCCGTCAAGTCCAAGATCGGTACCGACACAAAACCTCTCATCCTTTGTAAGCAGGCCAAACTAACTC
GTGGGAGCACAACAACCAACTTCATTTTGAGGGAAATTGTGATCTGTTTCGATCACCAGGGGACAAATCTGGTCAATTG
TACCCGATCAACAGATTGTAACCAGAAGCAGAAGATATACTACGTT
>Rxanthina_6
ATGCTTTCAAATCAAACATTGGTAAACGACCTGACTCGCTCTTGGCCGGACCTGAGAAATGGGAACCATAAAAGGTTCT
GGATAGAGCAGTGGGATAAACATGGCCAATGCTCAAAACAGACATATACACAAACGCGATACTTCCAGCGCTCCATCGA
GATGTGGAACCAATTCGATATTACTAATATCTTTACTGCCGCTGGAGTCGTATCACCGCTACGGACACAACCAAGTATT
GTCAACGCGAGCGACTTAAAAGCGCGCATTGAAGCAGTAACTCATACGGCACCTGTCCTTCGGTGCAGAAAAGCTACAG
TGCAGAACGTTCAACAGCCAGTTGATTTAATTTATGAAGTGGGCATTTGTTACGATTATCCTGGAACAAAACTGATTCC
TTGCAACCCCAAACAAGGAAAATGCACTGGAAAAGATATTTACTTT
>Rxanthina_16
ATGGACCGCTCATTGGAATCCAAGCTGAATATCTCTTGGCCCAACGTGAAATTCCCGAATAATATCCCCTTTTGGGGTG
CGCAATGGAGAAAGCATGGGTCATGTTCCATGAGCACATTTAACCAAACAGAGTATTTCAAGCAAGCCGACAATCTTTG
GAACGCACATAACATCACCAATATCCTCATTACGGGTGGATCATACCAAATGGATCAGAACACGACTATAATACGGTA
GCTGGTCCCATCCAAACTGCAACCGGAAAGGTACCGCAACTTCGTTGCCTACAACTTTCGCAAGTGACTCAATTGTTGC
ATGAAGTAGTCCTTTGTTATGATCATAAAGGAACAACGCTGATCGACTGTAACCGTAAACAGGCACCATGCGATCCTAA
CATCCAAATAAAGTTTATCAAG
>Rxanthina_27
ATACTCACTAGTTCGTATTTGGTAAGCAAGCTGAGCACCTCTTGGCCCGATGTGAAAAATGCAAATGATAAAATTTTTT
GGAAGAAACAATGGGACAAGCATGGCACATGTTCCTTGCAGACATACGACCAAGCACAATACTTCGAGCGGTCATACAA
CATGTGGAAAGAAACCAACCTGACAAACACCTTGGATAGCCTCATCAAACAGACACCGAGACAACAGAACGTCACAGAT
ATAGAACAACTCATTCAAGGAGTAACTGCAACTGAAAAGAAACCCCTCCTCCGTTGTGAACAATCTCATAGTAATGCCA
GTAAGTCTCTCTTGAAAGAGATTGTCATTTGCTATGCACACAACGGGATTACTGTGATCGATTGCGTCAGTCAACCAAA
ATCCACCTTCGAATGCTATGACGGATTTTGGTTTCCT
>Rxanthina_33
ATGTCCGCGCACTATCGTCTTCAAACCATATTGCTCCCATCCTCGTGGAGAAGCTATCTTATCGGTGTCTCCAACACAG
ACTTCTGGGAGAAAGAGTATAACAAACATGGCACATGTTCGGAGCTCAATATGCCACAGACAGAATACTTCACCAAAAC
CCATTGGCTATGGATGGTCAACAACATTTATAGTATATTTTCATCTTCGGTCAACAGACAAGGATATCTAATCTTGCCG
GGTTACACCTATGACTACACCGATCTTCTTGCCGCTGTTCAACTAGGAATCGGTGGCGTCACTCCCGTGCTTACTTGCA
AGGTCATAAATCAAAACACATACGTTCTTCACGAAGTGGTTATTTGTTATGATGCCTTGGGGATCAACCGCATCCATTG
TGTGCGACAATCGAGTTGCTCTTTCGCTCCTAACGGACGTGTGTTATATCCT
>Rxanthina_36
ATGCGTCGACACAATCATCTCGAAACTCAATTGCTGTCATCCTCATGGCCAAGCTACACTGGTCTGTCCAACCTGCACT
TTTGGGAGTACGAGTATAACAAGCACGGCACTTGTTCAGAGAATAACCTCAAACAGACGGACTATTTCACCAGAGCCGA
CGCTTTGTGGAGGCGCTACAATGTTACCAATATGCTTTTAACATCGCGCCACCAAATCTCGCCGGGATCCTCCTATCGT
TACGCTGACATTTTCTATGCCATTCGACAAGGAATTGGGGGATACACTCCTTTGATTCTTTGCAAGCAGGATCCGGCAA

ATAATAATTGGATTCTGCACGAGGTGAGAATTTGTTTCGATCCCTTGGGGAATAACGTCATCGCTTGTCGCAGAAGATC
GAGATATAGTTGCTCTTACACTAACAGCGGGTTGATAGACTATCCTGAG
>Rarvensis_3
ATTAATAGCGACATTGAAGCTGCACTAATTAAAGCTTGGCCCAGTATTATGGGTAAACCAAACCAAAAATTTTGGGAAC
ATGAATGGAGAAAACACGGTTCATGTTCCACAGATTTATTACCAACTTCTAATGACTACCTACTGAAAGGAGTTTACCT
GTGGGAGCAAATCAAGTTTCATGATTTGATGGGTGTGGGTCCGGGTGCCCAATTTAAGCCAAATACTCAATATGATGCG
AACACGGTGCTGGCGGCCATCACAAGCAAATACAAGGTCAAAACAATGCTGACCTGTCAGGGTGGGAAATACTGGATG
TTAGGTTTTGTTATTTAGGACCTTGGGTACTGGTAGATTGCCTGGACACCCAACGACCTCCCAGTACAGTTTGTACAGG
TTTAATTTATTATCCTAGAATA
>Rarvensis_11
GCTCCTCCATTGGAAGCCAGACTGAAGATCTCTTGGCCCAACCTAGAATATGGGAGTGATGCATCGTTTTGGAAAAGAG
AGTGGGACAGACATGGCACTTGTTCGATGCAGACACTTACACAAACACAATACTTCGATCGAGCCCATAAAATTTGGGT
GGAGAATAATATTACTGACATCCTCCGACAAGCAAGCGTCGTATCGGGGACAACAAGAGACTACGCACAGATAGAGTTG
CCCATAAGAACAAAAACTCAAAAGATGCCACTCCTCCGCTGCGGAAAGACTCGGTTGTTGCATGAAGTCGTTCTCTGCT
GGGACCATGATGCAAAACGTATGATCGACTGTGATGCTTCAGAAACAAATTGCCCAATCTATAATATCGATGTTCTA
>Rarvensis_13
CACAAGTTCTGGAAGGAGTATGATCTCTATTCCATTTTGAAACAAAAAAATATCAAGCCGGGGCAGTCTCGATCGTATT
CACTGGCTGATTTTGAAGCAGCCGTCAAGTCCAAGATCGGTACCGACACAAAACCTCTCATCCTTTGTAAGCAGGCCAA
ACTAACTCGTGGGAGCACAACAACCAACTTCATTTTGAGGGAAATTGTGATCTGTTTCGATCACCAGGGGACAAATTTG
GTCAATTGTACCCGATCAACAGATTGTGACCAGAAGCAGAAGACAACTAAATCGAGTATGATATACTACGTT
>Rarvensis_16
ATAATCGACTATTTGGAAACAGATTGGCCGTCGCTGGCCCGCCCGAGCCGCAACAGTACGCAGCTTTGGGCCGAAGAAT
GGAAAACACATGGCACTTGCTCACAGTCTGCCCTTCATCAGTACAAGTACTTCGCGAACGGTTCGACCCTCAAAGCCTC
GGTAGACATCCTCAAAATGCTACAACTTGCTGGTACCTATTTTTTCATCTTTACCTCCCTCGTCATTTATATATATATA
TATATGTTATATATTATT
>Rarvensis_24
ATGTATTTCAATAAAACACTGGTAAATGACCTGACTCGCTCTTGGCCGGACCTGAGAAATGGGAACCATCAAAGGTTTT
GGGAAGAGCAGTGGGATAAACATGGCAGATGCTCGGATCAGACGTACGGGCAAACGCGATACTTCCAGCGCGCCTTCGA
GATGTGGAACCAATTCGATATTACTCATATCTTCAGTGCCGCTGGCGTCGTATCACCGCCAGGGACACAGCCAAGTATA
GTCAACGCGAGCGACTTAGTAGCACGCATTGAAGCAGTAACTCAAGCGATACCTGTCCTTCGGTGCAGAAAAGCTACAG
TGCAGAACGTTCAACAGCCAGTTGATTTAATTTATGAAGTGGGCATTTGTTACGATTATCTTGGAACAAATCTGTTTCC
TTGTAATCCCAAACAAGGAAAATGCACTGGGAAAGATATTTACTTT
>Rarvensis_26
ATGCTTAGTCAATATCGTCTCCGAACCAAAATGCTGCCATTCTCATGGCCAAGCTTCACTCGTAGATCCAACATGGGCT
TCTGGAAATATGAGTATAATCAACACGGCACTTGTTCGGAGAATAACCTCGCGCAGACGGACTATTTTTACAAAGCCTA
TGCTCTCTGGATGCGCTACAATGCAAACTTTATATTATATGCATCTACAGTTATACCAGGCCACAAGATTGTGCCGGGA
TACCTCTACCATTACGCTGACCTTCTAAACTCCGTTCAACAAGCAATTGGTGGATTCACTCCTTCGCTTATGTGCAAGC
ACGAGCCTGCAAATAATACTTGGATTCTGCACGAAGTCATCATATGTTTTGATGCCATGGGGAACAACGTCATCAATTG
TGTTAGAGGATCTAGTTGCTCTGCCACTACCACCGGGTATATACACTATCCTTTGCAA
>Rarvensis_36
ATCTCAGAGTTGATGGGCAGTCTGGAAAAGAGTTGGCCATCTATGAGCTGCCCAAGCAGTAATGGTTACAGGTTCTGGT
CACACGAATGGGAGAAGCATGGCACCTGCTCCGAATCTGAACTTGATCAGAAAGATTACTTCCAAGCCGGTCTCAAGCT
CAAGGAAAAAGCAAACCTTCTTCAAGCCCTTAAAAAAGCTGGTAATTTAGTTCGTCTATACTCAATT
>Rminutifolia_20
GTTGTCTATTGCTTGAATGCTCAGGCTCCTCCATTGGAAGCCAGACTGAAGATCTCTTGGCCCAACCTAGAAATTGGGA
GTGATGCATCGTTTTGGGCAAGAGAGTGGGACAGACATGGCACTTGTTCGATGCAGACACTTACACAAACCCAATACTT
CGATCGAGCCCATAAAATTTGGGAGGAGAATAATATTACTGATATCCTCCAACAAGCTGGCATCAGATCGGGGGCAAAA
AAAGACTACGGACAGATAGAATTACCCATAAAATTAAAAACTCAAAAGATACCCCTCCTTCGCTGCCACAAACCTAACC
CGCAGACTCGGTGGTTGCATGAAGTGGTACTTTGCTGGGACCATGCTGCAAAAAATATGATCGACTGTAATGCTGGAGA
AACAAATTGCGGAAAAATTAAAATCGATGTTCTA
>Rminutifolia_33
TCTCTTTACTCGCAATATGTATTTAATGCCCAGATGCTTTCAAATCAAACATTGGTAAACGACCTGACTCGCTCTTGGC
CGGACCTGAGAAATGGGAACCATAAAAGGTTTTGGATAGAGCAGTGGGATAAACATGGCCAATGCTCACAACAGACATA
TACACAAACGCGTTACTTCCAGCGCTCCATCGAGATGTGGAACCAATTCGATATTACTAATATCTTTACTGCCGCTGGA
GTCGTATCACCGCTACGGACACAACCAAGTATAGTCAACGCTACCGACTTAAAAGCGCGCATTGAAGCAGTAACTCATA
CGGCACCTGTCCTTCGGTGCAGAAAAGCTACAGTGCAGAACGTTCAACAGCCAGTTGATTTAATTTATGAAGTGGGCAT
TTGTTACGATCATCCTGGAACAAAACTGATTCCTTGCAACCCCAAACAAGGAAAATGCACTGGGAAAGATATTTACTTT
>Rmajalis_2

CAGCGCCAAATCCACCTCAAGCCCCACCTCCAGCGCCCACTCCACCTCCTGATTCCTTCATTTTGGTCATTGTGTGGCC
CAATACCTTCTGTCTTTTTGAGTCATGTCAGCTGCATCCACAATCTTTTACACTACAAGGATTGTAGCCACAGGCGAAT
GGGAAATCCTTGGTGGACTGCTGGTTCAGAAATGGATGATAGCACCTTAAATGGCAAGAAAGATGATTTGAACAAGTAT
TGGCCAGACTTGACGCATTCCAAGTTTGAAGAGAGTAAAAGCTTTTGGATACATGAGTGGGAAAAACATGGTCGTTGCT
CTGCAAAGAGTCCTGCTAATTACTTGAGCCTGGTTTTTGATCTGATGAAAAAACATGATGTGGAACAGATATTTAAAAA
CAATGGTAAGAAA
>Rmajalis_12_
CTTCTCACGCGCTCACAAGTTGTGGCAGGAATTGCAGGCCAAGCATTTGCCGATGAAAAAATCGTACCAGCAGCTGACA
AAACTTACACCTACACCCAGCTCGAGCAAGCTATTGGAAAACGATTTGGCAGTAATTGGAAGGTTGTCATTCTTTGCAG
AAAGGTCAATCTCTCACCTGGTGGTCGTGGACCAAAAAAAATTCTACTTCTTTTGCATGAAATCGTATTCTGTTTCGAT
AACAAAGCAACGGACAAGAAGAGTTGCAACAGAAAAACAAATTGTGAGAACGCGAAAACTGTAACCTTCATAAAT

Supplementary File 3
>Rmoschata17_14
TCCTATGAATATTTTAAATTTGTGGTACAATGGCCACCAGCCCACTGCAGAGTTAGAAAATGCACACCCCAAGCCTTGC
AAAAGAAAATTTACACCATCCATGGCCTATGGCCGAGTAATTATTCTAAAGCTGTCGTGTATAAGTGTCCCGGATCGTC
TTTTCAAAATCCGGTAAATGCTCCTCCATTGGAAGCCAAACTGAAGATCTCTTGGCCCAACCTAGAACTTCCGAATGAT
GCAATGTTTTGGGCACTT
>Rpersica_25
ATGAGTGGTGGAGCCTATCATTTTTTCCAGTTTGTGCAACAATGGCCACCAACTCTTTGCAAAATTAAAAATTGCGTTG
TCCCAAACGCACCCAGAAACTTCAGCATCCACGGTTTGTGGCCAAGTAATGCTTCATACAGTTCGGGGGGTTTGTGTCC
TGGGGCGCGATTTGACTACGGTTTGGCAGGTGTGTTAGCACCTCTATTACAAAAATCTTGGCCGGACGTGGAAAAAGGT
AACGACACGAAATTTTGGAAACTTGAGTGGGATAGGCATGGAAAATGTTCCGATCAGACATTCGACCAAAGAAGATACT
TCACACAATCCCACAACATTTGGACGAGATATCATATTCTTAATATCCTTAAAAACTCTCAAATAGAACCAAATGGTAG
TTTTTACTCTTACACATCAATAGAATCCTCCATCCGAAGTGCAACTCAAACGAGACCCCGTATTCGAACAAGGAATCTT
CCAACTGATCCTAACAACAATACGTATGCTCACTACTTGCACGAAATAGTCCTTTGTTTTAAGTATGATGGGGTGACAC
CGGACAACTGCTCCGCGGTAGTCGGACCACCAGGAGATGTCAAATTTGAA
>Rpersica_14
TATTGCTTATTCTACGTTCTTTCATACGCAGTAATATGCATAGGATCTTATGATTATTTCCAATTTGTCCTACAATGGC
CACCAACGGTGTGTAGAATGAAACCATGCATCCAGGCAAATCCGCCCAGAAACTTCAGCATCCATGGTCTGTGGCCAAG
TAATTATTCATTCAATATGGTGCCAAGTGTCTGCGGTGGGGCGGCATACGTCCCCGCTGTGACTAATGCGTTAGCACCT
TTACTAAGAGTCTCTTGGCCGGACGTGGAAAAAGGCAACGAACAGGACTTCTGGAAACTTGAGTGGGACAGACATGGCA
AATGTTCCGAACAAACATTAAACCAAACAAGATACTTCACACAATCCCACAACATTTGGAATCGATATCATATTCTTGA
TATCCTTAAAAACTCTCAAATAGAACCAAACGGTAGTTTGTACCTTTACACAGATATACAATCCTCCATCCGAAAAGCA
ACTCAAATGACACCTCGTATTCGAACGAAGCAACTTTTAACCGATCCTAAGAACGCATCATCGTTTGCTTTCTACTTGC
ACGAAATGGTCCTATGTTATAAGTATGACGGGGTGACACCGGAAAACTGCTCGGCAGCAGGCTCACGGCAACAATATGT
CAAATTTGCTATAAAA
>Rrugosa14_28
TTTGTGCAACAATGGCCACCAACTTTTTGCAAAATTAAAAACTGCGTCCCAAACACACCCAGAAACTTCACCATCCATG
GTTTGTGGCCAAGTAATGCTTCATACAGTTCGGCGGGTTTGTGTCCTGGAGCGGTATTTAACTTCAATTTGGCAGGTGC
GTTAGCACCTCTATTACAAAACTCTTGGCCAGACCTGGAAAATGGTAACGCCACGAGATTTTGGAAACTTGAGTGGGAT
AGACATGGCAAATGTTCCGACCAGACATTAGACCAAAGAAGTTACTTCACACAATCCCACAACATTTGGACTAGATATC
ACATTCTTGATATCCTTAAAAACTCTCAAATAGAGCCAGATGGTAGTTTGTACTCTTACACATCAATAGAATCCTCCAT
CCGA
>Rchinensis2_16_Rchinensis3_17_Rchinensis3_19
ATGTCTACAGGGTATTACGAATATTTCAAACTTGTGCAACAATGGCCACCAACCACTTGCCAAAATGCAAACTGCCGCA
GAGTACCGCCACCTCGCCTCTTTACCCTCCATGGGTTTTGGCCATCTAATTATTCAAACAATGTCGTGGCTAATTGCAC
TAACGCAATATTTCAACGGATGGATCGCTCATTGGTATCTAAACTGAATATATCCTGGCCCAATGTGAAATATCCGAAT
AATATCCAGTTTTGGGATAAACAGTGGAGAAAACATGGCTCATGTTCCGTGCACACGTTTAACCAAACAGAGTACTTCA
CGCAAGCGGATAATCTTTGGAACTCACACAACATTACTGATATTCTTATAACTGGTGGGATCAAGCCAAATGGATCAGA
ATATGCATACGATACGGTAGAGCGGCCCATCCAAATTGCAACCGGGAAGGAACCCGAACTTCGTTGCGCACCATCCTCC
CTTGGAAGGCAATTGTTGCATGAAGTAGTACTTTGTTATAATCATAAGGGAACAACGCCCATCGACTGTAACCCTTTAC
ATTCAACCTGCGATCGTAACTTCCAAATAAAGTTTATT
>Rchinesis1_3_Rchinensis2_27
ATGGCAATGTTGAAATCATCGTTCGCTTTAATTGCTCTTGCTTTTGCTCTCTGTTTCACTATGAGCATTGGTTCCTATG
AATATTTTAAATTTGTGGTACAATGGCCACCAGCCCACTGCAGGGTTAGAAAATGCACACCCCAAGCCTTGCAACAGAA

AATTTACACCATCCATGGCCTATGGCCGAGTAATTATTCTAAAGCTGTTGTGTATAATTGTCCGGGATCGTCTTTTCAA
AATCCGGCTCCTCCATTGGAAGCCAAACTGAAGATCTCTTGGCCCAACCTAGAACGTCCGAACGATGCAATGTTTTGGG
CACTTGAGTGGGACAGGCATGGCAAGTGTTCGGAGCAGACATTTACACAAACCCAATACTTCAATCGAGCCCATGAAAT
TTGGGTGGGGAAGAATATTACTGACATCCTCCAGAGAGCTAGCATCTTATCGGGGAGACAAAAAGACTACGGAGTTATA
GAATTAGCCGTACGATCAAAAACTCAAAAGACACCCCTCCTTCGCTGCGAACAACCAAAGCAGAAACCTACGCAGAAAC
CTACGCAACCTACGCAGCGGACTCAGTGGTTGCATGAGGTGGTACTTTGCTGGGACTATCATGCCAAAAATATGATTGA
CTGTGATGATACAGAAGCAACATGTCAAGATACTTTTCCAATCGATATTCTG
>Rmultiflora_8
CAATACTCATTTGTTTTTGTGTCAAGGCCTCATTTTGGAATTTTAATGTTCGTTTGTTTCTTGTCACAATTAATTTCCA
CAGGATCGTATAAATATTTCAAATTTGTTGTACAATGGCCACCAGCCCACTGCAGTGTTTTTAAATGCCACAATAGCAA
CCCGCCAAAGATATACACCATCCATGGCCTATGGCCAAGTAATCGTTCTAATGCAGCTCGCAGTAAGTGCCAGGGATCG
TCATTTCAACAACCGGCTCTTCCATTGGAAGCCAAACTGAAGATCTCTTGGCCTAACCTAGAAAATTTGAGTGATGCGC
AGTTTTGGGAACTTGAATGGAACAGACATGGCAAGTGTTCGGAGCCGACATTTACGCAAACCCAATACTTCAATCGAGC
CCATGAAATTTGGATGATGGATGACATTAATATTACTGATATCCTCCAAAATGTTCACATCGTATCAGGGAAACAAAAA
ACCTACGCAGAGATAGAATTTCCCATTGAATCAAAAACTCAAAAGACAATCCTCCTTCGCTGCCTAAACCCTCAGAAGT
TGCATGAAGTGGTAATTTGCTGGGACCATGCTGCAAAAAATATGATCGACTGTAATCGTACAGAAGCAACATGCTCAAA
CAGCAGTCTAATTGATGTTCTA
>Rmultiflora_4_
TCACAATTAATTTCCACAGGGTCCTATGACTATTTCAAATTTGTTGTACAATGGCCACCAGCCCACTGCAGTGTTTTTA
AATGCCACAATAGTAACCCGCCACACATTTACACCATCCATGGCCTATGGCCAAATAATCGTTCTAAGGCAGTCGGGAA
TAAGTGCCAGGGATCGTCATTTCAAAAGCCGGCTCCTCCATTGGAAGCCAAACTGAAGATCTCTTGGCCTAACCTAGAA
AATTCGAGTGATGCGCACTTTTGGGAACTTCAGTGGAACAAACATGGCAAGTGTTCGGAGCCGACATTTACGCAATCCC
AATACTTCGATCGAGCCCATCAAATTTGGATGATGGAGAATATTAATATTACTAGTATCCTCCAAAATGTAAACATCGT
ATCAGGGAAAACAAAAAGCTACGAAGAGATAGAATTTCCCATTGAATCAAAAACTCACAAGACACCACTCCTTCACTGC
ATAAACACTCAGAAGTTGCATGAAGTGGTACTTTGCTGGGACCATGCTGCAAAAAAATTGATCGACTGTAAACATACAG
AAGCAACATGCTCAAGGAATAGTCCAATTGAGATTCTA
>Rmajalis_20
TTTTTTTTTTTCAACACAATAATGTCTCTAGGTTCATATGATCATTTTAAATTTGTACAACAATGGCCACCGGCCGTCT
GTGACACGTCTGGCTGCAACAGATCGGGGTTATCACGCTTCACGATCCATGGCCTTTGGCCAAATAATAAAACGTATGT
GAAAAATCAACCGACTTGTCTTACCAATCAGACCAACTCATTTAAGGCTGCGATACTCACTAATTCGAATTTGGTAAGC
AAGCTGAGCACCTCTTGGCCTGATGTGAAAAATGCAAATGATAAATTTTTTTGGAAGAAACAATGGGACAAGCATGGCA
CATGTTCCTTGCAGACATACGACCAAGCACAATACTTCGAGCGGTCATACAACATGTGGAAAGAAACCAACCTGACAAA
CACCTTGGATAGCCTTATCAAACAGACACCGAGACAACAGAACGTCACAGATATAGAACAACTCATTCAAGGAGTAACT
GCAACTGAAAAGAAACCCCTCCTCCGTTGTGAACAATCTCATAGTAATGCCAGTAAGTCTCTCTTGAAAGAGATTGTCA
TTTGCTATGCACACAACGGGATTACTGTGATCGATTGCGTCAGTCAACCAAAATCCACCTTCGAATGCTATGACGGATT
TTGGTTTCCT
>Rrugosa14_13
ATGTCTCTAGGTTCATATGATCATTTTAAATTTGTACAACAATGGCCACCGGCCGTCTGTGACACGTCTGGCTGCAACA
GATCGGGGTTATCACGCTTCACGATCCATGGCCTTTGGCCAAATAATAAAACGTATGTGAAAAATCAACCGACTTGTCT
TACCAATCAGACCAACTCATTTAAGGCTGCGATACTCACTAATTCGAATTTGGTAAGCAAGCTGAGCACCTCTTGGCCC
GATGTGAAAAATGCAAATGATAATTTTTTTTGGAAGAAACAATGGGACAAGCATGGCACATGTTCCTTGCAGACATACG
ACCAAGCACAATACTTCGAGCGGTCATACAACATGTGGAAAGAAACCAACCTGACAAACACCTTGGATAGCCTTATCAA
ACAGACACCGAGACAACAGAACGTCACAGATATAGAACAACTCATTCAAGGAGTAACTGCAACTGAAAAGAAACCCCTC
CTCCGTTGTGAACAATCTCATAGTAATGCCAGTAAGTCTCTCTTGAAAGAGATTGTCATTTGCTATGCACACAACGGGA
TTACTGTGATCGATTGCGTCAGTCAACCAAAATCCACCTTCGAATGCTATGACGGATTTTGGTTTCCT
>Rminutifolia_34
ATGTCTCTAGGTTCATATGATCATTTTAAATTTGTACAACAATGGCCACCGGCCGTCTGTGACACGTCTGGCTGCAACA
GATCGGGGTTATCACGCTTCACGATCCATGGCCTTTGGCCAAATAATAAAACGTATGTGAAAAATCAACCGACTTGTCT
TACCAATCAGGCCAACTCATTTAAGGCTAAGATACTCACTAGTTCGAATTTGGTAAGCAAGCTGAGCACCTCTTGGCCC
GATGTGAAAAATGCAAATGATAAATTTTTTTGGAAGAAACAATGGGACAAGCATGGCACATGTTCCTTGCAGACATACG
ACCAAGCACAATACTTCGAGCGGTCATACAACATGTGGAAAGAAACCAACCTGACAAACACCTTGGATAGCCTCATCAA
ACAGACACCGAGACAACAGAACGTCACAGATATAGAACAACTCATTCAAGGAGTAACTGCAACTGAAAAGAAACCCCTC
CTCCGTTGTGAACAATCTCATAGTAATGCCAGTAAATCTCTCTTGAAAGAGATTGTCATTTGCTATGCACACAACGGGA
TTACTGTGATCGATTGCGTCAGTCAACCAAAATCCACCTTCGAATGCTATGACGGATTTTGGTTTCCT
>Rmoschata08_18
ATGTTATTGAATCTAATCAGCTGCTCTTATATAAGTTTTGACATTCTTCCTTTCTGTTTTTTTTTTGTTTTTTTTTTTT
TCAACACAATAATGTCTCTAGGTTCATATGATCATTTTAAATTTGTACAACAATGGCCACCGGCCGTCTGTGATACGTC
TGGCTGCAAGAGATCGGGGTTATCACACTTCACGATCCATGGCCTTTGGCCAAATAATAAAACGTATGTGAAAAATCAA

CCGACTTGTCTTACCAGTCATGCCAACTCATTTAAGGCTACGATACTCACTAGTTCGAATTTGGTAAGCAAGCTGAGCA
CCTCTTGGCCCGATGTGAAAAATGCAAATGATAAAGATTTTTGGAACAAACAATGGGACAAGCATGGCACATGTTCCTT
GCAGACATACGACCAAGCACAATACTTCGAGCGGTCATACAACATGTGGAAAGAAACCAACCTGACAAACACCTTGGAT
AGCCTCATCAAACAGACACCGAGACAACAGAACGTCACAGATATAGAACAACTCATTCAAGGAGTAACTGCAACTGAAA
AGAAACCCCTCCTCCGTTGTGAACAATCTCATAGTAATGCCAGTAAGTCTCTCTTGAAAGAGATTGTCATTTGCTATGC
ACACAACGGGATTACTGTGATCGATTGCGTCAGTCGACCAAAATCCACCTTCGAATGCTATGACGGATTTTGGTTTCCT
>Rmajalis_11
ATGAGTATTTTTACATTGTTTCTCCCCCCAAACTCCACAGGCGGATACTATGAATATTTCAAGATGGTGGAACAGTGGC
CGTTAGGATATTGCACAGGTCGACCAGATCCTTGCAACAAAACTCCACCAAACATGTTCACCATCCATGGTTTTTGGCC
CAGTAATTATTCAACAAATCCAGCAGATACATGCAAAGGGACTTCGTTCAACGAGACTATGATGCGTAAAAATCAAACA
TTGGTATCCGCACTACAGAGAGTATGGCCAGACCTGGCGGACGGAGATGATTTTGGGTTCTGGAGACGTCAGTGGGACA
AACATGGGAAATGTTCGGAGCAAACATTTCAGCAAATGCAATATTTCGAGCGTGCAAATGTTATTTGGAACAAACCGAA
TATGACTTTGATCTTTAAAAATAATGGAATCCTAGCAGGGGGGGCAAAATATAACTCCACAGACTTGGTATCACGCATT
GAAAAGGTGCTTCGGACCACGCCCCTACTTCATTGCAAATGGGTAACGGATCCTAAT
>Rrugosa14_1
TACACCCATCTTGTCCAAATGTGGCCGCCGGGTCATTGTCATTTCCCACCACATTGCACTGCACCGATACCTCGGAACT
ACTTTAGTGTGCATGGCTTATGGCCAAGTAATCATAGTGTGCCCAAGATTACATACAAGATGTATATGTTCCCTTCTCC
TGAACACTGGTTTAAACTT
>Rrugosa14_2
TACGACTACTTTCAGATGGTATACCAATGGCAGCCAGCCTACTGTGTTGGTAGAAAAGATCCTTGCAGAATAAAACCAT
CAAAAATCTTTACCATTCATGGTCTATGGCCAAGTAATTTTTCAGACATTCCAGGGGATAAGTGCAAGGGGGCACTCTT
TAACGACAGTCTG
>Rrugosa14_3
GCAGAGCCATACGAATACCTACAGTTTGTCTTGCAATACGCTAGAGGTTCCTGTGTTAACGTCAAGAAATGCATTCGAC
CGGCGAGCTTGCCGGCCAAATTCACGGTTCATGGAATTTGGCCCACGAATATT
>Rrugosa14_7
TCCTTCAATGGCCAACTACATTATGCATTAACCAAATCTGCATCCCAAATATCCCGCTGGACTTCACTCTACATGGGTT
ATGGGCCACCAATATCTCTGGCCAAAACAACCCTTGTGTTG
>Rrugosa14_11
GGCAGGTCAGATTGTATCCCAAATCCCCCGCAGAATTACTTTACTATTCACGGGCTTTGGCCATCCAATTACTCGACTC
CTGGACAGGATTGCCATGGCACACCTTTTAGTGGAACTGAGGTATTC
>Rrugosa14_12
GATTGTGTCCGGAATCCCCCGCGGAATACTTTTACAATTCATGGCCTTTGGCCAAGTAATTACACGACACCTGAAAAGC
CATGCGTTGGCACACCCTTTAGTCCA
>Rrugosa14_23
TACTTCCAGTTTGTCCAGCAATGGCCGGCTACATTCTGCTATCAAAATTCGGATTGTGTCCGGAATCCCCCGCGGTATA
CTTTTACCATCCACGGCCTCTGGCCAAGTAATTACACGGCACCTGAAAAGCCATGCGTTGGCACACCCTTTAGTCCA
>Rrugosa14_24
ATGAAGTCCATCCCCTCAAAAATGGTCAAGCTTCTAATACTAGCACTAGTAGGATCATCATGTCTTTCAGTCCTTTGTG
CTGCAGAAGATTTTGATTTCTTCTACTTTGTTCAGCAGTGGCCAGGATCATTCTGTGATACACAGAAAAGTTGCTGCTA
TCCAACCACAGGGAAGCCTGCAGCAGATTTTGGGATTCATGGGCTCTGGCCAAATTACAAGGATGGTTCATACCCCTCA
AACTGTGATCCAAACAACCCCTTTGATCAATCTCAG
>Rrugosa14_25_Rrugosa19_25
GCAGAGCCATACGAATACCTACAGTTTGTCTTGCAATACGCTAGAGGTTCCTGTGTTAACGTCAAGAAATGCATTCCAC
CGGCGAGCTTGCCGGCCAAATTCACGGTTCATGGAATTTGGCCCACGAATATTTCC
>Rrugosa14_29_Rrugosa19_29
ATGGCTGCAGTAACAGTAGCATCAGTCATTGTGCTATTTTTCCTTCCACTTGGTGTGATCATAAATGCGGTCGGAGAGG
AAGCCGGACAGCCGTATTATGAGTACCTGCAATTTGTACAGCAATATCCCAGAACAGTGAATCGAAGGTCAACATTAAA
CAATTTCACCATTCATGGCCTATGGCCTAGCAATCGCAGCAATAATCATGTTATATTTTGTGGTCAAAATATCAGCGTG
AATACACTTTTAAGGCAG
>Rrugosa14_38_Rrugosa19_34
TACTTCCAGTTTGTCCAGCAATGGCCGGCTACATTCTGCCATCATAATCCGGATTGTGTCCGGAATCCCCCGCGGAATA
CTTTTACAATTCATGGCCTTTGGCCAAGTAATGACACGACACCTGAAAAGCCATGCGTTGGCACACCCTTTAGTCCA
>Rrugosa19_7
TTTGTCCAGCAATGGCCGGCTACATTCTGCTATCAAAATCCGGGTTGTGTCCGGAATCCCCCGCGGAATACTTTTACCA
TTCATGGCCTTTGGCCAAGTAATTACACGACACCTGAAAACCCATGCGTTGGCACACCTTTTAGTCTAGCTGAGGTATT
GCATGCATGGAATAAATTTCATTTT
>Rrugosa19_10

ATGTCTACAGGGTATTACGAATATTTCAAACTTGTGCAACAATGGCCACCAACCACTTGCCAAAATGCAAACTGCCGCA
GAGTACCGACACCTCGCCTCTTTACCCTCCATGGGTTTTGGCCATCTAATTATTCAAACAATGTCGTGGCTAATTGCAC
TAACGCAATATTTCAACGGGTATATAGTGTTATGTTCTTGCTTTCGCTTTTTATTTAT
>Rrugosa19_11
TTTCAAACCCTAAAAGATAGTTTCACGTTATTTAACATGCATTTTGATGAATCGATGCAGTGGCCTGGAGCATATTGCG
ACACAAAGCATAGTTGTTGCTATCCAAAGTCAGGGAAGCCTGCAGCAGATTTCGGCATTCACGGTCTCTGGCCAAACTA
CAAGGATGGCTCTTACCCTTCAAACTGTGATCCAGACAGTGTCTTCGACAAATCTGAGGTACACGACACCTATACTTTA
ATCAACATATATATAACC
>Rrugosa19_21
ATTCTCTGTGCTTTTGGGGTTGCTAATGCAGCTAACACCTACGATTATCTTCTACTAGTCCTTCAATGGCCAACTACAC
TATGCATTAACCAAAACTGCATCCTGAATCTCCCGCTGGACTTCACTCTACATGGGTTATGGGCCAGCAATATCTCTAG
C
>Rrugosa19_37
CTTCTCTCTGCGTGGGGGGCAGCTGATGCAGCCAATCCCTACGACTACCTCCAATTCGTCCAACAGTGGCCTGCTACAT
TCTGTTCCGGCAGGTCAGATTGTATCCCAAATCCCCCGCAGAATTTCTTTACAATCCACGGC
>Rrugosa19_45
ATGAGTAAGAAATGGAATCCATTTTATGTATTTTTTAACATTCTTTTCCCCTCGGAATATCTTTCTACAGCGAGCTACG
ATTATTTTCAATTTGTGCAACAATGGCCACCAAACTTCTGCTATGGTAGATATCCTGGTTGCAATAACACGCTATCATT
CACCATTCATGGCTTGTGGCCAAATAATATTTCTCAAGTTTATTACAATCATAATTGTCAAGGGAACGGATTTGACAAT
CAGGTACATATG
>Rrugosa_19
TTTGTGCAGCAATGGCCACCAAACTTCTGCTACGGTAGATATCGTCCTTGCAATAGGCAATCATTCACCATTCATGGCC
TGTGGCCAAATAATTTTTCACAAGTTAATTACAATCATATTTGTTCAGGGGCCGCATTTAAAAATCAG
>Rodorata_gigantea_9
AATTCTGCTTCAGGTTCTGTGAGACTATGCTTGTTTCTGCTAGTATTGGCATCGATGGCAACCTCCGTCAACTCACAAT
ATCAGTACACCCAGCTTGTCCAAATGTGGCCACTGGGTCATTGTCATTTCCCACCACATTGCACTGCACCGATACCTGT
GAATTACTTTAGTGTCCATGGCTTATGGCCAAGTAATCACAGTGTGCCCCAAATTAGATGCAACAATGCATATCTTCCC
TTCTCCGGG
>Rodorata_gigantea_20
ATGGGGATCATGCAACTCGCCCTGCTCCTTCTCTCTGCGTGGGGGGCAGCTGATGCAGCCAATCCCTACGACTACCTCC
AATTCGTCCAACAGTGGCCTGCTACATTCTGTTCCGGCAGATCAGATTGTATCCCAAATCCCCCGCAGAATTTCTTTAC
AATCCACGGCCTTTTTGTTTTTGGCAAAGAGTATTTGAGAGGGGGCCGGGGAGGC
>Rodorata_gigantea_25
CAATTTGTACTCCAGTACCCACCTGGAGTTTGCTATGACAGATCAAAAGGTTATTGCATCTTACCTCTGCCAACCAAGT
TTCATGTACATGGAATATGGCCTTCCAACTTCTCCGATATAGATGTCACTTGTGGTCAAGCACTGACGAATAACCCCTT
TGATAACGCGCAGGTGTGTATG
>Rodorata_gigantea_29
TACTTGTTGTTTGTTCAACAGTGGTCCAAGACTGTTTGTGGTGACAGCTGCAAAGCTCCCTCTCTAGTTTTTACAATAC
ATAGGCTCTGGCCCTCCAACCACACTAGCCCTCAGCTGTCGTGCACTGGT
>Rodorata07_1
ATGGGCTGCATGAGAATTTTGGATACACGAATGAGACTTGCTTATGGCACCTATGATTACCTTCTACTAGTCCTTCAAT
GGCCAACTACATTCTGCATTAACCAAAACTGTATCCCAAATCTCTCGCTAGACTTCACTATACATAGGTTATGGGCCAG
CAATATCTATGGCCAAAACAAACCTTCT
>Rodorata07_2_Rodorata_gigantea_40
CAATTTGTACTCCAGTACCCACCTGGAGTTTGCTATGACCAATCAAAAGGTCATTGCATCTCACCTCTCCCAACCAAGT
TTCATGTACATGGAATATGGCCTTCTAACTTCTCCAATACACTT
>Rodorata07_4
ATGAGACTATGCTTGTTTCTGCTAGTATTGGCATTGATGGTACCCTCCGTCAACTCAAAATATCAGTACACCCAACTTG
TCCAAATGTGGCCACTGGGTCACTGTCATTTCCCACCACATTGCACTGCACCGATACCTCGGAACTACTTTAGTGTGCA
TGGCTTATGGCCAAGTAATCACAGTGTGCCCAAGATTACATGCAAGAAT
GTATATGTTCCCTTCTTCGGAACACTG
>Rodorata07_101
ATGGGGATTAATGTCATCCTGATTCTTCTCTGCCTTATCAGCAACGTTGCTAGTGCAGCCAACACATACGATTATCTTC
AACTAGTACTGCAATGGCCAAACACGTTCTGCATCAGTAACCAAAAGTGCATATCAAATCTCCCACTAGACTTCACCAT
ACATGGGTTATGGCCTAGCAATTTCTCCGGCCAAAATAACCCATGT
GTGGGTGCGCCATTTAATAGAGCTCAG
>Rodorata07_15_120_1

GAATACCTACAGTTTGTCTTGCAATACGCTAGAGGTTCCTGTGTTAACGTCAAGAAATGCATTCCACCGGCGAGATTGC
CGGCCAAATTCACGGTTCATGGAATTTGGCCCACGAATATT
>Rodorata07_16_Rodorata_gigantea_24
TTTCTTTTCGCAATAATGTCTACAGGGTATTACGAATATTTCAAACTTGTGCAACAATGGCCACCAACCACTTGCCAAA
ATGCAAACTGCCGCAGAGTACCGCCACCTCGCCTCTTTACCCTCCATGGGTTTTGGCCATCTAATTATTCAAACAATGT
CGTGGCTAATTGCACTAACGCAATATTTCAACGGGTATATAGT
>Rodorata07_18_Rodorata_gigantea_16
ATGGCACAAAATAATATGGCTTACGTCCTCATGACCTGCACTACTGTTGCCTTACTCTTTACTCTGGCCTCCTCGTATA
CCGCCTACGAATACTTCTTGTTTGTTCAACAGTGGTCCAAGACCGTTTGTGGTAACAGCTGCAAAGCTCCCTCTCCAGT
TTTTACGATACATGGGTTGTGGCCCTCCAACCACACTGGCCCTCAGCTGAAGTGCACTGGTGCAGCATATAATCCCACA
GAG
>Rodorata07_19
CCATACGAATACCTACAGTTTGTCTTGCAATACCCTAGAGGTTACTGTGTTAACGACAAGAAATGCATTCCACCGGCGA
GCTTGCCAGCCAAATTCACGGTTCATGGAATTTGGCCCACGAATATT
>Rodorata07_20
ATGGCTGCAGTAACAGTAGCATCAGTCATTGTGCTATTTTTCTTTCCACTTGGTGTGATCATAAATGCGGTCGGAGAGG
CAGCCGGACAGCCGTATTATGAGTACCTGCAATTTGTACAGCAATATCCCAGAACAGTGTATCCAAGCTCAACATTAAA
CAATTTCACCATTCATGGCCAATGGCCTAGCAATTTCAGCAATATTAATCCTGTTATATTTTGTGGTCAAAATAGCAGC
>Rodorata07_24_Rodorata_gigantea_42
ATGTCTCTAGGTTCATATGATCATTTTAAATTTGTACAACAATGGCCACCGGCCGTCTGTGATACGTCTGGCTGCAAGA
GATCGGGGTTATCACGCTTCACGATCCATGGCCTTTGGCCAAATAATAAAACGTATGTGAAAAATCAACCGACTTGTCT
TACCAATCGGGCCAACTCATTTAAGGCTACG
>Rodorata07_28
TACTTGTTGTTTGTTCAACAGTGGTCCAAGACTGTTTGTGGTGACAGCTGCAAAGCTCCCTCTCCAGTTTTTACAACAC
ATGGGCTCTGGCCCTCCAACCACACTAGCCCTCAGCTATCGTGCACTGGTGTAGCATATAATCCTACAACG
>Rodorata07_29_Rodorata_gigantea_15
TTTGTGCAGCAATGGCCACCAAACTTCTGCTACGGTAGATATCGTCCTTGCAATAGGCAATCATTCACCATTCATGGCC
TGTGGCCAAATAATTTTTCACAAGTTAATTACAATCATATTTGTTCAGGGGCCGCATTTAAAAATCAG
>Rodorata07_37
TTTGTACTCCAGTACCCACCTGGAGTTTGCTATGACACATCAAAAGGTTATTGCATCTCACCTCTGCCAACCAAGTTCC
ATGTACATGGAATATGGCCTTCTAACTTCTCCGATATACATGTCACTTGTGGTCAAGCACTGAAGAATAACCCCTTTGA
TAACGCGCAGGTGTGTATGTGT
>Rodorata07_39
AAAATTTACACCATCCATGGCCTATGGCCGAGTAATTATTCTAAAGCTGTTGTGTATAATTGTCCGGGATCGTCTTTTC
AAAATCCG
>Rmoschata08_7
GCAGAGCCATACGAATACCTACAGTTTGTCTTGCAATACGCTAGAGGTTACTGTGTTAACGTCAAGAAATGCATTCCAC
GGGCGAGCTTGCCGGCCAAATTCACGGTTCATGGAATTTGGCCCACGAATATTTCC
>Rmoschata08_12
TGTTCTAACACTTGTCATTGTTTTTGCGTCACGCCTCACTCAACAATTTTAATATTTGTTTATTTCTTCTCACAATTAA
AATGTACAGGATCCTATGAATATTTTAAATTTGTGGTACAATGGCCGCCAGCCCACTGCCATTTTAGAAAATGCAAAAT
CCAAGCCTTGCGACAGAAAATTTACACCATCCATGGCCTATGGCCAAGTAATTATTCGAACTCTCCCGTGGAGTACTGC
CCGGGATCGCGATTTCAAAATCCG
>Rmoschata08_14_Rmoschata17_36
ATGTTAATGCAGTGGCCAGGATCATTCTGTGATACACAGAAAAGTTGCTGCTATCCAACCACAGGGAAGCCTGCAGCAG
ATTTTGGGATTCATGGGCTCTGGCCAAATTACAAGGATGGTTCATACCCCTCAAACTGTGATCCAAACAACCCCTTTGA
TCAATCTCAG
>Rmoschata08_15
TCCAAGACTGTTTATGGTGACAGCTGCAAAGCTCCCTCTCCAGTTTTTACAATACATGGGCTCTGGCCCTCCAACCACA
CTAGCCCTCAGCTGTCGTGCACTGGTGTAGCATATAATCCCACAACG
>Rmoschata08_30
AATCCCTATGACTACCTCCAATTCGTCCAACAGTGGCCTGCTACATTATGTTCCAGCAGGTCAGATTGTATCCGAAATC
CCCCGCAGAATTTCTTTACAATCCACGGC
>Rmoschata08_34
GGGATCATGCAACTCGCCCTGCTCCTTCTCTCTGCGTGGGGGGCAGCTGATGCAGCCAATCCCTACGACTACCCCCAAT
TCGTCCAACAGTGGCCTGCTACATTCTGTTCCGGCAGGTCAGATTGTATCCCAAATCCCCCGCAGAATTTCTTTACAAT
CCACGGCCTTTTTTTTTTTTGGCAAAGA
>Rmoschata08_37

TCCATGAGCCTATGCTTGTTTCTGCTAGTATTGGCATTGATGGCACCCTCCGTCAACTCAAAATATCAGTACACCCAAC
TTGTCCAAATGTGGCCACTGGGTCACTGTCATTTCCCACCACATTGCACTGCACCGATACCTCGGAACTACTTTAGTGT
GCATGGCTTATGGCCAAGTAATCACAGTGTACCCAAGATTACATGCAAGAAT
GTATATGTTCCCTTCTCCGGAACACTG
>Rmoschata08_39
TATTTTCAATTGGTGCAACAATGGCCGCCCACAACCTGCTATCCACCCAAAATTTGCCACAGAATTCCGCCCAGCTCGC
CACCACACCTCTTCACCATCCATGGGTTTTGGCCTAGTAATTATTCAGGCATTCACGTGGTTAATTGCACAGCGGGACT
ATTTCAAAGGGTATATCGTATT
>Rmoschata17_2
CCGGCCGTCTGTGACACGTCTGGCTGCAACAGATCGGGGTTATCACGCTTCACGATCCATGGCCTTTGGCCAAATAATA
AAACGTATGTGAAAAATCAACCGACTTGTCTTACCAATCAGGCCAACTCATTTAAGGCTAAG
>Rmoschata17_15
CAGTGGCCTGCTACATTCTGTTCCGGCAGGTCAGATTGTATCCCAAATCCCCCGCAGAATTACTTTACTATTCACGGGC
TTTGGCCATCCAATTACTCGACTCCTGGACAGGATTGCCATGGCACACCTTTTAGTGGAACTGAGGTATTC
>Rmoschata17_18
GTCCTTCAATGGCCAACTACATTATGCATTAACCAAAACTGCATCCCAAATCTCCCGCTGGACTTCACTCTACATGGGT
TATGGGCCAGCAATATCTCTAGCCAAAACAACCCTTGTGTT
>Rmoschata17_30
ATGCCGCaAAACAAATATGACTATCTACAACTAGTGCAACAATGGCCTAAAACGTTCTGCCATAATAACCAAGCTTGCA
TTCAAGGTGCAGCCCTCCCGGAGCTGTTCTCGATACATGGTATGTGGCCATCTAATTTCTCCGGCCAGAACGACGCTTG
TGTTGGAACTCGGTTCAGCATGAGAGAGGTACATATATAT
>Rlaevigata_3
AATTTTGACATAGTTTTTTTTTTCTTTTCTCTTTTGAAAATAATTTATGTAGGTTCGTATCACTATTTTAAATTTGTGC
AACAATGGCCGCCAACCACCTGCTACAACAAAGGTTGCCCAGGCCAACCAACATATTTCACCATCCACGGCCTTTGGCC
TAGCAATCGAACTTGGATACACAACCAAGGTTGCTCTGGGACGTCATTTAATGGGAATTTG
>Rlaevigata_8
ATGGCAGTAGTGGCATCGTCCTTCTCTTTCATTATTCTTACCTCTGCTTTCTTCCGGTGTTTCTGTTTGAGCATCGGTG
CCTACGATTATTTTCAATTTGTGCAGCAATGGCCACCGAACTTCTGCTACGGGACTAGATATCCTGGTTGCAACATTAA
CAGGCGATCATTCACCATTCATGGCCTGTGGCCAAATAATATTTCTCAAATTAATTACAATCATATTTGTGCAACGGGA
AGC
>Rlaevigata_9
AAGTACGACTACTTCCAGTTTGTCCAGCAATGGCCGGGTACATTCTGCTATCAAAATTCCGATTGTGTCCGGAATCCCC
CGCGGTATACTTTTACCATCCACGGCCTCTGGCCAAGTAACTACAACTCTACCAGTCTAACACTAGTATGTATGTGCGC
ATAT
>Rlaevigata_11
ACCCTGAAAGATAGTTTCTCGTTATTTAACATGCATTTTGATGAATCGATGCAGTGGCCTGGAGCATATTGCGACACAA
AGCATAGTTGTTGTTATCCAAAGTCAGGGAAGCCTGCAGCAGATTTCGGCATTCACGGTCTCTGGCCAAACTACAAGGA
TGGCTCTTACCCTTCAAACTGTGATCCTGACAGTGTCTTCGACAAATCTGAG
>Rlaevigata_31
ATCATCATCATCATGGGGATTGTCCTCCTGATTCTTCTCTGTGCTTTTCGGGTGGCTAGTGCAGCCAACACATATGATT
ATCTCCAACTAGTCCTGCAGTGGCCAAACACGTACTGCATCTACCAAAACTGCATATCAAACCTGCCACTCGACTTCAC
CATACATGGGTTGTGGCCTAGCAATTTCTCCGGCCAAAACAACCCATGTGTGGGCACGCCATTTAATAGAGCTCAG
>Rpersica_16
AGATCCGGATGGATCCAACCACCAACCACTGCTCGCGCCTCCGAGCTCGAATCCGCCCCAAGAAGACCACGCAACAACA
CCATCCATGCCCACTGGCCAACAAACCACCGCCACCACCCTACCTCTCCTACAAACGAATTGGACAACGAACCCATCAC
CACAAAATCTCCACTT
>Rpersica_18
TCATCATCATGCGAATTGTCCTCCAGATTTATTCTCTGTGCTTTTGAGGTTGCTAATGCAGCTAACACGTATGATTACC
TTCTACTAGTCCTTCAATGGCCAACTGCATTATGCATTAACCAAAACTGCATCCCAAATTTCCCTTTGGACTTCACTCT
ACATGGGTTATGGGCAATCAATATCTCTGGCCAAAACAACATTGTGTTG
>Rxanthina_10
ATGTTAATGCAGTGGCCAGGATCATTCTGTGATACACAGAAAAGTTGCTGCTATCCAACCACAGGGAAGCCTGCAGCAG
ATTTTGGGATTCATGGGCTCTGGCCAAATTACAAGGATGGTTCATACCCCTCAAACTGTGATCCAAACAACCCCTTTGA
TCAATCTCAG
>Rxanthina_15
ATGGGGATTGTCCTCCTGATTCTTCTCTGTGCTTTTGGGGTTGCTAGTGCAGCCAACACATACGATTATCTTCAACTAG
TCCTGCAATGGCCAGACACGTACTGCATCAACCAAAAGTGCATATCAAATCTCCCACTAGACTTCACCATACATGGGTT

ATGGCCTAGCAATTTCTCCGGCCAAAACAACCCATGTGTGGGTACACCATTTAATAGAGCTCAGGTACTCTCTCTCTCT
CTCTCTCTCTCTCTCTCT
>Rxanthina_17
AAATGGAGTCCATTTTATGTGTTTTTTAACATTCTTTTCCCCTCGCAATATCTTTCTACAGCGAGCTACGATTATTTTC
AATTTGTGCAGCAATGGCCACCAAACTTCTGCTATGGTAGATATCCTGGTTGCAATAACACGCTATCATTCACCATTCA
TGGCTTGTGGCCAAATAATTTTTCTCAAGTTTATTACAATCATAATTGTCAAGGGAACGGATTTGACTATCAGGTACAT
ATGTTTTTTAAAATCTATATA
>Rxanthina_22
ATGGCAATACTGAAATTGCCGAACGCTTTCATTGTTCTTGCTTTCGCTTTTTATTTATATTTCAGTGTGAGCACCGGAT
ACTATGAATATTTCAAACTGGTGCAACAATGGCCACCAGCCACCTGCCATAACGGAGGATGCCGTGGAATACCGCCAGG
CTCAGCACCACACCTCTTTACCATCCATGGGTTTTGGCCATCTAATTATTCAAACAATATCGTGGCTAATTGCACTAAG
CCACAATTTCAGCAGGTATATAGT
>Rxanthina_30
GCAGAGCCATACGAATACCTACAGTTTGTCTTGCAATACGCTAGAGGTTACTGTGTTAACGTCAACAAATGCATTCCAC
CGGCGAGCTTGCCGGCCAAATTCACAGTTCATGGAATTTGGCCCCCGAATATTTCCGAGCCGATTATCAGCTGTGACAT
GGCACCGAAACTACACTCGTTCAACAGAAACCTGGTATGTATGTAT
>Rxanthina_32
ATGGCATTCCTAGCAGTTAGGACACTAATTGTTCTTACGCTTATTTCTACATTTGCTAAAGCCGCAAACAAATATGACT
ATCTACAACTAGTGCAACAATGGCCTAAAACGTTCTGCCATAATAACCGAGCTTGCATTCAAGGTGCAGTCCTCCCGGA
GCTGTTCTCGATACATGGTATGTGGCCATCTAATTTCTCCGGCCAGAACGACGCTTGT
>Rxanthina_35
TTTTTTTTCAACACAATAATGTCTCTAGGTTCATATGATCATTTTAAATTTGTACAACAATGGCCACCGGCCGTCTGTG
ACACGTCTGGCTGCAACAGAACGGGGTTATCACGCTTCACGATCCATGGCCTTTGGCCAAATAATAAAACGTTTGTGAA
AAATCAACCGATTTGTCTTACCAATCAGACCAACTCATTTAACGCTGCG
>Rmajalis_15
ATCATCATCATGGGGATTGTCCTCCTGATTCTTCTCTGTGCTTTTGGGGTGGCTAGCGCAGCCAATACATATGATTATC
TCCAACTAGTCCTACAATGGCCAAACACGTTCTGCATCAACCAAAATTGCATATCAAATCTGCCACTCGACTTCACCAT
ACATGGGTTGTGGCCTAGCAATTTCTCCGGCCAAAACAATCCATGT
>Rmajalis_6
TTTCTTTTCGCAATAATGTCTACAGGGTATTACGAATATTTCAAACTAGTGCAACAATGGCCACCAACCACTTGCCAAA
ATGCAAACTGCCGCAGAGTACCGCCACCTCGCCTCTTTACCCTCCATGGGTTTTGGCCATCTAATTATTCAAACAATGT
CGTGGCTAATTGCACTAACGCAATATTTCAACGGGTATATAGTGTTATGTTCTTGCTTTCGCTTTTTATTTAT
>Rmajalis_8
GCAGAGCCATACGAATACCTACAGTTTGTCTTGCAATACCCTAGAGGTTACTGTGTTAACGACAAGAAATGCATTCCAC
CGGCGAGCTTGCCAGCCAAATTCACGGTTCATGGAATTTGGCCCACGAATATT
>Rmajalis_19
TACTTGTTGTTTGTTCAACAGTGGTCCAAGACCGTTTGTGGTGACAGCTGCAAAGCTCCCTCTCCAGTTTTTACAATAC
ATGGGCTCTGGCCCTCCAACCACACTAGCCCTCAGTTGTCGTGCACTGGTGTAGCATATAATCCCACAATG
>Rminutifolia_6
ATGTTAATGCAGTGGCCAGGATCATTCTGTGATACACAGAAAAGTTGCTGCTATCCAACCACAGGGAAGCCTGCAGCAG
ATTTTGGGATTCATGGGCTCTGGCCAAATTACAAGGATGGTTCATACCCCTCAAACTGTGATCCAAACAACCCCTTTGA
TCAATCTCAG
>Rminutifolia_7
CATTATTTATGCCTCAATGCCTTATTCAACAATATTAATATTTATTTATTTCTTCTCCAATTAAAATGCACAGGATCCT
ATGAATATTTTAAATTTGTGGTACAATGGCCACCAACCCACTGTCAAATTAAAGGATGCAAATCCCAAGCCTTGCAATT
GAAAATTTACACCATCCATGGCCTATGGCCAAGTAATCATTCTACCTCTCACGTGGAGTACTGCCGGGTAGCGCCATTT
CAAAATCCA
>Rminutifolia_10
CAATGGCCAACTACATTATGCATTAACCAAAACTGTATACCAAATCTCTCGCTAGACTTCACTATACATGGGTTATGGC
CCAACAATATCTCTGGCCAAAACAAACCTTGTGCTGGTACACCATTCGATAGAGCTATATATATATATATATATATATA
TATATAT
>Rminutifolia_13
TACGATTATTTTCAATTTGTGCAGCAATGGCCACCAAACTTCTGCTATGGTAGATATCCTGGTTGCAATAACACGCTAT
CATTCACCATTCATGGGTTGTGGCCAAATAATTTTTCTCAAGTTTATTACAATCATAATTGTCAAGGGAACGGATTTGA
CTATCAG
>Rminutifolia_14
CAGTTTGTCTTGCAATACCCTAGAGGTTACTGTGTTAACGACAAGAAATGCATTCCACCGGCGAGCTTGCCAGCCAAAT
TCACGGTTCATGGAATTTGGCCCACGAATATT

>Rminutifolia_24
GTTCTCAACGTTTCAAATGCTGAACCCTACGAGTACATGCAATTTGTACTCCAGTACCCACCTGGAGTTTGCTATGACA
AATCAAAAGGTGATTGCATCTCACCTCTCCCAACCAAGTTTCATGTACATGGAATATGGCCTTCTAACTTCTCCAATAT
ACTTGTCCGTTGTGGGCACGCACTGACGAATAACCCCTTTCATAAGGCGCAG
>Rminutifolia_26
ATGGGACTATGCTTGTTTCTGCTAGTATTGGCATTGATGGCACCCTCCGTCAACTCAAAATATCAGTACACCCAGCTTG
TCCAAATGTGGCCGCCGGGTCATTGTCATTTCCCACCACATTGCACTGCACCGATACCTCGGAACTACTTTAGTGTGCA
TGGCTTATGGCCAAGTAATCACAGTGTGCCCAAGATTACATACAAGAATGTATATGTTCCCTTCTCCGGAACACTG
>Rarvensis_6
TCCTATGAATATTTTAAATTTGTGGTACAATGGCCACAAGCGCACTGCCGCATTAAAAGATGCAAACCCCAAGCCTTGC
AACAGAAAATTTACACCATCCATGGCCTATGGCCAAGTAATTATTCGAAATCTCCTGTGGAGTACTGCATGGCATCGCC
ATTTCAAAATCCGGTAGGT
>Rarvensis_9
AGCTGCTATAGTAGAAAACCAATGTGCCATCATCACATCATATACTTGAATCCAAGCTTGCTTGTTATAATCATTGATT
CAATCATGGCTGCTTTGAACCGTATTACTCCGCTTGCTATATCTCTGCTACTTGTAGCTGCTTTTTGTGTTCCCCCGGC
ATCAGCCAAAACAAGCTATGAGTATCTGATGCTCGTTTTACAATGGCCATTTGCTGTCTGTGCAAGCAAAGTTTGCACC
TACAAATATCCTCCTGATAACGAATTCTTACTTCATGGCCTGTGGCCAGCGAATTTTTCAGGTGCTCAGTTACAGTGCA
GCGGTACCCCTTTCGATCGCAGCGAGGTATATATATTA
>Rarvensis_14
CTGTTCAAGGGTTCCCATGCAGAGCCATACGAATACCTACAGTTTGTCTTGCAATACCCTAGAGGTTACTGTGTTAACG
ACAAGAAATGCATTCCACCGGCGAGCTTGCCAGTCAAATTCACGGTTCATGGAATTTGGCCCACGAATATTTCC
>Rarvensis_15
TTAATTTATTTGCATTTAACGTTCTTTCTTCTCTCAATAATATCTACAGCGAGCTACCAATATTTTCAGTTTGTGCAGC
AATGGCCACCAAACTTCTGCTATGGTAGATATCGTCCTTGCAATAGGCAATCATTCACCATTCATGGCCTGTGGCCAAA
TAATTTTTCACAAGTTAATTACAATCATATTTGTTCGGGGGCCGCATTTAACAATCAGGTACTCGATCTA
>Rarvensis_18
TCAACTCAAAATATCAGTACACCCAACTTGTCCAAATGTGGCCACTGGGTCACTGTCATTTCCCACCACATTGCACTGC
ACCGATACCTCGGAACTACTTTAGTGTGCATGGCTTATGGTCAAGTAATCACAGTGTGCCCAAGATTACATGCAAGAAT
GTATATGTTCCATTCTCCGGAACACTGGTTTAAACTT
>Rarvensis_22
CAACAAAGCAGGGTTGTTGCTACCCAAAAGAGTATTAGAAAACATCCCAGTTTCACAATTGGTGGCATATGGCCTTATA
CTTTTTCTGGGGATAGACCAACCTACTGCAAATCTAAGACCCCTTTT
>Rarvensis_23
GATTACCTTCTACTAGTCCTTCAATGGCCAACTACATTCTGCATTAACCAAAACTGTATCCCAAATCTCTCGCTAGACT
TCACTATACATGGGTTATGGCCCAGCAATATCTCTGGCCAAAACAAACCTTGTGTTGGTACACCATTCGATAGAGCTAT
ATATATATATATA
>Rarvensis_29
CTACTAGTCCTTCAATGGCCAACTACATTATGCATTAACCAAAACTGCATCCCAAATCTCCCACTGGACTTCACTCTAC
ATGGGATATGGGCCAGCAATATCTCTAGCCAAAACAACCCTTGTGTT
>Rarvensis_32
TTTCAAACCCTAAAAGATAGTTTCTCGTTATTTAACATGCATTTTGATGAATCGATGCAGTGGCCTGGAGCATATTGCG
ACACAAAGCATAGTTGTTGCTATCCAAAGTCAGGGAAGCCTGCAGCAGATTTCGGCATTCACGGTCTCTGGCCAAACTA
CAAGGATGGCTCTTACCCTTCAAACTGTGATCCAGACAGTGTCTTCGACAAATCTGAGGTACACGACACCTATACTTTA
ATCAACATATATATATAACCTTAGTTTTTGGA
>Rarvensis_34
TTTCATATGGCACAAAATAATATGGCTTACGTCCTCATGACCTGCACTACTGTTGCCTTACTCTTTACTCTGGCCTCCT
CGTATACGGCCTACGAATACTTCTTGTTTGTTCAACAGTGGTCCAAGACCGTTTGTGGTAACAGCTGCAAAGCTCCCTC
TCCAGTTTTTACGATACATGGGTTGTGGCCCTCCAACCACACTGGCCCTCAGCTGAAGTGCACTGGTGCAGCATATAAT
CCCACAGAG
>Rarvensis_35
ATGGGGATCATGCAACTCGCCCTGCTCCTTCTCTCTGCGTGGGGGGCAGCTGATGCAGCCAATCCCTACGACTACCTCC
AATTCGTCCAACAGTGGCCTGCTACATTCTGTTCCGGCAGGTCAGATTGTATCCCAAATCCCCCGCAGAATTTCTTTAC
AATCCACGGCCTTTTTTTTTTTGGCAAAGAGTATTTGAGAGGGGGGAGGCAG

Supplementary File 4

>Rmultiflora_sc0006888_F_box_minus4

ATGGTGGTACAAATCTTATCCAGAATGCCTCCGAAATCTCTAATGCGATTCAAGTGCATTCATAAGTCATGGAACTCTA
TGATCAATAGTCGCCATGTCGTAGCTAAGCATCTCCAGTTTCACAGCAACCTATCCTCCTCCACTAGCATCCTTCTAAG
GCGTCCTGTCATCTGGAGAACCGAAACTAAGAATGAGGAAATCGTTTTTTCTTTGCTTACTCTTTGCAACGAGAATAAT
GGTGATGAGGATAACCTAGATTATGACATCGAGGACATCCACTTTCCACCTTCAATTGGTCTAAAAACTAGGGCACAAT
TTATTGAGAATCCTGGTCCAACTTATGAATGTGCAGATATTGTGGGTCATTGTGGTGGGATAATCTGTCTCTCTCTTTA
TGCTGCAGGCGACCTTGTCTTATACAATCCCGCAATTAGGGAATTCAAGGTTATACCCGAGCCATGCCTCCCACGTCCC
CGTCAGTTTTATTTCCGTTGTGATGCATTTGGTTATGATCCCATTTCTGAAGATTATATACTTGTTAACGTTGCAAGTT
ATGGTGAAAATAGATACGATGATGATCGTCTCGTTATTGAACCTCTGAGAGCAGAGATGTACACACTGGGTACTGATTC
TTGGAGAGAGATCAAGATTCACAATTTGGAAACCGAAACTACCATGTTTCGGCCTAATCATTTCCAGGTGTATTTCAAG
GGAAACTGCTACGGGTTGGCAGAAGAAATCAAGAAGGAATTCATCTCATCGTTTGACAGTCTTGAGGAGTATTACATTA
GGGAAGTAATCGTTTGGTTTAACACGAGCGATCGGGTTTTCCATAGTGCATTGACTCCTGATTGTTTGTATCGATATCC
AGCGCATGACTTCACTCTTACAGTGTGGAACAATTGCGTTGCTCTTTTTGGCTATAATCGTTGTGGAAGTAAACCATTT
GAAATTTGGGTGATGGGTGACTCTGATGGTTTCACTTGTTCATGGATAAAGCACATATCGGTTAACATTACGGAATCTC
CTCAACCATTGGTACTGTGGGAGAGCAACCAGAGTCTTTTGGTGTCCCCGCGTATACGAGTAGCCTTGTACAGCTTTGC
AACCAAAACGTTCAGGTATTTACCACTATGTGCTGCTGAACATTTCGATGCTATACCTCTTGTGAATAGTATAGTTCCA
CTCAATAGGGACCCAGTTTGTGTTGATATTTCT
>Rmultiflora_sc0006888_F_box_minus3
ATGGCAAACTATAGCAAACTGTATGCGTCTGAGGACTTTGTGGAGCAAATTCTATTAGAACTGCCTCCCAAACCTTTGA
TGCGATTTAAGTGTGTATGTAATCTGTGGTGCAATTTAATCAAAAGCCCTAGTTTCGTAGATAAACACATTTCCAGTTC
TATGCGAGGATCCTCTATGCCCATCCTTATCAAGCGCCCAGTTCCCACGGACAAGGACAATAACATTAAGGATGAGAAG
AAAGTTGGGAATGATGACGACGATGTCGAAACTCTCTTGACGTCACTTAATATCTGCAATGAGGATGATGATGATTACC
CTCTATCAACTGTAGTTGAGGATCTTAACGTTCCGCTTCCGGCTCCTCTGAAGCTAAAACATTCCTCGGATCTCACAAT
TGCAGGTCATTGTGATGGAATCATTTGTTTAAAACTTTTCACTGGTAACGTTATTTTATGCAACCCAGCTATAAAGGAA
TTCAAGCTTCTTCCCAAGTCTTTTCTTCTCCTTTGCAATGATAACTTTGATGATCCCTGGTCGCTTTCTTATGAATTAA
GATATTACAATGAATATTTGGGATTTGGCTATGATCCCAAAGGTAAAGATTACAAGGTTGTTAGATTCGTAATCTATAA
TGAGTCATGTTGTTGGTTCAAAGCAGAAGTATACACTACGGATTCTAATTCTTGGAGAGAGATCAAGTCCGAATATGAT
GGTAAAATCTTTATTGTTAATTGGTCTTCTGATATGCCTTTATACTTCAACGGAATTTGTTATTGGCACGTAAGTTGTG
CACACCTGGAATCTGTTCTTTCATTTGATATGGATAAGGAGCTATTTCATGAGATATTGATTCCAGATTTGCCAAACGG
ATGTAGAGTAGTGATGCTTACTATGTGGAAAGAGCTCATTGCTTTTTTCACCTATCAAGAAGAAATTGGAGTTCCTCAA
TCTTACGATATGTGGGTGATGATGGATGATCTTAGTGATGGTAAGGGTTCATGGACAAAACATTTAACTATAGGACCTG
TGGAAGGTGTTAAATTTCCATTGATATTTTGGAAAAATGACCAACTTCTTATGGTTTCTAATAACGGAAGTATCGTCTT
ATATAACCTTGGCATACAGAGATTAAACTATCTTCCTATTCATTCCATGAGAGATCTTTACTATAGTCAAGCACTTGTT
TATGTAAATAGTATTGTTTCCATCAGCGGAGGCAATGTACTTGAAGATATATATATTTCTGCGTTTTATGGCAATGGAA
AATTTCATTCCATCAACGGACGAGACATAGTAGATATTTCCGCTTTTTATGGCAATAGCGAGGAACAAGTCTCAGGTTC
ACCCCTTAGG
>Rmultuflora_sc0006888_F_box_minus2
ATGGCGGTAGAAATCTTATCCAGACTGCCTCCGAAATCTCTAATGCGATTCAAGTGCATTCGTAAGTCATGGAATTCTC
TGATCAATAATGCCCATTTTGTGGCCAAGCATCTCCACGTTCACAACAACGTATCCTCCTCTACTAACATCTTTCTAAA
ACGTCGTGTCATCAGGGCAACTGAATATAGGGATGAGGAAATCGTATGTACGTTACTTACTCTTCGCAATGAGAACAAT
TGTGCTGAGGACAACATATATTATGATATCGAGGACATCCAATTTCCTCCTTCAATTGGTCTAAAAAGTAGGGGACAAT
TTATTGAGATCCCTGGTCACTGCTCTTATGATTGTGCATATATTGTTGGTCATTGTGATGGGACATTTTGTCTAACTCT
TTATACCGCAAGCGACCTTGTTTTATACAATCCAGCAATCAAAGAAGTCAAGCTTATACCAGAGTCATGCCTTCGAGAT
AAGTTTATCGGTGCTGTAGGATTTGGCTATGATCCGAAGTCTGAAGATTACATACTGGTTAGCGTTACACGTTATGGTG
AAGAAGCATACGATGATCGTGTTGTTATTAATCCTCTGAGAGCAGAAATGTACACACTGGGTACTGATTCTTGGAGGGA
GATCAAGATCCACAATTTGGAAACCGAAACTACTTTTTTTTGGCCTGCACATTTCCAGGTGTACTTTAAGGGAAATTGT
TACTGGTTGGCATATGAAAACCAGAAGGAATTCATCAGTTACTTGGACAGACTTGAGGAGCAATATATTAGGGACGTCA
TCGTTTCGTTTGACACGGGCAATCAGGTTTTCCATACTATATTGGTTCCGGATTGTTTGTATGAATATCCAACGCATGA
ATTTTACCTCACAGTATGGAACGAATCAGTTGGTCTTTTTGGCTTTTATCGTGGTGGAAATAAACCCTTTGAAATTTGG
GTGATGGATGATTCTGATGGTGTTAACTCTACGTGGATAAAACACCTATCCATTGACGTTGTGGAACCTGCTATACCAT
TGGCACTCTGGGAGAAAAATGAGATTCTTTTGGTCTCCACATGGACACAAGTATCCTTGTACAACTTTGTAACTGAAAA
GTACATGTATTTACCGCTTTATGGTGCGCTTTATTTCGAAGCATTTCCCTATGAACCTAGTATAGTTCCACTCAAGAGG
>Rmultiflora_sc0006888_F_box_minus1
ATGGTAGAGTTGTGCAAGATGTCAGAAGAGATAGTGGTGCAAATGTTATCAAGGACGCCTCCTAAATCTCTAGTGCGAT
TCAAGTGTATCCACAAGTCATGGTATTCTATGATCAATGATCCACAATTTGCAGCCAAACATCTTCACTTTTATAACAA
TCCATCTTCGTCCACTGCCTTCCTCGTCAAGCGTCCTGTCATCCTCAGAAGTGAAACGAGCAATGAGAACGTTGTACTC
TCATATCTTCGTCTAGAAAATTATAGTAATGGTGACGATGAAGATCTTCATTTCGTAGTTGAGGACCTCATTTTCCCAC
CTTTTAAGGGTCTAAAAACTAGGGGACAATTTATTGAGCTCCCTGGACGTGATGATTCTGTGTATATCATTAGTCACTG
TGATGGCATTATTTTTCTAAGTCTGTATGCTGGCGACCTTCTTTTGTACAATCCAGCAATCAAAGAATTCAAGATTATT

CCGGCGTCATGTTGTCAAGATTGTTTTTGGAGCTTGGTGGGATTCGGATATGATCCGAGATCCAAAGATTACATTATTC
TAGAAATTGCATGTTATGGTGAGACAAATTGTGACCATCCTCAGCGTCTCATTGTTGATCCTCCAAAAGCTGCAGTTTA
CACACTAGGCATTGATTCTTGGAGAGAGATCCAGACTGATTACTTACAAACTGAAGATACCTACTTTTGGCCTACGGCA
TTCGATTTGTACTCGAAGGGAATTTTGTATTGGTTTGGTTATGAGGAAAAGAAGGAGTTCTTGGATGACATGGAAAGAT
GTGAGGAGACAAACAAGCAAGTGATGATTTTGTATGATACGGTAGATGAGCTATTTCATATTGCAATGCTTCCGGATAG
TTTCAACGAGCCTGCTTGCGGCATACATGATATTCATCTTACATTGTTAGATAATTCAATTGCTCTTTATGGTTTTAGC
ATTTTTGAATCGATTCATTCCATTCAAATATGGGTGACAGATGACATTAAAGAGGAATCTTGGACAAATTACTTGTCCT
TAGAGCCGATAGATGCTGTTCGGAGGTCATTGGCCTTTTGGAAGATCAACAAGGTTCTTATGATTGCCAAAGATGGGCG
TGTAGTTCTCTACAATCTGGTTACAGGAAAGCTCAAGCATTTTCCTATTCATGGCCTGCACCTAGGAGATGATATTCAA
GGCATTGTTTGTATGGATAGTATAGTTTCAGTTATGAGAAAACAA
>Rmultiflora_sc0006888_F_box_plus1
ATGACAGAGTTTTCTAAATTTCCTGAAGAGATGGCCTTGCAAATCTTATCAAGGATGCCACCTAAATCTCTGATGCGAT
TCAAGTGTGTCCGTAAGTCATGGTATACGTTGATCAACAGTCCCAGCTTCGTATCCAAACACCTCTTTAATTCCTTGCA
CAACAAACAGTCCACATGCATCTTTTGCAAGCGTTACGTCTTCAGGGATATCGCAACTAAAGATGTGGAATCTGTAATC
TCATTGATTACTTTTTCCAATGATGATGTTGGCGATAATGACCATGAGCATATCTCTCATTCGGTTATCCAAGACATCG
ATCTTCCACTTTCTATGAGTGGAGTACCGAAGAACCATGTCAATGAGCCTGAGCTTCTCGGAGCTGTATATATTACAGG
GCATTGTGATGGAATCATTTGTTTAGTCCATGGTGAGATTGTGCTATGGAATCCAGCAATTAAGCAATTCAAGGTTCTT
CCCAAGCCACTCCTTACAAATGGGATCGTAAATTCTATAGGATTTGGCTATGATCCCAGATCTAAAGATTACCAAGTTT
TTAGTTTTCCGACTCATGATGAGGACCGAACTAGCGAGCGTGATTTTAATTATCCTCCACAGGTTGAAGTATACAGCCT
CAGTACCGATTCGTGGACAGAGATCAACGCCGATCATTTAGAAACTGAAACTACCAACTTGTATCCTGAATATTTTCAA
ATGTGCTTTAAGGGAATTTGGTATTGGACCGGAAGTGAGCAACAAAAGGAATTCATGGTTGTTTATGATAGTATGGATG
AGGAATGGGTGAGGCAGCTGATCATTGTGTTTGATATGAATGATCAAGTATTTGAAGACATACTATTTCCATATAGTTT
ATACCACCCGATGATTCCTTATTTAGAAATGCGCGTTATTGTGTGGAATGAATCCGTCGCTCTTTTTGGTCAGTACCGT
TTTGGTTATGCTGATGATGCTTTTGGATTATGGGTAATGGATGATATTGTCAAGGGTTCTTGGACAAAACAATTAACCC
TAGAGGTCATAGTTGGTACTCGAATGATTTTGGAAATGTGGAAGAGCGACGAGATTCTTATGGTGGCTAATGACAACCG
TATATTCTCCTACAATTTCAGAACTGAAAAGATTAAATTTCTTCCCATTGAAAGTACGCATCCAACTTTTTCCGCAGCT
ATTGTATGCATAAACAGTATAGTTCCGGTTATTCATGGGAGGCAGCAAGCA
>Rmultiflora_sc0001861_F_box_minus3
ATGACGGAGTTCTGCAAATTGCCGGAAGAGATGGCCATGCAAATCCTATCAAGACTGCCTCCCCAATCTCTTATGCGAT
TCAAGGGCGTCCACAAGTCCTGGTATACTATGATCAACAATCCCAAATTTATAGTCAAGCACCTTTCCAATTCCATGCA
TAACCATTCCTCCATTTGTGTCCTTTTCAAGCTATCGGTCCTCAAGAACAACAACACAAGAGACAATGACGAGAAGGAA
ACTGTATTCTCGTTGCTTACATTTTGCAATGATGATGATGATCAGAAGGAGCATATCATTCATTATGTTGGCGATGATA
TTACGAAGAATCACTTTGTTGGGCTCAAGGTCAAGGATCTAGAATCTGTATGGATTATAAGCCATTGTGATGGAATTAT
ATGCCTAGCTGATTCTAGTAACGTCATTTTATGGAATCCAGCGCTTAGGGAATTCAAGCTTCTTAACCTCGATCCTTAT
CCGGATCAGGTCATGACTTCTTTTGGCTTCGGTTATGATCCAAAGTTAAAAGATTACAAGGTTGTCAACATTGGAAAGC
CTGGTGAGCAAGAATATGGTGACGGTCATCTGCTTAATAATCCTCCCACCGTCCAAGTATACACCTTAGGTACTAACTA
TTGGAGAGAGATCAAGACCGGCTCCTTGGAAACAGAAACTACGAATATGTGGCCCGACGAATTCGATATGTACTTTAAG
GGATTTGTTATTGGAGGGGAAGGGAGCAAATCAAGGAATTCGACAATTTTTGTGACAGAAATGAGGAGGAATATGTTA
GGCAATTAATCATTTCGTTTGATACGGTTGATGAGGTATTTCATCATATATTGTTTCCGGATAGTTTATATCAGTCATT
TGCGTGTTGGTATTCTAATATGAACGTTATAGTTTGGAATGAATCGGTTGCTCTTTTCGGCATGGATTATGGTATATTC
GAAGATTATTCACGGGTATTATGGGTGATGGAAGACTTTGGTGGAGTAGCTAAAGGTTCCTGGATAAAGCAATTCACCT
TTGGGACAGCAGCGGGAATTGAGAAACCATTGCAATTTTGGAATAGGGATGAGATTCTTTTGGTTTCCGAAGGGCGTTT
AGTCTCCTGCAACATCGATACTGAAAAGCTTAAGCATCTACCCATTCCTAACATGGATTTTGATCATTTGGAAGCTGCT
GTTTATGTTAATAGCATAGTTGGAATT
>Rmultiflora_sc0001861_F_box_minus2
ATGACAGAGTTTTCCAAATTTCCTGAAGAGATGGCCTTGCAAATCTTATCAAGGATGCCACCTAAATCTCTGATGCGAT
TCAAATGTGTCCGTAAGTCATGGTATACATTGATCAACAGTCCCAGATTCGTATCCAAACACCTCTTTAATTCCTTGCA
CAACAAACAGTCCACATGCATCTTTTGCAAGCGTTACGTCTTCAGGGATATCACCACTAAAGATGTGGAATCTGTAATC
TCATTGATTACTTTTTCCTATGATGATGTTTGTGATAATGACCATGAACATATCTCTCATTCAGTTATCCAAGACGTTG
ATCTTCCACTTTCTATGAGTGGAGTACCGAAGAACCATGTCAATGAGCCTGAGCTTCTCGGAGCTGTATATATTACAGG
GCATTGTGATGGAATCATTTGTTTAGTCCATGGTGAGATTGTGCTATGGAATCCAGCAATTAAGCAATTCAAGGTTCTT
CCCAAGCCACTCCTTACAAATGGGATCGTAAATTCTATAGGATTTGGCTATGATCCTAGATCTAAAGATTACAAAGTTT
TTAGTTTTCCAACTCATGATGAGGACCGAAGTAGCGAGCGTGATTTTAATTATCCTCCACAGGTTGAAGTATACAGCCT
CAGTACCGACTCGTGGACAGAGATCTACGCCGGTAATTTAGAAACTGAAACGACCAACTTGTATCCTGAATTTTTCCAA
ATGTACTTCAAGGGAATTTGGTATTGGACCGGAAGTGAGCAACAAAAGGAATTCATGGTTGTTTATGACAGTATGGATG
AGGAATGGGTGAGACAGCTGATCATTGTGTTTGATATGAATGATGAAGTATTTGAAGACATACTATTTCCATATAGTTT
ATACCACCCGATGATTCCTTATTTAGAAATGCGGGTTATTGTGTGGAATGAATCCGTCGCTCTTTTTGGTCAGTACCGT
TTTGGTTATGCTGATGATGCTTTTGGATTATGGGTAATGGATGATGTTGTCAAAGGTTCTTGGACAAAACAATTAACCC

TAGAGGTCATAGTTGGCACTCGGATGATTTTGGAAATTTGGAATAGCGACGAGATACTTATGGTGGCTAATGACAACCG
TATATTTTCCTACAATATCAGAACCGAAGAGATTACATATCTTCCCATTGAAAGTACGCATCCAACTTTTTCCGCAGCT
ATTTTATGCATAAACAGTATAGTTCCGGTTATTCATGGGAGGCAACGAGAA
>Rmultiflora_sc0001861_F_box_minus1
ATGGTGGTACAAATCCTCTCCAAACTGCCTCCAAAATCTCTAATGCGATTCAAGTGCATTCATAAGTCATGGAATTCTC
TGATCAATAGTCCCCATTTCGTCGCCAAACATTTCCAGTTTCAGAACAACCAATCATCTTCCACTACCATCCTTCTAAA
GCGTCCTGTAAACCGCAGCACTGAAACTGTGAATCAGGAAATGGTTATTTCGTTACTTACTCTTCGCAATGAGGATGAT
GGAGATGAGGATAACCTTTATTATGATATAGAGGACATCAAATTTTCGCCTTCAATTGGTCTAACAAGTAGGGGACAAT
TTATTGATACCCATGGTCAAACTTATGATTGTGCAAAAATTGTGGGTAATTGTGATGGGATATTTTGTCTCTCTCTTTA
TACTGCAAGAGACCTTGTTTTATACAACCCATCAATCAAGGAATTCAAGCTTATACCCGAATCATTCTTTCGAGATAAG
TATTCAGGTCCCACGGGATTTGGGTATGATTCCAAGTCTGAAGATTATATACTTGTTAGCGTTGCACGTTTTGGTTTGC
AAAAATACGGTGATCGTCTCGTTATTGAACCTCTGAGAGCAGAAATGTTCACACTGGGTGGTGATTCATGGAGAGAGAT
CAAGATTAACAATTTGGAAACCGAAACTACTATGTTTTGGCCTTACCATTTCCAGATGTATCACGCGGGAAAGTGTTAC
GGGTTGGCACATGAAATCAGGAAGGAATTCATCTCTTCGTATGACATTCTTGAGGAGCATTACATTAGGGAAGTGATTG
TTTGGTTTGACACGAGCGATCGAGTTTTCCATAATACATTGGTTCCTGATTGTTTGTATGAATTTCCAATGCATGACTT
CAGCCTTACAGCGTGGAACAATTCTGTTGCTCTTTTCGGCTATAATCGAGGTGGAAGTCTACCCTTTGAAATTTGGCTG
ATGGCTGACTTTGATGGTGTTCATTCATCGTGGATAAAGCATTTATCCATTGAAGTTGGGGGATCTCGTAGTTTAGGTC
GCAAAGCATTTACACCATTGGCACTTTGGGAGAGCAATGAGATTCTTTTGGTTTCCACAGGTACAGGAATAGCGGCATA
CAACTTGGTAACCGAAAGGTACAAGTATCTACCACTGTACGGTACATGGTATATTGAAGCTTTTCCTTATGTAACTAGC
ATAGTTCCTGTCAAACGAGGTAATAAGTTTGAGAACGAGGATATT
>Rmultiflora_sc0001861_F_box_plus1
ATGGTGGTAGAAATCTTATCCAGGCTGCCTCCGAAATCTCTAATGCGATTCAAGTGCATTCATATGTCATGGAATTCTA
TGATCAATAGTGCCCATTTTGTGGCCAAGCATCTCCACTTTCACAACAACCTATCCTCCTCTACTAACATCCTTCTAAA
ACGTCGTGTCATCAGGGCAACTGAAACTAGGGATGAGGAAATCGTATGTACGTTACTTACTCTTCACAATGAGAATGAG
TGTGATGAGGACGACGTACATTATGATATCGAGGATATTCAATTTCCTCCTTCAATTGGTCTAAAAAGTAGGGGACAAT
TTATTGAGATCCCTGGTCACTGTTCTTATGATTGTGCATTTATTATTGGTCATTGTGATGGGATATTTTGTCTAACTCT
TTATACCGCAAGCGACCTTGTTTTATACAATCCAGCAATTAAAGAAGTCAAACTCATACCAGAGTCAGGCCTTCAAGAT
AAGTATATAGGTGCTGTAGGATTTGGCTATGATCCCGAGTCTGAAGATTACATACTGGTTAGCGTTGCACGTTATGGTG
AAGAAGCATATGATGATCGTGTCGTTATTAATCCTCTGAGAGCAGAAATGTATACACTGGGTACTGATTCTTGGAGGGA
GATCAAGATCCACAATTTGGAAACCGAAACTACTTTTTTTTGGCCTGCACATTTCCAGGTGTACTTCAAGGGAAATTGT
TACTGGTTGGCATATGAAAACCAGAAGGAATTCATCAGTTACTTGGACAGACTTGAGGAGCAATATATTAGGGACGTCA
TTGTCTCGTTTGACACGGGCAATCACGTTTTCCATAGTATATTGGTTCCGGATTGTTTGTATGAATTTCCTACGCATGA
ATTTTACCTTACAGTGTGGAACGAATCAGTTGCTCTTTTTGGCTTTTATCGTGGTGGAAATAAGCCCTTTGAAATTTGG
GTGATGGATGACTCTGATGGTGTTAACTCTTCGTGGAGTAAACACGTATCCATTGACTCTGTGGAACCTGCTATTCCTC
TGGCACTTTGGGAGAGAAATCTTTTGATCTCCACGTGGACACAAGTAACCTTGTTCAGCTTTCTAACTGAAAAGTACAT
GTATTTACCGCTTTATGGTGCGCTTTATTTCGAAGCTTTTCCTTATGAGCATAGTATAGTTACAGTCAAGAGG
>Rmultiflora_sc0001861_F_box_plus2
ATGGTAGGGTTATGCAAGATGTCAGAAGAGATAGTGGTGCAAATGCTATCAAGGACGCCTCCAAAATCTCTAATGCGAT
TCAAGTGTATCCACAAGTCATGGTATTCTATGATCAATGATCCACTATTTGCTGTCAAACATCTTCACTTTTACAACAA
TCCATCTTCCTCTACTGCCTTCCTCGTCAAGCGTCCTGTCATTCTCAGAAGTGAAACGAGCAAAGAGAACGTTGTACTT
TCATATCTTCGTCTAGAAAATTATAGTAATGGTGACGATGAAGACCTTCATTTCATAGTTGAGGACCTCATTTTCCCAC
CTTTTAAGGGTATAAGGACTAGGGGCCAATTTATTGAGCTCCCTGGACGTGATGATTCTGTGTATATCATTAGTCATTG
TGATGGCATTATTTTTCTTACTTTGTATGCCGGCGACCTTGTTTTGTATAATCCAGCAATCAAAGAATTCAAGATTATT
CCAGCGTCATGTTGTCAAGATTGTTTCTGGAGCTTGGTGGGATTCGGATATGATCCGAGATCCAAAGATTACATTATTC
TAGAAATTGCGTGTTATGGAGGGACAAATTATGACCATCCTCAGCGTCTCATCGTTGATCCTCCTAAAGCTGCAGTTTA
CACACTAGGCATTGATTCTTGGAGAGAGATCAAGACTGATTACTTACAAACCGAAGATACCTACTTTTGGCCTACAGCA
TTCGATTTATACTCTCAGGGAATTTTCTATTGGTTTGGTTATGAGGAAAAGAAGGAGTTCTTGGATGACATGGAAAGAT
GTGAGGAGACAAACAAGCAAGTGATGATTTTGTATGATACGGGAGATGAGCTATTTCATATTGCAATGCTTCCGGATAG
TTTCAATGAGCCTGCATGCGGCGTACATGATATTCATCTTACATTGTTAGATAAGTCAATGGCTCTTTATGGTTTTAGC
ATTTTTGAATCGATTCCTTCCATTCAAATATGGGTGACAGATGACATTCGAGAGGAATCTTGGACAAAATACTTGTCCT
TAGAGCCGGTAGATGCTGTTCGGAGGTCATTGGTCTTTTGGAAGATCAACAAGGTTCTTATGATTGCCAAAGATGGACG
TGTAGTTCTCTACAACATCGTTACAGGAAAGCTCAGGTATTTTCCTATTCATGGCCTGCACCTAGGAGATGATATTCAA
GGCATTTTTTGTATGGATAGTATAGTTTCAGTTATGGGAAAACAA
>Rchinensis_NC_037090_F_box_minus4_1507747_1509120_
ATGATTTCGGACAAAACGAGGTTTGATAACAAATCTCTGGTCTGTAAGAAAAGATTTGGTATGGTGTTGGGAAATTTTT
TTTCAAACATATGCAGCAATCTTCTATGGTTGTTGAAGGCTTGTACGTTTTCGATAAAAAGACGTGATAATGAGAACGA
GGGAAAGACTTTAACATTGACGAACTTACCGGAAGATGTCACCATCGACATCCTTATGAGACTGCCGGTAAAATCACTT
TGTTGCCTCGAGTGTGTCTCTAAGACCTCTTTGAACATAGTGGGCAGCCCTCAGTTTGCTGCACAGCACACTCGACGTT

TATTGAACTCGTCTTCTGCAGTTGAAGTGCCTCAACTTATGCTTCTTGCACGATACTCTAATCTGCAGCATTGTGCAAT
AAATTTGCAGTCATTGAAGTACAATGGCACTAGCTGCTCGACGAAGACCAATTTTGCTGATATCGTTTTCATGAACGAT
TGGTATAGGTACGAGGTAGAATTTGTCTTCAGCAATTTGTTTGGAATTAGTAGGAGACTGAGTGTTAATAGAGAGCTGT
GCTACTTGGTCAATCCTCTTAGGGGAGAAGTCCTAGAGCTCCCGAGAAGTGATGTCAAAGCTCCAAACTATAATGGATA
TGATCCATGCATTCATGACCGGTACGGAATGGGATTTGATCATGTAAGTAGTACCTACAAGATTGTTCATATTTCCGAA
TATGCCTATGAAATTTCCAGCGACATAGCCGCCCGAGTTTATGCGATGGGAACAAGATCATGGCGACCGATACAATCAG
TTCCTCTGTGTCATTTCAGCAACAAGAGTGTATATGCGTATGGTGACAGGCATTGGTTGGTTGAAGGTGATAAAAGCCC
CAATAATTTTATTGTTTCCTTCGATTTCAGAAAAGATGAATTCTATTGGACTCACCATCCCGACTTGCAGGCTTCTTCT
TCTCACAGATTTTGGTATGATTTTCATTTGCTTAATCTCAAAGGATCTTTAGCCATCATGGATTATTGTTCATATCCGG
AAGAGTATATTAAGATATGGGTGCTGAAAAATTATGACAAAAAAGAGTGGGCGCTAGATTACACAATAAACATGAAAAC
GTTTGTAGGACGGCCTAGGGGCACGTTATTGAAATGGGGTGAATGGGAAAATGGCATTTTTACCATTCAAAGTGGTGTC
TGCTACTTTGTGGATCTCAGACGTGGTTCCATGAAAGGAGTAAAACTGCCTCCCAGCATCATGAATTACAATCTTTGGA
CCGCAGAGCAATACGAACAAAGTATATTCAGTTATACAGGAAGTATGGTTTCCCTAAAGAATTATGGCAATTTGATTGA
AGCAGCAAGACCAGGCATGTGGTTTCTACAG
>Rchinensis_NC_037090_F_box_minus3_5375622_5376560
ATGTCTGAAGAGATTATGGTGTCAATCCTATCAATATTGCCTCCCAAATCTGTGATGCGATTCAAATGCGTGCGTAAGT
CATGGTATTCCTTGATCAACAATTCTAGCTTCATAGCAAAGCACCTTTCTAATACCATGTGCAATAAACACTCCACATC
CATCCTTTGCAAGAGTTGCGTCCTCAGGGATATCAACACCGACGAGAAGGAAGTTGTCTGCTCATTGATTTCTCTTTCC
AGTAATAGCAATAACGATGAGCATCACATTCATTCTGCAGTCGAGGACATTCACATTCCGCTTTCTTTTGGTGTAACCA
CAAGGGGCCAGTTCAAGGGGGATGAAGTTCTATTAGCTGTATCTATTATAGGGCATTGTGATGGAATTATTTGTCTAGT
AGCTTCTTTGAACATTGTTTTATGGAATCCAGCAATCAAGGAATTCAAGATTCTTCCCAACCAGTGCCTTCCAAATGGG
ACTCTAAATTCGATGGCATTTGGCTATGATCCCAAATCTAAAGATTACAAATTTTTAAACATTGTGGATCCTAGTGAAG
AAACACTAGGTGAGCATCGTATCGTTTATGATTTTCCCAGAATAGAAGTATACACCTTGAGTACCGACTCTTGGAGAGA
AATGAAGACCTATAGTTTAGAAACAGAAACTACTATGTTCCTATGTTTTACCAAATTGTACTTCAATGGATTTTGTTAT
TGGATCGGATGTGAGAAACAAAAGGATTTCATGGATTATTTTGATAGAGATGACGAGGAATGGGTTAGGCAGGTGATCC
ATTCATTTGATATGAGTACTGAAGTCTTTGATCATATATTGCTTCCGAATAGTTTATACTTATACGAGCCACTGGCGCT
GTATTTTAACATGCACGTCATTTTGTGGAATGAGTCTATTGCTCTTTTTGGCTTGTATCATTGGCGGTCC
>Rchinensis_NC_037090_F_box_minus2_5433148_5434392
ATGGCAGGGTGCAAATTGCCAAGGGTGATGGTGGTACAAATCTTATCAAGAATGCCTCCGAAGTCTCTTATGCGATTCA
AGTGCGTTCATAAGTCATGGAATTCTCTGATCAGTAGTCGCCATGTCGTAGCTAAGCATCTCCAGTTTCACAACCACCT
ATCCTCCTCCACTACCATCCTTCTAAGGCGTCCTGTAATCTGGAGAACCGAAACAAAGAATGAGGAAATCGTTTTTTCT
TTGCTTACTCTTCGCAATGAGAATAATGGTGATGAGGATAACCTGGATTATGACATCGAGGACATCCACTTTCCGCCTT
CAATTGGTCTAAAAACTAGGGCACAATTTATTGAGAACCCTGGTCCAACTTACGAATGTGCAGATATTGTGGGTCATTG
TGGTGGAATAATCTGTCTCTCTCTTTATGCTGCAGGCGACCTTGTCTTATACAATCCCGCAATTAAGGAATTCAAGGTT
ATACCCGAGCCATGCCTCCCACGTCCCCGTCAGTTTTATTTCCGTTGTGATGCATTTGGTTATGATCCCAAGTCTGAAG
ATTATATACTTGTTAACGTTGCAAGTTATGGTGAAAATAGATACGATGATGATCGTCTCGTTATTGAACCTCTGAGAGC
AGAGATGTACACACTGGGTACTAATTCTTGGAGAGAGATCAATATTCACAATTTGGAAACCGAAACTACTATGTTTCGG
CCTAATCATTTCCAGGTGTATTTCAAGGGAAACTGCTACGGGTTGGCAGAAGAAATCAAGAAGGAATTCATCTCATCGT
TTGACAGTCTTGAGGAGTATTACATTAGGGAAGTAATCGTTTGGTTTAACACGAGCGATCGGGTTTTCCATAGTGCATT
GACTCCTGATTGTTTGTATCGCTATCCAGCGCATGACTTCAATCTTACAGTGTGGAACAATTGCGTTGCCCTTTTTGGC
TATAACCGTTGTGGAAGTAAACCCTTTGAAATTTGGGTGATGGGTGAATCTGATGGTTTCACTTGTTCATGGATAAAGC
ACCTATCGGTTGACATTACGGAATCTCCTCAACCATTGGTACTTTGGGAGAGCAACCAGAGTCTTTTGGTGTCCCCTCG
TATCCGAGTAGCATTGTACAGCTTTGCAACCAAAACGTTCAAGTATTTACCACTGTGTGCTGCTGAACACTTCGATGCC
ATACCTTTTGTGAATAGTATAGTTCCACTCAATAGGGACCTAGTATCTGTTAATATTTCT
>Rchinensis_NC_037090_F_box_minus1_5475947_5477151_
ATGGCAGAGTTTTGCAAAATGCCAGAAGAGATGGTGGTGCCAATCCTATCACGGTTGCTTCCGAAATCTCTAATGCGAT
TCAAGTGCATCCACAAGTCATGGCATTCTCTGATCAATAGTCCCCAGTTCATATCCAAGCATCTTCACTTTCACAACAA
CTTGTCTTCCTCCACTACCATCCTCTTAAAGCGTCCTGTAATGCGCAGAACCGATACTTTGAATGAGAAGATTGTTTGT
TCGTTCCTTAATCTTCACAATGCAAATGATGGTGATGAGGATAACCTTCATTATGATATCAAGGACCTCGAATTTCAGC
CTTCAATGGGTTTAAATACTAGGGGACAATTTATTGAAATACCTAATGAATATTATTATAATTGTGCATATATTATAGG
TCAATGTGATGGGATATTTTGTCTAACTCTTTATGCTGCAAAAGACCTTGTTTTGTACAATCCAGCAATCAAAGAATTC
AAGTTTCTTCCTGAGTCATGCCTTCAAGATAAGAATATAGGTTCTGTGGGATTTGGCTATGATCCCAAGTCTGAAGATT
ACATACTCGTTAGCGTTGTAAGTTATGGGCATGGGGAGCAATATTACAATGATGATCGTCTCGTTATTGATCCTATGAG
AGCAGAAGTTTACACAATGAGTACCGATTGTTGGAGAGAGATCAAGATTCACAATCTGGAAACCGAAACTACGTTTTTT
TGGCCTAGACATTTCCAAGTGTACTTTAAGGGTAATTGTTACTGGTTGGCACATGAAAAAAGGAAGGAATTCATCACTT
TGTATGACAGACTTGAGGAGTACTACATTTGGGAAGCAATTGTTTGTTTTGACACCGCCAATCGGATTTTTCATAATAT
ATTGGTTCCAGATTGCTTGTACGAATTCCCAATGCATGACTTGGACCTTGCAGTATGGCACGATTCCATTGCTCTTTTT
GGCTTTTATCGCGGTGGAAGTCGACCCTTTGAGATCTGGGTGATGGATAACTTCGATGGTCTCAACTCTTCATGGGTAA

AACAGCTATCCGTTGACATTGCGAAATCTCCTATACCGTTGGCACTTTGGGAAAGGAATAAGATTCTTTTGGTTTTCAC
ACATACACAAATAGCTTTGTACAGCTTTGTAACTGAAACATATCAGTATTTACCACTTTATGGTGCGAGTTTTTTCAGG
CTTTTCCTTATGTGGATAGTA
>Rchinensis_NC_037090_F_box_plus1_5558222_5559472
ATGGTAGAGTTTTGCAAGATTTCAGAAGAGATAGTGATGCAAATCCTATCAAGGACGCCTCCTAAATCTCTAATGCGAT
TCAAGTGTATCCAGAAGTCATGGAATTCTATGATCAATGATCCACAATTTGCAGCTAAACATCTTCACTTTTACAACAA
CCCATCTTCATCCACTGCCTTCCTTGTCAAGCGTCCTGTCATCCTCAGAAGCGAAACAAGCAATGAGAACGTTGTACTT
TCATATCTTCGTCTAGAAACTTATACTAATGGTGATGATGAAGACCTTCATTTCGTAGTTGAAGACATCATTTGTCCAC
CTTTTAAGGGTCTAAAGGCTCGGGGCCAATTTATTGAGCTCCCTAGACGTGATGATTCTGTGTATATCATTAGTCATTG
TGATGGCATCATTTTTCTGACTCTGTATACCGGCGACCTTTTCTTGTACAATCCAGCAATCAAAGAATTCAAGATTATT
CTAGCGTCATGTTGTCATGATTGTTGTTGGAGCACGGTGGGATTTGGATATGATCTCAAATGTAAAGATTACATAATTC
TAGAAATTGCTTGTTATGGTGAGACAAATTATAACGATCCTCAGCGTCTCGTTGTTGATCCTCCTATAGCTGCAGTTTA
CACACTAGGCATTGATTCTTGGAGAGAAATCAAGACTGATCACTTACAAACTGAAGATACCTACTTTTGGCCTACCGCG
TTCGATTTGTACTCGAAGGGAATTTTCTATTTGTTTGGTTATGAGGAAAAGAAGGAGTTCTTGGATGACATGGAAAGAT
GTGAGGAGACAAACAAGCAAGTGATGATTTTGTATGATACAAGAGATGAGCTATTTCATATTGCAATGCTTCCGGATAG
TTTCAATGAGCCTGCATGCGGCGTTCATGATATTCATGTTGCATTGTTGAATAAGTCCATTGCTCTTTATGGGTTTAGC
ATTTTCGAATCAATTCATTCCATTCAAATATGGGTAACGGATGACATTCGAGGCGCTCAGGAATATTCTTGGACAAAAT
ATTTGTCCCTAAATCCGGTAGATAATGTTCGGAGGTCATTGGCCTTTTGGAAGATCGACGAGGTTCTTATGATTGCCAA
AGATGGACGTGTAGTCCTCTGTAACCTCCTTACCGGAAAACTCAAGTATTTTCCCATTCATGGCCTGCACCTAGGAGAT
GATATTCAGGGCATTGTTTGTGTGGATAGTATAGTTCCACTTAATGGAAGAGAATTATCTAGACAT
>Rchinensis_NC_0370909_F_box_plus2_5618754_561998
ATGTCAGAGTTTTCCAAATTTCCTGAAGAGATGGCCTTGCATATCTTATCAAGGATGCCACCTAAATCTCTGATGCGAT
TCAAGTGTGTCCGTAAGTCATGGTATGTGTTGATCAACAATCCCAGCTTCGTGGCCAAGCACCTCTATAATTCCTTGCA
CAATAAACAGTCCACATGCATCTTTTGCAAGCGTTACGTCTTCAGGGATATCGCCACTAAAGATGTGGAATCTGTAGTC
TCATTGATTACTTTTTCTGATGATGATGTTGGTGATACTAACCATGAGCATATATCTCATTCGGTTATCCAGGACATCG
ATCTTCCACTTTCTATGAGTGGAATACCGAAGAACCATTTAAATGAGCCTGAGCTTCTCGGAGCTGTATATATTACCGG
GCATTGTGATGGAATCATTTGTTTAGTCCATGGTGAGATTGTGCTATGGAATCCAGCAATTAAGCAATTCAAGATTCTT
CCCAAGCCACTCCTTACAAATGGGATCGTAAATTCTATAGGATTTGGCTATGATGCCAGATCTAAAGATTACAAAGTTT
TTAGTTTTCCAACTCATGATGAGGACCGAAGTAGCGAGCGTGATTTTAATTATCCTCCACATGTTGAAGTATACAGCCT
CAGTACCGACTTGTGGACAGAGATCAACGCCGATCATTTAGAAACTGAAACAACCAACTTGTATCCTGAATTTTTTCAA
ATGTACTTCAAGGGAATATGGTATTGGACCGGAAGTGAGCAACAAAAGGAATTCATGGTTGTTTATGATAGTATGGATG
AGGAATGGGTGAGGCAGCTGATCATTGTGTTTGATATGAATGATGAAGTATTCGAAGACATACTATTTCCGTATAGTTT
ATACGGCCCGATGATTCCATATTTAGAAATGCGGGTTATTGTGTGGAATGAATCCGTCGCTCTTTTTGGTCAGTACCGT
TTTGGTTATGCTGATGATGCTTTTGGATTATGGGTAATGGATGATATTGTCAAAGGTTCTTGGACAAAACAATTAACTC
TAGAGGTCGTAGTTGGGACTCGGATGACTTTGGAAATGTGGAAGAGCGATGAGATTCTTATGGTGGCTAATGACAACCG
TATATTCTCCTACAATATCAGAACTGAAGAGATTAAATATCTTCCCATTGAAAGTACGCATCCAACTTTTTCCGCAGCT
ATTGTATGCATAAACAGTATAGTTCCGGTTATTCATGGGAGGCAACAAGCA
>Rchinensis_NC_037090_F_box_plus3_5720698_5722023
ATGGCAAACTTTAGCAAATTGTATTCGTCTGAAGACCTTGTGGAGCAAATTCTATCAGGACTGCCTCCCAAATCTTTGA
TGCGATTTAAGTGTGTGTGTGATTTGTGGTGCAATTTAATCAAGAGCCCTAGTTTTGTAGCTAAACACCTTTCGGGATC
TATGCGAGCATCCTCTATGCCCGTTCTTTTCAAGCGCCCAGTCCCCAGGGACAAGGAAAATAACATTATGGATGAGAAG
GGAGTTGAGAATGACGACGACGATGTCGGAACTCTATTGTGGTCACTTAATCTCTGCAATGAGGATGATAATGATTACC
TTCTATCAACCGTACTTGAGGAACTTAATGTTCCGCTTCCGGCTCCTCTTAAGCTAAAACATTCCTCGGATCTGACAAT
TGCAGGTCATTGTGATGGAATCATTTGTTTAAAACTTTTCACTGGTAACGTTATTTTATGCAACCCAGCTATGAAGGAA
TTTAAGCTTCTTCCCAAGTCTTTTCTTCTCCTTTGCAATGATGACTTTGATGATCTCTGGTCGCTTTCCTATGAATTAA
GATATTACACTGAACAATTGGGATTTGGCTATGATCCCGAAGGTAAAGATTACAAGGTTCTTAGATTCGTAATCTATGA
TGAGTCATGTTATTGGTTCAAAGCAGAAGTATACACTATGGATTCTAATTCTTGGAGAGAGATCAAGACCGAATATAAT
AATATAATCCAATTTGTTAATTGGTCTTCTGATCAGCCTATATACTTCAACGGAATATGTTATTGGCAAGTAAGTGGTT
CAAGGGGGGAGTTCATTCTATCATTTGATATGGGTAACGAGCTATTTCATGAGATATTGAATCCAGATTTGCCAGATAA
ATGTGGAGTGGTGAGGCTTGCAGTGTGGAAAGAGTTCATTTCTCTTTTTACCTATCAAGAAGAAATTGTAGTTCCTCCA
TCTTACGATATGTGGGTGATGATGGATGATCTTGGCGATGGCAAGGGTTCATGGACTAAATATTTTACTATAGGACCTG
TCGAAGGTGATAAATGGCCATTGCTATTTTGGAAAGGTGACCAACTGTTAATGGAAAGTAACGATGGACAGATCGTCTT
ATATAACATCGGCACACAAATATTAAAGTATCTTCCTATTCATTTCATCAGAGATCTTTACTATAGTCAAGAACTTGTT
TATGTAAATAGTATTGTTTCCATCAACGGAGGCAATGTACTTGAAGATATACATATTTCTGCATTTTATGGCAATGGCA
AGTTTTATTCCATCAACAAAGGAGACGTAATAGATATTTCTGCTTTTTATGGCATTACAAGT
>Rchinensis_NC_037090_F_box_plus4_5734186_5735463
ATGGCTGAGCTAATTTGCAAAATGCCGGAAGAGATGACATTGCAATTTCTTTCGAGGCTGCCTCCCAAATCTCTGATGC
GATTCAAGTGCATCCATAAGTCATGGTATGCTTTGATCAATAATCCCAAGTTCATAGACAAGCACCTCCACTTGTACAA

CAAAGACTCCTACACTTGCTTCCTTCTCAAGCGCTCCGTGGTTGCAAGAACCCAAAGTATCAAAGAGGAGATCTTATTT
TCATTCCTTTATGTTCCGAATGACAATGATGATGAAGACAGTCATCCTCATTGTGTTGTGGAGGACATCTATTTTCCGA
CTGCTATGGGTCTAAAGACTAAGGGGCACAATATTGAGCTCCCTGGGTCATACGGTGGTGAAACTATATACATTTTAGG
TCATTGTGATGGGATCATTTGTCTAGTTTATCATAGCGGAGGCCTTGTGTTCTACAACCCATCGATACGAGAATTCAAG
ATTATTCCCCCTTCATGTCTTACCGAGTCATTCTCTTGTGTTGGGGGATTTGGATATGATCCAAAATGTAAGGATTACA
AGGTGGTTAATATTGTACCATCAGGTGAAGATTCATATGATCATAACCAGCGTCTGGTTATTTATCCTCCAAGGGCAGA
AGTATACACTTTGAGTACTGATTCTTGGAGGCAAATCAAGATTGATTATTTAGAAACAGAAACTACTAGCTTTTGGCCT
GACATTTATCAGATGTGCTACAAGGGAGTTTTTATTGGTTGGGTCATGAACAAGATAAGGAATACCTCTGTTATTATG
ACAGGCTTTCGTCGCCAAGCATTAGGGATGTAATCCTTTTGTATGACACTGGTGAAGAGGTATTTCGTACTAGACTACT
TCCGGATAGTTTCAAGGACCTAGGATTGCATGCTTTATCTATGAGTCTAACAATGTGGAATGGATCTATTGCTCTTTTT
GGCTTTTCTTATTGGGGTCCGGATATTGAGTCCTTCAAAATATGGATGATGGATGACTTTGGCAGTTGGACAAAACACT
TGACCTATGAGACCATAATGGGAATTTATTTGTCATTGGTGTTATGGAGAAGTGATGACGTTCTTATGGTTGCCAATGA
CGGACGTATAGTCTCCTATAGTCTAAGTAGAGATAGGGTTAAGTATTTTCCAATTCAAGGTGTTTGGGGAACTTATCAA
GCTTTCGTTTGTGTGAATTCGAATAGTATTGTTTCAGTCAAGGGAGGCAACAAGGTTGAGAGCAGAGATATACAAACTA
GTAATGTCCTCCTG
>Rchinensis_NC_037090_F_box_plus5_5751338_5752603
ATGGCAGAGCTTTCCAAATTTGCTGAAGAGATTATGGTGGAAATCATGTCAAGGTTGCCTCCCAAATCTCTGATGCGGT
TCAAGTGTGTCCGTAGGTCATGGAATGCTTTGATCAATAATCCCAACTTTGCAGCCAAACACCTTGCTTCTTCGAAGCG
CACCCTGCTGTCATCATCCTCCACCACCATCATTTTTAGGCATTTTCTCATTGCAGACCTCAACCCTGACGAGATGGAA
ATGATACTGTCATTGTACAATTTTTGCAACGATTTTGATGGTTGCTTTCTTGAAGATATCCATTTTCCGCATTCCATGG
GTTTAGAGTGTAGGCGGAAATTTCACGAGCCGGGGTCTACTTTTGGAATTTCATGTTATTGTGATGGGATTATATGTCT
AGCTGACTATGGACAGAAACCGAACATAGTCTTATGCAACCCAGCAATCAAGGAATTCAAGCTTCTTTCCGAGTCGCAG
CTTGCCCTCTCTTCCCCGACATTCCGGAAAACAGCTGCCGTGGGTTTTGGTTGTGATCTGATGTTGAAAAATTATAAAG
TTGTCAGACTTATAAATAGTGGATGGCGGTATCGTGATGATCAAGATACGGTTATTCCTCATCTTTATGCAGAAGTGTA
CAACCTTCGCACAGATTCTTGGAAAGAAATCAAGATTGATGGTTTATTAAAGGAAAATAAAATCGTTGTGCCTGATTCG
AATGCTCAAACCAAGGGACTATGCTCCAAGGGAATTCTTTACTGGTGTGCAATGGAAGAAGAAAAGGTTTTAGAAGTTA
CCTGTGATGGTGATGATGAGCAAAAAGAAATTGATATGCATGCATGCATCATTTCGTTCAACGTAGGTGATGAATCATT
TCATGTTATAAATATTGGCTCATATGATGATCATTGTTGTTTAATAGATGGTGTACTTGGATTGTGGAAAGAATCCATT
GCTCTCTGTGTTCGTGGCTGGACTACCCTAGACATATGGGTGATGGATGACTTTGGTGGTGGTAAGGGTTCTTGGACAA
AATACTTGGCGATCGAGCCGGTAGTAAAGATTACATCTCAATTTGCATTATTTGGAAAAAGCGATGAGCAGTTTGTCTT
GGTTGCCTGTGATGATTCTGTTGTAATATTCTATGACATTTGTACCAACAAGTTTAACTATCTTCCTCTGAATGGCGTA
CTTCTGCATCATACTCAAGTTGTTGAATATGCGAGTAGTATAGTTTCAGTCAAAGAATGCAATAAGCTTGATATGGAAG
CA
>Rchinensis_NC_037090_F_box_plus6_5803902_5805155
ATGGAAAATTTCAGCAAATTGTCAGAAGAGATGGTGGTGCGAATCCTATCACGATTGCCTCCTAAATCTCTGATTCGAT
TCAGATGCGTCCGTAAGTTGTGGTACAATGTAATCAATAGTCCTAACTTCGTAGCCAAGAACCTTACTACTTCCAAGCA
CAATAAGTTCAGTTCATCCACTTGCATTCTTGCCAAGCATACTGTCCTCAAGGACAGCAACATTAAGGATAGGAATGAA
ATTTTGGAAGTCCTCAGGGACAACAGCATAGAAACAAAGAAAATTTTACTCTCATTGTGTAACCTTTGCAACGACAACG
ATGGTGATGACCCTAATCTTAATTATGTAGTCGACGACTTCACCGTTCCGCTTCCTTTAGGTCTACTTCCATTTAGTCT
AGAGATTGCAGGTCATTGTGATGGGATCATTTGTCTAAATAATTCATTTCTTGATGACATAGTCTTGTGCAATCCAGCA
ACCAAAGAATCCAAACTTCTTCCGAAGTCTTGTCTTCTTCTCCCTCCTCGACATCCAAATGACTACGATGAAATAGAGT
CTGATGTAAATGCTGTTGGATTCGGCTACGATTCCAAAGCTCAAGAATACAAGGTTGTTAGAATTGTATCATTTATTAC
TGGGGTTCATAAACCACTTCCTTCGAAAGCAGAAGTATATACCATTGGCACTAATTCTTGGCGAGAGATCAAGGATCAA
ACTGAAAGTCATGTTTTCTGGGCTGCTTCTTTTAAGTTGTTCTTGAAGGGATTTTATTTTTGGTGGGCATCAATCTGCC
CACCAGAGCAGGAAATCATCCTCTCATTTGATATGAATGAGGAGCTGTTTCATGATATATATATTCCAGAGAGTGTCCG
TCATGACATAGTTCGATGTAATAGAGGTCTTGCAGTGTGGAAAGAGTCCATTGCTCTTTTGGCTTACGGTGGAGACAGT
GGAGCTCAATCTTTTGACATATGGGTAATCGATGACTTCGGTGTCTTTAAGAGTTCATGGATAAAATACTTAACCATCG
GACCTCTAGAAGGCATTTCGATTCCATTGATATTTTGGAAGAGTAACGAGTTTCTTATGGCTGCCACTGATGGACGTCT
AGTGTCCTATAACCTTAGCACCCAAATGTTCAAGTATCTTCCTATTCATGGGGTGGAAGATCCACCATATATTCAAGCC
GTTGTTTATGTAAATAGTATTGTTTCGGTCCATGCAAGCAACAAACTTGAGGGCATAAATAACTCTAGC
>Rchinensis_NC_037090_F_box_plus7_5808024_5809247
ATGGGGAAGCTTTGCAAATTGTCAGAAGAGATGGTCGGGCAATTCCTTTCAAGATTGCCTCCTAAAGCATTGATGCGCT
TCAAATGTATTCATAAGTCGTGGTACAATCTAATAACTTCTCCTAGCTTTATAGCCAAGAACCTTTCTAATTCCAAGAA
CAACAAGTTTGCGTCCACCACTAGAATCCTTTTCAAGCGTACTGTCCTCAAGGACATTAAGGATAAGAATGAAATATTT
TATGTCCTTAGGGACAACAACAATGATAGGAGATACATTTTCCTTTCTTTACTTGATCTTTGCAACGATAATGATGGTG
ATGACCAAAACCTTCATTCTGTTGTCGACGACCTTATTGTTCCACTTCCTTTTAGTATATGTCCCTTTAGTTTACAAAT
TGCAGGTCACTGTGATGGTCTTATTTGTCTTGTTAATATTGTTAATGAGGAGGTTGCTTTGTGCAATCCAGCGATAAAG
GAATTCAAATTTCTTCCTAGGTCTTCCCTTCTTCTTCCTCGCAGACATCCAGAAGATGATGACGGCATAGAATCGGATG

TAAATGCTGTCGGATTTGGCTATGATTCTAAAACTCAAGATTACAAGATTGTTAGAGTTATAACATATATTACAGGGAT
TGCTTATACACTTCCTTCTAAAGCAGAGGTGTACACATTGAGTTCTCATTCTTGGAGAGAGATCAAAATTGATAAAGAA
TGTCATGTCTTTTGGACTCCTTCATTTGAGATACACTTCAGGGGAATTTATTATTGGAGTGCACTGACTTATCCTACTC
CAGGAGCGGATAAGGAAGCCATCTTTGCATTTGATATGAGTGAAGAGACATTTGAAGAGATACCGATTCCAGATGGTAT
TTGTGCCAGAGACGGTATTATTAAGTTCCTTGCTGTGTGGAAAGAATCTGTTGCTCTTATTTCTTGCATAGGAGATGGT
CCTAAATCCTTCGATATATGGGTAATGGATGACTCTAGTGGGATTAAAGGTTCATGGACAAAACACTTGGTCATTGGAC
CTATAGAATGCGAGATTCCATTGGTATTTTGGAAGAGTGATGAGCTTCTTTTGGTTATCTCTGATGGACGTGTGGTCTC
CTATCACCTTGGTAACAAAACTATCAAGTATCTTCCGATTCATGGCGTGGAAGATCCCCAATACATCCATGCTGTTGTT
TGCGTAAATAGTATGATTTCAGTTAAGAAGACCAAAGGT
>Rchinensis_NC_037090_F_box_plus8_5856394_5857662
ATGATGCAATCTTCCAAATTGGCGGAAGAGATAGTCGTACAATTCATGTCGAGATTGCCTCCTAAAGCCTTGATGCGAT
TCAAATGTATTCGTAAGTCATGGTACAATCTAATAAATAGTCCAAGTTTTGTGGCTCAGAATCTTTCTTATTCCATGAA
CAACAAATTCACCTCTTCCACTTGCATCCTTTCCAAGCATACTGTCCTCAAGGACGGTAACATTACAGATAGGAATGAA
ATTCTTGATATCCTTACGTATGGAAACAATGATAAGCAGCAAATTTTACTGTCATTGCTTAACCTATGCAACGATCATA
ATGGTGATGATCAAGAGCTTTTTTCTGTTATTAAGGACAACTTTATTGTTCCTTTTCCTTTTGATAAATGTTCGTTGAG
TTTAAAAATTGCAGGTCATTGTGATGGGATTATTTGTCTTGTTAACGTTGAGGATGTTGCTTTATGCAACCCTTCAATC
AAGGAATTCAATCATCTTCCAAAGTCTTGTCTTCTTCTCCCCCCAAAAAATTGGGATGATTATGAAAATGAAGATGATT
ATTATGAGGCATTAGAATCGGAATCAAATGCTGTGGGATTTGGCTATGATTCCAAAGCTAATGTTTACAAGGTTGTTAG
AATTGTCCAGTTTACTTCAGGGTATGTTTTTACTTCACATCCTTCAAGAGTAGAGGTGTACACCTTAGGGGCAAATTGT
TGGAGAGAGATCAAGGCAGATGTACTTGTAAGTACTGTCTGTTGGAGTCCTTCATTTGAGATGTACTTCAAGGGAATTT
ATTATTGGGATGCGTATAGCTATCTTACTCCTAGACAGTATAAGGACGGCATACTTGCATTTGATATGAGTGATGAGCT
ATTTTATTTGATATACCATCCGGAGACTACACGTGAATTTAACAAGAGCCTTGCTGTGTGGAAAGAATCCATAGCTCTT
ATCACCTACGAAGGAGATGCTCCTAAATGTTTCGATTTATGGCTAAATGAAGACTCTAGTTGTTTTAAAGGTTTATGGA
CAAAATACTTCACTATTGGACCGGTAGAAGTTGAGATTCCTTTAGTATTTTGGAAGAGTAACGAGATTCTTATGGTGAA
TGCTGATAAACATATAGTTTCTTATAACCTTGATACCCAAACACTCAAGTGTCTCCCAATGCATGGAGTGGAAGATCCT
GAGTATATTTATGCTATTATTTATGTAAGTAGTATTATTTCAGTAAACAGAGACAATAAGCTCGAGTGTACGACTACTT
CTATT
>Rchinensis_NC_037090_F_box_plus9_5879526_5880743
ATGACAGAGTTTTGCAAGATACCAGAAGCCATGGGATTGCAAATCCTATCAAGGCTGCCACCTAAATCTCTGATGCGAT
TCAAGTGCGTTCATAAGTCGTGGCATACTCTGATGAAGGACCCCAACTTCGTGGCCAAGCATCTTTCCAATTCCATGCA
CGACAATTTCTGTAGAACTACTGGTGTCCTTTTCAAACGTGAAAACTTCAAGGATACTGGCACTGCCGAGAGGCAAAGC
GAAAGTCTTTTGTCATTGATTAATTTCTGCAATGCTAATGGTGATGGTGAGCATGATATTCATTGTCTTGTCGAGGATG
TTACGAAGGGTCAGTTTAGCGGGTTCGAAGTTCTAGAGTCTGCATGGATTATAGGGCATTGTCATGGGATTATCTGTCT
AAGAAATGCTACTAAAATAATTCTATGGAACCCAGCAATTAGGGAAGTCAAGGTCACTTCGCCATATGTTCCAGATGAG
AACTTAAGTGATTTGGGAATAGGCTATGACCCTAAATCCGACACTTACAAAGTTGTTCACATTTCTTATGGTACTCAGG
AAGAATATGGTGATGGACATATCCTTATCGATCGACCCAAAACAGAAGTATACACCTTAGGTACTGATTCTTGGAGACA
GATCATGACGGGCTGTTTAGAAACGGAAACTACTCACTTTTGGTTTCAGGATTTCCACATGTACTTCAATGGATTTTGT
TATTGGAATGGGCGCGAGCAACTGAAGGAATACCAAAATTTTTATGATCTTCAAGAGGAGCATCATATTAGGCCAGTGA
TCATTTCGTTTGATATGGGTGATGAGGTATTTCATAATATGTTGCTTCCAGATTTTGTATATGAGACCTATATGTGGAG
TTATGTTTTGCGTCTTATGGCATGGAATGAATCTGTAGCTATTTTTGGCCTAGATCATGGTATAACTTCTCATGAATCT
TGGGGATTATGGGTGATGGATGACTTTGGTGGAGTTACGGGTTCTTGGATAAAGCAATTTAGCTTTGTGAGCGCAGTGG
GATTCTTGGATACGCCATTGCAAATTTGGAAGAGCGATGAAATTCTTATTGTTTCGAAAGAAAGGCGTGTCGTCTCATA
CAACCTTGATACTGAACAGTATAAGTATCTACCCATTCATAGCATGGATTCTGATTATTTTGAAGCTGTTGTTTACATG
AATAGTATAGTTTCGGTCAATGGAAGCAAAACC
>Rchinensis_NC_037090_F_box_plus10_6536594_6537988
ATGGCACTTGAGAAGGATTCAGAGATAGCAGAATTGACAGAGTCTGGCAAAAATATTGCGCAAGATGTGGTGGAACAAA
TCCTATCAACTCTGCCTCCCAAATCTCTTATGCGATTCAAGTGCGTCTCTAAATGGTGGTACCATCTCATCACCAGTCC
CAGGTTCGTAGCCAAGCACCTGTCCATTTCCAAACACAACAGACCCTCAACTTGTGCTCTTATAAAGAGTTTAGTCAGC
AATGACGCAGAAGCTCAAGAGCCTGAAATGGTTTTCTCATTGCTTAACTTTTCCTATGAAAATGATAATAACGCTGGTG
GTGCGCTTAGCACTAATCTTTCTAGTGTGGAAGACCTCACAATCCCTACAAGGGTAGTCGAATCACTTCGTATTATAGG
CCATTGTGATGGGATTGTTTGTCTAGCTTTAATCGATTATCAGCAGAGGCTAGCTAAACCTAGTCAAGTGTGTCTATGG
AATCCTGCAATTCAGCAATTTAAATTTCTTCCCGAGGAGCCATTCCTTCCAGATTGGTCCAAGGTACCACACAGCCGTA
TGGTCCAAGAATTTGCTTACCTGCGTCCAATATCTCTACTCAATGGTGAAACCATGGGGTTTGGCTATGATCCTAAATC
TAAAGATTACAAGGTTATTGACATTGGATTTTCTGATTCCAAATTTTATGGTGATCCAGAATGTTATGGCGGACATGTG
ATTGTTTATCCTCCAAAAGCAGTAGTATACACCCTGCAAACTGATTCTTGGAGAGAGATCAAGACTTTTTCTTTGGAAA
GGGAAACCAGTTACCTTTGGCCTGATACGTTCCAGCTATACTTGAAGGGTGTTTGTTATTGGTTGGGATATGAGCAACA
AAAGGAATTCTTATGTCTATTTCAGACTCACCAAGAAGAGGAAGAACGCATTGCGCGAGCGATCATTTCGTTTGATACT
AGTGATGAGGTTTTTCATGATATAATGTTACCGCATGGGCTACTGGAATTTTACGGTTTTGATAATTTCCTTACATTGC

ATCTTACGGAGTGGAATGAGTCTGTTGCTCTTTTCAGCTTGCTTTTTGAGGATGAGCACAAAGCTACAATGTGGGTGAT
GGATGCCAAAGGTGCTTGGACAAAGCAATTAACTTTTGAATATGTAGACTACTTTCCTTACAGCTTACCTCGAAAGATA
TTGGCATTTTGGAAGAGCAACGAAATTTTTGGAGTTGGAGAAAATGGATCTATTGTCTGCTATAACCTCAATACCAAAA
TTGTCAAGCATCTTCCGATTCGAAGTGTTCCAGATTATTTTCCACCCTCTAGAGACACTTTCTATCCTTTTTGTTGTAT
TGCTTATGTGAATAGTGTCGTACCAATCATGAATCATGTCAGAGAGCACATT
>Rchinensis_NC_037090_F_box_plus11_7143024_7144325
ATGACGTTGGAGTTTGGCAAAACTATTGATGAAGATGTGGTGGAGCAAATCCTATCAACTCTGCCGCCCAAATCTCTGA
AGCGATTCCAGTGCGTCTCTAATAGGTGGCATGCTCTGATCACCACTCCCAGGTTCGTAGCTAAGCACCTCTCCATTTC
CAGGCACAACAATCTCTCCACCAGTGTTCTTATGAAACGTAAAGTCCATGAGGACACCAACTCTGACGAGACTCAAGAG
TTTTTCTCATTTCTTCATTTTCGAAATGATGAAGATAATGACGTTGATGGTGTGCATGATGAGCATAGCTTTCTTTCTA
GTATCCAGGAATTCCATATTCCGTTTTCTACTGGTGTAAAGACTTGGGCCGAAGCACTTATAATTATAGGCCATTGTAA
CGGGATCATTTGTCTAGCTCAAGCAGTCTCTGGTGAGGTGATTATTTGCAACCCAGCAATTCATGAATATAAGCTTCTT
CCCCCCTCTCCGTACCTTCCAGATTCCGATTGGCCATATAGCGCCATATTTCGGTTCAGAGATGGATTGGGATTTGGAT
ATGATCCGAACTTTAACGAATATAAAATTGTTAACATTGGATTTCCTGCTCCAGAATTATCTACGCCTGATGGATATAA
CATTTATAATCCTCCCAAAGCAGCTGTCTACACCCTGGGTACTGATGCTTGGAGAAAGATCAAGACTGATACGTTAGAA
ACAGAAACTACTATTCTTTGGCCCCAAATATTCCAGATGCACTTCAAGGATATGTGCTTTTGGCTGGCACCTGAGCAAC
ACAAGGAATTGGATGTGCTTGATGAAGACGAGGAGCAATTCATTAGGGAAGTTATCGTTATGTTTGATACTGGGGATGA
GCTATTTCATAATATAATGTTACCGGATGAATTTGATTATCCATCAAAAAATTATTTTGTTCCGAACCTTTTAGTGTGG
AAGGACTCCGTTGCTCTTTTGGGAATACAAATTTCTCAATTTTCATCATATGGAATATGGGTGATAGATGAATTTGGTG
GTCATAACGGTGGTGCTTGGACAAAACACATAACTTTTGAGCTCCCTGTGGAACCGTTGATATTTTGGAAGAGCGACAG
GGTTCTTTTAAATGATCCTAACGACACTGATTATAGAGGACTTATATTAGATTATAATCTCGATACCAAAAAGCTTAAA
AATCTTCCCGTTCAAAGCGAGCGGAGTGACTCTTCTGCTATTGTGTATGTGAGCAGTATAGTTTCAGTATTGGGAGGCA
GCAAACCCAAGAACAAAGATAATTCTACACCCAATGTA
>Rchinensis_NC_037090_F_box_plus12_7149272_7150558
ATGAAAGAGTTTGACGGTGAAGATGTAGTGGAGCAAATCCTATCAATTCTGCCACCCAAAACTCTGATGCGATTCAAGT
GTGTCTCTCAAAGGTGGTATGCTCTGATCACTAGTCCCAGGTTCGTTGCCCAGCACATCTACAATTCCATGCACAACAA
TCTCTCCACTAGTGTTCTTATGAAACGTGAAGTCCAGAAGGACACCAACACTGACGAGACTGAAGAGTTGTTCTCATTC
CTTCATTTTCGAAATGATGAAGATAATGATGTTGATGGCGTGCATGACGAGCATAGTGTTCTTTCTAGTATCCAGGAAT
TCCATATTCCGTTTCCTACCGGTGTAAAGACTCCGAACGAATCGCTTATAATTATAGGCCACTGTAACGGGATCATTTG
TCTAGCTCAAGCAGTATCTGGTGAGGTGATTTTATGTAACCCAGCAATTCATGAATATAAGCTTCTTCCACCCTCTCCA
CACCTTCCAGATTCCGATTGGCCATGTAGCCCCTTATTCCGGTTCAGAGATGGTTTAGGATTTGGACACGATCCGAACT
TTAACGAACATAAAGTTATTAACATTGGATTTCCTGCTCCAGAATTATCTACACCTGATGGATTTAACATTTATAATCC
TCCCAAAGCAGCTGTCTACACCCTTGGTACTGATTCTTGGAGAGAGATCAGGACTGATTCTTTAGAAACAGAAACTACC
ATTCTTTGGCCCGAACATTTCCAGATGCACTTCAAGGATATGTGTTTTTGGCGGGGACATGAGCAACATAAGGAATTGG
ATGTGTACGATGCCCGCAAGGAGGAATTCATTAGGGACGTTATCATTATGTTTGATACTGGGGATGAGCTGTTTCATGG
TATAATCTTACCCGATGAATTTTATTTTCCATTAAAGAATTATTTTGGTGGGCACCTTTTAGTGTGGAAGGACTCTGTT
GCTCTTTTGGGAAGACAATTTCGTGAACTATCCTTTGGAATATGGGTGATGGATGAATTTGGTGGTCCTAATGGTGGTG
CTTGGACAAAATACGTAAGTTTTGAGCTCCCTGTGGAACCGTTGATCTTTTGGAAGAGCGAGGAGGTTCTTTTGAATGA
TTATAACGGGACTAAGTATAGCGGACTTATATTCTCTTATAATCTCGGTACCAAAAAGCTTAAAAATCTTCCCATTCAA
AGCAAGCGGACTGACTCTACTGCTGTTGTGCATGTGAACAGCATAGTTTCAGTCTTGGGAGGCAACAAACTCAACAGCA
AAGATAATTCTACACTCAATGTA
>Rchinensis_NC_037090_F_box_plus13_7425762_7426949R
ATGGATTCTTGTGTTTGCAATTTGCAAGATTTCAGTTTCACTCTTTCGTTTCTTGGCCATACTTTAATAATTATGTTGT
TCAGGATGAGAAACAAACGCAGGGAGGATGTTGCCAGTTCCATCACTGAAGATCTCCACATGGAAATCTTAGCAAGACT
CCCGGTAAAATCTCTCATTCGGTTTCAGTGTGTATGCAAGCGGTGGAGGGGTTTGATTCGCAGCTCAAGTTTTGTTACA
GCACATATCAGACAACATTCCCATACCCATCTAATTACGGATGACCGCTCAAGTTTTGACCAGGTTGAGCTTTGTTACT
CTCTTTTTCATTGTGAAAGATTTGAGCAGTCCCTGGAGTTAAAACCTCCCTTTCATCCCCAACTAGACTATAATTCCCG
ACTAGACTACTTCAAGATATATGGTTCAAAAAACGGTTTACTGTGTCTTGCTGGGAATGTAATGGATTCAAACAGCCCT
ATATACATATGGAACCCCTCTGTTAGGAAATTCAGGACTCTTCCACAACCAAGATTCGATCATTATTGTGTGGACTGTT
TTCCCAAGCATGTTGATATCCCTCTCTTATTTGGATTCCACCCAGAACTAAATGACTATAAGGTGATAAGGATGATGCT
GCCTGGTTCCATCAAAATGGAGGTTTATACTCTTAGCACAAACTCTTGGAAGATTATTCAAGTCATTCCACCTTGGCTC
AATACCATGAAGTTTTCTACTCGATCTGAATTTTGGAATGGAATGGCATATTGGCTTGCCACCAAAGATTCAATGGTTA
GGGTTGTGTTGTTTGATACTAACAATGAAGAATTTGAAGAGCTAGCGGTCCCAATTACGATTTTGAGTGATGATTGGGA
TGCATATATCGAAGTGTACAAAGAATCGATTTGCCTACTTCATGTCAAAGTTGAACTGGATAACGACGTTGGCTGTGAC
TCTATCGACTTCTGGGTTCTGCAAGAACAGGATTTTAAGAAACTGCATACTGTTTATTTTCCTCAAGTATATCATGTCA
TTTGGGGCTTTAGTGTGGATAATGAGCTCTTACTAGAAGATTATTCATCTGATGGCGAATTGGCGTTTTATAATCTTGA
ATCCAAACAGGTTCAGAAAATTCGAATCAGTTCCTATTTAAGCGTACATACTTACATTGAAAGTTTAGTTTTATTTGAT
ATG

>Rchinensis_NC_037090_F_box_plus14_9568955_9570373_
ATGGAGATAGACGACGTGAATATGTCAGGGGTTCACAAAAACATTGAACAAGACGTGGTGGAACAAATCCTATCAACTC
TGCCTCCCAAAACTTTGATGCGATTCAAGTGCGTTTCTAAAAGGTGGTATGCTCTCATCACCAATCCCAGGTTCGTAGC
TAAGCACTTGTCCATTTCCAAGCACAACAATCGCTCTACTCCTTCCGTCCTTTTGAAGCGTTTAGTCCATAAGGACAAT
AACACTGATAGTGAGACTCAAGCGGTATTCTCATTGCTCAAATTTCGCAATAATATTGATGGCGGCGGCGAAGCGCATA
GCTTTCTTTCCGGGGTAGAGGACATTGACATTCCTTCTTCTTTGAGTCTAAAGACGGGAGGCTCATCACTTCATATTAT
AGGCCATTGTAATGAGATCATCTGTCTAGTTCCAGCTGTTTCTGGTGAGGTGATATTATGGAACCCAGCAATTCATGAA
TTTAAGCCTCTTCCCCCTCAGCCTTACCTTCCCAATTCCCCAGAAATAATTGGTACCCTGCCGGGTTGGCCTAAAGATG
TTCCCTACAGTATACATCGTGAGTACATGGATAGTTTGGGATTTGGCTATGATCCCAGGTCTGATGACTACAAAGTTGT
TAACATTGGATATCCTTGTCTAGAAAGATCTCTTGATGGATATCGTATTAATTTTCCCCTGAAAACAGCTGTATATACC
TTGAGTACCGATTCTTGGAGAGAGATGAAGACTTTTTCTTTGGAAACACAATCTACTATCCTTTTCCCTGACCGCTTCC
AGATGTACTTCAAGGGAATGTGTTATTGGTCTGGACTTGAGCTGCATAAGGAAGTACATTTGTTTGACGCGATGGAAGA
GGAATTCATTAGGAACATTATCATTTTGTTCGATATGGGTGACGAGGTATTTCATGATATGCTGTTACCGGGTAGTCTA
TCTGATCCATTTGAGGTATCTTTTAATATGCGCCTTCTAGTGTGGAATGAATCCATTGCTCTTTTTGGATTGGAAAGTG
AGAATTCACCGTATGGTGTATCGTTTGGAATATGGGTGATAGATGAATTTGATGGTCCTAAGACCCCTTGGACTAAACA
TATATGTTTCGAGCTCAATGAAAAGCCGTTGGCATTTTTGAATAGCGATGAGATTCTTATGGAGGTGATAAAGGACACA
AGTGGACGTCTATTCTCATATAACCTTAGTACCAAAACTCTCAAGTATCTTCCCATTGATAGCGTGCAAAATGATTCTG
CAGCTGTTGTCTATGGGAATTACAGTATAGTTTCAATCTTGGGGGGCAACAAGCTGGAGAACAAAGATAATTCTACAAA
TGTAATTTCTAACGATGTTTTCTGTTCAAGTCTTAGACAAAAATATGCATCAATAACTATGTTTAAATTATATGTT
>Rchinensis_NC_037090_F_box_plus15_14254770_14255927
ATGGAGGAAGCAAACCTCCCCGAAGAGATTGTAGTGCTTATCCTCTCCTGGCTGCCCGTGAAATCCTTACTCCGATTCA
CCTGCGTTTCCAAACGCTTTCATTTCATCATTCTCTCTAACCCAAAATTTGCTAAATCCCAATTTCAAGCAGCTCGTGA
GCGCAAAACCCTGGATCAGAGACTCCTCTACAACACAACCGTCCCTCCACTCGAATCCCTAGACTTGGAGACGCTGCCG
TTATTTGGAGACTCTTCCTACCTCGAACGCAGACTCCTCTACTCCAAAATCGCCCCTCGACTCGAATCCCTACACTTGG
AGACGACGTCTTTTGGAGACCGTTCCTCCGTCAGAAAGCTACAGTTTCCTTTCCAGCCACCCGTCGGTCGTCTCTCGCT
ACTGGGCTCCTGCAATGGTATTGTATTTCTTGCCTTTGATGAGAGAGTATTCTATATCTGGAACCCATCGACTGGATTC
TTTAGGAAATTACCTGATCCAGGTTCTTTATGTGATCAAAGATTTCTGTTCAATTATGGTGTTGGCTATTTGTCGGCCA
CCGACGACTACAAAATTTTCATAGCCACCGATGTGTACGCCATTTTCTCGTCGAGAGCTCAAGCTTGGAAAAAACTTGA
GGTCTATTCGGAATCACCGCCTTCTTTTCGGGGGACCCTTTTGAACGAGGCACTTCATTGGCTCAATCAAGAGAACGGT
ATCGTGGCTTTTGATTTGGCCCAGGAGAAGTTCAGTAAAATGACAATGCCTAATTTCGATGAAAGAAGTTTAAATCAGT
TCGGCTATCTTGGGGTTTCTGCTGAAGGATGCCTTTCTTTTGCTCTCTCTCTGTGGGATGCTCATGACTGTATTCAAGT
GTGGGTCATGAAAGAATATGGTGTGCATGACTCATGGACTAAGCTCTATAACTTCAGGTTTTTGGATCCTCCAGAGGGG
ATGTGGTCTTTCAGTCGTGTATTGGTTTTGGAAACTAGTATTGTTGCCCACATATGCACTATGAAGGAGGTTGACAATG
ATTGTATTACCGACGTGAAAGGGTTGATAAAGATTCTTCACAAAGAAGAGGAGAAGTGTGGTGAGTATGTGATTGAAGG
CTTTGAACTTAAAATGATTCGATATCAAGAGAGTTTATGTCGGATAGATGAT
>Rchinensis_NC_037090_F_box_plus16_17725765_17727120
ATGGAAACTAGTAGAAGCAAAGTTGTTAAGGTGAGCGATACTTTAACACTAGGTCTGCAGGAAGATGTCCTATTAGAAA
TTCTCTCATATCTGCCTGTCAAGTCTTTACTTAGGTTTCGTTGCGTATCGCAAGAGTGGTATGCTCTCACCAAAAGTCC
TTATTTCATTTCCAAACACCTTGTGCATCGCTCTAGATGCTCTAATCCACATCATCCTTGTCTTCTCTTTCTCTCGCGT
CTAGCTGGAAGTTCTGAAAATAATTTCAAACCACTACTTGGATTCTCATTGCTTCGAGATGAAGAAACAACGTTTTTGA
GATTACCTTTCTGGAACCTTACAAGCCAAAGAAGGGATGGTTTATGGCTTGTGGGATCTAGTAATGGCCTGGTTTGCTG
TGTTCTGTTAATTGGGATTAAGAATGATAAGAATAGAACTACAGAGGGTGAAGAAGATAATGCTACTGTGTTCCAACAA
TTAATAGTAGTATGGAACCCAGCAACAGAACGATTTAGGTTTCTTCCTAAACCTAATAATAATATTACTTTGGATCAGG
ATGAGGAGGAGGATATACTACGTGTAAAGGACAACTCTTTGTCCTGTCCTCTTATAGGTTTTGATTTCGTACATGATCA
TGACATTACTGAATATAAACTGGTCAGAGTTTTCCATCATGATAGTGATGATCCCTACGATCATGGCAAAGTACTTACA
ACTTTTCGAGCTCAAGTCTTCGTCCAAATCACAAATTCTTGGAGACAAGCCAAGAACGAGTTAGGGTTTCCGTCATGTG
AAAATTCTTTTGCATCAACTTCGATTACATTGAATGGAGTGCTGTATTGGAAGGTACAGCGAGCAGGGGACGAGTGCTG
TGTTCTGTCATTCAATTTACGTGAGGAGGTTTTCAATGTGATACAGCTACCAATCGATCTACGAAATCATGATGATTGG
GGCGGACTCCAATTATATTCATGGAGAAATTCACCGGCGACTGTAGCACTTGATTATTCGGCCAATGACGATCTCAACG
CAGCGCTTTGGGTGATGAGTACTGATCAAGAATCAAAGAGGAGTTGTAAGAATGTTGCACAAATACAAAATCCAGCTTG
GACTCGACAGTTCAGATTCAAATACTTAGTAATTTCAGAAGCAACGCGCCTCCTGGGAAGTTGGAAAGATCAATTTCTC
TTTGCATCCGACAAAAGTGATGAAGTTTTTGATGCGGCAGCTGATAATATTGTGCCTCAAGACCTGTTTTCGTATGACC
CTAAAAGCGAGACAAAGAGAAAAATTCTCAAAGATGGAGAGAGGATCAAGTTTTGTGGAGGAGTAAATTATGTGGAGAG
CCTAGTTCCAGTC
>Rchinensis_NC_037090_F_box_plus17_19789495_19790751
ATGCCTTCATCCATGACAATGAGAGCGATGAAAATCAAAACAACCAACACTGAGAGAGATCAAGAAGTAGAAGACAAAA
GAGATGATGAGAAACCCTGCATTATTCTACAATTGCCCGACCACATAATCCTGGAGATCTTCTGTAGAATCCCAATAAA
CACGGTCATCCAATGCAAGTATGTATGCAAGTCTTGGCGCCGTTCTCTCACAGACCAAGAGTTCTCAAAATCCCTATTT

GCACGTACTCCGGCGTGCTTGTTTCTCCAAGACTATAGTAGGACCCCATGCCGGAGGCAAAACACCATACGTCATCGTA
TACCCCCGAATAAGCACATCTTGGCCAACTTGCAAAGCGTCTCGAGCCCAAACAACGTGGTCTTGAAGCTTTCAGATCC
CAATGCCTTATACAACAGGCTCCATTCAAGTGTTACAGGTATTATGGGATCCTGCAATGGCTTCCTCTGCTTATTCCGC
TATAATTGTAAAATTGGTGCTTTCATTTTTAACATATCCAATCCCATTACCGGCGAGTCTGTACCTCTTCCAGTCAATA
AAGAAATAGGCCGTCCGGCTCACTTTGGGTTTGGGTTTAGTCCCATAAGTGATGTCTATAAGGTGGTTGTGTTTACATC
CAATGAAGATAATGAACATCGCTTTCACAAAAGAAAATTGGACGTGATGGTTTTGACTGTTGGCTCTGGGATTTGGAGA
AGAATTGGCAAAGTATCCAATGTCATGCATGGAACACGAGAAGGGGTCTTTCATAATGGATTTCTTCACTGGGTTTGCC
ACTGTAGAAAAGGTTGTCACTTTTTCATTCGTGCATTTGATGTTGAAAGCGAGCGTTTCAAGGATTTACCAATGCCCCC
TTATTATTTCCGTCCCGATTCTATACCTCAAATGCGAGTCTTAGGAGGTTCACTTTCTGTAAGTGATGGCCGCACGTTT
TGGGTAATGAAAGAGTACGGCGTCAAGAGGTCTTGGACCAAGGAGCTTGTAATTGGACATGATACAATTGATCCTAATG
ACGCATATGGGTCCTATATACGATATGTTTCAGTTTTGAAATTTACAGAGGGGAAAGTTTTGTTGTTAGCAAACACTAA
TTTGTGCCTCTATACTCCTGAAACAAGGACCCTTGAGAGGGTTGAAATTGATGGGATGCCACCAGGGGGCTTCCATTTC
GTGGCAGACCATATTCCAAGCTTTGTTTCTCCGAAGGATATTATCAAGGATTACGTCTCCAAAGTACCTAGT
>Rchinensis_NC_037090_F_box_plus18_19818837_19820111
ATGACATCGAGAACAATGAACAAGCAGAAAATCAAGACAACCAACACTGAGAGAGATCAAGAAGTAGAAGACAAAAGAG
ATGATGAGAAACCCTGCATTATTCTACAATTGCCAGACCACATAATCCTGGAGATCTTCTGTAGAATCCCAATAAACAC
GGTCATCCAATGCAAGTATGTGTGCAAGTCTTGGCGTCGTTCTCTCTCAGACCACGAGTTCTCAAAATCCCTATTTGCG
AATTTCAAAAAAAAAGAAAAATCCCTATTTGCACGAACTCCGGCGTGGTTGTTTCTCCAAGACTATAGTAGGACCCACC
GGAGCCGAAACACCGAACGTTATCGTATATCCCCGAATAAGCACATCTTGGCCAACTTGGAAAGCGTCTCGAGCCCAAA
CAACGTGGTCTTGAAGCTTTCAAATCCCAATGCCTTATACAACAGGCTCCATACAAGTTTTACAGGTATTATGGGATCC
TGCAATGGCTTCCTCTGCTTATTCCGCCATAATCGTAAAATTGATGCTTTCATTTTTAACATATCCAATCCCATTACCG
GCGAGTCTGTACCTCTTCCAGTCAATAAAGAAATAGGCCGTCCAGCTCACTTTGGGTTTGGGTATAGTCCCATAAGTGA
TGTCTATAAGGTGGTTGTGTTTACATCCAATGAAGTTAATAAACATCGCTTTTGTTGTCAATTGGACGTGATGGTTTTG
ACTGTTGGCTCTGTGATTTGGAGAAGAATTGGCAAAGTATCCAATGACATGCATGGAACACGAGAAGGGGTCTTTCATA
ATGGATTTCTTCACTGGGTTTGTCGCTGTACGGAGGGTTTTTTCATACGTGCATTTGATGTTGAACGCGAGGGTTTCAA
GGATTTACCAATGCCCCCTTATTATTTCCATCCCGATTCTATACCTCGAATGCGAGTCTTAGGAGGTTCACTTTCTGTA
AGTGATGGCCGCACGCTTTGGGTAATGAAAGAGTACGGCGTCAAGAGGTCTTGGACCAAAGAGCTTGAAATTGGACATG
ATACAATTTATCCTAATGACCCATATGGGTCCTATATACGATGTGTTTCAGTTTTGAAATTTACAGAGGGGAAGGTTTT
GTTGTTAGCAAACACTAATTTGTGCCTCTATACTCCAGAAACAAGGACCCTTGTGAGGGTTGAGATTGATGGGATGCCA
TCAGGGGGCTTCCGTTTCGTGGCAGACCATATTCCGAGCTTTGTTTCTCCGAAGGATATTATCAAGGATTACCTCTCCA
AAGTACCTAGT
>Rchinensis_NC_037090_F_box_plus19_21203124_21204203
ATGGCGGAGTTCATACCCGAAGATGTGATAGTCAAAGTCCTGGAGAGGTTGCCGATCAAATCCTTAATCCGCTTCACCT
GCGTTTCAAAGCGCTGGCGTTTCATCATATTGTCCGACCCACAATTTGCCAAAACCCATTACAAAGTATCTTGTGAGCA
CCAAACCATCCGCCACAGACTCCTCTTCAACAGCCCCGACAAGTTTGGATTTGATTCCCTTGATTCCGAGTCGCCGTTG
TCGTTCGGAGACAATTCATGTGTGAGAAAGGTTAGAATCCCATTTTTGAAACCAGGCGACATTCTGAGTATGCCACGCT
CCTGCAATGGTTTGGTATGTGCAGCAGTTATTGTCTCGTTGTCACCAAAGATTCTGTATCATTGGTATATTTGGAACCC
ATCAACTGGATTCTTTAAAAAATTACCTGATCCTTCCATGAATATTGTGTATCTACAATATTATGGTATTGGCTATTTG
TCAGCCACTGATGACTACAAAATTCTCAAACCCAGGGAGATATTCTCATCGAGAGCTAACGTTTGGAAGACCATTGAGT
TCCCTAATTTGGATGAATTATACTTATCGAGTGAGGGGATTCGTTCAAATGAAGCACTCCATTGGTTCCACGCAGGTAA
TGCGGATATTGTTGCTTTTGATCTGTCAATTGAAGAATTCCGATCAATGCCACTACCTACTACTTTTGAGCACGGGTGT
TTCATACATCTGGCGGCTTCTGTTGGAGGATGCCTGTGTGCGTTTGATTTACAAAACGTTAAGGCTAGTGGCTCTATCT
ATATGTGGGTCATGAGAGAATATGGGGTGGCCGACTCCTGGACTAAGCTCTTCAACTTTAAGGTCTCCAGTCAGCCCGA
GGATATACGTATTTGGGGGCCGATATTCCTTGGGGACACTAGTATAATTTTTGAGTTTCAGATTGCGATTAAAGAGATT
GATGGTGGCAGGATACGTATGAATGATTTCAAGTTGATAAGGAGTGTTCATGAGAACGCAGAGAAGCTTGAAACGTGTG
CCTCGCGAGAGTTCATGATTGCATATGAAGAGACCCTACTTTGGCTAAACGAT
>Rchinensis_NC_037090_F_box_plus20_21569130_21570335
ATGGCAAGGAAAGATGAACTCGAGAAGCGGCGGCAGCGGAACCAACTGGTTCCCTCGGCTTGCGGTGGCTGTGTGCTTA
AACGTCCTCCTTTTGACGACGAGTACTACTACCAGTCGCTAGATACTGGTTCCCTCAAAAACGATATTATCATTGAGAT
ACTGAAAAGGCTACCAGCCAAGTCTTTATTGCGATTTCGTGCTGTATGCAAGTCATGGTGTGCTTTCATCTCTAATTCT
TCTCATTTTAGTAGGAAGCATCTAAGTCACGCAGTCACCGATAGCAGCAGCTTCCGGGTCCTTTATTCATTGATGCCTC
CCCTCTCCGTAGACTGCGAAGCTTTATTGAAGAAAGATGGTCCTGTTCAAGGCAGAAAGCTTAAACTACCCAACCGTTA
CAAACAGCACCGGCGAACGACTGGCAGTTTAAAACGTGAGGTTGCGAATATTGTTGTGGGTTCTTGCAATGGCTTGATA
TGTCTTCTATTAAAATGGGGCTACGTTGTCTTATGGAACCCTTGTACTAGAAAAGTCAACAAATTACCAAAGCAAACTC
ATGTTAAGACACATCAAACGTTTTACGGTTTCGGCTACGATTCCTGCACTGAAAGGTACAAGATCATTCTGGGGGACAT
GCCCTCGATGCCTAAAACATTAGCCCATACGGCAATTTTTACAACATCATGGAGGAATTTTGATGACGTTCAAATTCAT
AACGAAATATATGGTCGTGGATGCTTACTAAACGGAGCTCTACATTGGATAGAGAGTGCCCAATGGGATGCCATCGAGG
ATGACCATAGTGGCCCATCAAGATTGAGTATCATCTCTTTTGATTTGGCAGAGGAGAAATTTCAGAAGCTGGTGTCGTT

ACCCTCTCTTGTAACTCAAGATACGAGCGCAAAAGTTTGGACTTCTAGAGATTCTATCTTTGTATGCTTTTGGAATGGT
ACAGTCCGTACAGAGACAAAATTGACAATATGGGAGATGAAGGAATATGGGATTGAGGATTCCTGGACCAATGTTGGGC
ATATTTCATTCAACAGTCATCCCGAGTGTTATTGGCCGCTGAAATTTTTTGAGGATGGTAACTTTTTGATGACTATATG
CGGGGGCTTGCTAGTATATGATCCGGAGAAGAAGACAATCAGATCTAACAGTGATTTGCCCATCTTGTACATGGAGACT
TTAGTTTCACCATTCACCAGC
>Rchinensis_NC_037090_F_box_plus21_26018972_26020213
ATGCTGAGAACGAAACGAATGAGCCGGTGCAGCAACAATGCAAAGTTGGTGGACCTACCCATTGATATCCTCATCAACA
TCCTTTTGAGACTGCCGGCAAAATCACTTTGCTGCACACAATGTGTCTGTAAAACCCTGCTTCACATAGTTGAAGCTCG
TTCCTTTGCAACACTAGTGCATCTGCGCTTGCTTAATACTGCTTCCGATGCAGTCGCTGAAGTGCCTCAACTTTTGCTT
CTTAATGTTGCCCGAGATGGTGACAATGGAAGCTTAATAACTATCTCTCCATCGCAGATATATGATGGCAATGTCTTGA
CAAAAAGCAGACATGCCATTGTCTCAAAGATTGCATCCTACCGATATGGTTACAATGTTGGTTTTGTTTTCTGCAACTT
GTTCTTCTTTAATGACAAAAGGTATGATCGAGGTCCATGCTTCATTTTCAACCCTCTCAGGGGAGAAGTTCTCATGCTC
CCAAAGAGTACCGTCCAAGTTCCACCTTTTAGAAATATGATATTCATAGATTGGTACGGCATGGGGTTTGATGATATAA
CCAACACCTACAAGATTGTTCGTGTTTCCGGAAATGAACGATGCTGTTTGGTGGCCCAAGTTTATATATTAGGCACCAG
CTCTTGGAAACGGATAAGCTCAGTTCCTCCATGTAATTTAAGCACAAAAAAAATATCTGCATATGGAGACCTGCATTGG
TTAATTAATCGCTCAAGTACTAGAGGACAAATCTGCATAGTTTCTTTTGACTTCGAGAAGGAGGAGTTCTATTTGACGC
CTCATCCCGCATTAAGAAAGCCAGAATATTCTTTCTTGTCCCTTCACTTTGTTAATTTGAGAGGATCTATGGCAATTGT
TGATACTTCATCACATACACATATTGACATATGGGTTTTGAAGAGTTATGAGAAGAAAGAGTGGGTGCGAGATTACAGC
GTGAATCTCGAACTGCCTGGTGTAGAGCGTCCTTGGCATCTTGACCGGTGTGGTACTTGCATCGGTGAGTGGGAGAATG
GCATATTTTTCGTAGAATTTTATGTTACTACTAACGCATTCTTTGTGGATACAAGATGTGATTCTGTGAAACGTGTATC
ACTTGGAGGTTCAGAGTATACGAGGATCTTCAGCTATCCTGGGAGTTTGATTTCATTAAAAGATTATGGCAATTTGATT
AGAGCAGAAGCAGGTAACTGGAGGCCTGATTTCTCTGAAAAGTCTTGGCAAATTGAT
>Rchinensis_NC_037090_F_box_plus22_28383240_28384418
ATGTCAGACTACTTTCCTGAGGAAATCATACAAAGAATCCTGTTGAGGTTGCCTACGAAATCCCTAATCAAATCAACCC
TAGTCTGCAAGTCATGGATGTCACTCATCAAATCCTCTACCTTCATTCAATCCCATCTCTCCACCACAATCGACACCAA
CAACCAAAACGATGCCCACCTCCTCCTCCTCAGCGCTTTCTCTTACAAGGTTTCTGATCGTCTGCACTGGTTGCATTGG
GATAGCCCTGAATTCGGTGAGTATTCCTTGCTTGCAGACCCAGTCACTTTCTTGGAAAACGAAACTATTTCCGATCTGA
AGGTAGTCGGAACTTGTAACGGGCTCATATGCCTTGCGCCTCACCTATCTGATAACCATTCCCCCACTTTAATTTGGAA
CCCATGTATTAGAAAGATTGTGATTTTGCCTAGCCCACCTGCTTGTTTTACCTCTACTGAGTCTAAGTATGATGTGTGC
ATAGAGCATGGCTTTGGCTATGATTTACATTCCAATGACTATAAGGTGTTGAGAATTGTGACTCTTCCTGATGATCATT
TGGACTTGTCCCGATTCGCCACCGTGGTTCAGGTTTACTCATTAGCCAGGGGCTCCTGGAAGACGCTTGATGCTTCTGT
TGTTCCTGTAGATTTGCAGGGGGGTGGTTCTGGTATTGTATTTCTCAATGGTTCTCTGCATATGCTTGAAGTTCGTTTA
AGTGTCGAATATGATGATCATACGTATGAATATTGTAAGTATGGTGGAGATACTTTCATTGCTTCATTTGATATGGCCA
CCGAATTATTTGGCGAGGTGATGATGCCAGAAGCTTTACAGAGCAACAGATGTACGATTTCAAAATATGGGGACTCCCT
TGCCTTGATTAAGCATTATGATTATAACTGTGGTTGTGACATATGGGTAATGAAAGAGTATGGTGTCACAGAGTCGTGG
ACATATTTGTACAAGATAGCTGGGTCTCAAGATCGTATATATGGTTTCAAAAGGTGTGGGGAAGTTGTTCTGAGTGAGG
AAAATAATCTTGGTTGGTTAAGAATGATTTCGCTGGATCCTAAGAGCAAACAAGTTCAAGTTTTCGGAAATAAGGATTA
TATTTACTACTTCATGGATTCTTTTGTAGAGGGTCTCATCTTGCTTGACCACGTCAATGCCATTTCGTACCAA
>Rchinensis_NC_037090_F_box_plus23_28397189_28398376
ATGTCAGACTACTTTCCTGAGGAAATCATAGAAAGAATCCTGTTGAGGCTGCCTACCAAATCCCTAATCAAATCCACCC
TAGTGTGCAAGTCATGGATGTCACTCATCAAATCCTCTACCTTCATTCAATCCCATCTCTCCATCACAATCGACTCCAA
CAACCAAAACGACGCCCACCTCCTCCTCCTCAGCGCTTTCTCTTACAAGGTTTCTGATCGTCTGCACTGGTTGCATTGG
GATAGCCCTGAATTCGGTGAGTATTCCTTGCTTGCAGACCCAGTCACTTTCTTGGAAAACGAACCTATTTCCGATCTGG
AGGTAGTCGGAACTTGTAACGGGCTCATATGCCTTGCGCCTGACCTATCTGATAACCATTCCCCCACTTTAATTTGGAA
CCCATGTATTAGAAAGATTGTGATTTTGCCTAGCCCACCTGCTTGTTTTACCTCTACTCAGTATGATAAGTACATAGAG
CATGGCTTTGGCTATGATTCACATTCCAATGACTATAAGGTGTTGAGAATTGTGACTCTTCCTGATGATCATTCGGACT
TGTCCCGATTCGCCACTGTGGTTCAGGTTTACTCATTAGCCAGGGCCTCCTGGAAGACTCTTGATGCCTCTGTTGTTCC
TGTAGATTTGCAGGGTGGTCCTGGTATTGTACTATTTCTCAATGGTTCTCTGCATATGCTTGAAGTTCGTTTTAGTGTC
GGATATGATGATCATGCGGATGAATATTGTAAGTATGGTGGAGATACTTTCATTGCTTCATTTGATATGGCCACCGAAT
TATTTGGCGAGGTGATGATGCCAGAAGCTTTGCAGAGGAACAGATGTACCATTTCAAAATATGGGGACTCCCTTGCCTT
GATTAAGCATTATGATTATAACTGTGGTTGTGACATATGGGTAATGAAAGAGTATGGTGTCACAGAATCGTGGACATAT
TTGTACAAGATAGCTGGGGATCATGATTATATATATGGTTTCAAAAGGTGTGGGGAAGTTGTTCTGAGTGAGTTCAATA
ATCATGGTCGGTCAAGAATGATTTCGCTGGATCCTAAGACCAAACAAGTTCAAGTTTTCGGAAATAAGGATTATACTTA
CTACTTCATGGATTCTTTTGTAGAGAGTCTCATCTTGCTTGACCACGTCAATGCCATTTCGTACCAACGAGCAAGGAAG
AAG
>Rchinensis_NC_037090_F_box_plus24_36186560_36187792
ATGGTTCCCCTCAAGCAATATAGAAGAAGAAGAAGACCCCCCACTGAGATAAAAGACTTAACCCCTGACATATTGTTTG
AAATCTTTGCAAGAGTGCCCATGAAATATCTTTTGCAGTTAACAACAGTTTGCAAATCTTACAAGGCTTTAATTAGGAG

CCATGAGTTCAGAAGATTCCATCACGAAAAGAATACCATGAAAATTGCTCCTGATTATCTACTTGTTCGCAATAGAATT
GGAGGTCAACAAAGGGGCTTCTCCATTTATTCTGCTAGAACATTTGCCTATGATGAGAATTCAGGGCTTCCTAGATCCA
TAGATATTCCCATGCAGATGCAGCTCCATGACCTTCCTCTGTACGAATATGGTTTCACTGTTTATGGTTCTTGCAATGG
ATTGCTTTGCATCTCTTTATTCTCTCTGGACTTGGACTCTCCTTTATATCTGTATAATCCATCACTCAGAAAATTCAAG
CAACTTCCTCGAAGTGAATTTGAGCTTCCTCAAGGCACCTCCATTACTTCTCAGATGGCCACAAGTTTAGTTACTCTCG
GCTTTGGGTTCCATTCTGGGATGGATGACTATCAGGTTGTGAGGTTTGTACACCCCAATACAAGTATCTTTTATGCTGA
GGTTTACAGTCTCAAGTTGAATTCTTGGACACCAGTAGAGAATGTTGTAGATCCCCGTGAGGTTCATGCTTGGTTTATG
GAAGGATGTACATGCTTGGATGGAGTTATATATTGGCATTTAATGCAGCGTCCTAACATGTCCCTCGTTTCTTTTAATA
TGCACACTTCAGTTCTTGGAAAGAGGACGCTTCCTTATCAGTTGCAACCGGTGATGCGTCCAATTTGTTTGCAAGTGTT
GCATCCAGAGTGTTTGCGAGCGCTGCATGAAAATAGTGTGCAAGTGTCGAAGAAATCACTCTGTTTGTTCCAAGTAATA
CAGCGAGGCTGCCATCACTACTGTGACATATGGGTTCTGAAAGAGGATACTGAGGAAAAAATTGGCAGGATTCGTCTTC
CACCACATGCATCTATAGCATGGCCATTGGGATTTAGCACAAATGGCAATGTTGCTTATATGGTTATCACTGAGGATCA
AGACTTACCCCTGTTGGTTCAATATGTTCCTCTGCTGAACCGCATTCAGACTGTTATAACTCAAATAAGTCACAGACCC
TGGTACGTTGATGCTTATAGAGAGAGTCTGGTTCTACTCGATGAAATC
>Rchinensis_NC_037090_F_box_plus25_38266966_38268198
ATGGCAGAAGAGGTGGTGGTTCAAATCCTATCGAGGCTACCTCCCAAATCTTTGATGCGATTTAAATGCGTCCGTAAGT
CATGGTGTGCACTGGTCAATAATCCAGGATTCATATTCCAACAACTCCACTTGTACAACAAATTTTCCTCCACTCCCAT
TCTTATCAAGCGTACGGTCATTAGCCGGACTGAAAGTACCAGCGAGGAAGTTGTGTTTTCCTTCCTCAATCTTCATAAT
GATGATTATAACTGTCATCTTGAGGCTAACTGTCATTCTATTGTTGAGGACATCAATTTTCCAGCTTCTATGGGTCTAA
AGACGAGGGGCCAATTTATTGAGCTTCCTATGCCTAGTTCTAGTTCGTTTGATGAAGATGTATATGTTATAGGTCATTG
TGATGGGATCATTTGTCTTTTAGTTATATATATTAGCACTAGCGTTATCATATGCAATCCAGCAATCAAGGAGTATAGG
CTTGTTAAAGATGTAGATGTAATCTCATTTCATGTCGGATTTGGCTATCATCCCAAATCCAAAGATTACATACTTATTA
ATGTCATGTCGACTGGAGAGAAAGTATATGATAACGAGCGTCTCGTTATTCATCCTCCTAGGGCAAAATTATACACTTT
GGGAACTGGTTCTTGGAGAGAGATCAATACAAATTATTTGGAAACAGAAACTACCCACTTTTGGCCTAGTCTTTTCCAG
TTCTACTTTAAAGGAATTTTCTATTGGTTGGGAAATGAGCAAAGGAAGGAACTCATCTCTGAGTTTGACAGGGATGATG
AGGATAACAATAGACTAGTAATCCTTTTCTATGATGCAGGTGATGAGCTCTTTCATAGTTTATTGCTTCCAGATGGTTT
CTATGACCCAAGTGAGGGTCTTTATGGTATGCGTCTTGCATTGTTGAATGAATCCATTGCTCTTTATGGCTTTCACTGC
ATTGGTAACTGTCCTTCACCATTCGAAATTTGGATAATGGTCGACTTTGATGGTACCAACTGTTCTTGGACAAAACAGT
TGGCAGTTGTGCCCACGGTGGAACTTTATATGCCAGGGGCACTTTGGAAGAACAATGTTATTCTAATTAACAGAGAAGG
GCGAGTAGTTTCCTACAATTTTGATAAGGAGAAACTTAAATATAACCCGATACATGGTGTGCTTCGAGCAAGTTTTCAA
GCTACAATTTGTGTACATAGTATAGTTTCAGTCAAGGGACAACCAACC
>Rchinensis_NC_037090_F_box_plus26_43723185_43724546
ATGGCTATATACTTCATCGATGACTCTTTTTCTATCTCACGAGGTCTATTTGAAAAACGATTCCTGCCTTCATCCATGA
CATCGAGAACAATGAACAAGCAGAAAATCAAAACAACCAACACTGTGAGAGATCAAGAAGTAGAAGACGAAAGAGATGA
TGAGAAACCCTGCATTATTCTACAATTGCCAGACCACATAATCCTGGAGATCTTCTGTAGAATCCCCATAAACACGGTC
ATCCAATGCAAGTATGTGTGCAACTGTTGGCGTCGTTCTCTCTCAGACCACGAGTTCTCAAAATCCCTATTTGCGAATT
TCAAAAAAAAAGAAAAATCCCTATTTGCCCGAACTCCGGCGTGGTTGTTTCTCCAAGACTATAGTAGGACCCACCGGAG
CCGAAACACCAAACGTTATCGTATATCTCCGAATAAGCACATCTTGGCCAACTTGGAAAGCGTCTCGAGCCCAAACAAC
GTGGTCTTGAAGCTTTCAAATCCCAATGCCTTATACAACAGGCTCCATACAAGTGTTACAGGTATTATGGGATCCTGCA
ATGGCTTCCTCTGCTTATTCCGCCATAATGGTAAAATTGATGCTTTCATTTTTAACATATCCAATCCCATTACCGGCGA
GTCTGTACCTCTTCCAGTCAATAAAGAAATAGGTCGTCCAGCTCACTTTGGGTTTGGATTTAGTCCCATAAGTGATGTC
TATAAGGTGGTTGTGTTTACATCCAATGAAGTTAATGAACATCGCTTTCACAAAAGAAAATTGAACGTGATGGTTCTGA
CTGTTGGCTCTGGGATTTGGAGAAGAATTGGCAAAGTATCCAATGTCATGCTTGGAACACGACAAGGAGTCTTTCATAA
TGGATTTCTTCACTGGGTTTGCCACTATAGAAAAGGTTGTCATCACTTTTTCATACGTGCATTTGATGTTGAAAGCGAG
GGTTTCAAGGATTTACCAATGCCCCCTTATTATTTCCGTCCCGATTTTATGCCTCAAATGCGAGTCTTAGGAGGTTCAC
TTTCTGTAAGTGATGGCCGCACGTTTTGGGTAATGAAAGAGTACGGCGTCAAGAGGTCTTGGACCAAAGAGCTTGAAAT
TGGACATGATACAATTTATCCTAATGACCTATATGGGTCCTATATACCATATGTTTCAGTTTTGAAATTTACAGAGGGG
AAGGTTTTGTTGTTAGCAAAAACTAAATTGTGCCTCTATACTCCTGAAACAAGGACCCTTGTGAGGGTTGAGATTGATG
GGATGCCATCAGGGGGCTTCCATTTCGTGGCAGACCATATTCCGAGCTTTGTTTCTCCGAAGGAAATTATCAAGGATTA
CCTCTCCAAAGTACCTAGT
>M_domestica_S1_SFBB13_SFBB1_MG458583
AAATGCATACGCAAGTCTTGGTGCACTCTCATCAATACTCCAAGTTTTGTTGCCAAACACCTCAACAATTCTATGAACA
ACAAACTATCGTCCTCCACTTGCATCCTTCTCAACCGTTGTCAGAATCATGTTTTCCCGGATAGAAGTTGGAAACCAGA
AGTTTTCTGGTCCATAATTAATCTTTCCATTGATAGCGATGATCACAACCTTCATTATGATGTTGAGGACCTCAATATA
CCGTGTCCATTGGAAGGTCATGATTTTGTAGAGATTGGTGGCTATTGCAATGGGATTGTCTGTGTACTAGCATGGAAAA
CTCTTCATTGGATATATGTTATTTTATGCAATCCTGCAACTGGGGAATTTAGGCAACTTCCCCATTCATGCCTTCTTCA
ACCTTCCCGTTCTAGGAGAAAATTTGAATTGAACACGATCTCTACATTATTGGGATTTGGTTATGATTGCAAAGCTAAA
GAATACAAGGTCGTGCAAGTTATTGAAAATTGTGAGTATTCAGATGCTGaGCAATATGATTATCATCGTATTGCTCTTC

CTCACACGGCTGAGGTATATACCACGACTGCTAACTCTTGGAGAGAGATCAAGATTGATATATCAAGTGAAACCTATTG
TTATACTTGTTCAGTGTACTTGAATGGATTTTGTTATTGGATTGCAACCGATGAAGAAGATTTCATACTTTCATTTGAT
TTAGGTGATGAGATATTTCATAGAATACAATTGCCTTCTAGGAGAGACTCTGATTTTAAGTTTTCTAATCTCTTTCTGT
GTAATAAATCGATTGCTTCTTTTGGTTATTGTTGCAATCCAAGTGATGAGGATTCTACATTatat
>M_domestica_S1_SFBB2_MG458453
ATGACTAAGGTACGTGAAAGTGAAACTCCTGAAGATAGGGTGGCCGAAATCTTGTCCAGGTTGCCTCCGAAGTCTCTGA
TGCGTTTCAAATGTATAAGCAAGTCTTGGTGCACGGTCATCAACAATCCAAGTTTTATGGCCAAACACCTCAGCAATTC
CGTTAACAACAAATTCTCATCCTCCACTTGTATCCTTCTCCACCGTTCTCAGATGCCCGTTTTCCCGGACAGAAGTTGG
AAACGAGAATATTTCTGGTCCATGATTAATCTTTCCCATGATAGTGATGAACACAACCTTTATTATGATGTTGAGGACC
TAAATATACAATTTCCATTGGAAGATCATGATCATGTATCGATTCATGGCTATTGCAATGGGGTTGTCTGTCTAATAGT
AGGGAAAAATGCTGTTTTATACAATCCTGCAACGAGGGAACTGAAGCAACTGCCTGATTCATGCCTTCTTCTACCTTCC
CCTCCGGAGGGAAAATTCGAATTGGAATCGACCTTTCAAGGAATGGGATTTGGATATGATAGCAAAGCTAAAGAATACA
AGGTTGTGAAAATTATAGAAAATTGTGAGTATTCAGATGATATGCGAACATTTTCTCATCGTATTGCTCTTCCTCACAC
GGCTGAGGTATATATCACGACTACTAACTCTTGGAGAGTGATCGAGATTGAAATATCAAGTGATACCTATAATTGTTCT
TGTTCAGTATACTTGAAGGGATTTTGTTATTGGTTTGCAAGCGATGACGAGGAATACATACTTTCATTTGATTTAGGTA
ATGAGATATTTCATAGAATACAATTGCCTTATAGGAAAGAATCCGGTTTTTTGTTTTATGATCTTTTTCTGTATAATGA
ATCCATCGCTTCTTTTTGCTCTCTTTATGATAAAAGTGACAATTCTGGAATATTGGAAATACTT
>M_domestica_S1_SFBB3_MG458465
ATACGCAAGTCTTGGTGCACTCTCATCAATAGTCCAAGTTTTGTGGCCAAACACCTCAGCAATTCCTTGGACAACAAAC
TCTCATCCTCCACTTGTATCCTTCTCAACCGTTCTCAGTTTCACATTTTCCCGGATCAGAGTTGGAAACGTGAAGTTTT
ATGGTCCATGATTAATCTTTCCAGTGATAGTGATGTGCACAACCTTCATTATGATGTTAAGCCCTTAAATATACCGTTT
TCTAGGGATGACCATAATCATGTACAGATTCACGGGTATTGCAATGGGATTGTATGTCTAATAGAAGGGGATAATGTTC
TTCTATGCAATCCTTCAACGAGGGAATTCAGGCTACTTCCCAATTCATGCCTTCTTGTACCCCATCCCGAGGGAAAATT
CGAATTGGAAACAACCTTTCACGGAATGGGTTTTGGCTATGATTGCAAAGCTAATGAATACAAGGTTGTGCAAATTGTA
GAAAATTGTGAGTATTCGGATGATGAGCAAACATATCAACATTGTATTGCTTATCCTTACACGGCTGAGGTATACACCA
CGGCTGCTAACTTTTGGAAAGAGATCAAGATTGATATATCAAGTTCAACCCATCCCTATCCCTATTCTGTGTACTTGAA
GGGATTTTGTTATTGGTTTGCAACGGATGGCGAAGAATGCATACTTTCATTTGATTTAGGTGACGAGATATTTCATAGA
ATACAATTGCCTTCTAAGATAGAATCCGGTTTTAACTTTTGTGGTCTTTTTCTTTATAATGAATCTATCACTTCTTATT
GTTGTCGTTATGATCCAAGTGAGGATTCTAAATTATTTGAAATATGGGTAATGGATGACTATGATGGAGTTAAGAGTTC
ATGGACAAAACTCCTAACTGTTGGACCCTTTAAAGGCATTGAGTATCCATTGACACTTTGGAAATGTGACGAGCTTCTT
ATGCTTGCATCCGATGGAAGAGCCACCTCTTAT
>M_domestica_S2_SFBB3_MG458468
ATGTCCCAAGTGCGTGAAAGTGAAACTCTTGAAGACAGGGTGGTCGAAATCTTGTCTAGGTTGTCGCCCAAGTCTCTGT
TGCGATTCAAATGCATACGCAAGTCTTGGTGCACTCTCATCATTAGTACAAGTTTTGTTGCCAAACACCTCAGCAATTC
CTTGGACTACAAACACTCATCCTCCACTTGTATCCTTCTTAACCGTTCTCAGTTTCACATTTTCCCAGATCAGAGTTGG
AAACATGAAGTTTTATGGTCCATGATTAATCTTTCCATTGATAGTGATGTACATAACCTTCATTATGATGTTAAGTCCC
TAAATATACCGTTTCCTAGGGATGACCATAATCATGTACACATTCACGGTTATTGCAATGGGATTGTATGTCTAATAGA
AGGGGATAATGTTCTTCTATGCAATCCTTCAACGAGGGAATTCAGGCTACTTCCCGATTCATGTCTTCTTGTACCCCAT
CCTGAGGGAAAATTCGAATTGGAAACGACCTTTCACGGAATAGGTTTTGGCTATGATTGCAAAGCTAAAGAATACAAGG
TTCTGCAAATTATAGAAAATTGTGTGTATTCAGATGATGAGCAAACATATCAACATTGTATTGCTTTTCCTTACACGGC
CGAGGTATACACCACGGCTGCTAACTTTTGGAAAGAGATCAAGATTGATATATCAAGTACAACCCATCCCTATCCTTTT
TCTGTGTACTTGAAGGGATTTTGTTATTGGTTTGCAAGGGATGGCGAGGAATGCATACTTTCATTTGATTTAGGTGATG
AGATATTTCATAGAATACAATTGCCTTCTACGATAGAATCTGGTTTTAAGTTTTGTGGTATTTTTCTTTATAATGAATC
TATCATTTCTTATCGTTGTCGTTATGATCCAAGTGAGGATTCTAACTTATTCGAAATGTGGGTAATGGATGGGTATGAA
GGAGTTAAGAGTTCATGGACAAAACTCCTAACCGTTGGTCCCTCTAAAGGCATTGAGTATCCATTGACACTTTGGAAAT
GCGACGAGCTTCTTATGGTTGCCTCCGGTAGAAGAGTCACCTCTTATAATTCTAGTACCGAAAATCTCAAGGATCTTCA
TATTCCTCCAATTATGCATCAGGTTACAGGTTTGCAAGCTCTTATTTATGAGGAAAGTCTTGTTCCAATTAAG
>M_domestica_S1_SFBB4_MG458475
ATGTCCCAAGTGCGTGAAACTGAAACTCCTGAAGATAGGGTGGTCGCCATCATGTCCAAGTTGCCGCCCAAGTCTCTGA
TGCGATTCAAATGCATACGCAAGTCTTGGTGCACTCTCATCAATAATCCAAGTTTTGTGGCCAAACACCTCAGCAATTC
TGTGGACAACAATTTCTCATCCTATACTTGTATCCTCCTCAACCGTTCTCAGGTTCACGTTTTCCCGGACAAGAGTTGG
AAACATGAAGTTTTATGGTCCATGATTAATTTTTTTAATGATAGAGTTTCACGCACCCTTTATTATAATGTTGAGGACC
TAAATATACCGTTTCCAAGGGATGACCATGAACATATACTGATTCATGGTTATTGCAATGGAATTGTTTGTGTAATATC
AGGGAAAAATATTCTTTTATGCAATCCTGCAACGAGGGAATTCAGGCAACTTCCTGATTCATTCCTTCTCCTACCTTCC
CCTCTCGGCGGAAAATTCGAATTGGAGACCGACTTTGGAGGATTGGGATTTGGCTATGATTGCAGAGCTAAAGATTACA
AGGTTGTGCGAATTATAGAAAATTGTGAGTATTCAGATGATGAGCGAACATATTATCATCGTATTCCTCTGCCTCACAC
GGCTGAGGTATACACCATGGCTACTAACTCTTGGAAAGAGATCAAGATTGATATATCAAGTAAAACTTATCCCTGTTCT
TGTTCAGTGTACTTGAAGGGATTTTGTTATTGGTTTACAAGGGATGGTGAGGAATTCATACTTTCATTTAATTTAGGCG

ATGAGAGATTTCATAGAATACAATTGCCTTCTAGGAGAGAATCCGGTTTTGAGTTTTATTATATTTTTGTGTGTAATGA
ATCCATTGCTTCTTTTTGCTCTCTTTATGATCGAAGTCAAGATTCTAAATCATGTGAAATATGGGTAATGGACGATGAT
GGAGTCAAGAGTTCATGGACAAAACTCCTAGTCGCTGGACCCTTTAAAGGCATTGAGAAGCCATTGACACTTTGGAAAT
GTGATGAGCTTCTTATGATTGACACCGATGGAAGAGTCATCTCTTATAATTCTGGTATTGGATATCTCACCTATCTTCA
TATTCCTCCGATTATCAATAGGGTTATAGATTCCCAAGCTCTTATTTATGTAGAAAGTATTGTTCCAGTCAAG
>M_domestica_S1_SFBB5_MG458492
ATGTCCCAGGTCCGTGAAATTGAAACTCTTGAAGATAAGCTGGTCGAAATTCTATCTAGGTTACCGCCCAAGTCCTTGA
TGAGATTCAAATGCATACACAGGTCTTGGTGCGCTATCATAAGTAGTCCAAGTTTTGTGGCCAAACACCTCAGCAATTC
CATGGACAACAAACTCTCATCCTCCACTTGTATCCTTCTCAACCGTTGTCAGGTTCACGTTTTCCAGGATAGGAGTTGG
AAACAAGACGTTTTCTGGTCCATGATTAATCTTTCCATTGATAGTGATGAGAGAAATCTTCATTATGATGTTGAGGACC
TAAATATACCGTTTCCAATGGAAGATCAAGACAATGTAGAGCTTCACGGTTATTGCAATGGGATTGTCTCTGTAAAAGT
AGGGAAAAATGTTCTTTTATGCAATCCTGCAACAGGAGAATTCAGGCAACTTCCTAATTCATCCCTTCTTCTACCCCTT
CCCAAGGGAAGATTCGGATTGGAAACCATCTTTAAAGGATTGGGATTTGGCTATGATTGCAAAACTAAAGCGTACAAGG
TTGTGCAAATTATAGAAAATTGTGATTGTGAGTATTCAGAAGGTGAAGAATCATATTATGAGCGTATTCTTCTTCCTTA
CACGGCTGAGGTATACACCATGACTGCTAACTCTTGGAGAGAGATCAAGATTGATACATCAAGTGATACTGATCCGTAT
TGCATTCCCTATTCTGGTTCAGTGTACTTGAAGGGATTTTGTTATTGGTTTGCAAACGATAATGGGGAATACGTATTTT
CATTTGATTTGTGTGATGAGATATTTCATAGAATAGAATTGCCTTCTAGGGGACAATTCGATTTTAAGTTTTATGGTAT
TTTTCTGTATAATGAATCCATCGCTTCTTATTGCTCTCGTTACGAAGAGGATTGTAAATTATTTGAAATATGGGTAATG
GATGATTATGACGGAGTTAAGAGTTCATGGACTAAACTGCTAACTGTTGGACCCTTTAAAGACATTGATTATCCATTGA
CATTTGGGAAATGTGATGAGGTTCTTATGCTTGGCTCGTATGGAAGAGCGGCCTCTTGTAATTCTAGTACCGGAAATCT
CAAGTATTTTCATATTCCTCCCATTATCAATTGGATGATCGATTATGTGAAAAGTATTGTTCCAATCAAG
>M_domestica_S1_SFBB6_MG458504
ATGTCCCAGGTGCATGAAAGTGAAACTCCTGAAGATAAGGTGGTCGAAATCTTGTCAAGGTTGTCGCCCAAGTCCCTGA
TGAGATTCAAATGCGTACACAAATCATGGTGCACTATCATCAATAGTCCAAGTTTTGTGGCCAAACACCTCAGCAATAC
CGTGGACAACAAATTCTCATCCTTCACTCGCATCCTTTTCAACCGATGTCAGGTTCATGTCTTCCCGGACAGGAGTTGG
AAAAGAGATGTTTTCTGGTCTATGATTAATCTTTCCATTGATAGTGATGAGCACAACCTTCGTTATGATGTCGAGGACC
GAAATATACCCTTTCCTATAGAAGTTCAAGACAATGTACAGCTTTACGGTTATTGCAATGGGATTGTCTGTGTAATAGT
AGGGGAAAATGTTCTTCTATGCAATCCTGCAACAAGAGAATTCAAGCAACTTCCCGATTCATCCCTTCTTCTACCCCTT
CCCATGGGAAAATTCGGATTGGAAACCCTCTTTAAAGGATTGGGATTTGGCTACGATTGCAAAACTAAAGAATATAAGG
TTGTGCGAATTATAGAAAATTGTGATTGTGAGTATTCAGATGGTAAAGAATCATATATTGAGCGTATTCTTCTTCCTTA
CACGGCTGAGGTATACACCACGGCTGCTAACTCTTGGAAAGAGATCAAGATTGATACATCAAGTGATACTGATCCCTAT
TGCATTCCCTATTCTTGTTCAATGTACTTGAAGGGATTTTGTTATTGGTTTGCAAACGATAACGGGGAATACATATTTT
CATTTGATTTAGGTGATGAGATATTTCATAGAATAGAATTGCCTTTTCGGAGAGAATCCGATTTTAAGTTTTGTGGTCT
TTTTCTGTATAATGAATCCGTCGCTTCTTATTGCTCTTGTTACGAAGAGGATTGTAAATTGGTTGAAATATGGGTAATG
GATGATTATGATGGAGTGAAGAGTTCATGGACAAAACTTCTAACCGTTGGACCCTTTAAAGACATTGAGTCTCCTTTGA
AATTTTGGAAATGTGACGAGGTTCTTAGCCTTTCCTCGTATGGAAAAGCCACCTCTTATAATTCTAGTACCGGAAATCT
CAAGTATTTTCATATTCCTCCTATTATCAATTGGATGATAGATTATGTGGAAACTATTTTTCCTGTCAAG
>M_domestica_S1_SFBB7_MG458517
ATGTCCCAGGTGCGTGAAATTGAAATTCCTGAAGATAAGGTGGTCGAAATCCTGTCCAAGTTGCCGCCCAAGTCTCTGA
TGAGATTCAAATGCATACGAAAGTCTTTGTGCACTATCATCAATAGTCCAAGTTTTGTGGCCAAACACCTCAACAATTC
CATGGACAACAAACTCTCATCCACCACTTGTATCCTTCTCAACCGTTGTCAGGTTCACATTTTCCCGGACAGGAGTTGG
AAACAAGACGTTTTCTGGTCCATGATTAATCTTTCCTTTGATAGTGATGAGCACAACCTTCATTATGATGTTGAGGACC
TAAATATACCGTTTCCAATAGAAGATCAAGACAATGTAGAGCTTCATGGTTATTGCAATGGGATTGTCTGTGTAATAGC
AGGGAAAAATGTTCTTTTATGCAATCCTGCAACGAGAGAATTCAAACAACTTCCCAATTCATCTCTTCTTCTACCCCTT
CCCAAGGGAAGATTTGGATTGGAAACGACCTTTAAAGGAATGGGATTTGGCTATGATTGCAAAACTAAAGAATACAAGG
TTGTGCGAATTATAGAAAATTGTGATTGTGAGTATTCAGATGATGGAGAATCATACTATGAGCGTATTCTTCTTCCTCA
CACGGCTGAGGTATACACCACGACTGCTAACTCTTGGAAAGAGATCAAGATTGATATATCAATTGAAACTGGTTGGTAT
TGTATTCCCTATTCTAGTTCAGTGTACTTGAAGGGATTTTGTTATTGGTTTGCATACGATAACGGGGAGTACGTATTTT
CATTTGATTTAGGTGATGAGATATTTCATAGAATAGAATTGCCTTCTAGGAGAGAATCCGATTTTAATTTTTATGGTAT
TTTTCTATATAATGAATCCATCACTTCGTATTGCTATCGTCACGAAGAGGATTGTGAATTATTTGAAATATGG
>M_domestica_S10_SFBB7_SFBB21_MG458525
ATGTCCCCTGAAAGTGAAACTCCTGAAGATAAGATGGTCGAAATCTTGTCCAAGTTGCCGCCCAAGTCTCTGATGAGAT
TCAAATGCATACGCAAATCTTGGTGCACTATCATCAATAGTCCAAGTTTTGTGGCCAAACACCTCAGCAATTCCATGGA
CAACAAACTCTCATCCACCACTTGTATCCTTCTCAACCGTTGTCAGGTTCACGTTTTCCCGGACAGGAGTTGGAAACAA
GACGTTTTCTGGTCCATGATTAATCTTTCCATTGATAGTGATGAGCACAACCTTCATTATGATGTTGAGAACCTAAAGA
TACCGTTTCCAATGGAAGATCAAGACAATGTAGAGCTTCACGGTTATTGCAATGGGATTGTCTGTGTAATAGCAGGGAA
AAATGTTCTTTTATGCAATCCTGCAACAGGAGAATTCAGGCAACTTCCCAATTCATCTATTCTTCTACCCCTTCCCAAG
GGAAGATTCGGATTGGAAACGACCTTTAAAGGAATGGGATTTGGCTATGATTGCAAAACTAAAGAATACAAGGTTGTGC

GAATTATAGAAAATTGTGATTGTGAGTATTCAGAGGATGGAGAAACATACAATGAGCGTATTCTTCTTCCTCACACGGC
TGAGGTATACACCACGACTGCTAACTCTTGGAAAGAGATCAAGATTGATATATCAATTGAAACTCGTTGGTATTGCATT
CCCTATTCTGGTTCAGTGTACTTGAAGGGATTTTGTTATTGGTTTGCATACGATAACGGGGAGTACGTATTTTCATTTG
ATTTAGGTGATGAGATATTTCATAGAATAGAATTGCCTTCTAGGAGAGAATCCGATTTCAAGTTTTATGGTATTTTTCT
ATATAATGAATCCGTCACTTCGTATTGCTATCGTCACGAAGAGGGATGTCAATTATTTGAAATATGGGTAATGGACGAA
TATGATGGAGTTAAGAGTTTATGGACAAAACTGCTAACCATTGGACCCCTTAAAGACATTGATTATCCATTGACACTTT
GGAAATGTGACGAGATTCTTATGCTTGGCTCATATGGAAGAGCTGCCTCTTGTAATTCTAGTAGTGGAAATCTCAAGTA
TCTTCATATTCCTCCTATTATCGAATGGATGGTGGATTATGTGAAAAGTATTGTTCCAGTCAAGTGCATTGAGGGAAAA
GTTCCATTTTCTCCTATT
>M_domestica_S10_2_SFBB7_SFBB21_MG458526
CCGCCCAAGTCTCTGATGAGATTCAAATGCATACGCAAGTCTTGGTGCACTATCATCAATAGTCCAAGCTTTGTGGCCA
AACACCTCAGCAATTCCATGGACAACAAACTCTCATCCACCGCTTGTATCCTTCTCAACCGTTGTCAGGTTCATGTTTT
CCCGGACAGGAGTTGGAAACAAGACGTTTTCTGGTCCATGATTAATTTTTCCATTGATAGTGATGAGAATAACTTTCAT
TGTGATGTTGAGGACCTAAATATACCGTTTCCAAGGGAAGATCAAGACAATGTAGAGCTTCACGGTTATTGCAACGGGA
TTGTCTGTGTAATAGTGGGGAAAAATGTTCTTTTATGCAATCCTGCAACGGCAGAATTCAGGCAACTTCCCGATTCATC
TCTTCTTCTACCCCTTCCCAAGGGAAGATTCGGATTGGAAACGACCTTTAAAGGAATGGGATTTGGCTATGATTGCAAA
ACTAAAGAATACAAGGTTGTGCGAATTATAGAAAATTGTGATTGTGAGTATTCAGAAGATGGAGAATCATACTATGAGC
GTATTCTTCTTCCTCACACGGCTGAGGTATACACCGCGACTGCTAACTCTTGGAAGGAGATCAAGATTGATATATCAAT
TGAAACTCGTTGGTATTGCATTCCCTATTCTGGTTCAGTTTACTTGAAGGGATTTTGTTATTGGTTTGCATACGATAAT
GGGGAGTACGTATTTTCATTTGATTTAGGTGATGAGATATTTCATAGAATAGAATTGCCCTCGAGGAGAGAATCCGATT
TCAAGTTTTATGGTATTTTTCTGTATAATGAATCCGTCACTTCGTATTGCTATCGTCATGAAGAGGATTGTGAATTATT
TGAAATATGGGTAATGGACGACTATGATGGAGTTAAGAGTTCATGGACAAAACTGCTAACCATTGGACCCCTTAAGGAC
ATTGATTATCCATTGACACTTTGGAAATGTGACGAGATTCTTATGCTTGGCTCATAT
>M_domestica_S1_SFBB8_MG458530
ATGTCCCAGGTGCGTGAAAGTGAAACTCCTGAAGATAGGATGGTCGAAATCTTGTCCAGGTTGCCACCCAAGTCTCTGA
TGCGATTCAAATGCATACGCAAATCTTGGTGCACTCTTATCAATAGTCCATGTTTTGTAGCCAAACACCTCAGCGATTC
AGTGGACAACAAACTCTCATCCTCCACTTGTATCCTTCTCAACTGTTCTAAGGCTCACGTTTGCTCGGAAGAGAGTTGG
AAACAAGGAGTTTTATGGTCCGTGATTAATCTTTCCATTGATGGTGATGAGCTTCATTATGATgTTGAGGACCTAACTA
ATGTACCGTTTCTAAGGGATGACCAACATGAATTAGAGATTCACGGTTATTGCGATGGGATTATTTGTGTAACGGTAAA
CGAAAATTTCTTTTTGTGCAATCCTGCAACGGGGGAATTCAGGCAACTTCCTGATTCATGCCTTCTTCTACCCCTTCCC
GGTGTAAAAGAAAAATTCGGATTGGAAACGACACTTAAAGGACTGGGATTTGGTTATGATTGCAAAGCTAAAGAATACA
AGGTTGTGCGAATTATAGATAATTATGATTGTGAGTATTCAGATGATGGAGAAACATATATCGAGCATATTGCTCTTCC
TCACACTGCTGAAGTATACACCATGGCTGCTAACTCTTGGAAAGAGATCACGATTGATATATTAAGTAAAATATTATCA
TCATATAGCGAACCATATTCTTATTCAGTGTATTTGAAAGGGTTTTGTTATTGGTTGTCATGCGATGTAGAGGAATACA
TATTTTCATTTGATTTAGCTAATGAAATATCTGATATGATAGAATTGCCTTTTAGGGGAGAATTCGGTTTTAAGCGTGA
TGGTATTTTTCTGTATAATGAATCCaTCACTTATTATTGCTCTAGTTACGAAGAGCCTTCCACATTATTTGAAATATGG
GTCATGGATTACAATGACGGATTTAAGAGTCCATGGACAAAACACTTAACTGCTGGACCTTTTAAAGACATGGAGTTTC
CATTGACACCTTGGAAACGTAACGAGCTTCTTATGATTACCTCCGATGGAAGAGTTGCTTCTTATAATTCTTGTAGCGG
AAATTTCAAGTATCTTCATATTCCTGTTATTATTAATGAGAATAGGGTTGTAGATTACGTGAAAAGTATTATTCTAGTC
AAT
>M_domestica_S1_SFBB11_SFBB9_MG458560
AAGTCTCTGATGCGGTTCAATTGCATACGCAAGTCTTGGTGCACTCTCATCAATAGTCCAAGTTTTGGGGCCAAATACC
TCAGCAATTCTGTGGACAACAAACTCTCATCCTCCACTTGTATCCTTCTCAACCGTACTCAGATGCACGTTTTCCCTGA
CCAGAGTTGGAAATATGAAACTTTATGGTCCATGATGAATCTTTCCAATTATAGTGATGAGCACAACCTTCATTATCAT
TTTAAGGAACTAAATATACCGTTTCCAACGGAAGACCATCATCCTGTGCAAATTCACAGTTATTGCAATGGTATTGTAT
GTGTAATAATAGGGAAAAGTGTTCGTATTTTATGCAATCCTGCAACACGTGAATTCAGGCAACTTCCTGCTTCATGCCT
TCTTCTACCTTCCCCTCCCGAGGGAAAATTCCAATTGGAGACGATCTTTGAAGGATTAGGATTCGGCTATGATTACAAA
GCTAAAGAATACAAGGTTGTGCAAATTATAGAAAATTGTGAGTATTCAGATGATGAGCGAAGATATTATCATCGTATTG
CTCTTCCTCACACGGCTGAGGTATACACCACTACTGCTAACTCTTGGAAAGAGATTAAGATTGAGATATCAAGTAAAAC
CTATCAGTGTTACGGTTCAGAATACTTGAAGGGATTTTGCTATTGGCTTGCAAACGATGGCGAGGAATACATACTTTCA
TTTGATTTAGGTGATGAAATATTTCATATAATACAATTGCCTTCTAGGAGAGAATCTGGTTTTAAATTTTATAATATTT
TTCTGTGTAATGAATCCATTGCTTCGTTTTGCTGTTGTTATGATCCAAAGAAAGAAGATTCTACATTATGTGAAACATG
GGTAATGGACA
>M_domestica_S2_SFBB11_SFBB9_MG458561
ATGTTGAACAAAATTGCCGAAATGTCCCAGGTGCATGACAGTGAAACTCCTGAAGATGGGGTGGTCGAAATCCTGTCTA
GGTTGCCGCCCAAGTCTTTGATGCGATTCAAATGCATACGCAAGTCTTGGTGCACTCTCATCAGTAGTCCAAGTTTTGT
GGCCAAATACCTCAGCGATTCCGTGGACAACAAACTCTCATCCTCCACATGTATCCTTCTCAACCGTACTCAGATGCTC
GTTTTCCCTGACCAGAGTTGGAAATATGAAACTGTATGGTCCATGATGAATCTTTTCCATTATAGTGATGAGCACAACC

TTCATTATGATTTTAAGGACCTAAATATACCGTTTCCAACGGAAGACCATCATCCGGTGCAAATTCATAGTTATTGCAA
TGGTATTGTATGTGTAATAACAGGGAAAAGTGCTCGTATTTTATGCAATCCTGCAACACGGGAATTCAGGCAACTTCCT
GATTCATGCCTTCTTCTACCTTCCCCTCCGGAGGGAAAATTCCAATTGGAGACGATCTTTGAAGGATTAGGATTCGGCT
ATGATAACAAAGCTAAAGAATACAAGGTTGTGCAAATTATAGAAAATTGTGAGTATTCAGATGATGAGCGAAGATATTA
TCATCGTATTGCTCATCCTCATACGGCTGAGGTATACACCACGGCTGCTAACTCTTGGAAAGAGATCAAGATTGAGATA
TCAAGTAAAACCTATCAGTGTTTTTGTTCAGAATACATGAAGGGATTTTGTTATTGGCTTGCAAGCGATGGTGAGGAAT
ACATACTTTCATTTGATTTAGGTGATGAAATATTTCATATAATACAATTGCCTACTAGAAGAGAATCCGGTTTTAAATT
TTATAATATTTTTCTGTGTAATGAATCCATTGCTTCGTTTTGCTCTTGTTATGATCCAAATGATGAGGATTCTACATTA
TGTGAAATATGGATAATGGATGACTATGACAAAGTTAAGAGTTCATGGACAAAACTCTTAACCGTTGGACCCTTAAAAG
GCATTAATGAGAATCCATTGGCATTTTGGAAAAGTGACGAGCTTCTTATGGTTTCCTGTGATGGAAGAGTCACCTCTTA
TAATTCTAGTACCAAAAAACTCAGCTATCTTCATATTCCTCCTATTCTCAATGAGGTTAGAGAGTTCCAAGCTCTTATT
TATGTGGAAAGTATTGTTCCAGTC
>M_domestica_S1_SFB12_SFBB10_MG458573
AAATGCATACGCAAGTCTTGGTGCACTCTCATCAATAGTCCAAGCTTTGTGGCCAAACACCTCAACAATTCTGTGGATA
ACAAACTCTCATCCTCCACTTGTATCCTTGTCAACCATTCTCAGCCTCACATTTTCCCAGACAAGAATTGGAAACAAGA
AGTTTTCTGGTCCATGATTAATATTTCCATTGATAGTGATGAGCACAGGCTTCATTATGATGTTGTGGACCTAAATATA
CCGTTTCCATTGGAAGATCATGATTTTGTTCAGATTCACGGTTATTGCAATGGGATTGTATGTGTAATAGTAGGAAGTA
AATTTCTTTTATGCAATCCTGCAACGAGGGAATTCATGCAACTTCCCGATTCATGCCTTCTTCTACCCCCTGCTGAGGG
AAAATTCGAATTAGATACAACCTTTGAAGCATTGGGATTTGGCTATGATTGCAAAGGTAAAGAATACAAAGTCGTGCAA
GTTATAGAAAATTGTGAGTATTCAGATGATGAGCAAACATTTAATCATTGTACTACTCTTCCTCACACGGCTGAGGTAT
ACACCACGGCTGCTAACTCTTGGAAAGAGATCAAGATTGATATATCAAGCACAACCTATTCTTGGTCTTGTTCAGTGTA
CTTGAAGGGATTTTGTTATTGGTATGCAACGGATGACGACGAGGAATACGTACTTTCGTTTGATTTATGTGATGAGACA
TTTCATAGAATACAACTTCCTTCTAGGGGAGAATCTGGTTTTACATTTTTTTATATTTTTCTTCGTAATGAATCCCTTA
CATCTTTTTGCTCTCGTTACGATCGAAGTGGGGATTCTCAATCATGTGAAATATGGGTAATGGACGGTTACGATGGAGT
TAAGAGTTCATGGATAAAACTCTTAACGGTTGGAGCCTTGCAAGGCATTGAGAAGCCATTGACATTTTGGAAAAGTGAT
GAGCTTCTTATGCTTGACTCCGATGGAAGAGCCACCTCTTATAATTCTAGTACCGGAAATCTCAATTATATTCATATTC
CTCCTATTCTCAATAGGGTTGTAGATTTCGAAGTTCTTATTTATGTGAAAAGTATTGTTCCAATC
>M_domestica_S2_SFBB12_SFBB10_MG458574
ATGCTTGAAAGTGAAACTCTTGAAGAAAGGGTGGTTGAAATCTTGTCCAAGTTGCCAGCCAAGTCTCTAACGCGATTCA
AATGCATACGCAAGTCTTGGTGCACTCTCATTAATAGTCCAAGTTTTGTGGCCAAACACCTCAACAATTCCGTGGACAA
CAAACTCTCATCCTCCACTTGTATCCTTGTCAACCATTCTCAGCCTCACATTTTCCCAGACAAGAATTGGAAACAAGAA
GTTTTCTGGTCCATGATTAATATTTCCATTGATAGTGATGAGCACAGCCTTCATTATGATGTTGTGGACCTAAATATAC
CGTTTCCATTGGAAGATCATGATTTTGTTCAGATTCACGGTTATTGCAATGGGATTGTATGTGTAATAGTAGGGAAAAA
TTTTCTTTTATGCAATCCTGCAACGAGGGAATTCATGCAACTTCCCGATTCATGCCTTCTTCTACCCCCTGCTGAGGGA
AAATTTGAATTGGATACAACCTTTGAAGCATTGGGATTTGGCTATGATTGCAAAGGTAAAGAATACAAGGTCGTGCAAA
TTATAGAAAATTGTGAGTACTCAGATGATGAGCAAACATTTAATCATTGTACTACTCTTCCTCACACGGCTGAGGTATA
CACCATGGCTGCTAACTCTTGGAAAGAGATCAAGATTGATATATCAAGTACAACCTATTCTTGGTCTTGTTCAGTGTAC
TTGAAGGGATTTTGTTATTGGTATGCCACGGATGACGAGGAATACGTACTTTCATTTGATTTATGTGATGAGACATTTC
ATAGAATACCATTCCCTTCTATGGGAGAATCCGGTTTTACGTTTTTTTATATTTTTCTTCGTAATGAATCCCTTACATC
TTTTTGCTCTCGTTACGATCGAAGTGGGGATTCTCAATCATGTGAAATATGGGTAATGAACGACTATGATGGAGTTAAG
AGTTCATGGACAAAACTCCTAACTGTTGGACCCTTTCAAGGCATTGAGAAGCCATTGACATTTTGGAAAAGTGACGAGC
TTCTTATGCTTGCTTCCGATGGAAGAACCACCTCTTATAATTCTAGTATTGGAAATCTGAAGTATGTTCATATTCCTCC
TATTCTCAATAAGGTTGTAGATTTCCAAGCTCTAATTTATGTGGAAAGCATTGTTCCACTCAAG
>M_domestica_S1_SFBB9_SFBB11_MG458537
AAATGCATACACAAGTCTTGGTTCTCCCTCATCAATAGTCTAAGTTTTGTAGGTAAACACCTCAGCAATTCCGTGGACA
ACAAACTCTCATCCTCCACTTGTATCCTTCTCAACCGTTCTCAGGCCCACATTTTCCCAGACCAGAGTTGGAAACAAGA
AGTTTTCTGGTCCATGATTAATTTTTCCATTGATAGTGATGAGAACAACCTTCATTATGATGTTGAGGACCTAAATATA
CCGTTTTCATTGAAAGATCATGATTTTGTACTGATTTTTGGTTATTGCAATGGGATTGTCTGTGTAGAAGCAGGGAAAA
ATGTTCTTTTATGCAATCCTGCAACGAGGGAATCCAGGCAACTTCCCGATTCATGTCTTCTTCTCCCTTCCCCTCCTGA
GGGGAAATTCGAATTGGAGACGAGCTTTCAAGCATTGGGATTTGGCTATGATTGCAATGCTAAAGAATACAAGGTTGTG
CGAATTATAGAAAATTGTGAGTATTCAGATGATGAACGAACATTTTATCATCGTATTGCTCTTCCTCACACGGCTGAGT
TATACACCACAATTGCTAACTCTTGGAAAGAGATCAAGATCGATATATCAAGTACAACCTATTCTTGTTCTCATTCAGT
GTTCATGAAGGGATTTTGTTATTGGTATGCAACGGGGGGCGAGGAATACATACTTTCTTTTGATTTTGGTGATGACACA
TTTCATAGAATACAACTGCCTTCTAGGAGAGAATCCGGTTTTAGGTTTTATTATATTTTTCTGCGAAATGAATCCCTTG
CTTCTTTTTGCTCTCGTTACGATCGGAGTGAGGATTCTGAATCAAGT
>M_domestica_s9_SFBB11_AB539862
ATGTCTCATGTGCGTCAAAGCAAAACACCTGAAGATAGGGTAATTGAAATCTTGTCCAGGTTGCCACCCAAGTCTCTAA
TGCGGTTCAAATGCTTACACAAGTCTTGGTTCTCTCTCATCAATAGTCTAAGTTTTGTGGACAAACACCTCAGCAATTC

CGTGGACAACAAACTCCCATCCTCCACTTGTATCCTTCTCAACCGTTCTCAGGCTCATATTTTCCCAGACCAGAGTTGG
AAACAAGAAGTTTTCTGGTCCATGATTAATTTTTCCATTGATAGTGATGAGAACAACCTTCATTATGATGTTGAGGACC
TAAATATACCGTTTCCATTGAATGATCATGATTTTGTACTGATTTTTGGTTATTGCAATGGGATTGTCTGCATTGAAGC
AGGGAAAAAATGTTCTTTTATGCAATCCTGCAACGAGGGAATTCAGGCAACTTCCCGATTCATGTCTTCTTCTACCTTCC
CCTCCTGAGGGAAAATTCGAATTGGAAACGAGCTTTCAAGCATTGGGATTTGGCTATGATTCCAACGCTAAAGAACACA
AGGTTGTGAGAATTATAGAAAATTGTGAGTATTCAGATGAAGAACGAACATTTTATCATCGTATTGCTCTTCCTCACAC
GGCTGAGTTATACACCGCAACTGCTAACTCTTGGAAAGAGATCAAGATTGATATATCAAGTACAACCTATTCTTGTTCT
CGTTCAGTGTTCATGAAGGGATTTTGTTATTGGTATGCAACGGATGGTGAGGAATACATACTTTCTTTTGATTTAAGTG
ATGACAAGTTTCATATAATACAACTGCCCTCTAGGAGAGAATCCGGTTTTAGGTTTTATTATATTTTTATGCGAAATGA
ATCCCTTGCTTCTTTTTGCTCTCGTTACGATCGGAGTGAGGATTCTGAATCATGTGAAATATGGGTAATGGACGACTAC
GACGGAATTAAAAGATCATGGACAAAACTCTTAACCATTGGACCCTTACAAGGCATTAAGAAGCCATTGACATTTTGGA
AAAGTGATGAGCTTCTTATGCTTGACTCTGATGGAAGAGCCACCTCTTATAATTCTAGTACCGGAAATCTCAATTATAT
TCATATTCCTCCTATTCTCAATAGGGTTGTAGATTTCGAAGTTCTTATTTATGTGAAAAGTATTGTTCATGTCAAG
>M_domestica_S1_SFBB10_SFBB12_MG458550
ATGTCCCAATTGCATGAAATTGAATCTCCTGAAGATAAGGTGGTCGAAATCCTGTCCAGGTTGCTGCCCAAGTCGCTGA
TGCGATTCAAATGCATACGCAAGTCATGGTGCAATCTCATCAATAGTCCAAGTTTTGTAGCCAAACACCTCAACAATTC
TATGGACAACAAACTCTCATCCTCCACTTGTATCCTTCTCAACCGTTCTCAGGCTCACATTTTCCAGACCAGAGTTGG
AAACAAGAAGTTTTCTGGTCCACGATTAATCTTTCCATTGATAGCGATGAGCATAACCTTTATTATGATGTTGAGGACC
TAATTATACCGTTTCCATTGGAAGATCATGATTTTGTACTGATTTTTGGTTATTGCAATGGGATTATTTGTGTAGATGC
AGGGAAAAAATGTTCTTTTATGCAATCCTGCAACGAGAGAATTTAGGCAACTTCCCGATTCATGCCTTCTTCTACCCCCT
CCCAAGGGAAAATTCGAATTGGAAACGACCTTTCAAGCGTTGGGATTTGGCTATGACTGCAATTCGAAAGAATATAAGG
TTGTGCGAATTATAGAAAATTGTGAATATTCAGATGATGAGCAAACATTTCATCATCGTATTGCTCTTCCTCACACAGC
TGAGGTATACACCACGGCTGCTAACTCTTGGAAAGAGATCAAGATTGATATATCAAGTCAAACCTATCATTGTTCTTGT
TCAGTGTACTTGAAGGGATTTTGTTATTGGTTTGCAAGCGATAGCGAGGAATACATACTTTCATTTTATTTAGGTGATG
AGACATTCCATATAATACAATTCCCTTCTAGGAGAGAATCCGGTTTTACGTTTGATTATATTTTTCTCCGAAATGAATC
CCTTGCTTCTTTTTGCTCTCCCTACAGTCCAAGTGAGGATTCTAAATTATTTGAAATATGGGTAATGGATGACTATGAT
GGAGTTAAGAGTTCATGGACAAAATTCCTAACTATTGGACCCTTTAAAGGCATTGAGTATCCATTGACACTTTGGAAAT
GTGACGAGCTTCTTATGCTAGCCTCCGATGGAAGAGCCATCTCTTATAATTCTAGTATCGGAAATCTCAAGTATCTTCA
TATTCCTCCCATTATCAATGAGGTTGTTGATTTCGAGGCTCTTAGTTATGTGGAAAGTATTGTTCCGATCAAG
>M_domestica_S1_SFBB1_SFBB13_MG458439
ATGTCCCAAGTGCGTGAAAGTGAAACTCCTGAAGACAGGGTGGTCGAAATCTTGTCCAGGTTGTCGCCCAAGTCTCTGT
TGCGATTCAAATGCATACGCAAGTCTTGGTGCACTCTCATCAATAGTCCAAGTTTTGTGGCCAAACACCTCAACAATTC
CATGGACAACAAACTATTATCATCCACTTGCATCCTTCTCAGCCGTTCTCAGGCTCATGTTTTCCCGGATAACAGTTGG
AAACCAGAAGTTTTCTGGTCCATGATTAATCTTTCCATTGATAGTGATGAGCACAACCTTCATTATGATGTTGAGGACC
TAAATATACCTTTTCCATTGGAAGGTCATGATTTTGTACAGATTGAGGGATATTGCAATGGGATTGTCTGTGTAATAGC
AGGGACAAGTCTTTATTTGATAAATGTTCTTTTATGCAATCCTGCAACGGGGAAATTCAGGCAACTTCCCCCTTCCTGC
CTTCTTTTACCTTGCCGTCCTAAGGGAAAATTCCAATTGGAGTCGATCTTTGGAGGATTGGGATTCGGTTATGATTGCA
AAGCTCAAGAATACAAGGTTGTGCAAATTATAGAAAATTGTGAGTACTCAGATGATCAGCAATACTATTATCATCGTAT
TGCTCTTCCCCACACGGCTGAGGTATATACCATGGCTGCTAACTCTTGGAGAGTGATTAAGATTGATATATCAAGTGAA
ACCTATCATTATTCTTCTTCAGTGTACTTGAATGGATTTTTTTATTGGTTTGCAAATGATGGCGAGAAATACATACTTG
CATTTGATTTAGGTGATGAGATATTTCATAGAATACAATTGCCTTCTAGGAGAGAATCAGATTTTGAGTTTTCTAATAT
TTTTCTGTGTAATAAATCGATTGCTTCTTTTTGCTCTTGTTGCGACCCAAGTGATGAGGATTCTACATTATGTGAAATA
TGGGTAATGGATGATTATGATGGAGTTGAGAGATCATGGACAAAACTCTTAACCTTTGGACCCTTAAAAGACATTGAGA
ATCCATTTACATTTTGGAAAACTGATGAGCTTCTTATGGTTGCCGCCGGTGGAAGAGCCACCACTTATAATTCCAATAC
CAGAAATCTCAACTATCTTCATATTCCTCCTATTCTCAATGAAGTTAGAGATTTCGAAGCTCTTATTTATGTGGAAAGT
ATTGTTTCAGTGAGT
>M_domestica_S1_SFBB14_MG458600
ATGTTCCATATGCGTTTAAGCGAAACTCCTGAAGATAAGGTGGTCGAAATCCTGTCAAGGTTGCCACCCAAGTCTCTGA
TGCGATTCAAATGCACAAGCAAGTCTTGGTGCACTCTTATAAATAGTTCAAGCTTTGTTGCCAAACACCTCAGCAATTC
CGTAGACAACAAACTCTCATCCTCCACTTGTATCCTTCTCAACCGTTCTCAGATGCCGGTTTTCCCAGACAAAAGTTGG
AAATATGAAATTTTATGGTCCATGATTTATCTTTCCATTTATAGTGATGAGCACAACCATCACTATGATGTTGAGGACC
TAAACATACCGTTTCCATTAGAAGATCATCATCCTGTACAGATTCACGGTTATTGCAATGGGATTGTCTGTGTGATAGC
AGGGAAAACTGTTATTATTTTATGCAATCCTGGAACCGGGGAATTCAGGCAACTTCCCGATTCATGCCTTCTTGTACCC
CTTCCCAAGGAAAAAATTCCAATTGGAGACGATTTTTGGAGGATTGGGATTTGGTTATGATTGCAAAGCTAAAGAATACA
AGGTTGTGCAAATTATAGAAAATTGTGAGTATTCAGATGATGAGCGAACATTTTATCATAGTATTCCTCTTCCTCACAC
GGCTGAGGTATACACCATAGCTGCTAACTCTTGGAAGGAGATTAAGATTGATATATCAACTAAAACCTGTCCCAGTTCT
TGTTCAGTGTACTTGAAGGGATTTTGTTATTGGTTTGCAAGCGATGGCGAGGAATACATACTTTCATTTGATTTAGGTG
ATGAGATATTTCATAGAATACAATTGCCTTCTAGGAGAGAATCCAGTTTTAAGTTTTTTGATCTTTTTCTGTATAATGA

ATCCGTCACTTCTTATTGCTCTCATTATGATCCAACTGAGGATTCTAAATTATTTGAAATATGGGTAATGGACGATTAT
GATGGAATTAAGAGTTCATGGACAAAACTCCTAACTGTTGGACCCTTTAAAGGCATTGAGTATCCA
>M_domestica_SFBB15_MG458628
AAGTCCCTGATGCGGTTCAAATGCATACGCAAGTCTTGGTGCACTCTCATCAATAGTCCAAGTTTTGTGGCCAAACACC
TCAACAATTCCGTGGACAACAAACGCTCATCCAACACTTGTATCCTTCTCAACCGTTCTCAGATGCCCGTTTTCCCAGA
CAACAGTTGGAAATATGAAGTTTTTTGGTCCATGATTAGTCTTTCCATTGATAGTGATGAGCACAACCTTTATTATGAT
GTTGAGGACCTAAATATACCGTTTCCAATGGAAGACCATCATCCTGTAGTGATTCACGGTCATTGCAATGGGATTGTCT
GTGTAATAACAGGGAAAAATGTTGTTTTATGCAATCCTGCAATTGGGGAATTCAGGCAACTTCCCGATTGCCTCCTTCT
ACCCCTTCCCAACATAAAATTCCAATTGGAGACGAGCTTTGGAGGATTGGGATTCGGCTATGATTGCAAAGCTAAAGAA
TACAAGGTTGTGCGAATTACAGAAAATTGTGAGTATTCAGATGCTGAACGAACATATTACCATCGTATTGATCTTCCTC
ATACGGCTCAGGTATACACCACGACTGCTAACTCTTGGAAGGAGATCAAGATTGATATATCAAGTAAAAGCTATCTTGA
TTCTTGTCCAGTGTACTTGAAGGGATTTTGTTATTGGATTGCAAATGATGGCGAGGAATTCATACTTTCATTTGATTTA
GGTGATGAGATATTTCATAGAATACAAATGCCTCTTGGGAGAGAAGCCAGTTTGCAGTTTTGTAATCTTTTTCTGTATA
ATGAATCCCTTGCTTGTTTTTGCTCTCTTTACGGTCCAAGTGACAATTCTAGATTATTTGAAATATTTGAAATATGGGT
AATGGAC
>M_domestica_S1_SFBB16_MG458629
ATGTCTCAGGGGCATGAAAGTGAAGGTCCTGAAGATAGGGTGGTCGAAATCCTGTCCAGGTTGCCGCCCAAGTCTCTGA
TGCGGTTCAAATGCATACGCAAGTCTTGGTGCACCCTCATCAATAGTCCTCGTTTTGTGGCCAAACACCTCAACAATTC
CGTGGACAACAAACTATCATCCTCCACGTGTATTCTTCTCCACCGTTCTCAGACGCCCATTTTCCCTTGCGACAGTTGG
AAACGAGAATTCTTCTGGTCCATGATTAATTTTTCCATTGATAGTGATGAGAGCAACTTTCATTATGATGTTGAGGACC
TAACTAATGTACCGTTATTGCAATGGGAAGACCATCATGAAGTAGAGATTCACGGTTATTGCAATGGGATTGTCTGTGT
AACAGTAGGGGAATATTTCTTTTTGTGCAATCCAGCAACGGGGGAATTCAGTCAACTTCCCAATTCACGCCTTCTTCTA
CCCCTTCCCAGGGGAAAAGGAAAATTTGGATTGGAAACGACCGTTAAAGGATTGGGATTTGGCTATGATTGTAAAGCTA
AAGAATACAAGGTTGTGCGAATTATAGAAAATTATGATTGcGAGTATTCAGATGGTGAAGAAACATATATTGAACATAc
cGCTCTTCCTCACACGGCTGAGGTATACACAACAACTGCTAACTCTTGGAAAGAGATTAAGATAAATATATCCAGTAAA
ATATTATCATTTTACAGCTATCCCTATTCTTGTTCAGTGTACTTGAAAGGATTTTGTTATTGGTTGTCAAGCGATGACG
AGGAATACGTATGTTCATTTGATTTTGGTGATGAGATATTCGATAGGATAGAATTGCCTTCTAGGAGAGAATCTGGTTT
TAAGCTTGATGGTATTTTTCTGTATAATGAATCCATCACTTATTATTGCACTAGTTACGAAGAGCGTTCCAGATTATTT
GAAATATGGGTAATGGATAACTATGACGGAGTTAAGAGTTCATGGACAAAACATTTAACAGCCGGACCCTTTAATGGCA
TTGAGTTTCCACTGACACTTTGGAAACATGACGAGCTTCTTATGATTGCCTCCGATGGAAGAGCCACCTCTTATAATTC
TAGTACTAGAAATCACAAGTATCTTCATATTCCTGTTATTATT
>M_domestica_S1_SFBB17_MG458640
AAATGTATACGAAAGTCTTGGTGCAATCTGATCAATAGTCCACGTTTTGTGGCCAAACACCTCAGCAATTTCGTGGACA
ACAAACTCTCGTCCACCACTTGTATCCTTCTCAACCGTTCTCAGACTCATGTTTTCCCAGACAATAGTTGGAAACAAGA
AGTTTTCTGGTCCATGATCAATATTTCTATTGATAGTGATGAGCACAACCTTCATTATGATGTTGAGGACCTAAATATA
CCATTTCCGCTGGAAGATCATGATTACGTATTGATTCCCGGTTATTGCAATGGGATTGTTTGTGTGACAGCAGGTAAAA
ATATTCTTTTATGCAATCCTACAACGAGGGAATTCATGCGACTTCCCAGTTCATGCCTTCTTCTACCTTCCCGTCCCAA
GGGAAAATTCGAATTGGAAACGGTCTTTCGAGCATTAGGATTTGGCTATGATTGCAAAGCTAAAGAATACAAGGTAGTG
CAAATTATAGAAAATTCTGAGTATTCAGATGATGAGCGAACATATTATCATCGTATTCCTCTTCCTCACACGGCTGAGG
TATACACAACGGCTGCTAACTCTTGGAGAGAGATCAAGATTGATATATCAACTAAAACTTATTCCTGTTCTTGTCAAGT
GTACTTGAAGGGATTTTGTTATTGGTATGCAACGGATGCTGAGGAATACATACTTTCATTTGATTTAGGTGATGAGATA
TTTCATAGAATACAATTGCCTTCTAGAAGAGAATCCGGTTTTAAGTTTTATTATATCTTTCTTCGTAATGAATCCCTTG
CTTCGTTTTGCTCTCGTTACGATCGAAGTGATAAGTCTGAATCATGT
>M_domestica_S1_SFBB18_MG458650
ATGTCCCAGATGCGGAAAAATGAAACTCCTGAAGATAAGGTGGTTGAAATCCTGTCGAGGTTGCCGCCCAAGTCTCTCA
TGCGATTCAAATGCATACACAAGTCTTGGTGCACTCTCATCAAAAGTTCAAGTTTTGTGGCCAAACACCTCAGTAATTC
TATGGACAACAAACTCTCAACCTCCACTTGTATCCTTCTCAACCGTTCTGAAATGCCCGTTTTCCCGGACGACAGTTGG
AAGTATGAAGTTTTATGGTCCATGATTAATCTTTCCATTGATAGTGATGATCACAACCTTCATTATAATGTTGAGGACC
TAAATATACCGTTTCCAATGGAATACCATCATCCTGTATTGATTCACGGTTATTGCGATGGTATTTTCTGTGTAATTAC
AGGTGAAAATGTTGTTTTATGCAATCCTGCAATTGGGGAATTCAGGCAACTTCCCGATTCATGCCTTCTTCTACCTGCT
CCTCCTGAGAGAAAATTCGAATTGGAAACGACCTTTCGGGCATTGGGATTTGGCTATGATTGCAAAGCTAAAGAATACA
AGGTTGTGCGAATTATAGAAAATTGTGAATATTCTGATGATGAGCAAACATATAATCATCGTATTTCTCTTCCTTACAC
TGCTGAGGTATACACAACGACTGGTAACTCTTGGAAAGAGATCAATATTGATGTATCAAGTAAAGCCTATCCATGTTCT
TGTTCAGTGTACTTGAAGGGATTTTGTTATTGGTTTGCAACAGATGGCGAGGAATACATACTTTCATTTGACTTGGGAG
ATGAGATATTTTCCAGAATACAATTGCCTGCTAGGAAAGAATCCGGTTTTAAGTTTTATAGTCTTTTTCTGTATAATGA
ATCAGTCACTTCTTATTGCTCTCATTACGATCCAAGCGAGGATTCTAAATTATTTGAAATATGGGTGATGGACAACTAT
GACGGAGTTAAGAGTTCATGGAAG
>M_domestica_S9_SFBB19_MG458661

ATGGTCGAAATCTTGTCCAGGTTGCCGCCCAAGTCTCTGATGCGATTCAAATGTATACGAAAGTCTTGGTGCAATCTGA
TCAATAGTCCACGTTTTGTGGCCAAACACCTCAGCAATTTCGTGGACAACAAACTCTCATCGTCTACTTGTATCCTTCT
CAACCATTCTCAGGCTCACCCTTCCCCAGAACAGAGTTGGAAACAAGAAGTTTTATGGTTCATGATTAATCTTTCCATT
GATAGTGATGAGCACAATCTTCATTATGATGTTGAGGACCTAACTAATGTACCGTTTATGAGGGATGACTATGATGATA
TAGAGATTCACGGTTATTGCAATGGGATCGTCTGTGTAACATTAGGGGAAAATTTCTTTTTATGCAATCCTGCAACGGT
GGAATTCAGGCAACTTCCCGATTCATGTCTTATTCTACCCCTTCCCAGGGGAAAAGGCAAATTCGGATTGGAAACGACC
GTTAAAGGATTAGGATTTGGCTATGATTCTAAAGCTAAAGAATACAAAGTTGTGCGAATTATAGAAAATTATGATTGTG
AGTATTCAGATGGTGAAGAAACATATATTGAGCATATTGCTCTTCCTTACACGGCTGAGGTATACACCACGGCCGCTAA
CTCTTGGAAAGAGATCAAGATTGAATCAAGTAAAATATTATCATCTTATGGCTATCCCTATTCTTGTTCAGTGTACTTG
AAGGGATTTTGTTATTGGTGGTCTGGCGATGAACAGGAATACATATTTTCATTTGATTTAGCTGGTGAGATATTTGATA
GGATAGAATTGCCTTCTAAAAGAGAATCCGGTTTTAAGCGTGATGGTATTTTTCTGTATAATGAATCCATCACTTATTA
TTGCACTAGTTACGAAGAGAGTTCCAGATTATTTGAGATATGGGTATTGGATGACTATGACGGAGTTAAGAGTTCATGG
ACTAAACACTTAACCGCTGGACCATTTAAAGGCATTGAGTATCCATTGAAACTTTGGAAATGTGACGAGCTTCTTATGC
TTGCCTCTGATGGAAGAGTCACTTCTTATAATTCTAGAACCGGAAATCTCAAGTATCTTCATATTCCTATTATTATTAA
TAGAAATAGGGTTGTAGATTACGTTAAAAGTATTGTTCCAATCAAC
>M_domestica_S28_SFBB20_MG458663
AAATGCATTCGCAAGTCTTGGTGTACTCTTATCAATAGTCCAAGTTTTGTGGCCAAACACCTCAACAATTCCGTGGACA
ACAAACTATCATCCTCCACTTGCATCCTTCTCACACGTTCTCAACCTCTTGTTTTCCCGGACAACAGTTGGAAACCAGA
AGTTTTCTGGTCCATGATTAATCTTTCCATTGATAGTGATGAGCACAACCTTTATTATGATGTTGAGGACTTAAGTATA
CCGTTTCCTTTGGAAGATCATGATTTTGTACAGATTGACGGTTATTGCAATGGGATTGTCTGTGTAAGAGCATGGAAAA
CTCTTCATTTGGTAAATGTTCTTTTATGTAATCCTGCAGCGCGGGAATTTAGTCAACTTCCTCCTTCATGCCTTCTTCA
ACCTTCCCGTCCCAAGAGAAAATTTCAATTGGAGGCGATCGTCATAGGATTGGGATTTGGCTATGATTGCAAAGCTAAA
GAATACAAGGTTGTGCAAATTATACAAAATTGTGAGTATTCAGATGATGAGCAATACAATTATCATCGTATTGATCTTC
CTCACACGGCTGAGGTATACACCACAGCTGCTAACTCTTGGAGAAAGATCAAGATTGATATATCTAGTGGAACCTATGA
TTGTTCTTGTTCAACGTACTTGAAGGGATTTTGTTATTGGTTTGCAACCGATGGCGAGAAATACATACTTTCATTTTAT
TTAGGTGATGAGATATTTCGTAGGATACAATTGCCTTTTCGGAAAGAATCCGATTTTAAGTTTTCTAATATTTTTCTGT
GTAATGAATCCATTGCTTCTTTTTGCTCTTGTTGCGATCCAAGTGATGAGGATTCTACATTATGT
>M_domestica_S28_SFBB22_MG458665
GAAACTCTTGAAGATAAGGTGGTCGAAATATTGTCTAGATTGCCACCCAAGTCTCTGATGCGATTCAAATGCATGCGCA
AGTCGTGGTGCACTCTCATCAATAATCCAAGTTTTGTGACCAAACACCTCAACAATTCCGTGGACAACAAACTCTCATC
CTCCATTTGTATTTTTCTCAACCGTTCTCAGGCGCACATTTTCCCAGACCAGAGTTGGAAACAAGAAGTTTTCTGGTCC
ATGATTAAGCTTTCCATTGATAGTGCTGATCACAACCTTCATTATGATGTTGAGGACCTAAATATACCATTTCCATTGG
AAGATCACGATTTTGTAATGATTTTTGGTTATTGCAATGGAATTCTCTGTGTAGAAGCAGGAAAAATGATTCTTTTATG
CAATCCTACAACGAGGGAATTCAGGCAACTTCCCGTTTCATGCCTTCTTCTACCCCCTCCCAAGGGGAAATTCGAATTG
GAAACGACCTTTCAAGCATTGGGATTTGGCTATGATTGCAATGCTGAAGAATACAAGGTTGTGCGAACTATAGAAAATT
GTGAGTATTCAGATGATGAGCAAACATTTTATCATCGTATTGCTCTTCCTCACACGGCTGAGGTATACACCACGACTTC
AAATTCTTGGAAAGAGATCAAGATTGATATATCAAGTGATACGTATTCATGTTCTTGTTCAGTGTACTTAAAGGGATTT
TGTTATTGGTATGCAAGGGGTGGCGAGGAATACATACTTTCATTTCATGTAGGTGATGAGACATTTCATATAATACAAT
TTCCTTCTAAGAGAGAATCCGGTTTTACATTTGATTATATTTTTCTCCGAAATGATTCCCTTGCTTCTTTTTGCTCTCC
TCACTATCCTAGTGAGGATTCCAAATTATTTGAAGTATGGGTAATGGACGACTATGATGGGATTAAGAGTTCATGGACA
AAACTCCTAACAGTTGGACCCTTTAAAGGCATTCAGTATCCATTGACACTTTGGAAATGTGATGAGCTTCTTATGCTTG
CCTCTGGTGGAAGAGCCATATCTTATAATACTAGTACCGGAAATCTCACGTCTCTTCATATTCCTCCAATTATCAACAG
GGTTGTAGATTTCAAAGCTCTTATTTATGTGAAAAGTATTGTTCCACTCAAG
>M_domestica_S25_SFBB23_MG458666
AAAGAATACAAGGTTGTGCAAATTATAGAAAATTGTGAGTATTCAGATGACCAGCAATACTATTATCATCGTATCGCTC
TTCCTCACACGGCTGAGGTATATACCATGGCTGCTAACTCTTGGAGAGTGATCAAGATTGATATATCAAGTGAAACCTA
TCATTATTCTTCTTCAGTGTACTTGAATGGATTTTTTTATTGGTTTGCAATTGATGGCGAGAAATACGTACTTTCATTT
GATTTAGGTGATGAGATATTTCACAGAATACAATTGCCTTCTAGGAAGGAATCCGATTTTGAGTTTTCTAATATTTTTC
TGTGTAATAAATCGATTGCTTCTTTTTGCTCTCGTTGCGACCCAAGTGATGAGGATTCTACATTATGTGAAATATGGGT
AATGGATGATTATGACGGAGTTGATAGATCATGGACAAAACTCTTAACCTTTGGACCCTTAAAAGACATTGAGAATCCA
TTTACATTTTGGAAAACTGATGAGCTTCTTATGGTTGCCGCCGGTGGAAGAGCCACCACTTATAATTTCAGTACCGGAA
ATCTCAACTATCTTCATATTCCTCCTATTCTCAATGAAGTTAGAGATTTCGAAGCTCTTATTTATGTGGAAAGTATAGT
TCCAGTCAAG
>M_domestica_S1_SFBB24_MG458668
AAGTCTCTGATGCGATTCAAATGCATACGCAAGTCTTGGTGCACTCTCATCAATAGTCCAAGTTTTGTGGCCAAACACC
TCAACAATTCTGTGAACAACAAACTCTCATCCTCCACTTGTATCCTTCTCAATCGTTCTCAGCCTCACGTTTTTCCGGA
CAATAGTTGGAAGCTAGAAGTTTTCTGGTCCATGATTAATCTTTCCATTGATTGTGATGAGCACAACCTTCATTATGAC
GTTTCGGACCTAAATATACCGTTTCCACTGAAAGATCATGGCTTTGTACAGATCGACGGCAATTGCAATGGGATTTTTT

GTATAATAGCAGGGAAAAGTCGTTATTTTATAAATGTTCTTTTATGCAATCCTGCAATAGGGGAATTCAGGCAACTTCC
CCATTCATGCCTTCTTCTACCTTTCCCTCCCAAGGGAAAATTCGAATTGGAGACGATCTTTGCAGGATTGGGATTTGGC
TATGAATTCAAAGCTAAAGAATACAAGGTTGTGCAGATTATACAAAATTGTGAGTATTCAGATGATTTGAGAACATATT
ATCATCATATTGCTCTTCCTCACAGGGCTGAGGTATACACCACGGCTGCTAATTCTTGGAGAGAGATCAAGATTGATAT
ATCAAGTGAAACCTATCATTTTTCTTGCCCAGTGTACTTGAAGGGATTTTGTTATTGGTTTGCAACCGATGGAGAGGTA
TACATACTTTCATTTGATTTAGGTGATGAAATATTTCATAGAATACTATTGCCTTCGAGGAGAGAATCCAACTTTGAGT
TTTGTAATCTTTTTCTGTGTAATGATTCCATTGCTTCTTTTTGCTCTTGTTGGGATCCAAGTGATGAGGATCGTACATT
ATGCGAAATATGGATAATGGGTGACGGAGTTAAGAGTTTATGGACAAAACTCCTAACCTTTGGACCCTTGAAAGGCATT
GAGAAACCATTTGCGTTTTGGAAAAGTGACGAGCTTCTTATGGTTTCCTTCGATGGAAGAGCTACCTCTTATAATTCTA
GTACCGGAAATCTCAACTATCTTCATGTTCCTCCTATTCTCAATCAAGTTAGAGATTTCCAAGCTCTTATGTATGTGGA
AAGTATTGTTCCAATCAAG
>P_axillaris_S19_SLF11_AB933047
ATGGTAGACGGAATTATGAAGAAGTTTCACGAAGATGTGGTGATTTATATTCTGTTACGGCTGCCAGTGAAATCCCTCA
TGCGATTGAAATGCATCTCTAAAACATGGTACTCTCTCATGCAATCATCAACCTTCATCAATCTTCATCTCAATCGTAC
TACAACCTATAATGATGAATTAATTTTCTTCAAGCGCTCCATCAAATTAGAACCAGACCTTTTTAAAAACAAACTATCT
TTTCTTTATAGTGACAATGAGGATGACCTAACCCCTGTTTATCCAGATATAGATGTGCCATATTTGACCTCTGATTATT
GCAGTCGTTTTCATCAGCTTATTGGTCCTTGTCGTGGTTTGATTGCTTTGACAGACTTTACAGTTATTGTGTTATTGAA
TCCAGCCACTAGAAAATACAGGCTTCTCCCAGGCAGCCCTTTTGTTTGTCCAAAGGGTTTCACATTTGTCACGAGAGGT
GTTGGGTTTGGCTACAGCACGGCAGAAAATTATTACAAGTTAGTTAGGATTTTTGAAGTGTATACGGATCCTTATGATA
GGGATCTCGATGCGAGGCATTCCAAAGTTGAGGTATATGATTCGTGCACTGATTGTTGGAGAGACCTGGATCTTACGGT
AAAGTTGCTACCAAAAGTGCGGAGATTTGCTTGTTCTGAGATATTTTACAAGGAAATATTTCATTGGTGTGCACATGAC
GACACAGTGATGATTCTATGTTTTGACATTAGCCTTGAAACTTTTCATTACATGAAATTGCCTGATCATTGTCATTTCT
GGGACAATAAAGGTTATGGCCTCGCAGTACTAAATAACTATCTAACGTTTATTACCTATCCCAATCCAAGATGCGCGCT
TGATCCTGGACAAGAGTTTACGGATATTTGGATAATGGAGGAGTACGGTGTAAACGGGACCTGGATTAAGAAATACACA
ATCAGACCTCTTCCAATTGAATCCTCATTAGCAATTTGGAAGGATAATTTATTACTTCTTCAGAGCATAAGTGGAACTT
TGAGCACATATAATCTTAATTCCGATGAACTCAAGGAATTCAACCTCCAAGGTTTACTAGTACTCTGAGACTTGTAGT
TTACAAGGAAAGCTTGACCATAATTCCAAGAGAAAGCGATAATGGCACTGAAGTTCAAAATTTTTTAGAGTATGACAAT
TTGACT
>P_hybrida_S22m_SLF8A_AB933129
ATGTTGGATGGAATTATGAAACATTTGCCTAAAGATATAGCAATGTATATACTTTTGAGGTTTCCAGTGAAATCTCTTT
TACGATTCAAATTCATCTCTAAAAGTTGGTCCACTCTCATAGAATCCTCCACATTTATCAATATTCATCTCAAGCGCGC
TACAACAACCAATAATGAATTTCTTCTTTTCAGCCGCTCCTATAGAGAGGAAACAGAAGGATTTAAAAATGTCTTATCT
ATTCTTTCTGGTGGCAACAACGACGATCTTATGCCCGTTGTTTCAGATCTGGAACAACCATATCTAACCTTCACTGAAT
ACTATCTTTTCAATAAACTCGTTGGCCCTTGTAATGGTTTGATTGTTCTAACAGATTTTGAGATTATTGTCTTATTTAA
TCCAGCTACTAAAAAATTACATGCTAATCCCGCCTAGCCCTTTTGTTTGTCCAAAGGGTTTTCATCGCTCCTTTAGAGGG
GGTGTTGGGTTTGGTTTTGACTCTATTGTGAAGGACTACAAGTTTGTCACGATTTCAGAAGTTTTTAAAAATTCTGAAT
GGGGACCCGATGAGAAAGAGCAAAAAGTAGAGGTTTATGATATGCGTATTGATTCCTGGAGAGATTTGAATCATGTGGA
TCTACAGTTGCCTACTGTGTATTATTATCCATGTTTTGAGATGCTTTATAATGGAGCCTTTCATTGGTATGCAATTAAT
GATAGATTTGATCATGTAATTCTTAGCTTTGATATAAGTACTGAGATCTTTCACAGCATAAAGATGCCAGCTACTGGTA
AATCCTCTGGCGGAAAGAAGTATTGCCTCATAGTCTTGAACGAGTCTCTAACGTTGATTTGTTATCCCAATCCCGATTG
CGAGATGGATCCGACGAAAGATTCAATGGACATTTGGATAATGATGGAGTATGGTGCATACGAGTCTTGGACTAAGAAA
TACATAATTAAACCTCTTCCTATAGAATCCCCATTAACAATTTGGAGGGATCATTTATTGCTTCTTCAAAGCAAAAGAG
GACTCCTCGTTTCCTACGATCTCAGTTCAAATGAAGTAAAGGAATTCGACTTGCATGGCCATCCTAAAAGTTTGAGAGT
TCTAGTTTACAAGGAAAGTCTGATTTCCTTACCAAAAAGGGGATGCGAGCATGGTACAAAATTTAAAAATTGT
>P_axillaris_S19_SLF8_AB933044
ATGATGTTGGATGGAATTATGAAACATTTGCCTGAAGATATAGCAATGTATATACTTTTAAGGTTTCCAGTGAAATCTC
TTTTACGATTTAAATTCATTTCCAAAAGTTGGTCCACTCTCATAGAATCCTCAACATTTATCAATATTCATCTCAATCG
TGCTACAACAACCAAAAATAAATTCCTTCTTTTTAGCCGCTCCTACAGAGAGGAAACAGAAGGATTTAAAAATGTCTTG
TCTATTCTTTCTAGTGGCAACAACGACGATCTTATCCCCGTTGTTTCAGATCTGGAATTGCCATATCTAACCTTCACTG
AATACTATCTTTTCAATAAACTCGTTGGCCCTTGTAATGGTTTGATTGTTTTAACAGATTTTGAGATCATTGTCTTATT
TAATCCAGCTACTAAAAAATTACATGCTAATCCCGCCTAGCCCTTTTGTTTGTCCAAAGGGTTTTCATCGCTCCTTTAGA
GGGGGTGTTGGGTTTGGTTTTGACTCGATTGTGAAGGACTACAAGTTTGTCACGATTTCAGAAGTTTTTATGGATTCTG
AATGGGTACCTGATGAGAAAGAGCAAAAAGTAGAGGTTTATGACTTGCGTTTTGATTCTTGGAGAGATTTGAATCATGT
GGATCAACAGTTGCCTACTGTGTATTATTATCCATGTTTTGAGATGCTTTATAATGGAGCCTTTCATTGGTATGCAATT
AATGATAGATTGGATCATGTAATTCTTAGCTTTGATATAAGTACTGAGATCTTTCAAAGCATAAAGATGCCAGCTACTG
GTAAATCCTCTGGTGGGAAGAAGTATGGCCTCATAGTCTTGAACGAGTCTCTAACGTTGATTTGTTATCCCAATCCAGA
TTGCGAGATGGATCCATCTAAAGATTCAATGGACATTTGGATAATGATGGAGTATGGTATATACGAGTCCTGGACTAAG
AAGTACATAATTAAACCTCTTCCTATAGAATCGCCATTAACAATTTGGAGGGATCATTTATTGCTTCTTCAAAGCAAAA

GTGGACTTCTTGTTTCCTACGATCTTAGTTCCAATGAAGTAAAGGAATTCGACTTGCATGGTTATCCTAAAAGTTTGAG
AGTTCTAGTTTACAAGGAAAGCCTTATTTCCATTCCAAAAAGGGGATGCAAGCATGGTACAAAATTTAAAAATTGTCGA
AAAGGTATAACTATTTCATAT
>P_axillaris_S19_SLF8_AB933043
ATGACGTTGGATGGAATTATGAAACATTTGCCTGAAGATATAGCAATGCATATACTTTTAAGGTTTCCAGTGAAATCTC
TTTTACGATTTAAATTCATTTCCAAAAGTTGGTCCACTCTCATAGAATCCTCAACATTTATCAATATTCATCTCAATCG
TGCTACAACAACCACAAATGAATTCATTCTTTTCAGCCGCTCCTACAGAGAGGAAACAGAAGGATTTAAAAATGTCTTG
TCTATTCTTTCTAGTGGCAACAACGACGATCTTATCCCCGTTGTTTCAGATCTGGAACTGCCATATCTAACCTTTACTG
AATACTATCTTTTCAATAAACTCGTTGGCCCTTGTAATGGTTTGATTGTTTTAACAGATTTTGAAATTATTGTCTTATT
TAATCCAGCTACTAAAAATTACATGCTAATCCCGCCTAGCCCTTTTGTTTGTCCAAAGGGTTTTCACCGCTCCTTTAGA
GGGGGTGTTGGGTTTGGCTTTGACTCGATAGTGAAGGACTACAAGTTTGTCACGATTTCAGAAGTTTTTAAGGATTCTG
AATGGGTACCTGATGAGAAAGAGCAAAAAGTAGAGGTTTATGATTTGCGTTTTGATTCTTGGAGAGATTTGAATCATGT
GGATCAACAGTTGCCTACTGTGTATTATTATCCATGTTTTGAGATGCTTTATAATGGAGCCTTTCATTGGTATGCAATT
AATGATAGATTGGATCATGTAATTCTTAGCTTTGATATAAGTACTGAGATATTTCACAGCATAAAGATGCCAGCTACTG
GTAAATCCTCTGGTGGGAAGAAGTATGGCCTTATAGTCTTGAACGAGTCTCTAACGTTGATTTGTTATCCCAATCCAGA
TTGCGAGATGGATCCGTCTAAAGATTCAATGGACATTTGGATAATGATGGAGTATGGTGTATACGAGTCTTGGACTAAG
AAGTACATAATTAAACCTCTTCCTATAGAATCCCCATTAACAATTTGGAGGGATCATTTATTGCTTCTTCAAAGCAAAA
GTGGACTTCTTGTTTCGTGCGATCTTAGTTCCAACGAAGTAAAGGAATTCGACTTGCATGGTTATCCTAAAAGTTTGAG
AGTTCTAGTTTACAAGGAAAGCTTGATTTCCATTCCAAAAAGGGGATGCAAGCATGGCACAAAAATTAAAACTTGT
>N_alata_DD6_EF420256
ATGATGTTGGATGGAATTATGAAAAAATTACCTGAAGATGTTGTGATTTATATACTTTCGAGGTTTTCAGTGAAATCTC
TTTTACGATTTAAATTCATCTCCAAGAGTTGGTACACTCTTATACAATCATCAACATTTATCAATGTTCATCTCAACCG
CAGTACCATAACAAAAAACGAATTCATTCTTTTCAGCCGTTCCTTCAGAATAGAGACCGAAGGATTTAAAAATGTTTTA
TCTATTATTTCTAGTGACGATTACAATGATCTTAATGTCGTTTTACAAGATCTAGATCTGCCATATCTAACATTCACTC
CTAACTATCATTTTAATGAACTCGTTGGCCCTTGTAATGGTTTGATCGTCTTAACGGATGATGATGATATTATCGTCTT
ATTTAATCCAGCTACTAAAAATTACATGCTACTCCCACCTAGCCCTTTTGTTTGTTCAAAGGGTTATCATCGCTCCTTT
ATAGGTGGCGTTGGGTTTGGTTTTGATTCAATTGGGAATGACTACAAGTTTGTCAGGATTTCAGAAGTTTTTCTCGATA
CTTATTGGGGCCCCGAGGAGAGGGAGCAAAAAGTAGAGGTTTATGATTTGCGTAGTGATTCTTGGAGAGATTTGAATCA
TGTGGATCAACAATTGCCTACTATCTTTTGGAATCAATGTTTCGAGATGCTTCATAATGGAGCCTTTCATTGGTATGCA
GTTGGAGACTTAACGTATGAAATCCTTTGCTTTGACTTTAGCACTGAGATTTTTCGAAGCATGAAGATGCCAGAGAGTT
GTAATGCCTATGATGGGAAGCGTTATAGCCTTGCAGTCGTGAATGAGTCTCTAACATTAATTTGTTATCCCAGCCCAGA
CAGTGAGATTGATCAGACTCAAAATACCATGGATATCTGGATAATGATGGAGTACGGAGTAAATGAGTCTTGGACTAAA
AAATACATAATCAGTCCTCTTCCTATTGAATCCCCATTAACCATTTGGAGGGACCATTTATTGCTTCTTCAAAGCAAAA
CTGGACAGTTAATATCCTACAATCTTCGTTCCAATGAAGTCAAGGAATTCGATTTACGTGGTTATCCTGAAAGCTTGAG
AGCTATAGTTTACAAGGAAAGTTTGATTTCAGTTCCAAAAACGAAGACGCGAGCA
>P_hybrida_s7_FBX1_AB932986
ATGGCGGATGGAATTATGAAAAGACATCCCGAAGACGTACTAATATATATACTATGTAAGTTTAGAGTGAAATCCCTTC
TACGATTCAAAGGCGTTTCCAGGACATGGTACTCTCTCGTACAATCCTCAACATTCATCAATCTTCATCTGAATCGAAC
TACAACCACCAGAGATGAATTCATTATTTTCAGCCGCTCCGTCAGAAAAGAACCCAACGGATTTAGAAATGTCCTGTCT
ATACTTTCAAGTGACAATGACGATGATCTTAACCCTGTTTTTCCGGATCTAGACCCACCATATCTAACCTTCACTGAGT
ACTATGTTTATAATAAACTCGTCGGTCCTTGCAATGGTTTGATTGCTTTGACAGATTTTGAAGTTATTGTCTTATTTAA
TCCAGCGACTAGAAATTATATGCTACTCCCACCTAGCCCTGCTTGCCCAAAGGGTTTTCGTCGCAACTTTCGAGGTGGC
GTTGGGTTTGGTTTCGATTCGATTAGGAACGATTACAAGTTTGTTAGAATTTCAGAACTCTGCATGGATTCAGATTGGA
TTCCCGTTGAAGAACAAAAAGTTGAGGTTTATGATTTGAGCATTGATTCTTGGAGAGAATTGGATCATGTAGATCGACA
GTTACCTACTGTGCATTGGTTGCCACATTTCGAGATCTTTCACATGGGATCCTTCCATTGGTATGCAGATACAGACACA
GACACAATGGTAATTCTTTGTTTTGACATGAGCACTGAGATTTTTCGCAACGTGATGATGCCTGATTCTTGTAATGGCT
ATGATGGGAAGTGTTATAGCCTTAAAATACTGAATAGGTCTCTAACATTGATTTGTTACCCGGATCCATTTAGCGATAG
TGATCCTACACAAGATTCAATGGTCATCTGGATAATGATGGAATACGGTGCATACGAGTCTTGGACTAAGGAATACACA
ATCAGACCTCTTCCTATTGAATATCCATTAACAATTTTGAGGGATCATTTATTTTTTCTTGAAAGCAAAAGTGGACATC
TTGTTTGCTATAATCTTACTACCGATGAAGTAAAAGAATTCAATTTACATGGTTATCCTGAAAGTTTAAGAGTTATGGT
TTATAAGGAAAGCTTAACTTCAATTCCGAAAAGAGTACAA
>P_hybrida_S9_FBX1_AB933000
ATGGTCAATGGAGTTATGGAGAAATTGTCCGAAGATGTGTTTATTTATACACTTCTAAGGCTTCCGGTGAAATCACTCA
TGCGATTCAAATGTGTCTCTAAAGTAGGGTACACCCTAATACAGTCATCCGCTTTCATAAAGTTTCATCTCAACCGCAC
CACAACCACAGAGGAAGAACTCATTCTTTTGAAGCGCTCCATTGAAGAAGAATCTACCCGATATAAAACTATCTTGTCT
TTTCTTTCTAACGATGATGATAATCTTAACCCACTTTTTCCTGATTTGGATGTGCCATGCCTGGTATCTACTTGTAGTA
GTAATTTTGATCAACTGTTTGGTCCATGTCAGGGTTTGATTGCATTAATAGATTACGTTAACACTTTCTTATTAAATCC
AGCTACTAGAAATTATAGGATAATCCCGCCCAGCCCTTTGAGTAGTCAAGAAAGGGTGCATCTCTACGTTCAGGGTGCA

GGATTCGGGTTTGACTCGATTGCGAAAGAGTATAAAATTGTCATGATTGAAGTGATTTACAGGGACACTCCTTGCAAGG
ATCCTGAACTAGGAGAGAAGAAAGTTGAGGTTTATGATTTTAGCATTGATTCTTGGAGAGAACTTGATCATGTAAGTAA
TGACCTGCCCACGATGTTCTGGGTGCCTTGTTCTGAGATGTTTTACAAGGGAGCCTGCCATTGGTTTGCACTTTCCGAA
CCAGACACTGTGGATATTCTTTGTTTTGACTTGAGCAGTGAAGTTTTTCGGATCATGAAAATGCCTGATAGCTGTCGAT
TCTTCAATGGACCTAGTTATGGCCTCCTAATCAAGAATGAATCTCTAACCTTAATTTGTTATCCCGACGAAGAGTCTGA
GGTTTATCCAAGACAGGAGTCGATAGATATTTGGATAATGAAGGAATACGGTGCATATGACTCTTGGATTAAAAAACAA
ACTATTAGACCTCTGCCTATTGAAATACCATTATCAATTTGGAAGGATGATTTATTGCTTTTTCAAAGCAAAAATGGAT
TTCTGCTTTCATATGATCTTAATTCCGGTGAAGTTAAACAATACAATTTACATGGTTGTCCGAAGAGTTTGAGAGCTGT
AGTTTACAAAGAATGCCTGACCCAAATTCAAAGAGGAGGCGAGCATAGCACTGAAGTTCAAAAATTT
>P_hybrida_Sm_SLF2_AB933091
ATGATTAAACTGCCTGAAGATGTGGTGATTTATATACTTTTAAGATTGACAGCGAAATCTCTCTTGCGATTTAAATGTA
TTTCTAAGTATTGGTACACTCTTATCTTATCAAACACTTTTGTCAAACTTCATCTCAACCGCATCACAACCATAAAAGA
TGAATTCATCTTTTTGATTCGCACGTTCAGAGAAGAACCAGATCAATTGAAAAGTATTGCATCTTTTTTTTCTGGTGAT
GATAATAATAATCTTAGCACTCTTTTTCCAGATTTAGATGTGTCAGATCTTACCTCCTCTTCTTGTACTGTTATTAATC
AAATCATCGGTCCTTGCCACGGTTTAATTGCTTTGACAGATTCTTTTATCATAATCATACTTAACCCAGCTACTAGAAA
ATATGTTGTGCTTCCACCTAGCCCTTTTGGATGTCCAAAAGGTTACCATCGTTCCATTGAAGGTATTGGTTTTGGATAT
GATTCAATTGTGAACGAATACAAGGTTGTCAGGCTTTCAGAAGTTTATTGGGATCCTCCTACCGATTATTTTGGTCCTA
GAGAACCCAAAGTTGATATTTTTGATTTGAGCATTGATTCTTGGCGAGAACTTGATGTAGAATTTCCCTCGATATATTA
TCTGCCTTGCTCTGAGATGTATTACAAGGAAGCAGTTCATTGGTTTATTATTACAGACACAGTAGTCATTCTTTGTTTT
GATATCGGCACTGAGATTTTCCGCACAATGGATATGCCTGGAACCTGTACTTTTCTCGGCGGGCCACGTTATGGACTTG
CTGTTTTATATGAGTGCCTAACATTGATTTGTTACCCTGATCCAATGAGTTCAATTGACCCAACAAAAGATCTAATTGA
CATTTGGATGATGGAGGAGTATGGAACAAGCGAGTCTTGGATTAAGATATACACAATTAAACCTGTTCCTATTCCTATC
GAATCCCCACTAGCCATTTGGAAGGATCATTTGTTGCTTCTTCAGACAAAAAGTGGATATTTAATTTCCTATGATCTTA
ATTCCGATGAAGTGAAGGAATTCAATTTGAATGGCCATCTTGAAAGTTTGAGAGTTATAGTTTACACAGAAAGCATAAC
TGTGATTTCAAGAATAAGCGAACATGGTACACAAGTTCTGCAATTT
>N_alata_DD4_EF420254
ATGGTGGGTGGAATTATAAAGGCAGTTCCCGAAGATGTTGTTATTTATGTACTCATAAGGCTTCCCGTAAAATCTATCA
TGCGATTCAAATGCACCTCGAAAACTTTATACATTCTCATACGATCCACCTCTTTCAGCGATATTCACCTTAATCACAC
CACAACCTCACAAGATGAATCAATTCTCTTCAAGCGTTCCTTCAAAGAAGAAGCCAACCAATTTAAAAATGTCATATCT
TTTCTTTTTGGTGTTGATGATGCCGGTTTTGATCCTCTTCTTCCAGATCTAGAGGTGCCACATCTGACCACTGATTATG
GCAGTATTTTTCATCAACTCATAGGTCCTTGCCATGGTTTGATTGCTTTGACGGATTCGGTTCAAACCGTGTTGCTTAA
TCCAGCTACGAGACATTACCGGCTACTGCCGCCTTGCCCTTTTGGTTGTCCAAAGGGTTACCATCGTACCATTGAAGGT
GTTGGGTTTGGTTTCATCTCAATTCTGAATGACTTCAAGGTTGTAAGAATTTCAGATGTTTTCTGGGATCCGCCTTACG
GATATCCCGAAGGCCGAGATAGCAAAGTTGATATTTATGAGCTAAGCACCGATTCATGGAGGGAACTCGAACCTGTACA
GGTGCCTAGGGTATATTGGTTGCCTTGTTCTGAGATGGTTTACCAGGAAGCAGTTCATTGGTTTGCAACTATAGAAGAA
GTGGTCATTCTTTGTTTTGACATAGTAACAGAGACTTTTCGCAATATGAAAATGCCAGATGCGTGTTATTCTATTAAGC
AGTCACGTTATGGCCTTATTGTCTTAAATGAGTCTCTAGCGTTGATTTGCTACCCCGATCCACGGTGTGCAGTTGACCC
AACGCAAGATTTTATTCACATTTGGTTGATGGAGGAGTACGGTGTAAGCGAGACTTGGATTAAGAAATACACAATTCAA
TCTCTCCCAATTGAATCCCCATTAGCTGTTTGGAAGGATCATTTATTGCTACTTCAAAGCAAAATTGGGCAACTTATAT
CCTATGATGTTAACTCCGATGAAATGAAGGAATTCGATTTACATGGCTTTCCCAAAAGTTTGAGAGTTATAGTTTTCAA
GGAAAGCTTAACTTCGATTCCAAGTGGAAGCGAGCATGGTACACGAGTGCAAAAGTTT
>P_axillaris_S19_SLF1_AB933040
ATGGCGAATGGTATTTTAAAGAAATTGCCCGAAGATTTGGTGTTTCTTATACTATTAACATTTCCAGTGAAATCTCTTC
TGCGATTCAAATGTATCTCTAAAGCTTGGTCCATTCTCATACAATCCACCACTTTCATAAACCGTCATATCAATCGCAA
AACAAACACAAAAGCTGAATTCATTCTCTTTAAGCGTTCCATCAAAGATGAAGAAGAAGAATTTATAAATATCTTGTCT
TTTTTTTCTGGTCATGATGATGTTCTTAACCCTCTTTTTCCAGATATAGATGTCTCATACATGACGTCAAAATGCGATT
GCACTTTTACTCCACTCATCGGTCCTTGTGATGGTTTGATTGCTTTGACAGATACCATAATCACCATAATACTTAATCC
GGCTACCAGAAACTTCAGACTGCTCCCACCTAGCCCTTTTGGTTGTCCAAAAGGTTACCATCGTTCTGTTGAAGGGGTC
GGATTTGGCTTTGACACCATTTCAAATTACTATAAGGTTGTTAGGATTTCTGAAGTTTATTGTGAAGAAGCTGATGGTT
ATCCTGGTCCTAAAGATAGTAAAATTGATGTTTGTGATTTGAGTACTGATTCTTGGAGAGAATTGGACCATGTACAGTT
GCCATCGATTTATTGGGTGCCTTGTGCTGGAATGCTTTACAAGGAAATGGTTCACTGGTTTGCAACTACGGACACGTCG
ATGGTTATTCTTTGTTTTGACATGAGTACTGAGATGTTTCATGATATGAAAATGCCTGATACTTGTTGTAGGATTACAC
ACGAGCTGTATTATGGCCTTGTAGTCTTATGTGAGTCTTTCACATTGATTGGTTACTCCAACCCAATAAGTTCTATTGA
TCCAGTAGAAGATAAAATGCACATTTGGGTGATGATGGAGTACGGTGTAAGCGAGTCTTGGATTATGAAATACACGATT
AAACCTCTTTCTATTGAATCCCCTTTAGCTGTTTGGAAGAATCATATATTGCTTCTTCAAAGTAGAAGTGGACTTCTAA
TTTCCTATGATCTTAATTCCGGTGAAGCAAAGGAATTGAACTTACATGGTTTTCCAGACACTTTGAGTGTTAAGGTTTA
CAAAGAATGCTTAACTTCAATTCCAAAAGGGAGCGAGTACAGTACAAAAGTACAAAAATTT
>P_axillaris_S19_SLF_AY766154

ATGGCGAATGGTATTTTAAAGAAATTGCCCGAAGATTTGGTGTTCCTTATACTATTAACATTTCCAGTGAAATCTCTTC
TGCGATTCAAATGTATCTCTAAAGCTTGGTCCATTCTCATACAATCCACCACTTTCATAAACCGGCATATCAATCGCAA
AACAAACACAAAAGCTGAATTCATTCTCTTTAAGCGTTCCATCAAAGATGAAGAAGAAGAATTTATAAATATCTTGTCT
TTTTTTTCTGGTAATGATGATGTTCTTAACCCTCTTTTTCCAGATATAGATGTGTCATACATGACGTCCAAATGCGATT
GCACTTTTACTCCACTCATCGGTCCTTGTGATGGTTTGATTGCTTTGACAGATACCATAATCACCATAGTACTTAATCC
GGCTACCAGAAACTTCAGAGTGCTCCCACCTAGCCCTTTTGGTTGTCCAAAAGGTTACCATCGTTCCGTTGAAGGGGTC
GGATTTGGCTTTGACACCATTTCATATTACTATAAGGTTGTTAGGATTTCTGAAGTTTATTGTGAAGAAGCTGATGGTT
ATCCTGGTCCTAAAGATAGTAAAATTGATGTTTGTGATTTGAGTACTGATTCTTGGAGAGAATTGGACCATGTACAGTT
GCCATCGATTTATTGGGTGCCTTGTGCAGGAATGCTTTACAAGGAAATGGTTCACTGGTTTGCAACTACAGATACGTCG
ATGGTTATTCTTTGTTTTGACATGAGTACTGAGATGTTTCATGATATGAAAATGCCTGATACTTGTAGTAGGATTACAC
ACGAGCTGTATTATGGCCTTGTAATCTTATGTGAGTCTTTCACATTGATTGGTTACTCCAACCCAATAAGTTCTATTGA
TCCAGTAGAAGATAAAATGCACATTTGGGTGATGATGGAGTACGGTGTAAGCGAGTCTTGGATTATGAAATACACTATT
AGACCTCTTTCTATTGAATCCCCTTTAGCTGTTTGGAAGAATCATATATTGCTTCTTCAAAGTAGAAGTGGGCTTCTAA
TTTCCTATGATCTTAATTCCGGTGAAGCAAAGGATTTGAACTTACATGGTTTTCCAGACAGTTTAAGTGTTAAAGTTTA
CAAGGAATGCTTAACTTCAATTCCAAAAGGGAGCGAGTACAGTACAAAAGTACAAAAATTT
>Nalata_DD1_EF420251
ATGGTGGGTGGAATTATAAAGGCAATTCCCGAAGATGTTGTTATTTATGTACTCATAAGGCTTCCCGTAAAATCTATCA
TGCGATTCAAATGCACCTCGAAAACTTTATACATTCTCATACGATCCACCTCTTTCAGCAATATTCACCTTAATCACAC
CACAACCTTACAAGATGAATTAATTCTCTTCAAGCGTTCCTTCAAAGAAGAAGCCAACCAATTTAAAAATGTCATATCT
TTTCTTTTTGGTGTTGATGATGTCGGTTTTGATCCTTTTCTTCCAGATCTAGAGGTGCCACATCTGACCACTGATTATG
GCAGTATTTTTCATCAACTCATAGGTCCTTGCCATGGTTTGATTGCTTTGACAGATACCATAACCACAATCTTAATTAA
TCCGGCTACTAGAAATTTCAGATTGCTCCCACCTAGCCCTTTTGGTTGTCCCAATGGTTACCATCGTTCTGTTGAAGCG
CTTGGGTTTGGCTTCGACTCGATTGCGAATGACTATAAAATTGTTAGGCTTTCTGAAGTGTTTTGGGATCCTCTTTATG
ATTATCCTGGTCCTAGAGAGAGTAAAGTTGATATATATGATTTGAGCATTGATTCTTGGAGAGAACTGGATAGTGAACA
GTTGCCATTGATTTATTGGGTGCCTTGTGCTGAGACATTTTACAAGGAAGCGTTTCATTGGTTTGGAACTATAGATTTG
TCTATGGTGATTCTTTGTTTTGACGTGAGCACTGAGATTTTTCGTAATATGAAAATGCCTCGTACTTTTATTTTTGACA
ACGCGCAATATCCTGGCCTTGTAATCTTAAGCGAGTCTCTTACATTAATATGTTATCCGAACCCAATATCTATTGATCA
TATACAAGAAGTAACGCGCATTTGGGTGATGAAGGAGTACGGAGTAAGCGAGTCTTGGATTTTGAAAGACACAATTAGG
CTTCCTCCTATTGAATATCCGTTAGATATTTGGAAGAATAATTTATTGCTGTTTCAAAGCAAAAGCGGACTTTTGATTT
CCTATAATCTTAAATCCGATGAAGTAAAGGAACTCAAATTAAATGGTTTTCCTGGAAGTATGAGTGTTAAAGTTTACAA
GGAGTCCTTAACATCAATCCCAAGAGGGTTAAAATTA
>N_alata_DD3EF420253
ATGGTTAATGGAAGTATAAAGAAATTGCCCGAAGACTTGGTATTTTGTATGCTATTAAGATGTCCGGTGAAATCTCTCA
TGCGATTCAAATGCATATCTAAAGTTTGGTACCATTTCATACAATCCACCACTTTTATAAATCTTCATCTCAACCGCAC
AACAAGCGTAGAAAACGAATTCATTCTCTTCAAGCACTCCATCAAAGAAGATACAGGAGAATTTAAAAATGTCTTGTCT
TTTCTTTCTGGTCATGATAATGGTGCTCTTAACCCTCTTTTTCCAGATATAGACGTGTCATACATGGCCTCCAATTGTA
GTTGTACTTTTTTCCCACTCATCGGTCCTTGTAATGGTTTGATTGCTTTGACAGATACCATAACCACAATCTTAATTAA
TCCGGCTACTAGAAATTTCAGATTGCTCCCACCTAGTCCTTTTGGTTGTCCCAATGGTTACCATCGTTCTGTTGAAGCG
CTTGGGTTTGGCTTCGACTCGATTGCGAATAACTATAAGGTTGTTAGGATTTCTGAAATTTTTTGGAACCCTGTTTACG
ATTATCCTGGTCCTAGAGAGAGCAAAGTCGATGTTTATGATTTGAGCATTGATTCTTGGAGAGAACTGGACCATGTACA
GGTGCCACTGATTTATTGGTTGCCTTGTTCTGAGACATTGTACAATGAAGTGGTTCATTGGTTTGCATCTACAGACTTG
TCGCTAGTTATTCTTTGTTTTGACATGTGCACTGAGATCTTTCGTAATATTAAAATGCCTGATACTTTTATTTTTGACA
ACGCGGAATTTTATGGCCTGGTAATCCTAAGCGAATCTCTAACATTGATTTGTTACCCGAACCCAATATCTATTAATCC
AATCCAGGAACTGACCCACATTTGGGTGATGAAGGAGTATGGTGTAAGCGAATCTTGGTTTTTGAAAGACACAATTAGA
CCTCCTCCTATTGAACGCCCGTTAGATGTTTGGAAGAATAATATAATACTTTTTGAAAGCAAAAGCGGACTTTTGGTCT
CCTATAAGCTTAATTCCAATGAAGTAGAGGAACTCAAATTACATGGTTGTCCTGGAAGTTTGAGTGTTAAGGTTTACAA
GGAATCCTTAACTTCAATCCCGAGTGGGAGCGAGCACAGTACGAAAGTACAATTTTTT
>P_axillaris_S19_SLF13_AB933049
ATGATGGATGGAACTATGAAGAAATTGCCAGAAGATATGCGGATTTATATATTACTCCGGCTTCCTGTGAAATCTCTTA
CGCGATTTAAATGTGTTACTAAAAGTTGGCATACTCTCATACAATCATTCAATTTCATCAATTTTCATTTCAACCAAAA
ATCTACCACCAAAGATGAATTCATTCTCTTCAGGCGATCCATCAAACATCCGGACGGATTTAGCCATGTTTTGTCTTTT
CTCGTCGATCATGAGGGTAAAGATGATCTAGATCCTATATGTCCTGATATAGATATGCCATATCTGACCACAGGTTTTG
CTAGTAGTACCTCTCATCAATTCACTGGTCCTACCAATGGTTTGATTCTTTTGACAGACTCGTTAAACTTTCTATTATT
AAATCCAGCTACTAGAAGTTATAGGCTGCTCCCACCCAATCCTTTTTGTTGCCCTCGTGGTTTCCTTCGTCTTATTTAC
GGTGTTGGATTTGGCTATGACTCTATTCAAAAGACTTACAAGGTAATTAGAGTTTCACGTGTGTATGGGGATCCTCCAT
ACAATGATCGTAGTGAAATGTCGTGGGAAAGTGAGGTTTATGATTCGAGCACTGATTCTTGGAGACAACTAGCTAATGT
TGATCAAGAGTTGCCTGGGCCTTATATGCACCCTTACTCTGAGTTGTTTTACAAGGGAACCTTTCATTGGTATGCCCAA
GGACACATGCGTTTACTTCTCTGTTTTGATATCAACACTGAAATTTTTCACACAATGCAAGTGCCCAAAACTTGTGCTT

CGAGAGATGAGAAGTGTCATAGCTTAGTAGTCTTTGATGAGTGTCTAACATTTATTTGTTACCCTGACCCAAGGAGAGA
AAGTAGTCCAGTTCAAGAAACGATTGAGATTTGGATAATGCAGGAGTACAGCATAAGCGAGTCTTGGATTAAGAAATAC
ACAATTAGACCTCCTCCTATTGAATCCCCTTTGGCAATATGGAAGGATCGCTTATTGCTGCTTCAAGACAAAAGTGGAG
TCCTGATTGCCTATGATCTTAATTCGGATGAAGTCAAGGAATTCAAATTACATGGTCATCCTGAAAGTTTAAGAGTAAT
AGTTTACAAGGAAAGTTTGACTCCAATTCCTATAGGTAGTACACAAGTTGAACGATTT
>P_axillaris_S19_SLF3_AB568404
ATGACGGCCATGAAGAAATTGCCCATAGATGTGGTGATTAATATGTTATTTAGGCTTCCCGTGAAATCTCTCGCGCGAT
TCAAGTGTGTTACTAAAAGTTGGTACTCCCTCATACAATCAGCCGATTTCATCAATCGTCATCTCAACCGCGCCACTAC
CATCACAGATGAATTTATTCTCTTCAAGCGATCGTTCAAAGAACAGGAAGGATTTAGAAATGTAATGTCCTTTCTGGTC
GGTGGTGTAGGTGAAGACAATCTTGATCCTATTTCTCCTGATGTAGATGTACCATATCTGTCCACCAGTTATAGTTGTA
TCTGTCATCAACTCACTGGTCCTTGCCATGGTTTGATTCTTTTGACAGACTCCACAAACCTTGTCTTATTAAATCCAGC
TACTAGAAATTATAGGCTGCTCCCACCTAGCCCTTTTGGTATACAACGTGGGTTTTATCGTTCTGTTGCCGGTGTAGGA
TTTGGCTATGACTCGGTTCGCAAGACGTATAAGGTGGTCAGAATTTCAGAAGTTTACGGAGAACCTCCATTCAATTGTC
CTAGTGTGATGGAGTGGAAAGGTGAGGTTTATAATTCCAGCACTGATTCTTGGAGAGAACTAGATTGTGTGGATCAAGA
ATTGCCCTGGCCTTACAACTTCGCTTACTCTGAGATTTTTTATGAAGGAGCCTTTCATTGGTATGCCCACAAAAATGTG
GTTTTAATTCTTTGTTTTGATGTCAACACTGAAACTTTTCGCACAATGGAAGTGCCTGAACCTTGTGCTTCGTATGACG
AAAAGTGTCATAGCCTCCTAGTTTTAGATGAGTTTCTAACACTTTTTTGTTACCCTGATCCAAGGAGAGAAAGTAGTCC
AATACAAGAAACAATTGATATTTGGACAATGCAAGAATACAGGGTAAACGAGTCTTGGATTAAGAAACACACAATTAAA
TCTCCTCCTATTGAATCGCCATTGGCAATTTGGAAGGATCGCTTATTGCTTTTTCAAGACAAAAGTGGAATTCTGATAT
CTTATGATCTTAATTCCGATGAAGTCAAGGAATTCAAATTAGATGGTTATCCTGCAACTTTGAGAGTTATAATTTACAA
GGAAAGCTTAACTCCAATTCCTAAGGGTAGTACACAAGTTCAAAATTTT
>P_hybrida_S11_SLF3_AB933014
ATGAAGAAATTGCCTGAAGATGTGGTGATTTATATACTTTTTAGGCTTCCCGTGAAATCTCTTACACGATTCAAACGCG
TTACAAAACGTTGGTACTCTCTCATACGATCATCTAGTTTCATAAATCTTCATCTCACCGGTGCCACTAATACCATCAA
AGATGAATTAATTCTCTTCAAGCGATCGTTCAAAAAACGGGAGGGATTTAAAAATGTGTTGTCTTTTCTGCTCGGTAGT
AATGCTGAAGATGATCTTGATCCTATTTCTTCTGGTCAAGATGTGCCATATCTGTCCACCCGTTATAGTAGCATCTCTC
ATCAACTCACTGGTCCTTGCCATGGTTTGATTGTTTTGACAGACTCCACAAATTTTGTCTTATTAAATCCAGCTACTAG
AAATTACAGGCTGCTCCCACCCAGTCCCTTTGTATGCCCACGTGGTTTATATCGTTCTATTGGCGGTGTTGGATTTGGC
TATGACTTCATTCAGAAGAACTACAAGGTGGTTAGAATTTCAGAAGTGTACGGAGAACCTCCATTTAATTGTCCAAGTG
TGATGGAGTGGAAAGGTGAGGTTTATGATTCGAGCACTGATTCTTGGAGAGAACTAGCTTATGTGGATCAAGAGTTGCC
CTGGCCTTATAATTTCCCTTATTCTGAGATGTTTTACAATGGAGCCTTCCATTGGAATGCCCATAGAAATATGGTGGTA
ATACTTTGTTTTGACATCAGCACTGAAATATTTCGCAGTATGCAAGTTCCTGAATCTTGTGCGTCATATGACGAAAAGC
GCCATAGCCTTTTAATCTTGGATGACTCTCTAACAATTATTTGTTACCCTGACCCAAGAAGGGTGAGTAGTCCGGTACA
AGATACAATTGATATTTGGACAATGAATGAGTACAATGTAAACGACTCTTGGATTAAGAAATACACCATTAGATCTCCT
CCCATTGAATCCCCATTGGCAGTTTGGAAGGATTGCCGATTGCTTCTTCAAAATAAATGTGGATTTCTGATCTCCTATG
ATTTTTATTCCAATGAAGTGAAGGAATTTAAATTACATGGTTATCCCGGAAGTTTGAGAGTTATAGTTTACAAGGAATG
TATGACTCCGATTCCTAGAGGTAGTACACGAGTTCAAAAATTA
>N_alata_DD2_EF420252
ATGGTGGATGGAATCATGAAAGAATTGCCCGAAGATCTGGTGATATATGTAATTCTAATGCTTCCAGTGAAATCTCTAT
TGCGATTGAAAAGCTCCTGTATAACTTTTTGCAATATCATAAAGTCATCCACCTTCATTAATCTTCATCTCAATCGTAC
AACCAACGGCAAGGATGAATTGATTCTCTTCAAGCGTTCCTTCAAACAAGAAGAACCCAACCTTCATAAAAATGTCTTG
TCTTTTCTTCTCAGTGAAGATACTTTTAATCTTAAGCCTATTTCTCCAGATGTAGAGATTCCACATCTTACCAACACTA
ATGCCAGTGTTTTTCATCAACTCATTGGCCCTTGCAATGGTTTGATTGCCTTGACAGATTCACTGACCACTATCTTGTT
TAATCCAACAACTAGAATTTACAGACTAATCCCACCTTGCCCTTTTGGTACTCCACCGGGTTTCAGACGTTCCATTAGT
GGTATTGGGTTTGGCTTTGACTCGATCGCAAATGATTACAAGTTTGTTAGGATATCAGAGGTTTACAAGGACCCTTGTG
AGAAAGACATGAAAGTTGAGGTTTTTGACATGTGCACTGATACTTGGAGAGAACTGCATGGTCAACAACTGCCTATGGC
GTTTTGGACGCCTTGTTCAGAGATAATTTACAATTGTGCCTTTCATTGGTTTGCCACTGCAGACGATGTAGTAATTCTC
TGTTTTGACATGTGCGCCGAGAAATTTTACAATATGGAAACGCCTGGTACATGTCATTGGTTTGACGGTAAGTGTTATG
GACTTGTGATTTTATATAAGTCTCTGACGTTGATTTGCTACCCTGATCCAATGTCGACTGATCCAACGGAAGATTTGAT
GGATATATGGATAATGAAGGAGTACGGTAAAAAGGAGTCGTGGATAAAGAAATGCTCAATTGGACCTCTTCCTATTGAA
TCCCCATTAGCAGTTTGGAAAGATGATTTATTGCTTTTTCAAACCAAAAGTGGATATTTGATTGCCTATGATCTTAATT
CTGATGAAGTGAAGGAATTCAATTCACATGGTTTTCCCACGAGTTTGAGAGTTATAGTTTACAAGGAAAGCTTGACCCC
GATTCCAAGAAATGGGGATGGAACTGTAGTTCAACTATTT
>P_axillaris_S19_FBX2_AB933054
ATGTTGGATGGGACCATGAAGGAATTGCCCCATGATGTAATGATTTATATTCTTGTTATGCTCCCGGTAAAATCTCTAC
TACGATTCAAATGCAGCTGTAAAACTTTTTGCAATATCATAAAATCAGCCACTTTCATTAATCTTCATCTAAATCATAC
AACCAACTTCAAGGATGAATTGGTTCTCCTCAAGCGTTCCTTCAAAACAGATGAATACAACTTTTATAAATCTATCTTA
TCTTTTCTTTCTAGTAAAGAAGATTATGATTTTACGCCTATTTCTCCAGATGTAGAAATCCCACATTTGACCACCACTT

CTGCCTGTGTTTTTCATCAACTCATTGGTCCTTGCAATGGTTTGATTGCCTTGACAGATTCCCTGACCACTATCTTGTT
TAATCCAACAACTCGATATTACAGATTAATTCCACCTTGCCCATTTGGTATTCCGCGTGGTTTCAGACGTTCCATTAGC
GGTTTTGGCTTCGGTTTTGATTCAAATGCCAATGATTACAAGGTTGTTAGAATATCAGAAGTATACAAGTATCATTATG
ATAAAGATATGAAAGTTGACATTTATGACGTTTCCGTTGATTCTTGGAGAGAACTGAATCTTTTAGGTCAAAAGTTGCC
TATTGTGCTTTGGTTTCCTTGTTCTGAGATATTGTACAAACGAAACGTTCATTGGTTTGCCGTTGCAGATGATGTAGTA
ATTCTCTGTTTTGACTTCAGCACTGAACTATTTAAAAATATTGAAATGCCTAATGCACATGATATCGATGGAATGTCTT
ATGGCCTTGTGATTTTATATAAGTTTCTGACGTTGATTTGTTACCATTATCCAATGTTTACTGAGCCAACAGAAGATTT
GGTGGATATTTGGATAATGAAAGAGTACGGTCAAAAGGAGTCTTGGATAAAAAGATTCTCAGTTAATCTTCTTCCTATT
GAATCCCCGTTGGCAGTTTGGAAGGATGAATTATTGCTTCTTCAAACCAGAAGTGGACAATTGTTCACCTATGATCTTA
ATTCTGATGAAGTCAAGGAGCTTAATTTACATGGTTGTCCAGAAAGTCTGAGAGTAGTAGTTTATAAGGAAAGCTTGAC
CCTGATTCCAAGAAATGACGGTGGTGCTGAAGTTCAACCATTT
>P_axillaris_S19_FBX1_AB933053
ATGTTGGATGGGACCAGGAAGGAATTGCCCCGTGATGTAGTGATTTATATTCTTGTAATGCTCCCGGTAAAATCTCTAC
TGCGATTTAAATGCAGCTGTAAAACTTTTCGTAATATCATAAAATCAGCCACTTTCATTAGTCTTCATCTAAATCATAC
AACCAACTTCAAGGATGAATTGGTTCTCCTCAAGCGTTCCTTCAAAACAGATGAGTACAACTTTTATAAATCTATCTTA
TCTTTTCTTTTCAGTAAAGATGATTATGATTTTAAGCCCATTTCTCCAGATGTAGAAATCCCACATTTGACCACCACTT
CTGCCTGTGTTTTTCATCGACTCATTGGTCCTTGCAATGGTTTGATTGCCTTGACAGATTCCCTGACCACTATCTTGTT
TAATCCAACAACTCGATATTACAGATTAATCCCACCTTGCCCATTTGGTATTCCACGTGGTTTCAGACGTTCCATTAGC
GGTATTGGGTTTGGCTTTGATTCGGATGCAAATGATTACAAGGTTATTAGGCTATCAGAAGTTTACAAGGAACCTTGTG
ACAAAGATATGAAAGTTGATATTTATGACTTTTCCGTTGATTCTTGGAGAGAACTTTTAGGTCAAGATGTGCCATTTGT
ATTTTGGTTGCCATGTGCTGAGATATTATACAAAAGAAACGTTCATTGGTTTGCGTTTGCAGACGATGTAGTAATTCTT
TGTTTTGACATGAGCACCGAAAAGTTTAACAATATGAGTATGCCTGATCCATGTCATTTTTATGATGGAAAGTGTTATG
GCCTGGTGATTTTGTGTAAGTCGCTGACGCTGATTTGTTACCCTGACCCAATGTCAAGTAATCCAACAGAATATTTGAC
AGACATTTGGATAATGAAGAAATACGGTGAAAAGGAGTCTTGGAAAAAGAGATGCTCAATTAGACTTCTTCCTATTGAA
TCCCCACTAGCCGTTTGGAAGGATGAGATATTGCTTCTTCAGAGCAAAATGGGACATTTGATTGCCTATGATCTTAATT
CGAACAACGTCCGGGAATTAGATTTACATGGTTATCCCGAGAGTTTGAGAATTATAATTTACAGGGAAAGCTTGACCGC
GATTCCAAGAAATAAGGATTGCATAGAACTTCAAAATTTT
>P_axillaris_S17_SLF9_AB933029
ATGTTGGATGGGAGCATGAAGGAATTGCCCCAAGATGTAGTGATTTATATACTTGTAATGCTCCCGGTAAAATCTCTTC
TACGATTCAAATGCAGCTGTAAAACTTTTTGCAATATCATAAAATCATCCACTTTCATTAATCTTCATCTGAATCATAC
GACCAACGTCAAGGATGAACTGGTTCTCCTCAAGCGTTCCTTCAAAACAGATGAATACAACTTTTATAAATCTATGTTA
TCTTTTCTTTCCAGTAAAGAAGATTATGATTTTAAGCTCATTTCTCCAGATGTAGAAATTCCACATTTGACCACCACTT
CTGCCTGTGTTTTTCATCAACTCATTGGTCCTTGCAATGGTTTGATTGCCTTGACAGATTCCCTGACCACTATCGTGTT
TAATCCAGCAACGCGAAAGTACAGACTAATCCCACCGTGCCCATTTGGTATTCCGCGTGGTTTTAGACGTTCCATTAGC
GGTATTGGGTTTGGCTTTGATTCGGATGCAAATGACTACAAGGTTGTTAGGCTATCAGAAGTTTACAAGGAACCTTGTG
ACAAAGAAATGAAAGTTGATATTTATGACTTTTCCGTTGATTCTTGGAGAGAACTTTTAGGTCAAGAGGTGCCTATTGT
GTATTGGTTGCCATGTGCTGAGATATTATACAGAAGAAACTTTCATTGGTTTGCATTTGCAGACGATGTAGTAATTCTT
TGTTTTGACATGAACACCGAAAAATTTCACAATATGGGAATGCCAGATGCATGTCATTTCGATGATGGAAAGTGTTATG
CCCTGGTGATTTTATGTAAGTGCATGACGCTGATTTGTTACCCTGATCCAATGCCAAGTAGTCCAACAGAAAAAATTGAC
AGATATTTGGATAATGAAGGAATACGGTGAAAAGGAGTCTTGGATAAAGAGATGCTCAATTAGACTTCTTCCTGAATCC
CCATTAGCAGTTTGGAAGGATGAGATATTGCTTCTTCAAAGCAAAATGGGACACTTGGTTGCCTATGATCATAATTCTG
ATGAAGTGAAGGAATTAGATTTGCATGGTCTTCCGACGAGTCTGAGAGTTATAATTTACAGGGAAAGCTTGACCCTGAT
TCCAAGAAGTAAGGATAGCATAGACCTTGAACAATTT
>P_axillaris_S17_SLF9_AB933030
ATGTTGGATGGGACCATGAAGGAATTGCCCCAAGATGTAGTGATTTATATACTTGTAATGCTCCCGGTAAAATCTCTAC
TACGATTCAAATGCAGCTGTAAAACTTTTTGCAATATCATAAAATCATCCACTTTCATTAATCTTCATCTAAATCATAC
GACCAACGTTAAGGATGAATTGGTTCTCCTCAAGCGTTCCTTCAAAACAGATGAATACAACTTTTATAAATCTATCTTA
TCTTTTCTTTCCAGTAAAGAAGATTACGATTTTAAGCCCATTTCTCCAGATGTAGAAATTCCACATTTGACCACCACTT
CTGCCTGTGTTTTTCATCAACTCATTGGTCCTTGCAATGGTTTGATTGCCTTGACAGATTCCCTGACCACTATCGTGTT
TAATCCAGCAACTCGAAAGTACAGATTAATCCCACCATGCCCATTTGGTATTCCGCGCGGTTTCAGACGTTCCATTAGC
GGTATTGGGTTTGGCTTTGATTCGGATGCAAATGATTACAAGGTTGTTAGGCTATCAGAAGTTTACAAGGAACCTTGTG
ACAAACAAATGAAAGCTGATATTTATGACTTTTCCGTTGATTCTTGGAGAGAAATTTTAGGTCAAGAGGTGCCTATTGT
GTATTGGTTGCCATGTGCTGAGATATTATACAAAAGAAACTTTCATTGGTTTGCGTTTGCAGACGATGTAGTAATTCTT
TGTTTTGACATGAACACGGAAATATTTCACAATATGGGAATGCCAGATGCATGTCATTTCGATGATGGAAAGTGTTATG
GCCTGGTGATTTTATGTAAGTGCATGACGCTGATTTGTTACCCTGATCCAATGCCAAGTAGTCCAACAGAAAAAATTGAC
AGATATTTGGATAATGAAGGAATACGGTGTAAAGGAGTCTTGGATAAAGAGATGCTCAATTAGACTTCTTCCTGAATCC
CCATTAGCAGTTTGGAAGGATGGGATATTGCTTCTTCAAAGCAAAATGGGACATTTGATTGCCTATGATCATAATTCTG

ATGAAGTTACGGAATTAGATTTGCATGGTCTTCCCACGAGTTTGAGAGTTATAATTTACAGGGAAAGCTTGACCCCGAT
TCCAAGAAGTAAGAATAGCACAGAACTTGAACAATTTCAGAAGTGGGATAACTCTTCAACTCTAAAT
>P_hybrida_Sm_SLF9_A_AB933097
ATGAAGGAATTGCCCCAAGATGTAGTGATTTATATACTTGTAATGCTCCCGGTAAAATCTCTACTACGATTCAAATGCA
CATGCAAAACATTTTGCAATATCATAAACTCGTCCACTTTCGTTAATCTTCATCTAAATCATACGACCAATGCCAAGGA
TGAATTGGTTCTCCTTAAACGTTCCTTCAAAACAGATGAATACAACTTTTATAAATCTATCTTATCTTTTCTTTACAGT
AAAGAAGATTATGATTTTACGCCCATTTCTCCAGATGTAGAAATTCCACATTTGACCACCACTTCTGCCTGTGTTTTTC
ATCAACTCATTGGTCCTTGCAATGGTTTGATTGCCTTGACAGATTCCCTGACTACTATCGTGTTTAATCCAGCAACTCG
AAAGTACAGATTAATCCCACCATGCCCATTTAGTATTCCGCGTGGTTTCAGACGTTCTATTAGCGGTATTGGGTTTGGC
TTTGATTCGGAGGCAAATGATTACAAGGTTGTTAGGCTATCAGAAGTTTATAAGGAACCTTGTGACAAAGAAATGAAAG
TTGATATTTATGACTTTTCCGTTGATTCTTGGAGAGAACTTTTAGGTCAAGAGGTGCCTATTGTATATTGGTTGCCATG
TGCTGAGATATTATATAAAAGAAACTTTCATTGGTTTGCATTTGCAGACGATGTAGTAATTCTTTGTTTTGACATGAAC
ACCGAAAAAATTTCACAATCTAGGAATGCCAGATGCATGCAATTTCAATGACGGAAAGTGTTATGGCCTGGTGATTTTAT
GTAAGTGCATGACCCTGATTTGTTACCCTGATCCAATGCCAAGTAGTCCAACAGAAAAACTGACTGATATTTGGATAAT
GAAGGAATACGGTGAAAAGGAGTCTTGGATAAAGAGATGCTCAATTAGACTTCTGCCTGAATCCCCATTAGCAGTTTGG
AAGGATGAGATATTGCTTCTTCAAAGCAAAATGGGACATTTGATTGCCTATGATCATAATTCTGATGAAGTTAAGGAAT
TAGATTTGCATGGTCTTCCCACGAGTTTGAGAGTTATAATTTACAGGGAAAGCTTGACCCCGATTCCAAGAAGTAAGGA
TAGCATAGAACTTGAACAATTT
>P_hybrida_Sm_SLF9_B_AB933098
ATGAACTATACAAAAATGTTGGATGGGACCATGAAGGAGTTGCCCCAAGGTGTAGTGATTTATATACTTGTAATGCTCC
CGGTAAAATCTTTACTACGATTGAAATGCACCTGCAAAACATTTTGCAATATCATAAAATCGTCCACTTTCATTAATCT
TCATCTAAATCATACGACCAATGTCAAGGATGAATTGGTTCTCCTCAAACTATCCTTCAAAACAGATGAATACAACTTT
TATAAATCTATCTTATCTTTTCTTTACAGTAATGAAGATTATGATTTTACGCCCATTTCTCCAGATGTAGAAATTCCAC
ATCTGACCACCACTTCTGCCTGTGTTTTTCATCAACTCATTGGTCCGTGCAATGGTTTGATTGCCTTGACAGATTCCCT
GACCACTATCGTGTTTAATCCAGCAACTCGAAAGTACAGATTAATCCCACCATGCCCATTTAGTATTCCGCGTGGTTTC
AGACGTTCTATTAGCGGTATTGGGTTTGGCTTTGATTCGGATGCAAATGATTACAAGGTTGTTAGGCTATCAGAAGTTT
ACAAGGAACCTTGTGACAAAGAAATGAAAGTTGATATTTATGACTTTTCCGTTGATTCTTGGAGAGAACTTTTAGGTCA
AGAGGTGCCTATTGTGTATTGGTTGCCATGTGCTGAGATATTATACAAAAGAAACTTTCATTGGTTTGCATTTGCAGAC
GATGTAGTAATTCTTTGTTTTGACATGAACACCGAAAAAATTTCACAATATGGGAATGCCAGATGCATGTAATTACAATG
ATGGAAAGTGTTATGGCCTGGTGATTTTATGCAAGTGCATGACGCTAATTTGTTACCCTGATCCAATGCCAAGTAGTCC
AACAGAAAAAATTGACAGATATTTGGATAATGAAGGAATACGGTGAAAAGGAGTCTTGGATAAAGAGATGCTCAATTAAA
CTTCTTCCTGAATCCCCATTAGCAGTTTGGAAGGATGAGATATTGCTTCTTCAAAGCAAAATGGGACATTTGATTGCCT
ATGATCATAATTCTGATGAAGTCAAGGAATTAGATTTACATGGTCTTCCCACAAGTTTGAGAGTTATAATTTACAGGGA
AAGCTTGACCGCAATTCCAAAAAGTAAGGATAGCATAGAACTTGAACAATTT
>P_axillaris_S19_SLF10_AB933045
ATGTTGGATTGGACCATGAAGGAGTTGCCCCAAGATGTTGTGATTTATATATTTGTAATGCTCCCGGTAAAATCTCTGC
TACGATTCAAATGCACCTGTAAAACATTTTGCCATATCATAAAATCATCCACTTTCATTAATCTTCATCTAAATCATAC
GACCAACTTCAATGATGAATTGGTTCTCCTTAAGCGTTCCTTCGAAACAGATGAATACAACTTTTATAAATCTATCTTA
TCTTTTCTTTTCGCTAAAGAAGATTATGATTTTAAGCCCATTTCTCCAGATGTAGAAATTCCACATTTGACCACCACTG
CTGCCTGTATTTGTCATCGACTCATTGGTCCTTGCAATGGTTTGATTGTCTTGACAGATTCCCTTACCACTATCGTATT
TAATCCAGCAACTCTAAAGTACAGACTAATCCCACCATGCCCATTTGGTATCCCGCGTGGTTTCAGACGTTCCATCAGC
GGTATTGGTTTTGGCTTTGATTCGGATGCAAATAATTACAAGGTTGTTAGGCTATCAGAAGTTTACAAGGAACCTTGTG
ACAAAGAAATGAAAGTTGATATTTATGACTTTTCCGTTGATTCATGGAGAGAACTTTTAGGTCAAGATGTGCCTTTTGT
CTTTTGGTTTCCATGTGCTGAGATATTATACAAAAGAAACTTTCATTGGTTTGCATTTGCAGACGATGTAGTAATTCTT
TATTTTGACATGAACACCGAAAAATTTCACAATATGGGAATGCCAGATGCATGTCATTTCGCTGATGGAAAGTCTTATG
GCCTGGTGATTTTATTTAAGTGCATGACGCTGATTTGTTACCCTGATCCAATGCCTAGTAGTCCAACAGAAAAAATTGAC
AGATATTTGGATAATGAAGGAATACGGTGAAAAGGAGTCTTGGATAAAAAGATGCTCAATTAGACTTCTTCCTGAATCC
CCATTAGCAGTTTGGAAGGATGAGATATTGCTTCTACACAGCAAAATGGGACATTTGATAGCCTATGATCTTAATTCGA
ACGAAGTCCAGGAATTAGATTTACATGGTTATCCCGAGAGTTTCAGAATTATAATATACAGGGAAAGTTTGACCGCGAT
TCCAAGAAATAATGATTGCATAGAACTTCAAAATTTTAGATGTAGC
>P_axillaris_S19_SLF10_AB933046
ATGCACAATACAATACAAAAGATGTGGGATAAGACCATGAAGGAATTGCCAAAAGATGTAGTGATTTATATACTTGTAA
TGCTCCCGGTAAAATCTCTACTACGACTCAAATGCACCTGTAAAACTTTTTGCCATATCATAAAATCATCCACTTTCAT
TAAGCTTCATTTAAATCATACGACAACCTTCAAGGATGAATTGGTTCTCCTTAAGCGTTCCTTCAAAACAGATGAATAC
AACTTTTATAAATCTATCTTATCTTTTCTTTTCAGTAAAGAAGATTATGATTTTAAGCCCATTTCTCCAGATGTAGAAA
TTCCACATTTGACCACCACTTCTGCCTGTACTTTTCATCGACTCATTGGTCCTTGCAATGGTTTGATTGTCTTGACAGA
TTCCCTGACCACTATCCTGCTTAATCCAGCAACTCGAAAGTACAGACTAATCCCACCATGCCCATTTGGTATCCCGCGT
GGTTTCAGACGTTCCATTAGCGGTATTGGGTTTGGCTTTGATTCGGATGCAAATGATTACAAGGTTGTTAGGCTATCAG

AAGTTTACAAGGAACCCTGTGACAAAGAAATGAAAGTTGATATTTATGACTTTTCCGTTGATTCTTGGAGAGAACTTTT
AGGTCAAGATGTGCCTTTTGTATTTTGGTTTTCATGTGCTGAGATATTATACAAAAGAAACTTTCATTGGTTTGCATTT
GCAGACGATGTAGTAATTCTTTGTTTTGACATGAACACCGAAAAATTTCACAATATGGGAATGCCAGATGCATGCCATT
TCGATGATGGGAAGTGTTATGGCCTGGTGATTTTATTTAAGTGCATGACGCTGATTTGTTACCCTGATCCAATGCCAAG
TAGTCCAACAGAAAAATTCACGGATATTTGGATAATGAAGGAATACGGTGAAAAGGAGTCTTGGATAAAGAGATGCTCA
ATTAGACTTCTTCCTGAATCCCCATTAGCAGTTTGGAAGGATGAGATATTGCTTCTACAGAGCAAAATGGGACATTTGA
TTGCCTATGATCTTAATTCGAACGAAGTCCAGGAATTAGATTTAAATGGTTATCCCGATAGTTTGAGAATTATAATTTA
CAAGGAAAGCTTGACCGCGATTCCAAGAAATAAGGATTGCATAGAACTTCAAAAATTTCAGATGATT
>P_hybrida_Sm_SLF15_AB933105
ATGGGAGATGAAATATTGGAAAAATTGCCTAAAGATATCGTGATTTATATATTTTTAATGGTTCCAGTAAAATCTCTCG
TACGATTCAAATGTGTCTCAAAAGATTGGTATACTCTCATACAATCTTCAACATTTATCGGTCTCCATTTTAATCGTAC
CACCACCACCACCAAGGATGAATATATGCTCGTCAAGCGCTCTTTTAAAGAAGAATCGAATCGTTTCAGAAGTGTAATG
TCTTTTCTCTCTGGTGGTCTTGATGATGATGATGATCTTTACCCTGTTTCTCCGGATCTAGATGTGCCATTTCTGACTA
CCACTAATTCTTGTACTTTCCATAGAATCATGGGCCCATGCAATGGTTTGATTGTTTTAACAGATAAGATAACTACCGT
ACTATTCAATCCAGCTACTAGAAGTTATAGGCTACTCCAACCTGGCCGTTTTGGTTGTCCTGTGGGTTTTCATCGTTCC
ATTAATGGTGTCGGGTTTGGCTTTGACTCTGTTGCGAATAGCTACAAGATTGTTAGAATTGCAGAAGTTAATGGGGAGC
CTCCTTTTTATTGTTATACCATGAGAGAGTGGAAAGTTGAAATTTATGAATCTAGCGTTGATGCATGGAGAGAACAAGA
TCAAGTGTTTCGACAATTGCCCAATGTATTTTGGTATCCTTGTTTTGAGATGTTTTACAAAGGGGCCTCTCATTGGTTT
GCCCATGCAAATACAATAGTAATTCTTTGTTTTGACATAATCACTGAAACTTTTCGCAGTATAAAGTTTCCTAATACTT
GTCATTTCCAAGACGAGAATTGCTATAGCCTCCTAATTTTAAATGATTCTCTAACTTTGATTTGTTACCCCTATCCAGA
GAAAGTAGTAGAATATGAAAAAGATTTCATGGAAATTTGGATAATGATGGAATACGGTGTGGACGAGTCTTGGATTAAG
AAATATTCAATTACACCTCTTTCTATTGAAACACCATTAGCCGTTTGGAAGGATCATTTATTGCTCCTTGAAAGCAGAA
GTGGAAGTTTGATTTCCTACGATCTTAATTCTGGTGAAGTCAAGGAACTTAACTTACATTGTTGGCCAACAAGTTTTAG
AATTGCAATTTACCAGGAAAGCTTGACTTTAATTCCAGAAGAGAGAGAGCATAGTACCAAATGCCCAAAAAATTTTAGAG
AGT
>P_hybrida_S0m_SLF7_AB933078
ATGGCAGAAGGAATACTTAAAGGGTTGTTTGGAGATGTTATGATTTATATACTTTTGAGGCTTCCGCTGAAAACGCTGT
TGCGATTCAAATGCATCTCTAAAACATTGTACAATATCATACAATCATCAACTTTCATCAATCTTCATCTCAACCGTAC
CACTACCACCAATGATGAATTCATTCTCTTCAACCGCTCCATTAAAGAAGCACATAACGAATTTAAAAGTGTCATGTCT
TTTTATGCTTGTAGTCACGATAATTATGATATTCATTCTATTTCTCCAGATCTAGATGTTCCAAACATGAAGCCCTCTA
TTTCTAGTGTTTCGCATAGACTAATAGGTCCTTGTCACGGTTTGATTGTTTTAACAGATACGGTAGAAACGATCTTACT
TAATCCAGCTACTAGAAATTATAGGATACTCCGACCTAGTCCTTTTGATTGTCCAATGGGATTCTGTCGTTCCATTGCG
GGTGTTGGGTTTGGCTTTGACTCGATTGCGAATGACTACAAGATTGTACGGGTTTTAGAAGACTATGGCGATCCTCCGT
TCTATGATTTTGCGTTGAGAAAGTGGAAGATCGATGTGTATGAATTGACTATTGATTCCTGGAGAGAACTAGATTATAT
GGATCTAGAGTTGCCTCATGTCCACAGGTATCCTTGTTCCGAGATGTTTTACAACGGGACCACTCATTGGTTTGGAAGA
ACGGAAACAGTGGTTATTCTTTGTTTTGACATGAGCACAGAGACTTTTCGCAATATGAAAATGCCGGATGCGTGTCATT
TCAAAGACAGGAAGAGTTATGGCCTCGTTGTTTTAAATGACTCTCTAACACTGATCTGTTACCGCCATCCTGGGTGTGT
AATTGATCCTGCAAAAGACTTCATGGAAATTTGGACAATGAAGGAGTATGGTGCAGGTGAGTCTTGGATTAAAAACTAC
ACGATTGCACCTCTTTCTATTAAATCCCCATTAGCAGTTTGGAAAAACCATTTCCTGCTACTTGAGTACCATCGTAGTG
GAGTTCTGTTTTCCTATGATCTTAATTCTGATGAAGTCAAGGAACTCAATTTACATGGCTGGCCTCAGAGTTTGAGGGT
ATCAATTTATAAGGAAAGCTTGACTTTAATTCCTAAAGGAAGCGAGCATAGTACTCAAGTTCAAAATTTT
>N_alata_DD9_EF420259
ATGATACCGAAGATGGGAGATGGAACCGTGGAAAAATTGCCTAAAGATGTTGTGATTTATATAATTTTGAGGCTTCAGG
TAAAATCTCTCATTCGATTCAAATGTGTCTCTAAGACTTGGTACATTCTAATACAATCATCAACATTCATCTATCTTCA
TCTCAGTCATACTACCACCAGCAATGATGAATTGGTTCTCTTCAAGCGTTCCTACAAAGAAGAACCAAACCGATTCAAA
AGTGTATTGTCATTTCTCTCAAGTGGTCATGATGATGATGATCTTCACCCTGTTTCTCCAGATCTAGACATGCAATATA
TGACCACCAGTAGTGCTTGTACTTGCCATAGGATTATAGGCCCTTGCAATGGTTTAATTTTTTTAACGGATAAGCTAAA
TAATGTTCTGTTTAACCCAACTACTAGAAACTACAGGTTACTCACACCCAGTCCTTTTGGCTGTCCACTGGGTTTCCAT
CGTTCCATTAATTGTGTTGGGTTTGGTTTTGACTTGATTGTGAATGACTATAAGATTGTTCGGATTTCAGAAGTTCGCG
GGGAGCCTCCTTTCTATTGTGATTCTATGAGAGAGTGGAAAGTTGAGGTTTATGAATTGAGGACTGATTCTTGGAGGGA
ATTAGATCAAGTAAATCTACAGTTGCCCTATGTGCATTGGAATCCTTGTTCTGATATGTTTTACAGTGGGGCCTCTCAT
TGGTTCGGAAATGCTAATACGGTGGTAATTCTTTGTTTTGACTTAAGCACCGAGACTTTTCGAAATATGAAAATGCCTA
ATACTTGCCATTCCAGAGATGAAAAGTGTTACGGCCTCGTAGTCTTAAATGAATATTTGACACTGATTTGTTACCCCTA
TCCAGGTAAGGTAATCGATCCTTTAAAAGATTTCATGGACATTTGGATGATGAAGGATTACGGTGTAAACGAGTCATGG
ATTAAGAAATACACAATTACCCCTCTTTCTATTGAATCCCCATTAGCGGTTTGGAAGGATCATTTACTGCTTCTTCAAA
GCAGAAAAGGATTTTTGGTTTCCTATGATCTTAAATCTAAAGAAGTCAAAGAATTCAATTTCCATGGTTGGCCAAAAAG
TTTGAGAGCAACAGTTTACAAGGAAAGTTTGACTTTACTTCCGAAAGAAAGCGAGCATAATAAACAAGTTCAATTT
>P_hybrida_S5_FBX2_AB932974

ATGGCAGATGGATTTGTCATTAAACTCCCCAAGGATGTGATGATTTATATACTTTTAAGGCTTCCAGTAAAATCCCTTT
TACGACTAAAATGTGTATCTGAAAATTGGTACACTCTCATACGATCATCGACATTCATCAATCTTCATCTATATCATCC
GATCAAGGCCAACTGTGAATTCATTCTTTTCAAGCGCTCCTACAACGAAGAACCCAACCAATTTAGAAGTATCATGTCT
TTTCTCTCTAGTTTTCAGGATAATAATTGCCTTCACCATGTTTCTCCCGATATAGAGGTGCCACATTTGACTACCACTA
CTTCTTGTATTTTTCAAAGACTCATAGGTCCTTGCCATGGTTTAATTGCTTTAGTGGATAACATATCGGTAGTGTTATT
TAATCCAGCTACTAGAAGTTATAGACTACTCAAACCAAGTCCTTTTGGTTGTCCATTCGGTTTCCGTCGTTCCATAAAT
GGTATTGCATTTGGTTTTGACTCGATTACAAATGAATACAAGATTGTTAGGTTAGCAGAAGTTCGCGGGCAACCTCCTT
TTTATTGTTTAAGTTTGAGAGAGTTGAGAGTGGAGGTTTATGAATTGAGTATTGATTCATGGAGGGAGGTGGAACATGT
GGATCAACAGTTGCCTTTTGTGCGTTTGTATCCTTGCTCAGAGTTATTCTACAAAGGGTCCTCTCATTGGTTTGCAAAC
ACAAATACAGTTGTAATTCTTTGTTTTGACATGACCACAGAGACGTTCCGCAATATTAAAATGCCTAATACTTGCCATT
TCGAAGACAGGAAGTGCTACGGCCTGGTAATCCTGAATGAGTATCTAACATTGATTTGTTACCCTTATCCAGGTTGTGA
AATTAATCCTGAAATGGATTTTATGGAGATTTGGGTAATGAATGACTACGGTGTAAACGAGTCTTGGATTAAGAAAAGC
ACACTTACACCTCTTCCAATTGAATCGCCACTGGCAATTTGGAAGGATCATTTATTGATCCTTCAAACCATAAGTGGAC
ATTTGATTTCCTGTAATCTTAATTCTAATGAAGTCAAGCAATTCAATTTACATGGTTGGCCCAAAAGCTTGAGAGTGAC
AATTTACAAGGAAAGCCTGCATAATACACGTTCAACAACTTTGGAGAGGATTATCTCTCTAACT
>P_inflata_unknown_haplotype
AAGAAATTCAAGATGGCAGATGGAATTGTGATAAAATTGCCTAAAGATGTGGTGACGTATATATTTTTGACGTTTCCAG
TAAAATCTCTCCTACGATTGAAATGTGTTTCCAGGAATTTGCATACATTCATACAATCATCGGCATTCATTAATCTTCA
TCTCAATCGTACCAGCATCATCAACGAGGAATTCATTCTTTTCAAACGCTCATTAAAAGAAGAACCCGATCGATTTAGA
AATATCATGTCTTTTCTCTCTAGTGGTCATGATAATTATGACCTTCACCATGTTTCTCCAGATCTAGATGTGCCATATC
TGACTACAACAGGAGCTTGTACATCTCACAGATTCATGGGTCCTTGCCATGGCTTAATTGTCTTCACGGATGGTGAAGA
AACAGAAGTATTATTTAATCCATCAACTAGAAATTATAGGCTACTCACACCTAGCCCGTTTGATAGCCCATTGGGTTTC
CATCGTAGCATTGACGGTATTGCATTCGGTTTTGATTCGATTGGAAATGATTACAAGATTGTGAGGATCGCAGAACTTC
ATGGAGAACCACCTTTTAATTGTTTTAGTACGAGAGAGTGGAGAGTTGAAGTTTTCGAAATGAGTATTGATTCATGGAG
AGAGGTAGAAAATGTGGATCAACAACTGCGTTATGTGCATTGGTATCCCAGTGCTGATTTGTTCTACAAAGGGGCCTCT
CATTGGTTTGGCAACGAAAATAGAGTCCATGTAATTGTTTGTTTTGACATGTGTACAGAGATATTTCGCACTTTTAAAA
TGCCTAGTACTTGCCATTACAAAGACAAAAATTTTTACTGCCTTGTAGTCTTGAATAAGTGTCTAACGTTGATTTGTTA
CCCCTATCTAGGTTATGAAATTGATCCTGCAATTGATTTCATGGAGATTTGGATAATGAAGGAGTATGGGATATACGAG
TCTTGGAGTAAAACATACAGAATCAGACCTCTTGCAATTGAATCGCCATTGGCAATTTGGAAGGATCATTTATTGCTCC
TTCAGAGCATAAGTGGGTATTTGATTTCCTATGATCTGAATTCTGGTGAAGTCAAGGAATTCGAATTGAATGGTTGGCC
CGACAGTTTGCGAGTAACAGTTTACAAGGAAAGCTTGGCTTTAATTCCAAATTCCAAAAGACCGCGAGCA
>P_hybrida_S10_SLF17_AB933012
ATGGCAGATGGAATTGTGATAAAATTGCCTAAAGATGTGGTGACGTATATATTTTTGACGTTTCCAGTAAAATCTCTCC
TACGATTGAAATGTGTTTCCAGGAATTTGCATACACTCATACAATCATCGGCATTCATTAATCTTCATCTCAATCGTAC
CAGCATCATCAACGAGGAATTCATTCTTTTCAAACGCTCATTAAAAGAAGAACCCGATCGATTTAGAAATATCATGTCT
TTTCTCTCTAGTGGTCATGATAATTATGACCTTCACCATGTTTCTCCAGATCTAGATGTGCCATATCTGACTACAACAG
GAGCTTGTACATCTCACAGATTCATGGGTCCTTGCCATGGCTTAATTGTCTTCACGGATGGTGAAGAAACAGAAGTATT
ATTTAATCCATCAACTAGAAATTATAGGCTACTCACACCTAGCCCGTTTGATAGCCCATTGGGTTTCCATCGTAGCATT
GACGGTATTGCATTCGGTTTTGATTCGATTGGAAATGATTACAAGATTGTGAGGATCGCAGAACTTCTTGGAGAACCAC
CTTTTAATTGTTTTAGTACGAGAGAGTGGAGAGTTGAAGTTTTCGAAATGAGTATTGATTCATGGAGAGAGGTAGAAAA
TGTGGATCAACAACTGCGTTATGTGCATTGGTATCCCAGTGCTGATTTGTTCTACAAAGGGGCCTCTCATTGGTTTGGC
AACGAAAATAGAGTCCATGTAATTGTTTGTTTTGACATGTGTACAGAGATATTTCGCACTTTTAAAATGCCTAGTACTT
GCCATTACAAAGACAAAAATTTTTACTGCCTTGTAGTCTTGAATAAGTGTCTAACGTTGATTTGTTACCCCTATCTAGG
TTATGAAATTGATCCTGCAATTGATTTCATGGAGATTTGGATAATGAAGGAGTATGGGATATACGAGTCTTGGAGTAAA
ACATACAGAATCAGACCTCTTGCAATTGAATCGCCATTGGCAATTTGGAAGGATCATTTATTGCTCCTTCAGAGCATAA
GTGGGTATTTGATTTCCTATGATCTGAATTCTGGTGAAGTCAAGGAATTCGAATTGAATGGTTGGCCCGACAGTTTGCG
AGTAACAGTTTACAAGGAAAGCTTGGCTTTAATTCCAAATTCCAAAAGACCGCGAGCA
>P_hybrida_S9_SLF16_AB932997
ATGTCGGATGAAATTGTGATAAAGTTGCCTAATGATGTGGTGATGTATATACTTTTGAAATTTCCAGTAAAATCTCTCT
TACGATTCAAACGTGTTTCTAGAGATTTGTATACTCTCATACAATCATCGGTACTCATAAATCTTCATCTCAATCGTAA
CATCACCGCCAACAATGAATTCATTCTTTTCAAACGCTCATTAAAAGAAGAACCCAACTTATTTAGAAGTATCATGTCC
TTTCTCTCTAGTGGTCATGATGATTATGATCTTCACCGTGTTTCTCCAGATCTAGATGTGCCATATCTGACTAACACAG
GAGGCTGTACGTTTCACAGATTCATGGGTCCTTGCCAGGGCTTGTTGGTTTTAACAGATTGCGAAGAAACAGTACTATT
TAATCCATCAACTAGAAATTATAGGCTACTCCAACCTAGCCCGTATGATAGTCCATTGGGTTTCCATCGTAGCATTAAC
GGTATTGCATTCGGTTTTGACTCGATTGGAAATGAATACAAGATTGCGAGGCTTGCAGAACTTCGTGGGGAACCACCCT
TTAATTGTTTTACTATGAAAGAGTGGAGAGTTGAGGTTTACGAATTGAGCATTGATTCATGGAGAGAGATAGAAAATGT
GGATCAACAGCTGCCTTATGTGCATTGGTATCCGTGTGGCGAGTTGTTCTATAAAGGTGCCGCTCATTGGTTTGGCCAC
GCAAATAGAGCTCGTGTAATTCTTTGTTTTGACATTAGTACGGAAACATTTCGCGATATTAAAATGCCTAATACTTGCC

ATTACAAAGACAGGAAGTGTTACGGCCTTGTTGTCTTGAATGAATGTCTAACGTTGATTTGTTACCCCTATCCAGGTTG
TCAAATTGATACTGCAATAGATTTCATGGAGATTTGGATGATGAAGGAGTATGGTATAATCGAGTCTTGGAGTATGAAA
TACAAAATCACACCTCTTGCAATTGAATCGCCATTGGCAATTTGGAAGGATCATTTATTGCTCCTTCAAAGCATAAGTG
GGTATTTGATTTCCTATGATCTGAATTCTGATGAAGTCAAGGAATTCGAATTGAAAGGTTGGCCCGAGAGTTTGCGAGT
AAATGTTTACAAGGAAAGCTTGGCTTTAATTCCAAAAGACCGCGAACATAATATGCGGTTATCAATT
>P_hybrida_S9_SLF16_AB932998
ATGTCGGATGAAATTGTGATAAAGTTTCCTAATGATGTGGTGATGTATATACTTTTGAAATTTCCAGTAAAATCTCTCT
TACGATTCAAACGTGTTTCTAGAGATTTGTATACTCTCATACAATCATCGGTACTCATAAATCTTCATCTCAATCGTAA
CATCACCGCCAACAATGAATTCATTCTTTTCAAACGCTCATTAAACGAAGAACCCAACTTATTTAGAAGTATCATGTCC
TTTCTCTCTAGTGGTCATGATGATTATGATCTTCACCGTGTTTCTCCAGATCTAGATGTGCCATATCTGACTAACACAG
GAGGCTGTACGTTTCACAGATTCATGGGTCCTTGCCAGGGCTTGTTGGTTTTAACAGATTGCGAAGAAACAGTACTATT
TAATCCATCAACTAGAAATTATAGGCTACTCCAACCTAGCCCGTATGATAGTCCATTGGGTTTCCATCGTAGCATTAAC
GGTATTGCATTCGGTTTTGACTCGATTGGAAATGAATACAAGATTGCGAGGCTTGCAGAACTTCGTGGGGAACCACCTT
TTAATTGTTTTACTATGAAAGAGTGGAGAGTTGAGGTTTACGAATTGAGCATTGATTCATGGAGAGAGATAGAAAATGT
GGATCAACAGCTGCCTTATGTGCATTGGTATCCGTGTGGCGAGTTGTTCTATAAAGGTGCCGCTCATTGGTTTGGCCAC
GCAAATAGAGCTCGTGTAATTCTTTGTTTTGACATGAGTACGGAAACATTTCGCGATATTAAAATGCCTAATACTTGCC
ATTACAAAGACAGGAAGTGTTACGGCCTTGTTGTCTTGAATGAATGTCTAACGTTGATTTGTTACCCCTATCCAGGTTG
TCAAATTGATACTGCAATAGATTTCATGGAGATTTGGATGATGAAGGAGTATGGTATAATTGAGTCTTGGAGTATGAAA
TACAAAATCACACCTCTTGCAATTGAATCGCCATTGGCAATTTGGAAGGATCATTTATTGCTCCTTCAAAGCATAAGTG
GGTATTTGATTTCCAATGATCTGAATTCTGATGAAGTCAAGGAATTCGAATTGAAAGGTTGGCCCGAGAGTTTGCGAGT
AAATGTTTACAAGGAAAGCTTGGCTTTAATTCCAAAAGACCGCGAACATAATATGCGGTTATCAATT
>P_axillaris_S19_SLF14_AB933050
ATGAAGATCGCATTGGAAGAAATCCATGATGGCAATGGAGTTGTGAAAAAATTGCCAAAAGATGTGGTGAATAATATAA
CCTTAAAGCTTCCAGTAAAATCTCTCTTGCGATTTAAATGTGTCTCTCAATTTTGGTACGCGTACATACAATCATGGGC
ATTCATCATTCTTCATCACAATTGTGCTAGCAGTGTCAACGATGAAATTATTCTCTTCAAGCGCTCGTTCAAAGAAGAA
CATGACCATTTTAAAAGTATTATGTCATTTCTCTCTAGTGGTCATGATGGCGATGACTTTCACCATGTCTCTCCCGATT
TAGAAGTGCCATATCTAACTAACACTGCTTCTTGTACTTTTCACCGATTCATTGGTCCTTGCCATGGTTTAATTGTATT
AACGGATAAAGTAACTGCAGTATTATTTAATCCAGCAACTAGAAATTATAGGCTACTCAAACCTAGCCCTTTTGGCAGT
CCACTAGGTTTCCATCGTTCCATTAATGGTATTACATTTGGTTTTGACTCGATTGCAAATGAATACAAGATTGTCAGAC
TTGCTGAAATTCGTGGGGAGCCACCTTTCTATTGCTATTCTGTGAGAGAGTGGAGAGTTGAGGTTTATGAATTGAGCAT
TGATTCATGGAGAGAGGTAGAAAATGTGGATCAACAGTTGCCTTATGTGCATTGGTATCCTTGCGCTGAGTTATTCTAC
AAAGGTACATCTCATTGGTTCGGAAACACAAATACAGTTGTTATTCTTGGTTTTGACATGAGTACCGAGACTTTTCGCA
ACATTAAAATGCCTAATACTTGTCATTTCAAGGACAGGAAGTGTTATGGCCTCGTAGTCTTGAACGAGTCTCTAACGTT
GATTTGTTACCCCTATCCACGGTGTGAAATTGATCCAGCAATAGATTTTATGGAGATTTGGATAATGAAGGAGTACGGC
GTAAATGACTCTTGGAGTAAGAAGTACACAATTATACCTCTTGCGATTGAATCACCATTGGCAATTTGGAAGAATCATT
TATTGCTACTCCAAAGCATAACTGGACATTTGATTTCCTATAATCTTAATTCTGATGAGATCAAGGAATTCAATTTACA
TGGTTGGCCTAAAAGTTTGAGAGTAAAAATTTACAAGGAAAGTTTGACATTAATTCCAAAAGAAAGTGAGTTAATACAG
CTCAATAATATTAGAGCAGAT
>P_hybrida_S22_SLF18_AB933071
ATGGTGGAGGGACTTTTGAAAACATTACCCAATGATTTAACTATTTATATACTTTTGATTCTTCCGGTGAAAGCTCTGA
TGCGACTGAAATGTGTTTCCAAAACTTGTTACACTCTCATACAATCGTCTGCCTTCGTGGATCTTCATCTAAATCGCAA
AACAACATCCAAAGATGAGTGCATTCTCTTAAAGCGTTCCCTCGAAGAAGGAATCAACCGATATAAAACTAGCTTGTCT
TTTCTTTGCGGTGATGACCATGATTATCTTAGTCCGATCATTCATGATGTAGATGTGACACATTTGACAACCAATTGTA
ATTTCTGCCATGATCAACTTGTCGGTCCTTGCCATGGATTGATCGCTTTAATGCACTCTCCTACCACTGTTTTATTTAA
TCCATCTACTAGAAAATATAAGCTTCTCCCACCCAGTCCTCTTCGTCATCTAAAGGGATTCTATCGTTCAATGGAAGGT
GAAGGGTTTGGCTTCGACTCCATTATAAATAACTACAAGGTTGTTAAAATTTCAACTATTTATAAGGTTGATCATTTCG
ATTATCTTGAAGAGATTGGGAGAAAAGTAGAGGTCTATGATTTAAGCACTGATTCTTGGAGAGAATTGGATCATGTGGC
TCAAGAGTTGACCACATTATGCTGTGTCGAGTGTACCCAGATGTTCTACAAGGGAGCTTGCCATTGGATTGCAACTCAA
GATCTTGACGCATTCAGAATTCTTTGTTTTGACATGAGCTCTGAAGTTTTTCGAAGTTTGAAAATTCCTGAAAATTGTC
ATTTATTCGAGGGACCATGGTGTAGGCTCGCGTTAATACAAGAGTCTCTAACGTTAATTTATTATCGCTATCCGGATCA
ATCGACTGCTCAAGGAAAAGATTCGTCGGTTGTTTGGATAATGAAAGATTACAGTGTACATGAGTCTTGGGTTAAGAAT
TACACTATTACTTCTGTTCCTATTCATTCTCCATTAGCTGTTTGGAAGGGTTATTTGTTGGTTTTTGAAGGTAAAAGTG
GATGTTTGATGTCTTATGATCTGATTTGCAATAAAATCAAGGAGTTGAATTTTCATGGCTTTCCTGAAAGTCTCAGAGC
TTTGGTTTACAAGGATAGCTTGATATCAATTCCAATTGGAAGCGAGCATTCTGCACAAGTTCACAGATTT
>P_hybrida_S11_FBX1_AB933024
ATGGCAGATCGAATTACGAAGAGGTTGACACAAGATGTGATTGTCCATATACTTATAAGGCTTGCAGTGAAACCTCTTA
TGCGATTGAAATGTGTCTCGAAAAGTTGTTACACTCTTATAAAATCCTCCACTTTCCTCAATCTTCATATCCACAGAAA
AACAACTTCCAAAGATGAACTCATTCTCTTCAAGCGTTCATTCAAGCAAAACACTGGCCAATATACTACGATCTTGTCA

TTTCTGTCTGGCGATGATGATGACTATCTTAACTCGATTTTTCCAGATCTAGATGTGACTCATCTTACCTCGATTCATC
ATTATAATAATGATCAACTCGTCGGTCCTTGCCATGGTTTGATTGCATTGATGGACTCCCATATCACCATCTTATTTAA
TCCGTCTACCAGAATTTATAAACTTCTGCCGCCCAACCCTTTTGGTTGTCAAAAAGGTTTTTTTGATTCAACCGAAGCT
GTTGGATTTGGCTTCGACTCTATAGCCAACGACTACAAGGTTGTTAGAATTTCAATAATTTACATTGTTAACGACGGGT
ATCCAGATGAGCATGAGAGAAAAGTTCAGATCTATAATTTGAGTAATGATTATTGGAGAGAGATAGATCATGCTGGTCA
ACAACTGACCACGTTTTTTATTGACCAATGTTCTCAAATGTTTTACAAGGGAACTTGTCACTGGATTGCATCTCAAGAC
ATAGACGCATTCTTAGTTCTTTGTTTTGACATGAGCACCGAGATCTTTCGAAGTTTTAAAATACCCGAAACTTGTCATT
ACTCTGATGGACCATGTTGTAGACTTGTTTTATTACATGATTCTCTAACATTGATTTATTACCCCTACCCAGAGCCTGT
GATTCCTCTGGAAAAAGAGATGCTGAACGTTTGGGTAATGAGGGATTACAGTACATATGAGTCTTGGATTAAAAAGTAC
ACAATTACAGGCCTTCCTATTGAAACTCCATTAGCAGTTTGGAAGAATTGTTTGTTGCTTTTTCAGAATAGAAGTGGCT
GTTTGATGTCCTACAATCTTGAATCCAATGAAGTCAAGGAATTAAACTATCATGGCTATCCTCAAAGTCTGAGAGTCGC
AGTTTACAAGGATAGCTTAGCTTCAATTCCAAGAGAAACTGAGCAAGTTCATAAATTC
>P_axillaris_S19_SLF6_AB568422
ATGGCTGATGGAATTATCAAAAAGTTGTCCGAAGATGTGGTTATTTTTATATTTTTCAGACTTCCAGTAAAATCTCTCA
TGCGATTCAAATTTGTCTCGAAATCATTTTTCACTCTCATACAATCCTCAACCTTTATCAATCTTTATCTCTACAACAC
AACAACTTCTAGAGATGAATATATTCTGTTAAAGCGTTGCTTCATACAAGAAAACAACCAATATGAGACTATTTTGTCT
TTTCTTGCTGGTGGTGATGATGATTATCTAAACCCAATTTTTCAAGATCTAGATGTGACTCATCTGACCTCCACTCGTA
ATTGTGATCATGATCAACTCATCGGTCCTTGTCATGGTTTAATGGCGTTGATGGACACCCAAACCACTATCTTATTTAA
TCCATCTACTAGAAATTATAGACCTCTCCGACCCAGCCCTTTCGGTTGTCCACAAGGTTTCCATCGTTGTATCCAAGCT
GTTGGGTTTGGCTTTGACACTGTCTCAAATGACTACAAGGTTGTTAGAATTTCGATAATCTATAAGGTAGACTACGATG
ACGAGTATCCAGTAGAGCGTGACCGAAAGTTTGAAGTTTATGATTTGGGTATTGATTATTGGAGAGAATTAGATAATTT
GAGTCAAGAGTTGACGACGTTTTGTGTTACTCATTGTTCTCAAATGTTTTACAAGGGTGCTTGTCATTGGATTGCATCT
CTAGACATAGACGCTTACATAATTCTTTGTTTCGATATGAGCTTTGAGACTTTCCGAAGTCTTAAAATTCCTGAATCTT
GTCATATAATTAACGGACCGACCTGCAGACTCGCTTTAGTGCATGACACTTTGACGTTGATTTATTACCCCTACCCGGA
GCCCGAGATTCCTGTGGAAAAAGATTTGATAAACATCTGGTTTATGACGGAATACAACGTATATGAGTCTTGGATCCGA
AAATACACAATTAGAGGTCTTCTTATTGACTCCCCATTAACAGTTTGGAAGGGTTATTTGTTGCTTTATCAGAGTAGAA
GTGGATACTTGATGTCCTATAATCTTAATTCCAACGATGTGAGGGAATTCAATTTTCATGGTTATCCTAAAAGTCTTAG
AGCTATAGTTTACAAGGATAGCTTGACTTCAATTCCAAGAGAAAGCGAGCATGCAAAACAAGTTTATAAATTT
>P_axillaris_S19_FBX3_AB933055
ATGAATAAATTGGCCCAAGATATGGTTGTTAATATACTTTTGAGGCTTCCCGTAAAATCTCTCATGCGATTCAAATGTG
TCATTAAAACTTATTACAGTCTCATACAATCCTCCTCTTTCATCAATCTCCAGCTCAACCGTGTCACCACTGACAAAGA
TGAATTGGTTCTTTTTAAGCGCTCATTCGAAGAAGATATTCACCGACATAAAACTATCTTGTCTTTTCTTTCCAGCAGT
GATGTTGATAGTTCTCTCAACCCCATCAGTCCAGATCTAGATGTGCCTCGTATGACCAATGCTTATAGTAATAATTTTG
ATCAACTTATTGGTCCTTGCAAAGGTTTGATTGCTTTGATGAATCACCTTGTCACTGTCTTAATTAATCCATCCACTAG
AAATTATAGGCTGCTCCCATCTAGTCCTTTTGATTCTCCACCGGGTTTCTATCGTTCTATTGAAAGTGTTGGGTTTGGC
TTTGACTCCATTGCAAATGATTACAAAGTTATTAGAATTCTAGAAGTTTACTGGATAGATCATGGGTATCCTCTAGGAG
GTGAGAAAAAAGTTGAGATTTATGATTTGGGTATTGATTCATGGAGAGAATTGGATCATGTGGATCAACAATTTCCCCA
GTTGCATTGGTTACCATGTTCACAAATGTTTTACAAGGGGGCTTGTCATTGGATTGCCATCCCGTTGGTAGACCCGATG
GTAATTTTATCTTTTGATCTGAGTACTGAGATCTTTCGCACCATTAAAATGCCTGACAATTGTTGTTTTTCAGATGGAC
CGTGTTATAGCCTCGTGTTGTCCAATGATTCTCTTACATTAATATGTTACCCGGATCCAGCACAGGTTGTTGATCCTAC
AAAAGATTTGATAGACATATGGATAATGAAGGATTACGGTGTCCATGAATCATGGATTAAGAAAAATACAATTATACGT
CTTCGTATTGTATCTCCATTAGCAGTTTGGAGAGAATCTTTACTGCTTTGTGAACGCAAAAATGGAATTTTGATGTTCT
ACAATCTTTGTTCCAATGAAGTCAAGGATTTCAATTTACATGGTTCTCCTAAAAGTCTGAGAGCAATGGTTTACAAGGA
AACCTTAACTCCAATTCCAAAAGGAAACGAGAAGAGCACAGAAGTTCAAAAATTT
>P_axillaris_S19_SLF4_AB568410
ATGAAATTATATTGTAAAGAATACAAGATGGCGGATAGAATTTTAATGAAATTGCCCCAAGATGTGTTTATTTATATAC
TTTTGAAGCTTCCTGTGAAACTTCTCATGCGATTCAGATGCGTCTCTAAATCATGTTACACACTTATACAATCTTCTGC
TTTTATCAATATTCATCTACACCGCACCACAACACCGGAAGATGAATATATTCTCTTCAAGCGCTCCTTCAAAGAAGAT
GTTGAAAGTTATAAAGGCATCATTTCTTTTTTTTTCTAGTCATAATGATGATGGCAATCTAAACTCTATTATTCCAGATT
TAGATGTTCCTAATATGACATCCCTTTATAGTATTGACTATGACAAACTCATTGGTCCTTGTCATGGTTTGATTGCTGT
GATGGATTCACGTTCCACCATCTTGTTCAATCCATCTACTAGAAAATATAGACTGCTCCCCTCAAGTCCTTTTGGCATT
CCGAAGGGATACTATCGATCCATTGAAAGTGGTGGGTTTGGTTTCGACTCCGTTGTTAATGACTACAAGGTTTTTCGAA
TTTCTGATGTTTACACCGAAGATCGATTCGGGTATCCTGAGGAGGGAGAGAGAAAGGTTGAAGTTTATGAAGTGGGTAT
TGATATTTGGAGAGAATTGGATCACGTGGATCAAGAGTTGCCCAGATTGTTTTGGTTGACTTCTTCGATGTATTATAGT
GGAGCTTACCATTGGATTACAACTTTAAATCATGAAGACCAACTGATAATTCTTTGTTTTGACATGAGTACTGAAATTT
TCCGCAACATAAATACGCCTGATACCCGTCAATTTCCAAGTGGAACATGTCATAGCCTCGTGCTGTTGAATGAGTGTCT
AAGTTTCATGTGTTACCCCTATCAAGGTCAAGGACCCGAGATTGACCATACAACAGATTTGATTGATATTTGGATGATG
AAAAATTATAATGTTTACGAGTCTTGGACAAAGAAATACATAATTAGAGTTCTTCCTATTGATGAATCACCATTAGCAG

TGTGGAAAGATTCTTTATTGTTTTTTCAAGGAAAAAGTGGATATTTGATGTCGTGTGATTTTAAATCCGAAGAAGTCAA
GGAATGGAATTTACACGGTTGTCAGAAAAGTATGAGAGCTATAGTTTACAAGGAAAGCTTGGTTGCAATTCCAAGTGGA
AGCCAAAGTAGTACACAAGTTCAAAACATT
>P_hybrida_Sm_SLF12Sm_A_AB933101
ATGCCGGACGGAATTATTATGAAATTGCATCAAGATATTATTATCTATATGCTTTTGAGGCTTCCAGTAAAGTTTCTCT
TGCGATTCAAATGCATTTCTAAATATTGTCACACTCTCACAAAATCTTCCACTTTTATCAATATTCATCTCAACCGCGC
CACAACTTCAGAAGATGAATATATTCTTTTCAAGCGCTCCTTCAAAGAAGATGTTGAAAGCTATAAAGGCATATTTTCT
TTTCTTTCTACTGATAATGGTGATGATCTTAACTGTATTTTTCCAGATCTAGATGTTCCTAATATGACATCCCTTTATA
GTATTACGCAGGACAAACTCATTGGTCCTTGCCATGGTCTGGTTGCTGTGATGAATGTAAGTTCCATCATCTTGTTAAA
TCCAGCTACTAGAAAATATAGACTGCTCCCGTCAAGCCCATTTGGTGTTACTAAGGGATTCTATCGTGACATTGAAAAT
GGTGGGTTTGGTTTCGATTCCGTTGTTAATGACTATAAGGTTTTTATAATTTCTGAAGATTACACAGAAGATCGTTATG
GGTATCCTGAAAAGGGAGAGAGAAAAGTTGAGGTTTATGAATTGGGTATTGATGTCTGGAGAGAATTGGATCATGTGGA
TCAACAGCTGCCCAAGTTGTTTTGGATGACTTCTTCGATGCCTTATAATGGAACTTATCATTGGTTAATAACATTAAGT
TATGAACATCGATTGATACTTCTTTGTTTTGACATGAGCACTGAAATTTTTCGCTATATCAAGACGCCTAACACTCGCT
ATTTTTCAAGTGGAACACGCCATAGCCTCGTGCTCCTGAATGATTGTCTAAGCTTCATGTGTCACCCCTTTCCAGGACC
CGAGATTGATCCAACAAAAGATTTTATTGACATCTGGATGATGAAAGATTATAATGTTTATGAGTCTTGGATAAATATA
TACACAATTAGAATTCTTCCTATTCATGAATTCCCTTTGGCAATATGGAAAGATTCTTTGTTGTTTTTTCAAGGAAAAA
CTGGATATTTGATGTCATATAATCTTAATACCGATGAAGTCAAGGAACTGAGTTTAAACGGTTGTAAGAGAAGTATGAG
AGCTATAGTTTACAAAGAAAGCTTGGCTCCCATTCCAGAAGGAAGCGAAAGTAGTACACAAGTTCACAACTTC
>P_hybrida_Sm_SLF12_B_AB933102
ATGCCGGACGGAATTATTATGAAATTGCATCAAGATATTATTATCTATATGCTTTTGAGGCTTCCAGTAAAGTTTCTCT
TGCGATTCAAATGCATTTCTAAATATTGTCACACTCTCACAAAATCTTCCACTTTTATCAATATTCATCTCAACCGCGC
CACAACTTCAGAAGATGAATATATTCTTTTCAAGCGCTCCTTCAAAGAAGATGTTGAAAGCTATAAAGGCATATTTTCT
TTTCTTTCTAGTAATAATGGTGATGATCTTAACTGTATTTTTCCAGATCTAGATGTTCCTAATATGACATCCCTTTATA
GTATTACGCAGGACAAACTCATTGGTCCTTGCCATGGTCTGGTTGCTGTGATGAATGTAAGTTCCACCATCTTGTTAAA
TCCAGCTACTAGAAAATATAGACTGCTCCCGTCCAGCCCATTCAGTGTTCCCAAGGGATTCTATCGTGACATTCAAAAT
GGTGGGTTTGGTTTCGATTTCGTTGTTAATGACTATAAGGTTTTTATAATTTCTGAAGTTTACACAGAAGATCCTTACG
GGTATCCTGAAGAGGGTGAGAGAAAAGTTGAGGTTTATGAATTGGGTATTGATGTCTGGAGAGAATTGGATCATGTGGA
TCAACAGCTGCCCAAGTTGTTTTGGATGACTTCTTCGATGCCTCATAATGGAGCTTATCATTGGTTAATAACATTAAGT
TATGAACATAAATTGATACTACTTTGTTTTGACATGAGCACTGAAATTTTTCGCTATATCAAGACGCCTAACACTCGTT
ACTTTTCAAGTGGAACACGCCATAGCCTCGTACTCCTGAATGATTGTCTAAGCTTCATGTGTCACCCCTTTCCAGGACC
CGAGATTGATCCGACAAAAGATTTTATTGACATCTGGATGATGAAAGATTATAATGTTTATGAGTCTTGGATAAATATA
CACACAATTAGAATTCTTCCTATTCATGAATTCCCTTTAGCAATATGGAAAGATTCTTTGCTGTTTTTTCAAGGAAAAA
ATGGATATTTGATGTCATATAATCTTAATACCGATGAAGTCAAGGAACTGAGTTTAAACGGTTGTAAGAGAAGTATGAG
GGCTATAGTTTACAAAGAAAGCTTGGCTCCCATTCCAGAAGGAAGCGAAAGTAGTACACAAGTTCACAACTTT
>P_axillaris_S19_SLF5_AB568416
ATGAAGATGCCACATGGAATTATGAAGAAATTGCCTGAAGATATGATTCTATGTATATTTCTGAGGATTCCTGTAAAAT
CTCTTATGCGATTCAAATGCGTCTCTAAAAACTATTACACTCTCTTACAATCCACCACCTTCATCAATCTTCATCTCAA
TCGCACCACAACGGTGAAAGATGAATTCATTCTCCTTAAGCGCTCTTTCAAAGAAGATCTTAATCAATATAAAACTATA
TTTTCTTTTCTTTCAGGTGATGGTGATCATGATTATCTTAACCCCATTTTTTCAGATTTTGATGTGCCTAATATGACCG
ACACTCAGAGTATTATTTTTGATCAACTCATTGGTCCTTGTCATGGTTTGATTGCTTTGATGGATGATTTTACAACTAT
CATATTTAATCCATCTACAAGAATTTTTAGGCTACTCCCTCCCAGCCCTTTTGATCGTCCAAAGGGATACCACCGATCC
ATCAAATGTCTTGGATTTGGTTTTGACTCAGTTGTTAATGACTATAAGGTTGTTAGAATATCTGAGTTTCTCAAGGATG
ATTGTTACGGATATGTTCAAGTGGAAGAGGAAAATGTTGAGATTTATGAACTGGGGATTGATTGTTGGAGGGAATTGGA
TCGTGTAAATCAACAATTTCCTACCATATTTTGGGTGCCTTGTTCTCAGATTTTTTATATGGGAACTTTTCATTGGATT
GCCCAAAGGGTAATTCTTTGTTTTAACATGAGTACTGAGATTTTTCACCATATAAGGATGCCAGATCCTTGTCATAATA
TTCGCAATCATAGCCTCGTCATCCTAAATGAGTCCCTAACCTTGATATGTTACCGTTCCGTAGCGCCAACAAGTGATCC
AATAGAAGATTTGATGGAAATTTGGATATTGAAAGATTATGATGTATCTGAGTCTTGGGTTAAGAAATACACAATTAGA
AGTCTTCCTATTAAAATCCCATTAGCCATTTGGAAAGACAATTTATTGCTTTTTCAAAACAGAAGTGGATATTTGATGG
TATATGATCTTCGTACTGGTAATGTCAAGGAATTAAATATACATGGTTGTCCCGAAAGTATGAGAGTCACAGTTTACAA
GGAAAACTTGACTATAATTCCAAGTGGAAGCGAGAGCAGTACATCAGTTCACAAGTTT
>P_axillaris_S19_SLF5_AB933041
ATGCCACATGGAATTATGAAGAAATTGCCTGTAGATGTGATTCTTTGTATATTTCTGAGGATTCCTGTAAAATCTCTTT
TGCGATTCAAATGCATCTCTAAAAATTATTACTCTCTCTTACAATCCACCATCTTCATCAATCTTCATCTCAATAGCAC
CACAACGGTGAAAGATGAATTCATTCTCCTTAAGCGCTCTTTCAAAGAAGATATTAATCAATATAAAACTATATTTTCT
TTTCTTTCAGGTGATGGTGACCATGATTATCTCAACCCCATTTTTCCAGATTTTGACGTGCCTAATATGACCGACACTC
AGAGTATTATTTTTGATCAACTCATCGGTCCTTGTCATGGTTTGATTGCTTTGATGGATGATCTTACAACTATCATATT
TAATCCATCTACAAGAAATTTTAGGCTACTCCCTTCCAGCCCTTTTGATCGTCCAAAGGGATACCACCGATCCATCAAA

GGTCTTGGGTTTAGTTTCGACTCAGTTGTTAATGACTATAAGGTTGTTAGAATATCCGAGTTTCTCAAGGATGATTGTT
ACGGGTATGTTCAAGTGGAAGAGGAAAATGTTGAGATTTATGAACTAGGGATTGATTGTTGGAGGGAATTGGATCATGT
AAATCAACAATTTCCTAAAATATTTTGGGTGCCTTGTTCACAGATTTTTTACATGGGAACTTTTCATTGGATTGCCCAA
AGGGTAATTCTTTGCTTTAACATGAGTACTGAGAATTTTCACCATATAAGAATGCCAGATCCTTGTCATAATATTCGCA
ATCATAGCCTCGTCATCCTGAATGTGTGCCTAACCTTGATTTGTTACCGTTCCATAGCGCCAACGAGTGATCCAATAGA
AGATTTGATGGAAATTTGGATATTGAAAGATTATGATGTATCTGAGTCTTGGGTTAAGAAATACACAATTAGAAGTCTT
CCTATTAAAATCCCATTAGCCATTTGGAAAGATAATTTATTGCTTTTTCAAAGCAGAAGTGGATACTTGATGGTATATG
ATCTTTGTACTGATAATGTCAAGGAATTAAATATACACGGTTGTCCTGACAGTATGAGAGTCGTAGTTTACAAGGAAAA
CTTGACTATAATTCCAAGTGAAGGCGAAAACAGTACACCAGTTCACAAATTT
>P_persica_ppa026586m_PG6
ATGACAGAGGAAATGGAGGTGCAAATCCTAGCAAGGCTGCCTCCCAAATCCCTAATGCGATTCAAATGTGTGTGTAAAT
CATGGCACGCTCTCCTCAATAACCCCCATTTTGTAGCCAAGCACCTTCACTTGTACAACAACCAACCTTCCTCGACTTG
CATCCTCTTCAAGCGTTCTGTCCTCAGCAGGACAGAACACAACAAGGAGGAGCTCGTATTCACATTCCTTAATCTTCGC
AACGACAACAAAAGCAATGCTGATCATAACCTTATCAATTGTAACGTTGAGGACCTCCATTTCCCACGTTCTATGGGTT
TAAAAAGTAGGGGCCAATTCATTGAGCTCCCTGGTTTGGAGCTTGGTGAATCTGTCCACATTGTAGGTCATTGTGATGG
GTTGTTTTGTTTGTCTCTTTACACTGGAGAACTTGTTTTCTATAATCCAGCTATCAAGGAGTTTAGGGTTCTTCCCCAA
TCATGCCTTGAAGATGCCTTCTCATGCACTTTGGGGTTTGGCTATGATCCCAAACGTAAAGATTATGTACTTCTCAGCA
TTATGTCTTATGGGGAGGAAATATTGGATGACGAGCGTCTCGTCATCCATCCTCCCCAAGCAGAAATATACACATTGAG
CACTAATTCTTGGAGGGAGATTGAGACTCATTACTTGGAAACAGAAACGACCTACTTTTGGGGCAATGAGACTTTCTCG
ACCTATTTCAATGGAGTGTTTTATTGGTTGGGGTATGAGGAGAAAAAGGAGTTTGTGTCTTTCTATGACAGGCTTGAGG
AGGAAAAGAAGCAAGTGATCATTTTGTTTGACACATTTGATGAAGTTTTTCACAATATGCCACTTCCGGATTGTTTCTA
TGAGTTTCCAACTCATGAAATGTCCCTTACGGTGTGGAACGAATCCATTGACACCATTGCTCTTTTCGGGTTTTATCGT
TGCGAATTTGAAACCTTTGAGGTTTGGGTGATGGATGAGTTTGATGGGTGGACAAAACACTTATCTGTTGTGCCCAAAG
TGGATCAGGAGGTGGATATACCATTGGCACTTTGGAGGAGGAACGAGGTTCTTTTGGTTGACAGAGATGGACGCATATT
CAGCTACAACTTCCATACCGAAAATCTTAAGTTTTTCCCTGTTCATGGTGTATCCAGAGGAGATTTTCAGGCTGTTGTT
TGTGTTAATAGTATAGTTCCAGTCAAA
>P_mume_SLFL3_AB280958
ATGACATACTTTTACAAAATGACAGAAGAAATGGAGGTGCAAATCCTAGCAAGGCTGCCTCCCAAATCCCTAATGCGAT
TCAAATGTGTGTGTAAATCATGGCATGCTCTCCTCAATAACCCCCATTTTGTAGCCAAGCACCTTCACTTGTACAACAA
CCAACCTTCCTCCACTTGCGTCCTCTTCAAGCGTTCTGTCCTCAGCAGGACAGAGCACAACAAGGAGGAGCTTGTATTC
ACATTCCTTATTCTTCGCAACGACAACGAAAGCAATGCTGATCATAACCTTATCAATTGTAACGTTGAGGACCTCCATT
TCCCACGTTCTATGGGTTTAAAAAGTAGGGGCCAATTCATTGAGCTCCCTGGTTTGGAGCTTGGTGAATCTGTCCACAT
TGTAGGTCATTGTGATGGGTTGTTTTGTTTGTCTCTTTACACTGGAGAACTTGTTTTCTATAATCCAGCTATCAAGGAG
TTTAGGGTTCTTCCCCAATCATGCCTTGAAGATGCCTGCTCATGCACTTTGGGGTTTGGCTATGATCCCAAACGTAAAG
ATTACGTACTTCTCAGCATTGTGTCTTATGGGGAGGAAATATTTGATGACGAACGTCTTGTCATCCATCCTCCCCAAGC
AGAAATATACACATTGAGCACTAATTCTTGGAGGGAGATTGAGACTCATTACTTGGAAACAGAAACGACCTACTTTTGG
GGCAATGAGACTTTCTCGGCCTATTTCAATGGAGTGTTTTATTGGTTGGGGTATGAGGAGAAAAAGGAGTTTGTGTCTT
TCTATGACAGGCTTGAGGAGGAAAAGAAGCAAGTGATCATTTTGTTTGACACATTTGATGAAGTTTTTCACAATATGCC
ACTTCCGGATTGTTTCTATGAGTTTCCATCTCATGAAATGTCCCTTACGGTGTGGAACGAATCCATTGCTCTTTTCGGG
TTTTATCGTTGTGAATTTGAACCCTTTGAGGTTTGGGTGATGGATGAGTTTGATGGGTGGACAAAACACTTATCTGTTG
TGCCCAAAGTGGATCAGGAGGTAGATATACCATTGGCAATTTGGAGGAGGAACGAGGTTCTTTTGGTTGACAGAGATGG
ACGCATATTCAGCTACAACTTCGATACCGAAAATCTGAAGTATCTCCCTGTTCATGGTGCGTCCAGAGGAGATTTTCAG
GCTGTTGTTTGTGTTAATAGTATAGTTTCAGTCAAA
>P_avium_SLFL1_S2_AB360340
ATGTGGGAAGAGATGGCGTTGCGCCGTATCCTACCAAGATTGCCTTCCAAATCTCTAATACGATTCAAGTGTGTTCGTA
AATCGTGGTATAATTTGATCAACAGTCCCACGTTCGTGGAAAATCACCTGTCCAATTCCATGCACAACAAACTCTCCAC
TTGCGTCCTTTTCAATCGTTTTGTCCAGAGGGACGCTAACACTGGCGAGAAGGAACTTGGATTCTCCTTCCTTTATCTC
CGCAATGATTATGATGATGCTGAGCATAACGTCAATTTTCTTGTTGAGGACATCAAATTTCCACTTTCTTCGGGACAAT
ATATTGGGCTCGAGGTTATAGAATCGCCGTATATGGCAGGCCATTGTCATGGGATAGTTTGTCTAAGTGATTCTAGTAG
CAACCTTGTTTTATGCAATCCAGGAATCAAGGAAATCAAGCTTCTACCCAAATCATGCCTTCCAGATTGGTGGGGATGT
GCTGTGGGGTTTGGATATGATCCCAAATCTAAAGATTACAAAGTTAGTAGAATTGCATCTTATCAGGCTGAAATTTATG
GTGACGGTCTTATTCCTCGTCCCAGAGTCGAAATATACACCCTCAGTACTGATTCTTGGCGAGAGATCAAGAATAATTC
TTTAGAAACAGATACTACTTGCTTTTTTCCTGATTATTTCCAGATGTACTTCCAGGGAATTTGTTATTGGGTGGGGTAT
GAGCAACCAAAACAATCTGTGGAATACGAGGATGAGGAACAGAAGCCAATGGTCATTCTCTTCGACACTGGTGATGAAA
TATTTCATCGTATATTATTTCCTGATAGTTTTTATATGTATGAGGAGGGATCATCTTATGCTTATGAAATGTCTTATAT
TATGTATACTGATCTGCGCATTATACTGTGGAATGGATCTATTGCTCTTTTTGGCTTTAATCGTTTTAGTGCATTCCCT
GATTCCTATGGAATTTGGGTGTTGGCTGACTTTGACGGTGCTAAGGGTTCTTGGACAAAACACTTAACTTTTGAGCCAT
TGGTGGCCATTAAGAGGGTATTGGAATTTTGGAAGAGTGACGAGATTCTTATGGTTACAGAAGATGGAGATATAGTATC

TTACAACCTTGCAACTGAGAAGCTCAAGAACATTCCCATTAATAGCCCATCTGATTTCGAAACTGTTGTGTATGTGAAT
AGTTTAGTTCCGATAACAGGAGGCAATAAGCTTGAGAGTGCAGATATA
>P_persica_ppa021716m_PG6
ATGTGGGAAGAGATGGCGTTGCATCGTATCTTACCAAGATTGCCTTCCAAATCTCTAATACGATTCAAATGTGTTCGTA
AATCGTGGTATAATTTGATCGACAGTCCCACGTTCGTGGAAAATCACCTCTCCATTTCAATGCACAAGATACACTCCAC
TTGCGTCCTTTTCAATCGTTTTATCTACAGCGATGCTAACACGGGTGAGAAGGAACTTGCATTCTCCTTCCTTTATCTC
CGCAATGATTACGATGATGATGCTGAAAATAACGTCAATTTTGTTGTTGAGGACATCAAATTTCCACTTTCTTCAAGAC
AATATATTGGGCTCGGGGTTATAGAATCGCTATGTATGGCAGGCCATTGTCATGGGATAGTTTGTCTAAGTGAGTCTAA
TAGCAACCTTGTTTTATGCAATCCAGGAATCAAGAAAATCAAACTTCTACCCAAATCATGCCTTCCAGATTGGTGGGGA
TGTGCTGTGGGCTTTGGATATGATCCCAAATCTAAAGATTACAAAGTTAGTAGAATTGCATCTTATCAGGCGGACATTT
ACGGTGATGGTCTTATTCCTCCTCCTAGAGTTGAAATATACACCGTCAGTACTGATTCTTGGCGAGAGATTAAGAATAA
TTCTTTAGAAACAGAAGCTACTTTCTTTTGTCCTGATTATTTTCAGATGTACTTCCAGGGAATTTGTTACTGGGTGGGG
TTTGAGCAACCAAAACAATCTGTGGAATATGAGGATGAGGAACAAAAGCCAATGGTCTTTTTATTTTATACGGGTGATG
AAATATTTCATAATATATTACTTCCTGATAGTTTTTATACATATGAGGACGGATCGTCTTATGCTTATGAAATGTCTTA
TATTATGTATACTGACCTGCGAATTATACTGTGGAATGGAGTGATTGCTCTTTTTGGCTTTAATCGTTTCAGTGCATTT
CCTGACTCCTACGGAGTTTGGGTGTTGGATGACGGTGCTAAGGGTTCTTGGACACAACACTTAACTTTTGAGCCCTTGG
TGGGCATTAAGAGGGTGTTGGAATTTTGGAAGAGTGACGAGATTCTGATGGTTACCGAAGATGGAGATATAGTATCTTA
CAACCTTGCAACTGAGAAGCTTAAAAATCTTCCCAGGAATAGCTCATCTGATTTCGAAACTATTGTGTATGTGAATAGC
TTAGTTTCGATCACAGGGAGGCAACAAGCT
>P_dulcis_SLFd_AB101660
ATGTGGGAAGAGATGGCATTGCGCCATATACTACCAAGATTGCCTTCAAAATCTCTAATGCGATTCAAGTGTGTTCGTA
AATCATGGTATACTTTGATCAACAATCCCACGTTCGTGGAAAATCATCTTTCTAATTCGATGCAGAGCAAACTGTCTAC
CTGCGTCCTTTTCAGTCGTTTTGTCCAGAGCGACGCTAACAGTGATGAGAAGGAACTTGCATTCTCCTTGCTTTATCTC
CGAAATGATTATGATGATGATGAGCATAACGTCAATTTTGTTGTTGAGGACATCAAGTTTCCACTTTCTTCGGGACGAT
TTATTGGGCTCGAGGATGTAGAATCTCCAAGTATTTTAGGCCATTGTAATGGGATAGTTTGTCTAAGTCCTTGTAGTGA
CAACCTTGTTTTATGCAATCCAGCAATCAAGGAAATCAAACTTCTACCCAAATCAGGCCTTCCAGATTGGTGGGGATGT
GCTGTGGGGTTTGGATATGATCCCAAATCTAAAGATTACAAAGTTAGTAGAATTGCATCTTATCAGGCGGAAATTGATG
GTCTTATTCCTCCTCCTAGAGTTGAAATATACACCCTCAGTACCGATTCTTGGCGAGAGATCAACAATAATTCTTTAGA
AACAGACAGTACTTGCTTTTTCCCTGATTATTTCCAGATGTACTTCCAAGGAATTTGTTATTGGGTGGGGTATGAGCAA
CCAAAACAATCTGTGGAATACGAGGACGAGGAACAGAAGCCAATGGTCATTTTCTTCGACACTGGTGATGAAATATTTC
ATAATCTATTATTTCCTGATAGTTTTTATATGTATGAGGAAGGATCATCTTATGCTTATGAAATGTCTTATCTTATGTA
TTGTGATCTGCGCATTATACTGTGGAATGGATCTATTGCTCTTTTTGGGTTTAATCGTTTTAGTGTATTTCCAGACTCC
TATGGAGTTTGGGTGTTGGATGACTTTGACGGTGCTAAGGGTTCTTGGACAAAACACTTAACTTTTGAGCCATTGATGG
GCATTAAGAGGGTATTGGAATTTTGGAAGAGTGACGAGATTCTTATGGTTACCATAGATGGAGATATAGTATCGTACAA
CCTTGACACTGAGAAGCTTAAGAATCTTCCCATGAATATCCCGTCTGATTTCGAAACTATTGTACATGTGAATAGCTTA
GTTTCGATCACAGGAGGCAACAAACTTGAGAGTGTAGATATA
>P_dulcis_SLFc_AB081587
ATGTGGGAAGAGATGGCATTGCGCCATATACTACCAAGATTGCCTTCAAAATCTCTAATGCGATTCAAGTGTGTTCGTA
AATCATGGTATACTTTGATCAACAATCCCACGTTCGTGGAAAATCATCTCTCTAATTCGATGCAGAGCAAACTGTCTAC
CTGCGTCCTTTTCAGTCGTTTTGTCCAGAGTGACGCTAACAGTGATGAGAAGGAACTTGCATTCTCCTTCATTTATCTC
CGAAATGATTATGATGATGATGAGCATAACCTCAATTTTGTTGTTGAGGACATCAAGTTTCCACTTTCTTCGGGACAAT
TTATTGGCCTCGAGGATGTAGAATCTCCAAGTATTTTAGGCCATTGTAATGGGATAGTTTGTCTGAGTCCTTGTAGTGA
CAACCTTGTTTTATGCAATCCAGCAATCAAGGAAATCAAACTTCTACCCAAATCAGGCCTTCCAGATTGGTGGGGATGT
GCTGTAGGGTTTGGATATGATCCCAAATCTAAAGATTACAAAGTTAGTAGAATTGCATCTTATCAGGTGGAAATTGATG
GTCTTATTCCTCCTCCTAGAGTTGAAATATACAGCCTCAGTACCGATTCTTGGAGAGAGATCAAGAATAATTCTTTAGA
AACAGATACTACTTGCTTTTTTCCTGATTATTTCCAGATGTACTTCCAAGGAATTTGTTATTGGGTGGGGTATGAGCAA
CCAAAACAATCTGTGGAATACGAGGATGAGGAACAGAAGCCAATGGTCATTTTCTTCGACACGGGTGATGAAATATTTA
ATCATATATTATTTCCTGATAGTTTTTATATGTATGAGGAGGGATCATCTTATGCTTATGAAATGTCTTATCTTATGTA
TTGTGATCTGCGCATTATACTGTGGAATGGATCTATTGCTCTTTTTGGCTTTAATCGTTTTAGTGTATTTCCTGACTCC
TATGGAGTTTGGGTATTGGATGACTTTGACGGTGCTAAGGGTTCTTGGACAAAACACTTAACTTTTGAGCCATTGATGG
GCATTAAGAGGGTATTGGAATTTTGGAGGAGTGACGAGATTCTTATGGTTACCGAAGATGGAGATATAGTATCTTACAA
CCTTGCAACTCAGAAGCTTGAGAATCTTCCCATGAATAGCTTATCTGATTTCGAAACTATTGTGTATGTGAATAGCTTA
GTTTCGATCACAAGAGGCAACAAGCTTGAGAGTGTTGATATA
>P_mume_SLFL1_Sf_AB280956
ATGTGGGAAGAGATGGCATTGCGCCATATACTACCAAGATTGCCTTCAAAATCTCTAATGCGATTCAGGTGTGTTCGTA
AATCATGGTATATTTTGATCAACAATCCCACGTTCGTGGAAAATCATCTCTCTAATTCGATGCAGAGCAAACTGTCTAC
CTGCGTCCTTTTCAGTCGTTTTGTCCAGAGCGACGCTAACAGTGATGAGAAGGAACTTGCATTCTCCTTCCTTTATCTC
CGAAATGATTATGATGATGATGAGCATAACGTCAATTTTGTTGTTGAGGACATCAAGTTTCCACTTTCTTCGGGACGAT

TTATTGGGCTCGAGGATGTAGAATCTCCAAGTATTTTAGGCCATTGTAATGGGATAGTTTGTCTAAGTCCTTGTAGTGA
CAACCTTGTTTTATGCAATCCAGCAATCAAGGAAATCAAACTTCTACCCAAATCAGGCCTTCCAGATTGGTGGGGATGT
GCTGTGGGGTTTGGATATGATCCCAAATCTAAAGATTACAAAGTTAGTAGAATTGCATCTTATCAGGCGGAAATTGATG
GTCTTATTCCTCCTCCTAGAGTTGAAATATACAGCCTCAGTACCGATTCTTGGCGAGAGATCAAGAATAATTCTTTAGA
AACAGATACTACTTGCTTTTTTCCTGATTATTTCCAAATGTACTTCCAAGGATTTTGTTATTGGGTGGGGTATGAGCAA
CCAAAACAATCTGTGGAATACGAGGATGAGGAACAGAAGCCAATGGTCATTTTCTTCGACACGGGTGATGAAATATTTC
ATCATATATTATTGCCTGATAATTTTTATATGTATGAGGAGGGATCATCTTATGCGTATGAAATGTCTTATATTATGTA
TACTGATCTGCGCATTATACTGTGGAATGGATCTATTGCTCTTTTTGGGTTTAATCGTTTTAGTGTATTTCCTGACTCC
TATGGAGTTTGGGTGTTGGATGACTTTGACGGTGCTAAGGGTTCTTGGACAAAACACTTAACTTTTGAGCCATTGATGG
GCATTAAGAGGGTATTGGAATTTTGGAAGAGCGACGAGATTCTTATGGTTACAGAAGAAGATGGATCTATAGTATCGTA
CAACCTTGAAACTGAGACGCTTAAGAATGTTCCCATGAATAGCCCATCTGATTTCGAAACTATTTTGTATGTGAATAGC
TTAGTTCCAATCACAGGCGGCAACAAGCTTGGGAGTGTAGATATG
>P_persica_ppa016317m_PG6
ATGTCTGAAGAGATGGTGTTGCATATCCTATCAAGATTGCCTTCAAAATCTCTAATGCGATTCAAGTGTGTCCGTAAAT
CGTGGTATACTATGATCAACGATCCCATGTTTGTAGAAAGGCACCTCTCCAATTCCATGCACAACAAACGCTCCACTAG
TTGCATCCTTGTCAATCGTTTTCTCCACAGCGACACTAGCACTGGTGAGAAAGAACTTGGATTCTCCTTCCTTTATTTT
TGCAATGATTATGACGATGATGAGCATAAAGTCAATTCTTTTGTTGAGGACATCAAATTTCCATTTTCTTCAGGAGGAC
AACGTAGTGGGTTTGAGGTTATAGAATCTCTAAGTATGATAGGCCAGTGTGATGGGATAGTTTGTTTATGTGACCGTCG
TGACAACATTGTTTTATGCAATCCAGCAATCAAGGAACTCAAGCTTCTTCCCAAATCATGCCTTCCACAGTTGATCCAG
TGTGCTGTGGGGTTTGGATACGATCCCAAATCAAAAGATTACAAAATTCATAGAATTTCATGTGATGGTGAGGAAATTT
ATGATCAGCGTCTTGTTTTTTTCCCTCCTAGAGTTGAAATATACACCCTCACTACTGATTCCTGGCGAGAGATCAAGAA
TAATTATTTAGAAACAGAGAACACTTTCTTTTGGCCTGAATATTTCGAGATGTACTGGAAAGGAATATGTTATTGGTTG
GGGTATGAGCAACCAAAAGAATTCGAGTCTTACTTTGATAGACTCGAGGATGAGAAAAAGAAGACAATGATATTTTTGT
TTGACACCGGTGATGAGGTATCTCATAACATATTACTTCCAGATTGTTTATATGAGGCAGCGGTATATCGTTTTGACAT
GCGCATTCTTCTGTGGAATGAATCTGTTGCTTTGTTTGGCCTAGATAGTTTTGGTACATTTGTTGAATGCTATGGATTA
TGGGTGTTGGATGACTTTGACGGTGCTAAGGGTTCTTGGACTAGACACTGCACTTTTGAGTCCGTGATAGACATTAAAA
GGGTATTGGCATTTTGGAATAATGACGAGATTCTTCTGGTTGACGGATGTGGGAATATAGTATCCTACAACCTTGGTAC
CAATAACCACAAAAATCTTTTAATATATACTGAGAATCTATGTCGTGTCGAAACTATTGTATATGTGAATACTTTAGTT
CCGATCATGGGAGGCAACAAGCTTGAGAGTGTAGATAAG
>P_persica_ppa019333m_PG6
ATGGCAGATCCAATCTTTTCAAAATTGTCAGAAGAGCTGGTGACGCATATCCTGTCAAGGCTGCCTCCCAAATCTCTCC
TGCGATTCAAATGCATTCGCAAGTCGTGGTCTGTTTTGATTACTACCCCTCAGTTTGTAGAAATGCACCTCTCCAACTT
CAGTTCCACCCGACACCAAATCTCCACCCCCTCCACCACCATCCTTTTCAAGCATTTTATCCTCACAGACCTCAACACA
GACCAAAAGGAACTCCTTTTCTCGATGTTTAATCTCCGCACTGATGATGTTGACAATGATGATATGGACGGTGGCGATG
ATCACCTCCGTCCCGTTTTCGAGGACCTCGAAGTTTCTTGTTTTCGAGGTGTAGACACAGGGGGAGAGTTTTATAGGGT
TGGCGTGGACATTTCAAGCCATTGTGATGGGATCGTTTGTCTGACTGATTTCCACAAAAAGGTGGCCTTATGCAACCCG
GCCATTAGAGAATTCAAGCTTCTTCCTGAATCAGACGTTCTTCTTTCTTCCCCTGAAGAAGTCGCCATACTTGGGGTAG
GGTTGGGGCGTGATCTCAAGCATTCTAAGAATTATAAAGTCGTCCGGCTTGTGACTTATGGAGACAAAAAAGTCGACGA
CGATCGTTTTGTTGTTCATCCTCCGAGAGCAGAAGTGTTCACCCTGCAGGGTTCAGATTCGTGGAAAGAGATCGAGATC
GGGAACATAGTGACGAAAACCGGATTCTTTTTGCCTCAGCATGCTGTGTCTGTGTACTGCAAGGGTGTTGTTTATTGGC
CTGCAACAGATAAGGACAGGGAGTACGTCGTCAGCTATAATTATACAGACGATGAGTATGATTACTGCGAGGAAAACGA
GGGCGAAGACGAGGAAGACAACCCTGAAAACAACGAGCGGCGGGCGAGGGAGGAGGAGGAGAGGAAGGAAAGGGACATG
AAAGCAGGAATTCTTTCGTTCGACTTTGGTGAAGAGGTGTTTGATATTATACCGTATCCTCCCGATGTAGGGTATTATG
AGAGCAAGATTTTTGGGGTGTGGAAGGAAAGCGTTGCTATATTTATTTATGCGTCAATGCCAGATTGCTTTGACATATG
GGTGATGGATGAGAGTGGGGAGAAGGGTTCCTGGACAAAATACTTAACCTTTGAGCCGGAAAAGCCATTGTTCCCGTTG
GCGTTGTGGAAGAGCGAGGAAGTTGTTATGGTGGCCAGGGATGATGTTGTGCTAATTTTCTACAACCTTGAAACCAGAA
AGTTTAAGTATCTTCCTCTTAATGGTGTGTTTTTTGATCGAACTGAAGCTGTTGTTTGTGCCAGCACTTTGGTTTCGAT
CCAGGGAGACAAACAAACTCAG
>P_persica_ppa025849m_PG6
ATGGCAACGTTGAGCAAATTTTCTGAAGATATGATGGGGAATATCCTGTCGAGACTGCCTCCCAAGTCTCTGATGCGAT
TTAGATGCGTCCTCAAGTCGTGGCATGACCTAATCGATAAACCTAGCTTCGTGGACCAGCACCTTTCCACTTCTATGGA
CAACAAAGTCACCTCCTCCACTTGCGTCCTCCTCAAGCACAATGTCCTCACGGACCCCACCATTAAGGACGACGAGAAG
GCAGTTAGAGCTACTCTCTTCAACCCCGACAGTAACCAAAGGGACATTTTACTCTCCTCGCTTAATCTCGGCAGCCTTG
TCGACGACGGTCTTGAGATTGAGAACCATGTCGTTCCGCCGCCTATGAGAGGATACGCATTGAGCCTAGAAATTTCAGG
CTCTTGTGATGGCCTCATCTGCCTCAATACTTTTAACAGCGAGGACATTGTTTTATGCAATCCAGCACTCGAGGAATAC
AGAGTTCTTCCCAAGTCTTGCATTCTTTTGCCTCCGCGAGTTCCACGACAAGTTGAAGAAAATGAAGACGATGATTATT
ATGAAGAAGATGACGATGACGAGATAGAATCGAACCCGAAATGTGTGGGGTTCGGGTACGATCCGAATTCGAAAGACTA
CAAAGTTGTTCGAGCTGCACAATTTGTCTCCGGGGTTTTTACCCAGCACCCCTCCAAAGTAGAGGTTTACAGCTTGGCT

GCCGATACTTGGAGAGAGATCCCTGTTGACATCCAGCCTCATGGTTCTCTAAACCCGTCTTACCAGATGTACTTCAATG
GATTCTTTTACTGGATCGCGTACTGGACGGAGGAAAGAAATGTCATCCTTTCGTTTGACATGAGCGAGGAGGTGTTTCA
TGACATAGCTCTTCCGGAGAGTGGCCCAGATGCATATGAATACACAAGCATTGCAGTGTGGAAAGACTCTCTGGTTCTC
TTGACCTCCCCGGTGGAAAACGAAGCTCCTAAAACCTTAGACTTGTGGGTCTTGGATGAAGAGTTGAAAGGTGCTAAGG
GTTTGTGGACAAAGCACTTGGCTATAGGACCTCTGGCAAAAGGGGTTGAGGCTCCATTGGTGTTTTGGAAAGATGAGGA
GCTTCTTATGGTTACGACTAATGGGGATGTGGTAAACTATAGCCTTGATACACAAATGCTCAAGCATGTCCCCCGCCAT
GGATTGGGAGAGCCAACCAATATCCAAGCGGTTCCTTACGTGAATAGTATTGTTTCAATTAAGCCAGGCAACAAGATTG
AAAGCATA
>P_mume_SLFL2_AB280957
ATGATGAGAGAAATGGCAACGTTGAGCAAATTTTCTGAAGATATGATGGGGAATATCCTGTCGAGACTGCCTCCCAAGT
CTCTGATGCGATTTAGATGCGTCCTCAAGTCGTGGCATGATCTAATCGATAAACCTAGCTTCGTGGACCACCACCTTTC
CATTTCTATGGACAACAAAGTCACCTCCTCCACTTACGTCCTCCTCAAGCACAATGTCCTCACGGACCCCAGCATTAAG
GACGACGAGAAGGCAGTTAGAGCTACTCTCTTCAACCCCGACAGTAACCAAAGGGACATTTTACTCTCCTCACTTAATC
TCGGCAGCCTTGTCGACGACGGTCTTGAGATTGAGAACCATGTCGTTCCGCAGCCTATGAGAGGATACGCATTGAGCCT
AGAAATTTCAGGCTCTTGTGATGGCCTCATCTGCCTCAATACTTTTAACAGCGAGGACATTGTTTTATGCAATCCAGCA
CTCGAGGAATATAGAGTTCTTCCCAAGTCTTGTATTCTTTTGCCTCCGCGAGTTCCACGTCAATTTGAAGAAAATGAAG
ACGATGATTATTATGAAGAAGACGAAGACGATGAGATAGAATCGAACCCAAAATGTGTGGGGTTCGGGTACGATCCGAA
CTCGAAAGACTACAAAGTTGTTCGAGCTGCACAATTTGTCTCTGGGGTTTTTACCCAGCACCCCTCCAAAGTAGAGGTT
TACAGCTTGGCTGCCGATACTTGGAGAGAGGTCCCTGTTGACATCCAGCCTCATGGCTCTCTAAACCCGTCTTACCAGA
TGTACTTCAAGGGATTCTTTTACTGGATCGCGTACTGGACGGAAGAAAGAAATGTCATCCTTTCGTTTGACATGAGCGA
GGAGGTGTTTCATGACATAGCTCTTCCGGAGAGTGGCCCAGATGCATATGAATACACAAGCATTGCAGTGTGGAAAGAC
TCTCTGGTTCTCTTGACCTACCCGGTGGAAAACGAAGCTCCTAAAACCTTAGACTTGTGGGTCTTGGATGAAGACTTGA
AAGGTGCTACTAAGGGTTTGTGGAAAAAGCACTTGGCTATAGGACCTCTGGAAAAAGGGGTTGAGGCTCCATTGGTGTT
TTGGAAAGATGAGGAGCTTCTTATGGTTACGACTAATGGGGATGTGGTAAACTATAGCCTTGATACACAAAAGCTCAAG
CATGTCCCCCGCCATGGATTGGGAGAGCCAACCAATATCCAAGCGGTTCCTTACGTGAATAGTATTGTTTCAATTAAGC
CAGGCAACAAGATTGAAAGCATA
>P_persica_ppa016207m_PG6
ATGCCAGAAGAAATGGTGGTGCAGATCTTGTCAAGGCTTCCTCCGAAATCTCTAATGCGATTCAGATGCGTTCATAAAT
CGTGGTACAATTTCATCAATGACCCCAACTTTGTGGATGTCCACCTCTCCAAATCCATAGACAACAGATTTTCATCCAA
GACCTCAACTTGTGTCCTCTTCAAGCGTTGTGTCCTCAACAATGAAGCAAATCATATTTTGTTGTCACTTGTTGATCTT
AGCAATGACAACGATGATGTTCAAATTCAATCTAATTCTATCCGAGACCTCAACCTTAATAATGTTCCTCCACTTTCTG
TAGGGCTAAGGCACGACTGTTTGGACATTGCAGGCCATTGCCATGGGATTATTTGTCTAACTGATTTCTCTGAGAATGT
TTTTCTATGCAACCCAGCTCTAAAGCAACTCAAGCTTCTTCCCAAGTCATGCCTTCGTCTACCTCAACCTCCCCCGAAT
ACCTTGAACAGATTGCAGTCGACAGGGGTAGCTGTGGGATTTGGCTACGACAGCAGAGCCAGGGTTTACAAAGTTGTTC
GAATCGTAATGCATTTTGAGGGGTTTTGGATCTTGTTCTTCCCTCACATGGCAGAGGTATATACCATGAGCTCTAACTC
TTGGAGAGAGATCAAGACTGATATACCAAGCACTGTGGTTTGGTCATCTTCTTCTTCCCAAATCTACTTCAAGGGTGTT
TACTATTGGTTTGCCTTGGAGCTGGACAAGGAAACCCTAGACGAAAACAAGAAAGTCATGCTCTCCTTTGACATGGACG
ATGAGTTATTTTTCCATACGCCGGTGCCGGATAGTTTACAAGATTCAGAAGAAAACTATGGAAGCCTTGGAGTGTGGAA
TGAATCCATTGCTCTTTTTTCTTATCACGTAGAAAGTGGGGTTTCTAAATTCATAGACATATGGGTGATGGATGGTTTT
TGTGGTACCAAGGGTTGTTGGACAAAGCACTTAACCATCGAACCTATAGCAGGCATTGGGATGCCGTTGACATTTTGGA
ACAGTGATGAGCTTCTTCTAGTTGCCACAGATGGATATGTAGTTTCCCACAATTTCGATACAAAAGTGCTTAGAAATCT
TCCTATTCATGGTGTGCTTTTTGAACATTTTCAAGCTGTGCTTTATACAAGTAGCCTCATTTCAGTTAAT
>P_aviumSFB3_AY571665
ATGACATTCACATTACGTAAGAACGAGATCTTAATCGACATCCTAGTAAGACTACCTGCAAAATCCCTCATTCGGTTTC
TATGTACATGCAAGTCGTGGAGTGATTTGATTGGCAGCTCAAGTTTTGTTCGCACACACCTTCATAGGAATGTCACAAA
ACATGCTCATGTCTATCTACTTTGCCTTCACCACCCACAATTTGAACGTCAGAACGACAATGATGACCCATATGATATA
GAAGAACTTCAGTGGTCACTTTTTTCCAATGAAAAGTTTGAGCAGTTCTCCAATTTAAGCCATCCTTTAGAAAACACAG
AGCATTTTAGAATATATGGTTCAAGCAATGGTTTAGTTTGCATGTCGGATGAGATATTGAATTTCGATAGTCCTATACA
AATATGGAACCCATCGGTTAGGAAATTCAGGACTCTTCCAATGAGCACCAACATTAACATGAAATTTTCCCATGTTTCT
CTCCAATTTGGGTTCCACCCCGGGGTTAATGACTACAAGGCTGTAAGGATGATGCATACCAACAAAGGTGCCTTGGCAG
TTGAGGTTTATAGTCTTAAAACAGATTGTTGGAAGATGATTGAAGTAATTCCTCCTTGGTTAAAATGCACTTGGAAGCA
TCATAAGGGTACATTTTTTAATGGAGTAGCATACCACATCATTGAGAAAGGTCCTATATGCAGCATTATGTCATTCGAT
TCAGGCAGCGAAAAATTCGAAGAATTCATAACACCAGATGCCATTTGCAGTCCACGGGAGTTATGCATTGACGTCTACA
AGGAACTAATTTGCTTGATTTTTGGATTTTATGGTTGTGATGAGGAGGGCATGGACAAAGTTGACTTGTGGGTTCTGCA
GGAAAAACGGTGGAAACAATTGTGTCCTTTTATTTTTCCTTTGAATCATTGTCATCGTACAATCGGGATTAGTATAGAT
AATGAACTCATAATGCAAAGAAGAGATTTCATTAAAGGAGTAGCATATCTGTATTTGTGTAATTACGAATCCAAGCAAG
TTCTTGAAACAGGAATTCAATTGGCCGTCATGAGATATGGCGAAATCGAATTCTTGTTTGCAATTACTTACACAGAAAG
TTTGGTTTTGCTCAATAATTAT

```
>A_thaliana_F_box_AT3G06240_NM111499
ATGAAAGCGATCCAGTTGCTGTGGGAAGCGATAATGGAGGCGACGAAGAGAGAAAGACGGAGAGAAGATGACGACGGCG
AAAAAGCTTCACCGGAATCACTCGTTCTTCCACCAGAGATCATTACAGAAATTCTTCTCCGATTACCAGCCAAATCGAT
CGGGCGATTCAGGTGCGTATCAAAGCTCTTTTGCACTTTATCGTCAGATCCAGGGTTCGCGAAGATTCACCTCGATCTG
ATCCTTCGAAACGAATCCGTAAGATCGCTCCACCGTAAGCTCATTGTGTCTTCACATAATCTGTACTCGTTAGATTTCA
ATTCGATCGGTGACGGAATTAGGGATTTAGCGGCTGTGGAACACAATTATCCTCTTAAAGACGATCCAAGCATTTTCTC
TGAGATGATTAGGAATTACGTGGGGGACCATCTGTACGATGATCGTCGCGTGATGCTTAAGCTGAATGCGAAATCGTAT
CGAAGAAACTGGGTTGAGATCGTTGGATCTTCCAATGGTTTAGTGTGTATCTCTCCTGGTGAAGGAGCTGTTTTCTTGT
ATAATCCAACTACCGGAGATTCCAAGAGATTACCTGAAAATTTTCGTCCCAAATCTGTAGAATACGAAAGAGATAATTT
CCAAACTTATGGATTTGGTTTCGATGGTCTCACTGATGATTACAAATTGGTGAAGCTTGTTGCTACCAGTGAAGATATT
CTCGATGCTAGTGTCTATTCCTTGAAGGCTGACTCATGGAGACGGATCTGCAATTTGAATTATGAGCACAACGATGGCT
CCTACACGTCCGGTGTGCATTTCAACGGTGCGATTCACTGGGTGTTCACAGAGAGTAGGCACAACCAAAGAGTGGTTGT
AGCATTTGATATTCAAACCGAGGAGTTTCGAGAGATGCCAGTGCCTGATGAAGCTGAAGATTGTTCCCATAGGTTTAGC
AACTTTGTGGTCGGAAGTCTCAATGGACGTCTCTGTGTGGTCAATAGTTGCTACGATGTGCATGATGATATATGGGTGA
TGAGTGAGTACGGTGAAGCTAAATCCTGGAGCAGAATTCGAATCAACTTGTTGTATAGGTCGATGAAACCGCTCTGTTC
GACTAAGAACGATGAAGAGGTTCTTCTGGAGCTTGATGGAGACCTGGTGTTGTACAACTTTGAAACCAATGCATCGAGT
AATCTAGGAATTTGTGGGGTTAAGCTCAGTGACGGGTTCGAGGCAAATACATACGTAGAGAGCCTCATATCACCCAACT
CTTATGGTATAGAGAGC
```
