# Supplementary material for: The identification of the Rosa S-locus and implications on the evolution of the Rosaceae gametophytic self-incompatibility systems
Source: Sci Rep. 2021 Feb 12;11:3710. doi: 10.1038/s41598-021-83243-8 (PMC7881130; doi:10.1038/s41598-021-83243-8)
Supplement: Supplementary file 4 — Supplementary Information 4. [file 41598_2021_83243_MOESM4_ESM.pdf]

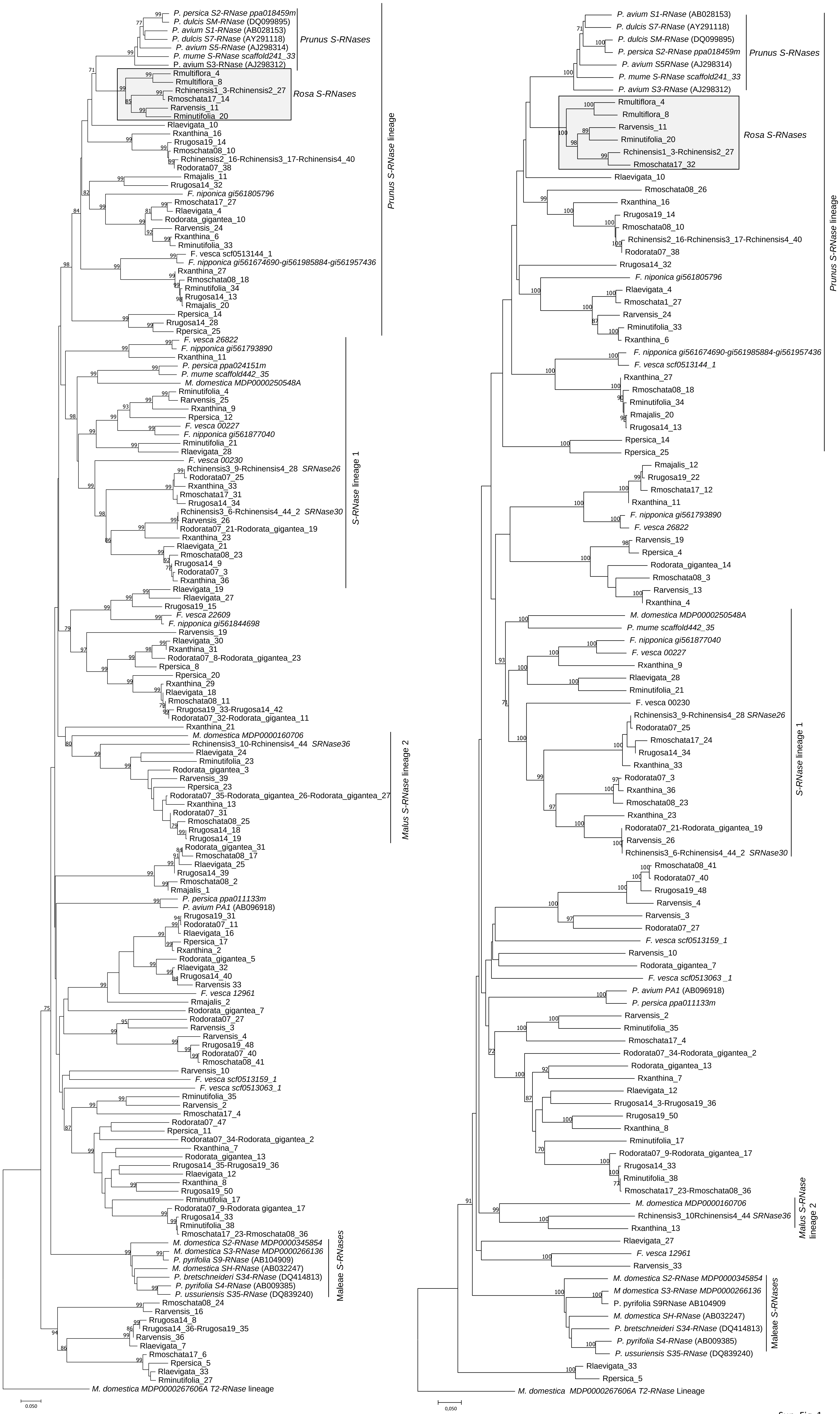

P. persica S2-RNase ppa018459m
P. dulcis SM-RNase (DQ099895)
P. avium S1-RNase (AB028153)
P. dulcis S7-RNase (AY291118)
P. avium S5-RNase (AJ298314)
P. mume S-RNase scaffold241_33
P. avium S3-RNase (AJ298312)
Prunus S-RNases
Rmultiflora_4
Rmultiflora_8
Rchinensis1_3-Rchinensis2_27
Rmoschata17_14
Rarvensis_11
Rminutifolia_20
Rosa S-RNases
Rlaevigata_10
Rxanthina_16
Rrugosa19_14
Rmoschata08_10
Rchinensis2_16-Rchinensis3_17-Rchinensis4_40
Rodorata07_38
Rmajalis_11
Rrugosa14_32
F. niponica gi561805796
Rmoschata17_27
Rlaevigata_4
Rodorata_gigantea_10
Rarvensis_24
Rxanthina_6
Rminutifolia_33
F. vesca scf0513144_1
F. nipponica gi561674690-gi561985884-gi561957436
Rxanthina_27
Rmoschata08_18
Rminutifolia_34
Rrugosa14_13
Rmajalis_20
Rpersica_14
Rrugosa14_28
Rpersica_25
Prunus S-RNase lineage
F. vesca 26822
F. nipponica gi561793890
Rxanthina_11
P. persica ppa024151m
P. mume scaffold442_35
M. domestica MDP0000250548A
Rminutifolia_4
Rarvensis_25
Rxanthina_9
Rpersica_12
F. vesca 00227
F. nipponica gi561877040
Rminutifolia_21
Rlaevigata_28
F. vesca 00230
Rchinensis3_9-Rchinensis4_28 SRNase26
Rodorata07_25
Rxanthina_33
Rmoschata17_31
Rrugosa14_34
Rchinensis3_6-Rchinensis4_44_2 SRNase30
Rarvensis_26
Rodorata07_21-Rodorata_gigantea_19
Rxanthina_23
Rlaevigata_21
Rmoschata08_23
Rrugosa14_9
Rodorata07_3
Rxanthina_36
S-RNase lineage 1
Rlaevigata_19
Rlaevigata_27
Rrugosa19_15
F. vesca 22609
F. nipponica gi561844698
Rarvensis_19
Rlaevigata_30
Rxanthina_31
Rodorata07_8-Rodorata_gigantea_23
Rpersica_8
Rpersica_20
Rxanthina_29
Rlaevigata_18
Rmoschata08_11
Rrugosa19_33-Rrugosa14_42
Rodorata07_32-Rodorata_gigantea_11
Rxanthina_21
M. domestica MDP0000160706
Rchinensis3_10-Rchinensis4_44 SRNase36
Rlaevigata_24
Rminutifolia_23
Rodorata_gigantea_3
Rarvensis_39
Rpersica_23
Rodorata07_35-Rodorata_gigantea_26-Rodorata_gigantea_27
Rxanthina_13
Rodorata07_31
Rmoschata08_25
Rrugosa14_18
Rrugosa14_19
Malus S-RNase lineage 2
Rodorata_gigantea_31
Rmoschata08_17
Rlaevigata_25
Rrugosa14_39
Rmoschata08_2
Rmajalis_1
P. persica ppa011133m
P. avium PA1 (AB096918)
Rrugosa19_31
Rodorata07_11
Rlaevigata_16
Rpersica_17
Rxanthina_2
Rodorata_gigantea_5
Rlaevigata_32
Rrugosa14_40
Rarvensis 33
F. vesca 12961
Rmajalis_2
Rodorata_gigantea_7
Rodorata07_27
Rarvensis_3
Rarvensis_4
Rrugosa19_48
Rodorata07_40
Rmoschata08_41
Rarvensis_10
F. vesca scf0513159_1
F. vesca scf0513063_1
Rminutifolia_35
Rarvensis_2
Rmoschata17_4
Rodorata07_47
Rpersica_11
Rodorata07_34-Rodorata_gigantea_2
Rxanthina_7
Rodorata_gigantea_13
Rrugosa14_35-Rrugosa19_36
Rlaevigata_12
Rxanthina_8
Rrugosa19_50
Rminutifolia_17
Rodorata07_9-Rodorata gigantea_17
Rrugosa14_33
Rminutifolia_38
Rmoschata17_23-Rmoschata08_36
M. domestica S2-RNase MDP0000345854
M. domestica S3-RNase MDP0000266136
P. pyrifolia S9-RNase (AB104909)
M. domestica SH-RNase (AB032247)
P. bretschneideri S34-RNase (DQ414813)
P. pyrifolia S4-RNase (AB009385)
P. ussuriensis S35-RNase (DQ839240)
Maleae S-RNases
Rmoschata08_24
Rarvensis_16
Rrugosa14_8
Rrugosa14_36-Rrugosa19_35
Rarvensis_36
Rlaevigata_7
Rmoschata17_6
Rpersica_5
Rlaevigata_33
Rminutifolia_27
M. domestica MDP0000267606A T2-RNase lineage
0.050
P. avium S1-RNase (AB028153)
P. dulcis S7-RNase (AY291118)
P. dulcis SM-RNase (DQ099895)
P. persica S2-RNase ppa018459m
P. avium S5RNase (AJ298314)
P. mume S-RNase scaffold241_33
P. avium S3-RNase (AJ298312)
Prunus S-RNases
Rmultiflora_4
Rmultiflora_8
Rarvensis_11
Rminutifolia_20
Rchinensis1_3-Rchinensis2_27
Rmoschata17_32
Rosa S-RNases
Rlaevigata_10
Rmoschata08_26
Rxanthina_16
Rrugosa19_14
Rmoschata08_10
Rchinensis2_16-Rchinensis3_17-Rchinensis4_40
Rodorata07_38
Rrugosa14_32
F. niponica gi561805796
Rlaevigata_4
Rmoschata1_27
Rarvensis_24
Rminutifolia_33
Rxanthina_6
F. nipponica gi561674690-gi561985884-gi561957436
F. vesca scf0513144_1
Rxanthina_27
Rmoschata08_18
Rminutifolia_34
Rmajalis_20
Rrugosa14_13
Rpersica_14
Rpersica_25
Prunus S-RNase lineage
Rmajalis_12
Rrugosa19_22
Rmoschata17_12
Rxanthina_11
F. nipponica gi561793890
F. vesca 26822
Rarvensis_19
Rpersica_4
Rodorata_gigantea_14
Rmoschata08_3
Rarvensis_13
Rxanthina_4
M. domestica MDP0000250548A
P. mume scaffold442_35
F. nipponica gi561877040
F. vesca 00227
Rxanthina_9
Rlaevigata_28
Rminutifolia_21
F. vesca 00230
Rchinensis3_9-Rchinensis4_28 SRNase26
Rodorata07_25
Rmoschata17_24
Rrugosa14_34
Rxanthina_33
Rodorata07_3
Rxanthina_36
Rmoschata08_23
Rxanthina_23
Rodorata07_21-Rodorata_gigantea_19
Rarvensis_26
Rchinensis3_6-Rchinensis4_44_2 SRNase30
S-RNase lineage 1
Rmoschata08_41
Rodorata07_40
Rrugosa19_48
Rarvensis_4
Rarvensis_3
Rodorata07_27
F. vesca scf0513159_1
Rarvensis_10
Rodorata_gigantea_7
F. vesca scf0513063 _1
P. avium PA1 (AB096918)
P. persica ppa011133m
Rarvensis_2
Rminutifolia_35
Rmoschata17_4
Rodorata07_34-Rodorata_gigantea_2
Rodorata_gigantea_13
Rxanthina_7
Rlaevigata_12
Rrugosa14_3-Rrugosa19_36
Rrugosa19_50
Rxanthina_8
Rminutifolia_17
Rodorata07_9-Rodorata_gigantea_17
Rrugosa14_33
Rminutifolia_38
Rmoschata17_23-Rmoschata08_36
M. domestica MDP0000160706
Rchinensis3_10Rchinensis4_44 SRNase36
Rxanthina_13
Malus S-RNase lineage 2
Rlaevigata_27
F. vesca 12961
Rarvensis_33
M. domestica S2-RNase MDP0000345854
M domestica S3-RNase MDP0000266136
P. pyrifolia S9RNase AB104909
M. domestica SH-RNase (AB032247)
P. bretschneideri S34-RNase (DQ414813)
P. pyrifolia S4-RNase (AB009385)
P. ussuriensis S35-RNase (DQ839240)
Maleae S-RNases
Rlaevigata_33
Rpersica_5
M. domestica MDP0000267606A T2-RNase Lineage
0,050

A B

Sup. Fig. 1
